# Supplementary material for: DELTA50: A Highly Accurate Database of Experimental 1H and 13C NMR Chemical Shifts Applied to DFT Benchmarking
Source: Molecules. 2023 Mar 7;28(6):2449. doi: 10.3390/molecules28062449 (PMC10051451; doi:10.3390/molecules28062449)
Supplement: Supplementary file 1 [file molecules-28-02449-s001.zip › DELTA50-paper-SI.pdf]

Supporting Information

DELTA50: A Highly Accurate Database of Experimental ^{1}H and ^{13}C NMR Chemical Shifts Applied to DFT Benchmarking

Ryan D. Cohen,[1,2,*] Jared S. Wood,[1,3] Yu-Hong Lam,[1] Alexei V. Buevich,[1] Edward C. Sherer,[1] Mikhail Reibarkh,[1] R. Thomas Williamson,[3] and Gary E. Martin[2]

[1] Merck & Co., Inc., Rahway, NJ 07065, USA
[2] Chemistry and Biochemistry, Seton Hall University, South Orange, NJ 07079, USA
[3] Department of Chemistry and Biochemistry, University of North Carolina Wilmington, Wilmington, NC 28409, USA
* Correspondence: ryan.cohen@merck.com

Table of Contents

I. DFT-Optimized Cartesian Coordinates for DELTA50 Compounds 3

II. Experimental NMR Spectra for DELTA50 Compounds 40

III. Probe Set NMR Data 151

Table S1. Comparison of DFT δ_H and δ_C predictions for **bicyclo[2.1.1]hexan-2-one** 151

Table S2. Comparison of DFT δ_H and δ_C predictions for **α-pinene** 152

Table S3. Comparison of DFT δ_H and δ_C predictions for **aquatolide** 153

Table S4. Comparison of DFT δ_H and δ_C predictions for **naupliolide** 154

Table S5. Comparison of DFT δ_H and δ_C predictions for **echinopine B** 155

Table S6. Comparison of DFT δ_H and δ_C predictions for **parthenolide** 156

Table S7. Comparison of DFT δ_H and δ_C predictions for **diepoxyguainaolide** 157

Table S8. Comparison of DFT δ_H and δ_C predictions for **cannabicitran (CBT-C)** 158

Table S9. Comparison of DFT δ_H and δ_C predictions for **ingenane diterpene 8** 159

Table S10. Comparison of DFT δ_H and δ_C predictions for **artemisinin** 160

Table S11. Comparison of DFT δ_H and δ_C predictions for **nobilisitine A** 161

Table S12. Comparison of DFT δ_H and δ_C predictions for **intricarene** 162

Table S13. Comparison of DFT δ_H and δ_C predictions for **strychnine** 163

Table S14. Comparison of DFT δ_H and δ_C predictions for **holstiine** 164

Table S15. Comparison of DFT δ_H and δ_C predictions for **colchicine** 165

Table S16. Comparison of DFT δ_H and δ_C predictions for **hexacyclinol** 166

Table S17. Comparison of DFT δ_H and δ_C predictions for **homodimericin A** 167
Table S18. Comparison of DFT δ_H predictions for **strychnobaillonine** 168
Table S19. Comparison of DFT δ_C predictions for **strychnobaillonine** 169
Table S20. Comparison of DFT δ_H predictions for **sungucine** 170
Table S21. Comparison of DFT δ_C predictions for **sungucine** 171
Table S22. Comparison of DFT δ_H and δ_C predictions for **paclitaxel** 172
Table S22 (continued). Comparison of DFT δ_H and δ_C predictions for **paclitaxel** 173
Table S23. Comparison of DFT δ_H and δ_C predictions for **sungucine** versus its 5′-epimer 174
IV. Homodimericin A NMR Spectra 175
V. Probe Set Geometries 182
VI. Density Functionals and Basis Sets Employed 275
VII. References 279

I. DFT-Optimized Cartesian Coordinates for DELTA50 Compounds

Optimized geometries *in vacuo* at the B3LYP/6-31G(d) level from Gaussian 16, Revision C.01 [1] for each compound in the DELTA50 training set are provided below. Optimized coordinates of the dominant conformer (≥ 98%) are provided. Experimental proton and carbon chemical shifts were referenced to TMS at 0.00 ppm (see acquired spectra in Section II).

For conformer determinations and Boltzmann weightings, a mixed torsional, low-mode sampling search in MacroModel was performed with the OPLS4 force field [2], as implemented in Schrodinger software suite, version 2021-1 [3]. DFT Gibbs free energies were then calculated at the level of M06-2X/6-31+G(d,p) including the SMD solvent model for chloroform. The Boltzmann probabilities were calculated using the following equation.

$$p_i = \frac{e^{-\varepsilon_i/kT}}{\sum_{j=1}^{M} e^{-\varepsilon_j/kT}}$$

where p_i is the probability of the ith state
ε_i is the energy of the ith state
k is the Boltzmann constant
T is temperature
M is the number of states

Compound 1: **Nitromethane**

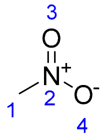

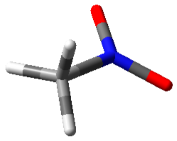

Atom #	Carbon Attachment	Atom	Calculated Atomic Coordinates x	y	z	Experimental δ (ppm)
1	-	C	-1.325474	-0.000005	-0.002538	62.49
2	-	N	0.174089	0.000000	-0.011776	-
3	-	O	0.731302	1.092955	0.003254	-
4	-	O	0.731302	-1.092950	0.003254	-
5	C1	H	-1.665785	-0.906616	-0.499708	4.33
6	C1	H	-1.665789	0.906569	-0.499774	4.33
7	C1	H	-1.635114	0.000035	1.045083	4.33

Compound 2: **Nitroethane**

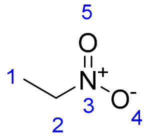

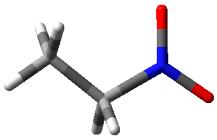

Atom #	Carbon Attachment	Atom	Calculated Atomic Coordinates x	y	z	Experimental δ (ppm)
1	-	C	-1.878328	0.098482	-0.000511	12.31
2	-	C	-0.627705	-0.758597	0.000661	70.47
3	-	N	0.654059	0.052770	0.000123	-
4	-	O	1.697683	-0.592331	-0.000557	-
5	-	O	0.570845	1.276342	0.000288	-
6	C1	H	-2.754961	-0.558268	-0.000005	1.59
7	C1	H	-1.922726	0.738517	0.884010	1.59
8	C1	H	-1.922383	0.736632	-0.886423	1.59
9	C2	H	-0.544965	-1.399815	-0.880815	4.42
10	C2	H	-0.545407	-1.397857	0.883625	4.42

Compound 3: **Acetaldehyde**

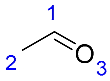

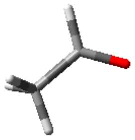

Atom #	Carbon Attachment	Atom	Calculated Atomic Coordinates x	y	z	Experimental δ (ppm)
1	-	C	0.234239	0.399962	-0.000002	199.97
2	-	C	-1.171113	-0.148450	0.000001	30.99
3	-	O	1.237659	-0.277271	0.000001	-
4	C1	H	0.302577	1.512156	0.000003	9.80
5	C2	H	-1.713937	0.218871	-0.880943	2.21
6	C2	H	-1.154729	-1.240768	-0.000028	2.21
7	C2	H	-1.713936	0.218830	0.880963	2.21

Compound 4: **Oxirane**

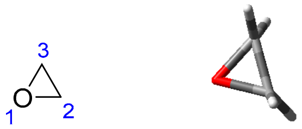

Atom #	Carbon Attachment	Atom	Calculated Atomic Coordinates x	y	z	Experimental δ (ppm)
1	-	O	0.000001	0.855353	0.000000	-
2	-	C	-0.734442	-0.371867	-0.000001	40.94
3	-	C	0.734441	-0.371867	0.000001	40.94
4	C3	H	1.274772	-0.595107	0.920457	2.69
5	C3	H	1.274774	-0.595106	-0.920455	2.69
6	C2	H	-1.274772	-0.595105	-0.920458	2.69
7	C2	H	-1.274775	-0.595106	0.920454	2.69

Compound 5: **Acetonitrile**

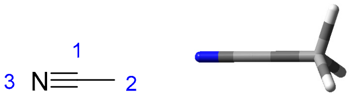

Atom #	Carbon Attachment	Atom	Calculated Atomic Coordinates x	y	z	Experimental δ (ppm)
1	-	C	0.280559	0.000022	0.000032	116.33
2	-	C	-1.181127	-0.000005	-0.000006	1.91
3	-	N	1.440866	-0.000010	-0.000013	-
4	C2	H	-1.560884	-0.950715	0.387217	2.01
5	C2	H	-1.560927	0.810703	0.629685	2.01
6	C2	H	-1.560842	0.139978	-1.016971	2.01

Compound 6: **Cyclopropane**

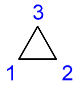

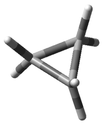

Atom #	Carbon Attachment	Atom	Calculated Atomic Coordinates x	y	z	Experimental δ (ppm)
1	-	C	-0.492427	-0.718224	0.000003	-3.15
2	-	C	-0.375776	0.785563	-0.000007	-3.15
3	-	C	0.868266	-0.067308	0.000002	-3.15
4	C1	H	-0.827012	-1.206263	0.911497	0.25
5	C1	H	-0.827011	-1.206266	-0.911490	0.25
6	C3	H	1.458202	-0.113027	-0.911499	0.25
7	C3	H	1.458179	-0.113014	0.911519	0.25
8	C2	H	-0.631365	1.319196	0.911510	0.25
9	C2	H	-0.631371	1.319189	-0.911526	0.25

Compound 7: **Acetone**

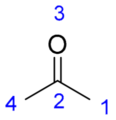

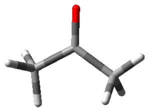

Atom #	Carbon Attachment	Atom	Calculated Atomic Coordinates x	y	z	Experimental δ (ppm)
1	-	C	1.293088	-0.614813	0.000004	30.94
2	-	C	-0.000003	0.185303	0.000026	206.93
3	-	O	0.000030	1.400974	-0.000010	-
4	-	C	-1.293122	-0.614774	-0.000013	30.94
5	C1	H	-1.341564	-1.267122	0.881099	2.17
6	C1	H	-2.148522	0.063608	-0.000211	2.17
7	C1	H	-1.341318	-1.267479	-0.880857	2.17
8	C4	H	1.341193	-1.267740	0.880691	2.17
9	C4	H	1.341663	-1.266896	-0.881290	2.17
10	C4	H	2.148524	0.063541	0.000546	2.17

Compound 8: **Oxetane**

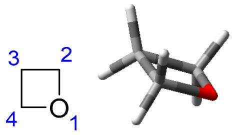

Atom #	Carbon Attachment	Atom	Calculated Atomic Coordinates x	y	z	Experimental δ (ppm)
1	-	O	0.000000	-1.072579	0.000125	-
2	-	C	1.036814	-0.064801	-0.000084	72.55
3	-	C	-0.000000	1.077698	0.000082	22.35
4	-	C	-1.036814	-0.064801	-0.000084	72.55
5	C2	H	1.672620	-0.132539	0.892519	4.76
6	C2	H	1.672232	-0.132514	-0.892972	4.76
7	C3	H	-0.000000	1.710902	0.890879	2.70
8	C3	H	-0.000000	1.711261	-0.890459	2.70
9	C4	H	-1.672620	-0.132539	0.892519	4.76
10	C4	H	-1.672232	-0.132514	-0.892972	4.76

Compound 9: **Methyl acetate**

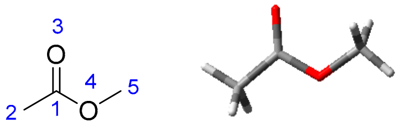

Atom #	Carbon Attachment	Atom	Calculated Atomic Coordinates[a] x	y	z	Experimental δ (ppm)
1	-	C	0.000000	0.493825	0.000000	171.55
2	-	C	-1.133612	1.492846	0.000000	20.71
3	-	O	1.180758	0.763097	0.000000	-
4	-	O	-0.476542	-0.773738	0.000000	-
5	-	C	0.523356	-1.804412	0.000000	51.62
6	C2	H	-1.765505	1.345823	0.882046	2.06
7	C2	H	-0.725713	2.504101	0.000000	2.06
8	C2	H	-1.765505	1.345823	-0.882046	2.06
9	C5	H	-0.026169	-2.746405	0.000000	3.67
10	C5	H	1.155351	-1.728879	-0.889147	3.67
11	C5	H	1.155351	-1.728879	0.889147	3.67

Compound 10: **N,N-Dimethylformamide (DMF)**

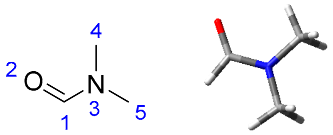

Atom #	Carbon Attachment	Atom	Calculated Atomic Coordinates x	y	z	Experimental δ (ppm)
1	-	C	0.697924	-0.825549	0.000000	162.52
2	-	O	0.209607	-1.943285	0.000000	-
3	-	N	0.000000	0.348854	0.000000	-
4	-	C	0.665195	1.635661	0.000000	31.44
5	-	C	-1.452628	0.332528	0.000000	36.48
6	C1	H	1.792762	-0.653290	0.000000	8.02
7	C4	H	0.394574	2.221023	0.889346	2.89
8	C4	H	1.749320	1.489175	0.000000	2.89
9	C4	H	0.394574	2.221023	-0.889346	2.89
10	C5	H	-1.846975	0.839609	-0.890461	2.96
11	C5	H	-1.777086	-0.708689	0.000000	2.96
12	C5	H	-1.846975	0.839609	0.890461	2.96

Compound 11: **Propionitrile**

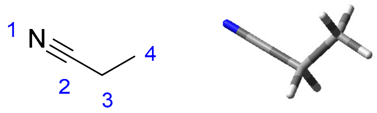

Atom #	Carbon Attachment	Atom	Calculated Atomic Coordinates x	y	z	Experimental δ (ppm)
1	-	N	-1.370404	-1.429413	0.000000	-
2	-	C	-0.773054	-0.434092	0.000000	120.68
3	-	C	0.000000	0.812731	0.000000	10.92
4	-	C	-1.519806	0.567322	0.000000	10.48
5	C3	H	-0.293533	1.398433	0.879652	2.36
6	C3	H	-0.293533	1.398433	-0.879652	2.36
7	C2	H	2.048163	1.525644	0.000000	1.30
8	C2	H	1.825611	0.003809	0.886224	1.30
9	C2	H	1.825611	0.003809	-0.886224	1.30

Compound 12: **Isoxazole**

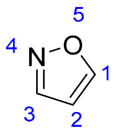

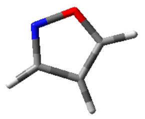

Atom #	Carbon Attachment	Atom	Calculated Atomic Coordinates x	y	z	Experimental δ (ppm)
1	-	C	0.000000	1.127837	0.000000	157.64
2	-	C	1.128139	0.368535	0.000000	103.47
3	-	C	0.617674	-0.960436	0.000000	149.02
4	-	N	-0.694334	-0.995568	0.000000	-
5	-	O	-1.093882	0.345149	0.000000	-
6	C1	H	-0.184497	0.345149	0.000000	8.48
7	C2	H	2.156947	2.192680	0.000000	6.38
8	C3	H	1.164069	-1.895339	0.000000	8.31

Compound 13: **Isobutylene (2-Methylpropene)**

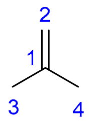

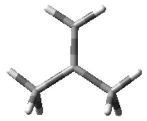

Atom #	Carbon Attachment	Atom	Calculated Atomic Coordinates x	y	z	Experimental δ (ppm)
1	-	C	0.000000	-0.000000	0.122960	142.45
2	-	C	0.000000	-0.000000	1.459489	110.47
3	-	C	0.000000	1.278083	-0.678954	24.13
4	-	C	-0.000000	-1.278083	-0.678954	24.13
5	C2	H	0.000000	-0.924634	2.031659	4.66
6	C2	H	0.000000	0.924634	2.031659	4.66
7	C3	H	0.000000	2.163190	-0.035443	1.73
8	C3	H	0.880040	1.332255	-1.334921	1.73
9	C3	H	-0.880040	1.332255	-1.334921	1.73
10	C4	H	-0.000000	-2.163190	-0.035443	1.73
11	C4	H	-0.880040	-1.332255	-1.334921	1.73
12	C4	H	0.880040	-1.332255	-1.334921	1.73

Compound 14: **2-Butyne**

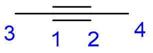

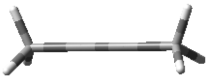

Atom #	Carbon Attachment	Atom	Calculated Atomic Coordinates x	y	z	Experimental δ (ppm)
1	-	C	0.000000	0.000000	0.604587	74.54
2	-	C	0.000000	0.000000	-0.604587	74.54
3	-	C	0.000000	0.000000	2.066081	3.37
4	-	C	0.000000	0.000000	-2.066081	3.37
5	C3	H	0.000000	1.021502	2.466253	1.75
6	C3	H	-0.884647	-0.510751	2.466253	1.75
7	C3	H	0.884647	-0.510751	2.466253	1.75
8	C4	H	0.000000	1.021502	-2.466253	1.75
9	C4	H	0.884647	-0.510751	-2.466253	1.75
10	C4	H	-0.884647	-0.510751	-2.466253	1.75

Compound 15: **t-Butyl nitrate (2-Methyl-2-nitropropane)**

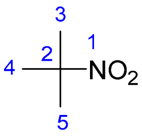

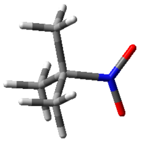

Atom #	Carbon Attachment	Atom	Calculated Atomic Coordinates x	y	z	Experimental δ (ppm)
1	-	N	-0.146964	-0.975496	0.000000	-
2	-	C	0.063658	0.559662	0.000000	85.06
3	-	C	-1.299721	1.246854	0.000000	27.87
4	-	C	0.866281	0.878877	1.268433	27.87
5	-	C	0.866281	0.878877	-1.268433	27.87
6	C3	H	-1.143931	2.331156	0.000000	1.62
7	C3	H	-1.883032	0.980536	-0.884879	1.62
8	C3	H	-1.883032	0.980536	0.884879	1.62
9	C4	H	1.069422	1.954161	1.303318	1.62
10	C4	H	0.303440	0.609249	2.168502	1.62
11	C4	H	1.816375	0.340115	1.273084	1.62
12	C5	H	1.069422	1.954161	-1.303318	1.62
13	C5	H	0.303440	0.609249	-2.168502	1.62
14	C5	H	1.816375	0.340115	-1.273084	1.62
15	-	O	-1.293622	-1.411438	0.000000	-
16	-	O	0.866281	-1.670614	0.000000	-

Compound 16: **Tetrahydrofuran (THF)**

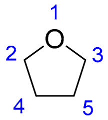

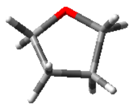

Atom #	Carbon Attachment	Atom	Calculated Atomic Coordinates x	y	z	Experimental δ (ppm)
1	-	O	0.000000	-0.000000	1.251442	-
2	-	C	0.000000	1.172681	0.430760	67.99
3	-	C	-0.000000	-1.172681	0.430760	67.99
4	-	C	-0.307936	0.703722	-0.996748	25.62
5	-	C	0.307936	-0.703722	-0.996748	25.62
6	C2	H	-0.742265	1.877577	0.823672	3.75
7	C3	H	0.742265	-1.877577	0.823672	3.75
8	C3	H	0.987905	1.656495	0.483033	3.75
9	C2	H	-0.987905	-1.656495	0.483033	3.75
10	C4	H	-1.391540	0.644345	-1.155142	1.85
11	C5	H	1.391540	-0.644345	-1.155142	1.85
12	C4	H	0.110732	1.365421	-1.761405	1.85
13	C5	H	-0.110732	-1.365421	-1.761405	1.85

Compound 17: **N,N-Dimethylacetamide (DMAc)**

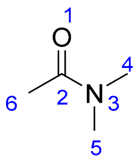

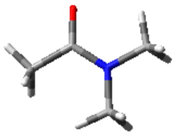

Atom #	Carbon Attachment	Atom	Calculated Atomic Coordinates x	y	z	Experimental δ (ppm)
1	-	O	1.067377	-1.472559	0.000130	-
2	-	C	0.728849	-0.293308	-0.000029	170.66
3	-	N	-0.596288	0.083564	-0.000285	-
4	-	C	-1.623986	-0.946085	-0.000013	35.20
5	-	C	-1.083783	1.450223	0.000127	38.05
6	-	C	1.778984	0.813138	-0.000038	21.58
7	C4	H	-1.133732	-1.918451	-0.000364	2.94
8	C4	H	-2.261301	-0.855378	0.890146	2.94
9	C4	H	-2.262012	-0.855052	-0.889618	2.94
10	C5	H	-0.261812	2.164327	-0.000763	3.01
11	C5	H	-1.704698	1.640728	-0.886873	3.01
12	C5	H	-1.703029	1.640949	0.888265	3.01
13	C6	H	2.754921	0.326973	0.000021	2.08
14	C6	H	1.703172	1.453769	-0.886055	2.08
15	C6	H	1.703108	1.453846	0.885917	2.08

Compound 18: **Cyclobutanone**

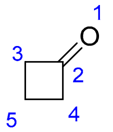

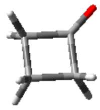

Atom #	Carbon Attachment	Atom	Calculated Atomic Coordinates x	y	z	Experimental δ (ppm)
1	-	O	-0.000000	0.000000	1.883380	-
2	-	C	-0.000000	0.000000	0.680062	209.40
3	-	C	-0.000000	1.111393	-0.385557	47.74
4	-	C	-0.000000	-1.111393	-0.385557	47.74
5	-	C	0.000000	0.000000	-1.480192	9.71
6	C3	H	-0.887021	1.754218	-0.350761	3.09
7	C3	H	0.887021	1.754218	-0.350761	3.09
8	C4	H	-0.887021	-1.754218	-0.350761	3.09
9	C4	H	0.887021	-1.754218	-0.350761	3.09
10	C5	H	-0.887077	0.000000	-2.118265	2.01
11	C5	H	0.887077	-0.000000	-2.118265	2.01

Compound 19: **Butyrolactone**

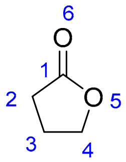

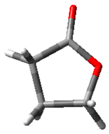

Atom #	Carbon Attachment	Atom	Calculated Atomic Coordinates x	y	z	Experimental δ (ppm)
1	-	C	0.889487	-0.001520	0.003368	177.65
2	-	C	-0.026980	1.209424	0.169334	27.80
3	-	C	-1.406468	0.669083	-0.219782	22.20
4	-	C	-1.269380	-0.822646	0.127783	68.48
5	-	O	0.127216	-1.134993	-0.043504	-
6	-	O	2.089064	-0.028869	-0.072118	-
7	C2	H	0.012392	1.522563	1.220723	2.50
8	C2	H	0.334092	2.042919	-0.436677	2.50
9	C3	H	-2.237210	1.144067	0.308940	2.27
10	C3	H	-1.574055	0.791455	-1.295294	2.27
11	C4	H	-1.545750	-1.029880	1.169152	4.35
12	C4	H	-1.839665	-1.486270	-0.526076	4.35

Compound 20: **Isobutyronitrile (2-Cyanopropane)**

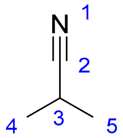

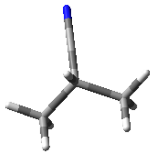

Atom #	Carbon Attachment	Atom	Calculated Atomic Coordinates x	y	z	Experimental δ (ppm)
1	-	N	-0.405239	-2.185681	0.000000	-
2	-	C	-0.026629	-1.087975	0.000000	123.76
3	-	C	0.438658	0.309211	-0.000000	19.84
4	-	C	-0.026629	1.032249	1.279737	19.95
5	-	C	-0.026629	1.032249	-1.279737	19.95
6	C3	H	1.536699	0.274152	0.000000	2.70
7	C4	H	0.325365	0.517215	2.178429	1.33
8	C4	H	0.367828	2.053774	1.288429	1.33
9	C4	H	-1.119520	1.084616	1.320106	1.33
10	C5	H	0.325365	0.517215	-2.178429	1.33
11	C5	H	0.367828	2.053774	-1.288429	1.33
12	C5	H	-1.119520	1.084616	-1.320106	1.33

Compound 21: **Furan**

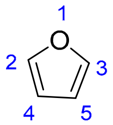

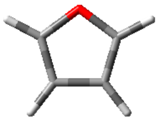

Atom #	Carbon Attachment	Atom	Calculated Atomic Coordinates x	y	z	Experimental δ (ppm)
1	-	O	-0.000000	0.000000	1.161192	-
2	-	C	-0.000000	1.094672	0.347334	142.54
3	-	C	-0.000000	-1.094672	0.347334	142.54
4	-	C	0.000000	0.717590	-0.960054	109.45
5	-	C	-0.000000	-0.717590	-0.960054	109.45
6	C2	H	-0.000000	2.049543	0.850322	7.45
7	C3	H	-0.000000	-2.049543	0.850322	7.45
8	C4	H	0.000000	1.374522	-1.818771	6.40
9	C5	H	-0.000000	-1.374522	-1.818771	6.40

Compound 22: **3-Butyn-2-one**

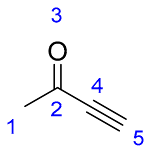

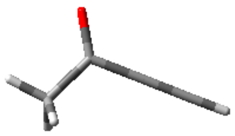

Atom #	Carbon Attachment	Atom	Calculated Atomic Coordinates x	y	z	Experimental δ (ppm)
1	-	C	1.287423	-1.061063	0.000015	32.63
2	-	C	0.452063	0.202176	-0.000033	183.94
3	-	O	0.933957	1.320930	0.000034	-
4	-	C	-0.994215	0.005826	-0.000121	81.84
5	-	C	-2.191020	-0.168654	0.000038	78.10
6	C1	H	1.052982	-1.669866	-0.881556	2.38
7	C1	H	1.053211	-1.669598	0.881837	2.38
8	C1	H	2.346708	-0.795971	-0.000143	2.38
9	C5	H	-3.250066	-0.301720	0.000205	3.21

Compound 23: **Pyrimidine**

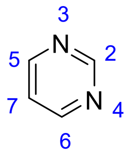

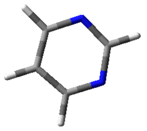

Atom #	Carbon Attachment	Atom	Calculated Atomic Coordinates x	y	z	Experimental δ (ppm)
1	C2	H	-0.000000	-0.000000	-2.397628	9.21
2	-	C	-0.000000	-0.000000	-0.309221	159.10
3	-	N	0.000000	1.199170	-0.716460	-
4	-	N	-0.000000	-1.199170	-0.716460	-
5	-	C	0.000000	1.184371	0.622298	156.94
6	-	C	-0.000000	-1.184371	0.622298	156.94
7	-	C	0.000000	0.000000	1.356463	121.58
8	C5	H	0.000000	2.154249	1.117670	8.76
9	C6	H	-0.000000	-2.154249	1.117670	8.76
10	C7	H	0.000000	0.000000	2.441707	7.34

Compound 24: **1,4-Pyrazine**

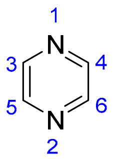

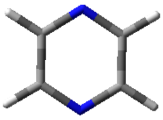

Atom #	Carbon Attachment	Atom	Calculated Atomic Coordinates x	y	z	Experimental δ (ppm)
1	-	N	0.000000	0.000000	1.409502	-
2	-	N	0.000000	0.000000	-1.409502	-
3	-	C	0.000000	1.133170	0.698425	145.18
4	-	C	-0.000000	-1.133170	0.698425	145.18
5	-	C	0.000000	1.133170	-0.698425	145.18
6	-	C	-0.000000	-1.133170	-0.698425	145.18
7	C3	H	0.000000	2.067277	1.257022	8.60
8	C4	H	-0.000000	-2.067277	1.257022	8.60
9	C5	H	0.000000	2.067277	-1.257022	8.60
10	C6	H	-0.000000	-2.067277	-1.257022	8.60

Compound 25: **1,2-Pyrazine (pyridazine)**

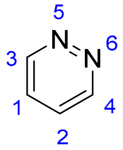

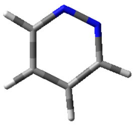

Atom #	Carbon Attachment	Atom	Calculated Atomic Coordinates x	y	z	Experimental δ (ppm)
1	-	C	0.000000	0.691979	1.180878	126.36
2	-	C	-0.000000	-0.691979	1.180878	126.36
3	-	C	0.000000	1.322940	-0.067711	151.72
4	-	C	-0.000000	-1.322940	-0.067711	151.72
5	-	N	0.000000	0.668403	-1.232308	-
6	-	N	-0.000000	-0.668403	-1.232308	-
7	C1	H	0.000000	1.272126	2.099037	7.49
8	C2	H	-0.000000	-1.272126	2.099037	7.49
9	C3		0.000000	2.407140	-0.151885	9.23
10	C4	H	-0.000000	-2.407140	-0.151885	9.23

Compound 26: **Cyclopentane**

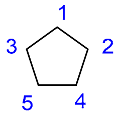

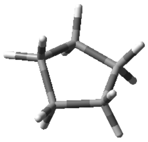

Atom #	Carbon Attachment	Atom	Calculated Atomic Coordinates x	y	z	Experimental δ (ppm)
1	-	C	-0.000000	0.000000	1.308322	25.84
2	-	C	-0.000000	1.243115	0.371790	25.84
3	-	C	-0.000000	-1.243115	0.371790	25.84
4	-	C	-0.328308	0.694836	-1.031133	25.84
5	-	C	0.328308	-0.694836	-1.031133	25.84
6	C1	H	0.877601	0.006551	1.963936	1.50
7	C1	H	-0.877601	-0.006551	1.963936	1.50
8	C2	H	-0.702811	2.017236	0.698224	1.50
9	C2	H	0.995910	1.703363	0.359696	1.50
10	C3	H	0.702811	-2.017236	0.698224	1.50
11	C3	H	-0.995910	-1.703363	0.359696	1.50
12	C4	H	-1.415293	0.589197	-1.152471	1.50
13	C4	H	0.027837	1.344896	-1.838294	1.50
14	C5	H	1.415293	-0.589197	-1.152471	1.50
15	C5	H	-0.027837	1.344896	-1.838294	1.50

Compound 27: **2-Methyl-2-butene**

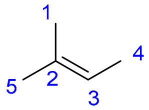

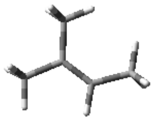

Atom #	Carbon Attachment	Atom	Calculated Atomic Coordinates x	y	z	Experimental δ (ppm)
1	-	C	-1.508735	0.496709	0.000000	17.32
2	-	C	0.000000	0.450978	0.000000	132.10
3	-	C	0.735359	-0.671272	0.000000	118.44
4	-	C	0.267551	-2.099880	0.000000	13.41
5	-	C	0.663899	1.808386	0.000000	25.63
6	C1	H	-1.972994	-0.492122	0.000000	1.60
7	C1	H	-1.880102	1.041295	0.879589	1.60
8	C1	H	-1.880102	1.041295	-0.879589	1.60
9	C3	H	1.820291	-0.552712	0.000000	5.19
10	C4	H	-0.821428	-2.196887	0.000000	1.56
11	C4	H	0.649157	-2.636213	-0.879870	1.56
12	C4	H	0.649157	-2.636213	0.879870	1.56
13	C5	H	1.755677	1.729246	0.000000	1.68
14	C5	H	0.365948	2.396391	-0.879917	1.68
15	C5	H	0.365948	2.396391	0.879917	1.68

Compound 28: **Methyl t-butyl ether (MTBE)**

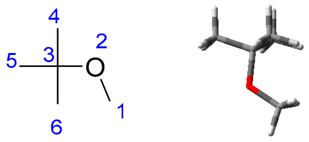

Atom #	Carbon Attachment	Atom	Calculated Atomic Coordinates x	y	z	Experimental δ (ppm)
1	-	C	0.635656	-1.945029	0.000000	49.47
2	-	O	-0.443936	-1.034131	0.000000	-
3	-	C	-0.139565	0.374999	-0.000000	72.79
4	-	C	-1.524158	1.033189	-0.000000	26.99
5	-	C	0.635656	0.773487	1.266946	26.99
6	-	C	0.635656	0.773487	-1.266946	26.99
7	C1	H	0.189192	-2.943662	0.000000	3.21
8	C1	H	1.272407	-1.853184	-0.892175	3.21
9	C1	H	1.272407	-1.853184	0.892175	3.21
10	C4	H	-1.438804	2.125188	-0.000000	1.19
11	C4	H	-2.088966	0.725958	-0.886242	1.19
12	C4	H	-2.088966	0.725958	0.886242	1.19
13	C5	H	0.737202	1.862898	1.324953	1.19
14	C5	H	0.104275	0.429520	2.160987	1.19
15	C5	H	1.645898	0.350169	1.281904	1.19
16	C6	H	0.737202	1.862898	-1.324953	1.19
17	C6	H	0.104275	0.429520	-2.160987	1.19
18	C6	H	1.645898	0.350169	-1.281904	1.19

Compound 29: **Tetrahydropyran (THP)**

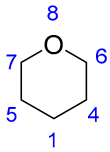

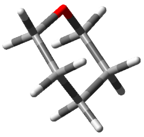

Atom #	Carbon Attachment	Atom	Calculated Atomic Coordinates[a] x	y	z	Experimental δ (ppm)
1	-	C	-0.626248	1.328824	0.000000	23.46
2	C1	H	-1.701325	1.095140	0.000000	1.64
3	C1	H	-0.541876	2.422329	0.000000	1.64
4	-	C	0.020517	0.731339	1.259702	26.60
5	-	C	0.020517	0.731339	-1.259702	26.60
6	-	C	0.020517	-0.797942	1.179649	68.71
7	-	C	0.020517	-0.797942	-1.179649	68.71
8	-	O	0.663440	-1.265666	-0.000000	-
9	C4	H	1.058694	1.078846	1.344361	1.57
10	C4	H	-0.507657	1.055870	2.165785	1.57
11	C5	H	1.058694	1.078846	-1.344361	1.57
12	C5	H	-0.507657	1.055870	-2.165785	1.57
13	C6	H	0.567381	-1.242901	2.016957	3.65
14	C6	H	-1.018037	-1.174735	1.213826	3.65
15	C7	H	0.567381	-1.242901	-2.016957	3.65
16	C7	H	-1.018037	-1.174735	-1.213826	3.65

[a]: Atomic coordinates for chair conformation.

The chair conformation of THP was found to comprise 99.98% of the Boltzmann population. Thus, the boat and twisted boat conformations can be neglected.

Compound 30: **N-Methylpyrrolidine**

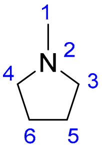

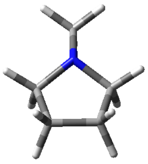

Atom #	Carbon Attachment	Atom	Calculated Atomic Coordinates[a] x	y	z	Experimental δ (ppm)
1	-	C	0.337276	2.099453	-0.000000	42.14
2	-	N	0.446990	0.654778	-0.000000	-
3	-	C	-0.163353	0.003840	-1.157016	56.27
4	-	C	-0.163353	0.003840	0.778520	56.27
5	-	C	-0.163353	-1.489826	-0.778520	24.06
6	-	C	-0.163353	-1.489826	-0.000000	24.06
7	C1	H	-0.713743	2.454949	-0.886306	2.38
8	C1	H	0.833530	2.510891	0.886306	2.38
9	C1	H	0.833530	2.510891	0.886306	2.38
10	C3	H	0.405145	0.224036	2.068209	2.50
11	C3	H	-1.202898	0.357961	1.320962	2.50
12	C4	H	0.405145	0.224036	-2.068209	2.50
13	C4	H	-1.202898	0.357961	-1.320962	2.50
14	C5	H	0.734195	-1.983997	1.162545	1.81
15	C5	H	-1.029164	-2.010529	1.199099	1.81
16	C6	H	0.734195	-1.983997	-1.162545	1.81
17	C6	H	-1.029164	-2.010529	-1.199099	1.81

[a]: Atomic coordinates for equatorial methyl conformation.

N-Methylpyrrolidine exhibits two conformations with the methyl group either equatorial or axial. The equatorial configuration comprises 98.96% of the Boltzmann population, allowing for the axial configuration to effectively be ignored in chemical shift calculations.

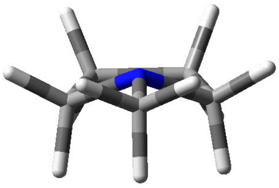

Conformer 1 (98.96%)

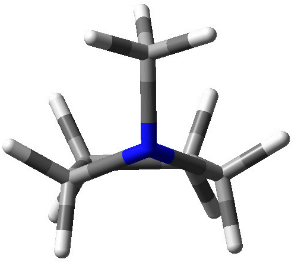

Conformer 2 (1.04%)

Compound 31: **Cyclopentanone**

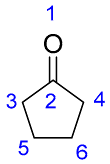

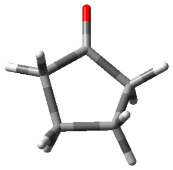

Atom #	Carbon Attachment	Atom	Calculated Atomic Coordinates x	y	z	Experimental δ (ppm)
1	-	O	-0.000000	-0.000000	2.138951	-
2	-	C	-0.000000	-0.000000	0.927928	220.72
3	-	C	-0.000000	1.244707	0.029822	38.37
4	-	C	-0.000000	-1.244707	0.029822	38.37
5	-	C	0.298048	0.713931	-1.382020	23.25
6	-	C	-0.298048	-0.713931	-1.382020	23.25
7	C3	H	-1.005695	1.686525	0.083726	2.17
8	C3	H	0.699264	1.997654	0.405732	2.17
9	C4	H	1.005695	-1.686525	0.083726	2.17
10	C4	H	-0.699264	-1.997654	0.405732	2.17
11	C5	H	-0.115120	1.343317	-2.176147	1.96
12	C5	H	1.383118	0.661440	-1.539712	1.96
13	C6	H	0.115120	-1.343317	-2.176147	1.96
14	C6	H	-1.383118	-0.661440	-1.539712	1.96

Compound 32: **Pivalonitrile**

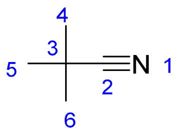

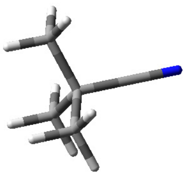

Atom #	Carbon Attachment	Atom	Calculated Atomic Coordinates x	y	z	Experimental δ (ppm)
1	-	N	0.000000	0.000000	2.360404	-
2	-	C	0.000000	0.000000	1.199078	125.94
3	-	C	0.000000	0.000000	-0.279922	28.09
4	-	C	0.000000	1.464540	-0.772873	28.43
5	-	C	1.268329	-0.732270	-0.772873	28.43
6	-	C	-1.268329	-0.732270	-0.772873	28.43
7	C4	H	0.000000	1.481013	-1.868679	1.37
8	C4	H	-0.887101	2.000032	-0.420003	1.37
9	C4	H	0.887101	2.000032	-0.420003	1.37
10	C5	H	1.282595	-0.740506	-1.868679	1.37
11	C5	H	2.175629	-0.231764	-0.420003	1.37
12	C5	H	1.288528	-1.768268	-0.420003	1.37
13	C6	H	-1.282595	-0.740506	-1.868679	1.37
14	C6	H	-1.288528	-1.768268	-0.420003	1.37
15	C6	H	-2.175629	-0.231764	-0.420003	1.37

Compound 33: **Cyclopent-2-en-1-one**

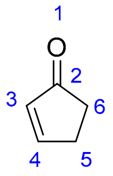

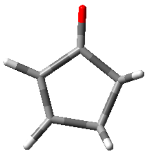

Atom #	Carbon Attachment	Atom	Calculated Atomic Coordinates x	y	z	Experimental δ (ppm)
1	-	O	0.062874	2.101042	0.000000	-
2	-	C	-0.000000	0.886527	0.000000	210.57
3	-	C	-1.226581	0.053604	0.000000	134.63
4	-	C	-0.904837	-1.248423	0.000000	164.76
5	-	C	0.586361	-1.494497	0.000000	28.97
6	-	C	1.199661	-0.079145	0.000000	34.01
7	C3	H	-2.222203	0.482809	0.000000	6.22
8	C4	H	-1.619092	-2.067869	0.000000	7.73
9	C5	H	0.883541	-2.084946	0.876941	2.70
10	C5	H	0.883541	-2.084946	-0.876941	2.70
11	C6	H	1.821800	0.119109	0.879224	2.37
12	C6	H	1.821800	0.119109	-0.879224	2.37

Compound 34: **N-Methylpyrrole**

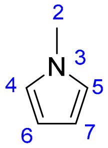

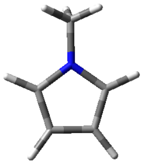

Atom #	Carbon Attachment	Atom	Calculated Atomic Coordinates x	y	z	Experimental δ (ppm)
1	C2	H	1.122656	2.408002	0.000000	3.67
2	-	C	0.077753	2.072793	0.000000	36.08
3	-	N	-0.025623	0.626404	0.000000	-
4	-	C	-0.019442	-0.174768	1.119668	121.66
5	-	C	-0.019442	-0.174768	-1.119668	121.66
6	-	C	-0.019442	-1.491662	0.711854	108.18
7	-	C	-0.019442	-1.491662	-0.711854	108.18
8	C2	H	-0.419796	2.475976	-0.886418	3.67
9	C2	H	-0.419796	2.475976	0.886418	3.67
10	C4	H	-0.018103	0.263657	2.108051	6.61
11	C5	H	-0.018103	0.263657	-2.108051	6.61
12	C6	H	-0.033705	-2.355849	1.362526	6.14
13	C7	H	-0.033705	-2.355849	-1.362526	6.14

Compound 35: **Pyridine**

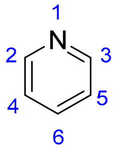

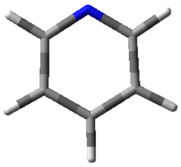

Atom #	Carbon Attachment	Atom	Calculated Atomic Coordinates x	y	z	Experimental δ (ppm)
1	-	N	0.000000	0.000000	1.420887	-
2	-	C	0.000000	1.142176	0.721977	149.74
3	-	C	-0.000000	-1.142176	0.721977	149.74
4	-	C	-0.000000	1.198645	-0.673027	123.78
5	-	C	-0.000000	-1.198645	-0.673027	123.78
6	-	C	-0.000000	0.000000	-1.385530	136.09
7	C2	H	0.000000	2.059822	1.308640	8.62
8	C3	H	-0.000000	-2.059822	1.308640	8.62
9	C4	H	-0.000000	2.157868	-1.182641	7.30
10	C5	H	-0.000000	-2.157868	-1.182641	7.30
11	C6	H	-0.000000	0.000000	-2.472431	7.69

<u>Compound 36</u>: **t-Butylethylene (3,3-Dimethyl-1-butene)**

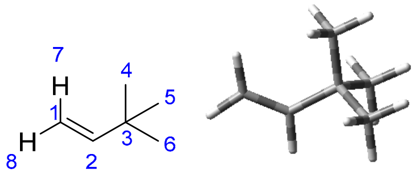

Atom #	Carbon Attachment	Atom	Calculated Atomic Coordinates x	y	z	Experimental δ (ppm)
1	-	C	-0.126415	-2.206499	0.000000	108.86
2	-	C	0.568530	-1.068294	0.000000	149.88
3	-	C	0.032129	0.351659	0.000000	33.66
4	-	C	-1.504297	0.399969	0.000000	29.16
5	-	C	0.568530	1.072457	1.258635	29.16
6	-	C	0.568530	1.072457	-1.258635	29.16
7	C1	H	-1.212586	-2.231006	0.000000	4.91
8	C1	H	0.374902	-3.170922	0.000000	4.83
9	C2	H	1.659520	-1.129168	0.000000	5.86
10	C4	H	-1.851348	1.439804	0.000000	1.01
11	C4	H	-1.920343	-0.091428	-0.886937	1.01
12	C4	H	-1.920343	-0.091428	0.886937	1.01
13	C5	H	0.250199	2.122321	1.268782	1.01
14	C5	H	0.199058	0.594541	2.172973	1.01
15	C5	H	1.664817	1.054964	1.289888	1.01
16	C6	H	0.250199	2.122321	-1.268782	1.01
17	C6	H	0.199058	0.594541	-2.172973	1.01
18	C6	H	1.664817	1.054964	-1.289888	1.01

Compound 37: **Cyclohexane**

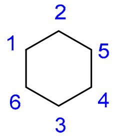

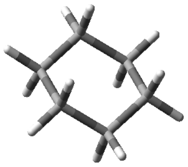

Atom #	Carbon Attachment	Atom	Calculated Atomic Coordinates[a] x	y	z	Experimental δ (ppm)
1	-	C	-1.270575	0.733567	0.229404	26.93
2	-	C	-0.000000	1.467134	-0.229404	26.93
3	-	C	-0.000000	-1.467134	0.229404	26.93
4	-	C	1.270575	-0.733567	-0.229404	26.93
5	-	C	1.270575	0.733567	0.229404	26.93
6	-	C	-1.270575	-0.733567	-0.229404	26.93
7	C1	H	-1.329754	0.767734	1.327713	1.43
8	C2	H	-0.000000	1.535468	-1.327713	1.43
9	C3	H	-0.000000	-1.535468	1.327713	1.43
10	C4	H	1.329754	-0.767734	-1.327713	1.43
11	C5	H	1.329754	0.767734	1.327713	1.43
12	C6	H	-1.329754	-0.767734	-1.327713	1.43
13	C1	H	-2.163615	1.249164	-0.146953	1.43
14	C2	H	-0.000000	2.498328	0.146953	1.43
15	C3	H	-0.000000	-2.498328	-0.146953	1.43
16	C4	H	2.163615	-1.249164	0.146953	1.43
17	C5	H	2.163615	1.249164	-0146953	1.43
18	C6	H	-2.163615	-1.249164	0.146953	1.43

[a]: Atomic coordinates for chair form of cyclohexane (> 99.9% of Boltzmann distribution).

<u>Compound 38</u>: **t-Butylacetylene**

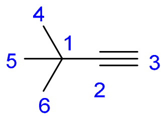

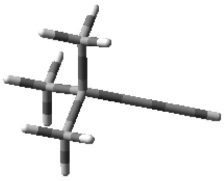

Atom #	Carbon Attachment	Atom	Calculated Atomic Coordinates x	y	z	Experimental δ (ppm)
1	-	C	0.299144	0.000020	0.000000	27.23
2	-	C	-1.174913	0.000162	0.000000	93.21
3	-	C	-2.383181	0.000246	0.000000	66.36
4	-	C	0.808400	1.460302	0.000000	30.88
5	-	C	0.808400	-0.730286	1.264506	30.88
6	-	C	0.808400	-0.730286	-1.264506	30.88
7	C3	H	-3.449350	0.000284	0.000000	2.07
8	C4	H	1.904911	1.475745	0.000000	1.25
9	C4	H	0.456098	1.998231	-0.886296	1.25
10	C4	H	0.456098	1.998231	0.886296	1.25
11	C5	H	1.904875	-0.739966	1.276783	1.25
12	C5	H	0.457933	-0.230491	2.173519	1.25
13	C5	H	0.457933	-1.766265	1.287910	1.25
14	C6	H	1.904875	-0.739966	-1.276783	1.25
15	C6	H	0.454561	-1.766265	-1.287910	1.25
16	C6	H	0.457933	-0.230491	-2.173519	1.25

Compound 39: **Benzene**

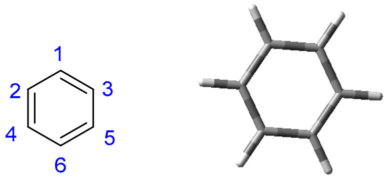

Atom #	Carbon Attachment	Atom	Calculated Atomic Coordinates x	y	z	Experimental δ (ppm)
1	-	C	0.000000	1.396612	0.000000	128.34
2	-	C	1.209501	0.698306	0.000000	128.34
3	-	C	-1.209501	0.698306	0.000000	128.34
4	-	C	1.209501	-0.698306	0.000000	128.34
5	-	C	-1.209501	-0.698306	0.000000	128.34
6	-	C	-0.000000	-1.396612	0.000000	128.34
7	C1	H	0.000000	2.483624	0.000000	7.36
8	C2	H	2.150881	1.241812	0.000000	7.36
9	C3	H	-2.150881	1.241812	0.000000	7.36
10	C4	H	2.150881	-1.241812	0.000000	7.36
11	C5	H	-2.150881	-1.241812	0.000000	7.36
12	C6	H	-0.000000	-2.483624	0.000000	7.36

Compound 40: **N-Methylpiperidine**

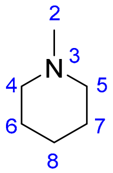

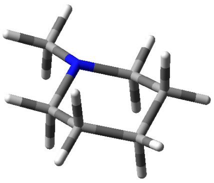

Atom #	Carbon Attachment	Atom	Calculated Atomic Coordinates[a] x	y	z	Experimental δ (ppm)
1	C2	H	0.306115	2.814503	0.000000	2.25
2	-	C	-0.674985	2.297496	0.000000	46.87
3	-	N	-0.562141	0.848381	0.000000	-
4	-	C	0.097724	0.358034	1.209700	56.53
5	-	C	0.097724	0.358034	-1.209700	56.53
6	-	C	0.097724	-1.173280	1.258284	25.96
7	-	C	0.097724	-1.173280	-1.258284	25.96
8	-	C	0.751697	-1.759410	-0.000000	23.73
9	C2	H	-1.228316	2.626094	-0.887079	2.25
10	C2	H	-1.228316	2.626094	0.887079	2.25
11	C4	H	1.144244	0.727945	1.275961	2.35
12	C4	H	-0.436303	0.763048	2.078205	2.35
13	C5	H	1.144244	0.727945	-1.275961	2.35
14	C5	H	-0.436303	0.763048	-2.078205	2.35
15	C6	H	0.620496	-1.510648	2.162182	1.60
16	C6	H	-0.940512	-1.522536	1.331180	1.60
17	C7	H	0.620496	-1.510648	-2.162182	1.60
18	C7	H	-0.940512	-1.522536	-1.331180	1.60
19	C8	H	1.823705	-1.512325	-0.000000	1.42
20	C8	H	0.680300	-2.854217	-0.000000	1.42

[a]: Atomic coordinates are for conformer 1, which represents > 99% of the Boltzmann population.

N-Methylpiperidine exhibits two conformations with the methyl group either equatorial or axial. The equatorial configuration comprises 99.60% of the Boltzmann population, allowing for the axial configuration to be ignored in chemical shift calculations.

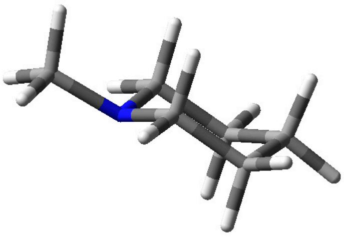

Conformer 1
(99.60%)

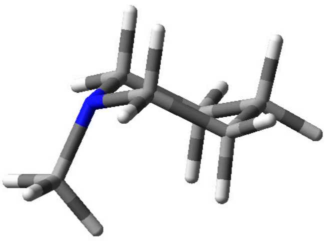

Conformer 2
(0.39%)

Compound 41: **Cyclohexanone**

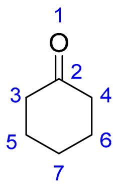

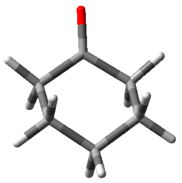

Atom #	Carbon Attachment	Atom	Calculated Atomic Coordinates[a] x	y	z	Experimental δ (ppm)
1	-	O	-1.263609	1.956120	-0.000000	-
2	-	C	-0.413572	1.085927	-0.000000	212.15
3	-	C	0.158071	0.501362	1.286793	42.00
4	-	C	0.158071	0.501362	-1.286793	42.00
5	-	C	0.158071	-1.042001	1.267030	27.03
6	-	C	0.158071	-1.042001	-1.267030	27.03
7	-	C	0.829137	-1.591400	0.000000	25.02
8	C3	H	1.196136	0.856697	1.380658	2.34
9	C3	H	-0.407459	0.903074	2.132693	2.34
10	C4	H	1.196136	0.856697	-1.380658	2.34
11	C4	H	-0.407459	0.903074	-2.132693	2.34
12	C5	H	0.658987	-1.421571	2.165729	1.87
13	C5	H	-0.879451	-1.401316	1.312464	1.87
14	C6	H	0.658987	-1.421571	-2.165729	1.87
15	C6	H	-0.879451	-1.421571	-1.312464	1.87
16	C7	H	1.893839	-1.314426	0.000000	1.72
17	C7	H	0.791517	-2.687790	0.000000	1.72

[a:] Atomic coordinates are for conformer 1, which represents 98.91% of the Boltzmann population.

Cyclohexanone exhibits two conformations, chair and twist-boat. The chair configuration comprises 98.91% of the Boltzmann population, allowing for the twist-boat conformation to be ignored in chemical shift calculations.

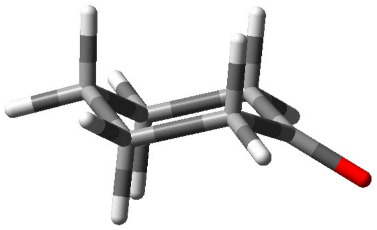

Conformer 1
(98.91%)

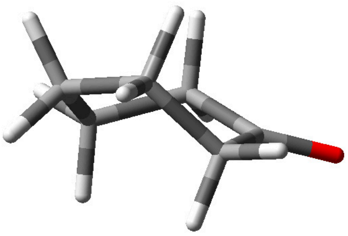

Conformer 2
(1.09%)

Compound 42: **Cyclohex-2-en-1-one (2-Cyclohexenone)**

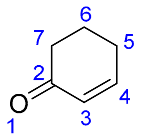

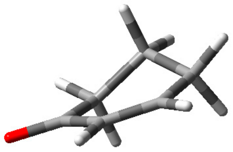

Atom #	Carbon Attachment	Atom	Calculated Atomic Coordinates x	y	z	Experimental δ (ppm)
1	-	O	-2.361295	0.001724	-0.080774	-
2	-	C	-1.143351	0.022365	0.018018	199.74
3	-	C	-0.380622	1.289614	-0.051625	129.99
4	-	C	0.960739	1.326104	0.025453	150.61
5	-	C	1.819221	0.093708	0.131664	25.69
6	-	C	1.075885	-1.163703	-0.342437	22.75
7	-	C	-0.328663	-1.243271	0.271587	38.13
8	C3	H	-0.974066	2.195379	-0.146979	6.03
9	C4	H	1.470878	2.288967	0.014595	7.00
10	C5	H	2.743146	0.236151	-0.443511	2.35
11	C5	H	2.140602	-0.025522	1.179610	2.35
12	C6	H	0.988069	-1.130692	-1.436432	2.03
13	C6	H	1.652501	-2.063136	-0.097731	2.03
14	C7	H	-0.898731	-2.099010	-0.102835	2.44
15	C7	H	-0.251295	-1.364837	1.363507	2.44

Compound 43: **Fluorobenzene**

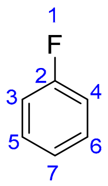

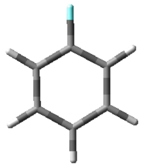

Atom #	Carbon Attachment	Atom	Calculated Atomic Coordinates x	y	z	Experimental δ (ppm)
1	-	F	0.000000	0.000000	2.282591	-
2	-	C	0.000000	0.000000	0.931458	162.86
3	-	C	-0.000000	1.217551	0.260430	115.32
4	-	C	-0.000000	-1.217551	0.260430	115.32
5	-	C	-0.000000	1.208664	-1.135566	129.96
6	-	C	-0.000000	-1.208664	-1.135566	129.96
7	-	C	-0.000000	-0.000000	-1.835837	123.98
8	C3	H	-0.000000	2.142010	0.828608	7.06
9	C4	H	-0.000000	-2.142010	0.828608	7.06
10	C5	H	-0.000000	2.151613	-1.675360	7.34
11	C6	H	-0.000000	-2.151613	-1.675360	7.34
12	C7	H	-0.000000	-0.000000	-2.921902	7.13

Compound 44: **Nitrobenzene**

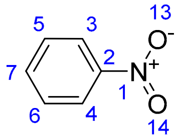

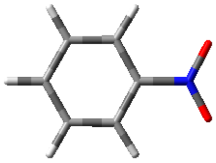

Atom #	Carbon Attachment	Atom	Calculated Atomic Coordinates x	y	z	Experimental δ (ppm)
1	-	N	-0.000000	-0.000000	1.717815	-
2	-	C	-0.000000	-0.000000	0.245092	148.25
3	-	C	0.000000	1.220735	-0.427765	123.52
4	-	C	-0.000000	-1.220735	-0.427765	123.52
5	-	C	0.000000	1.212318	-1.821048	129.31
6	-	C	-0.000000	-1.212318	-1.821048	129.31
7	-	C	0.000000	0.000000	-2.516503	134.56
8	C3	H	0.000000	2.142803	0.140191	8.24
9	C4	H	-0.000000	-2.142803	0.140191	8.24
10	C5	H	0.000000	2.153082	-2.363343	7.56
11	C6	H	-0.000000	-2.153082	-2.363343	7.56
12	C7	H	0.000000	0.000000	-3.602893	7.70
13	-	O	0.000000	1.089518	2.289919	-
14	-	O	-0.000000	-1.089518	2.289919	-

Compound 45: **1,4-Benzoquinone (p-Benzoquinone)**

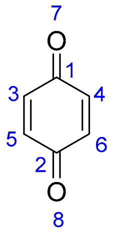

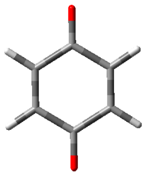

Atom #	Carbon Attachment	Atom	Calculated Atomic Coordinates x	y	z	Experimental δ (ppm)
1	-	C	0.000000	-0.000000	1.445285	187.21
2	-	C	0.000000	-0.000000	-1.445285	187.21
3	-	C	0.000000	1.269144	0.671532	136.56
4	-	C	-0.000000	-1.269144	0.671532	136.56
5	-	C	0.000000	1.269144	-0.671532	136.56
6	-	C	-0.000000	-1.269144	-0.671532	136.56
7	-	O	0.000000	-0.000000	2.670223	-
8	-	O	0.000000	-0.000000	-2.670223	-
9	C3	H	0.000000	2.182717	1.259654	6.79
10	C4	H	-0.000000	-2.182717	1.259654	6.79
11	C5	H	0.000000	2.182717	-1.259654	6.79
12	C6	H	-0.000000	-2.182717	-1.259654	6.79

Compound 46: **Toluene**

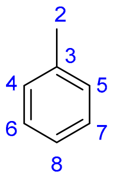

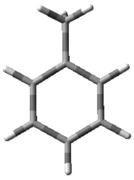

Atom #	Carbon Attachment	Atom	Calculated Atomic Coordinates x	y	z	Experimental δ (ppm)
1	C2	H	1.060485	2.801442	-0.000000	2.36
2	-	C	0.028512	2.425416	-0.000000	21.46
3	-	C	-0.004238	0.913882	-0.000000	137.89
4	-	C	-0.007351	0.194294	1.202312	129.05
5	-	C	-0.007351	0.194294	-1.202312	129.05
6	-	C	-0.007351	-1.201161	1.205396	128.24
7	-	C	-0.007351	-1.201161	-1.205396	128.24
8	-	C	-0.006588	-1.905024	0.000000	125.31
9	C4	H	-0.011732	0.734438	2.146855	7.17
10	C5	H	-0.011732	0.734438	-2.146855	7.17
11	C6	H	-0.012171	-1.738466	2.150420	7.25
12	C7	H	-0.012171	-1.738466	-2.150420	7.25
13	C8	H	-0.009692	-2.991785	0.000000	7.16
14	C2	H	-0.466342	2.837579	-0.886013	2.36
15	C2	H	-0.466342	2.837579	0.886013	2.36

Compound 47: **Norbornadiene (2,5-Norbornadiene)**

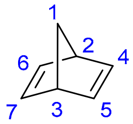

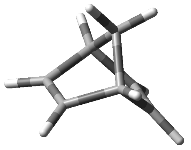

Atom #	Carbon Attachment	Atom	Calculated Atomic Coordinates x	y	z	Experimental δ (ppm)
1	-	C	0.000000	0.000000	1.355167	75.32
2	-	C	0.000000	1.122368	0.271791	50.26
3	-	C	-0.000000	-1.122368	0.271791	50.26
4	-	C	1.245986	0.667933	-0.520792	143.43
5	-	C	1.245986	-0.667933	-0.520792	143.43
6	-	C	-1.245986	0.667933	-0.520792	143.43
7	-	C	-1.245986	-0.667933	-0.520792	143.43
8	C1	H	0.899910	-0.000000	1.979632	1.99
9	C1	H	-0.899910	0.000000	1.979632	1.99
10	C2	H	0.000000	2.159751	0.612341	3.58
11	C3	H	-0.000000	-2.159751	0.612341	3.58
12	C4	H	1.937013	1.338860	-1.019357	6.76
13	C5	H	1.937013	-1.338860	-1.019357	6.76
14	C6	H	-1.937013	1.338860	-1.019357	6.76
15	C7	H	-1.937013	-1.338860	-1.019357	6.76

Compound 48: **Anisole**

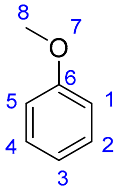

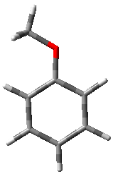

Atom #	Carbon Attachment	Atom	Calculated Atomic Coordinates x	y	z	Experimental δ (ppm)
1	-	C	0.000000	0.529515	0.000000	113.90
2	-	C	-1.373336	0.242655	0.000000	129.45
3	-	C	-1.808034	-1.077795	0.000000	120.65
4	-	C	-0.886000	-2.130483	0.000000	129.45
5	-	C	0.476156	-1.839995	0.000000	113.90
6	-	C	0.930030	-0.516890	0.000000	159.55
7	-	O	0.325395	1.857237	0.000000	-
8	-	C	1.698003	2.211176	0.000000	55.14
9	C1	H	-2.074585	1.071481	0.000000	6.91
10	C2	H	-2.874609	-1.287350	0.000000	7.29
11	C3	H	-1.228212	-3.161259	0.000000	6.95
12	C4	H	1.204690	-2.646844	0.000000	7.29
13	C5	H	1.995503	-0.316937	0.000000	6.91
14	C8	H	1.726725	3.302499	0.000000	3.81
15	C8	H	2.213204	1.835708	0.894422	3.81
16	C8	H	2.213204	1.835708	-0.894422	3.81

Compound 49: **Maleic anhydride**

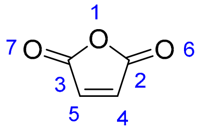

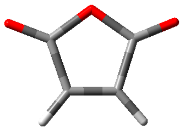

Atom #	Carbon Attachment	Atom	Calculated Atomic Coordinates x	y	z	Experimental δ (ppm)
1	-	O	-0.000730	-0.971804	0.000000	-
2	-	C	-0.000164	-0.158714	1.131991	164.07
3	-	C	-0.000164	-0.158714	-1.131991	164.07
4	-	C	0.000754	1.258255	0.667804	136.49
5	-	C	0.000754	1.258255	-0.667804	136.49
6	-	O	-0.000164	-0.600004	2.245452	-
7	-	O	-0.000164	-0.600004	-2.245452	-
8	C4	H	0.000693	2.089998	1.360223	7.03
9	C5	H	0.000693	2.089998	-1.360223	7.03

Compound 50: **2,5-Dihydrofuran**

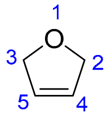

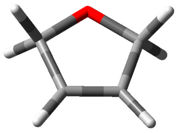

Atom #	Carbon Attachment	Atom	Calculated Atomic Coordinates x	y	z	Experimental δ (ppm)
1	-	O	0.000642	-1.189607	-0.000000	-
2	-	C	-0.000207	-0.371239	1.172196	75.41
3	-	C	-0.000207	-0.371239	-1.172196	75.41
4	-	C	-0.000207	1.045499	0.665433	126.19
5	-	C	-0.000207	1.045499	-0.665433	126.19
6	C2	H	-0.886896	-0.600210	1.785570	4.65
7	C2	H	0.886347	-0.599315	1.786304	4.65
8	C3	H	-0.886896	-0.600210	-1.785570	4.65
9	C3	H	0.886347	-0.599315	-1.786304	4.65
10	C4	H	0.000462	1.912389	1.317664	5.89
11	C5	H	0.000462	1.912389	-1.317664	5.89

II. Experimental NMR Spectra for DELTA50 Compounds

Experimental ^{1}H and ^{13}C NMR spectra for each compound of the DELTA50 test set were acquired at 298 K for ≤ 10 mM samples dissolved in deuterated chloroform, $CDCl_3$, containing 0.03% v/v tetramethylsilane (TMS) for chemical shift reference. Potassium carbonate (K_2CO_3) was used to neutralize residual DCl present in $CDCl_3$. All compounds, solvents, and reagents were at least 98% grade and purchased from Sigma-Aldrich. A 600 MHz Bruker AVANCE III HD NMR spectrometer equipped with a liquid N_2-cooled 5-mm broadband Prodigy™ probe was used for spectral acquisition. Spectra were processed in Mestrelab Research's MestReNova, version 14.2.

For a few compounds (*viz.*, DMF, DMAc, 2-methyl-2-butene, cyclopent-2-en-1-one, N-methylpiperidine, cyclohex-2-en-1-one, and toluene), peak assignments and chemical shifts were not immediately obvious from the 1D spectra. Thus, 1D selective NOESY and 2D NMR experiments were used to clarify any ambiguity. The spectra and processing parameters used were as follows:

- 1D ^{1}H selective gradient **NOESY**: 500 ms mixing time, 32 scans, gauss 180 selective refocussing pulse shape, pulse program *selnogpzs.2* (uses zero-quantum suppression)
- ^{1}H-^{1}H gradient **COSY**: 2 scans, 8 ppm spectral width, 2048 x 128 points, pulse program *cosygpppqf*, sine square 0° apodization in both F1 and F2.
- ^{1}H-^{13}C multiplicity-edited gradient **HSQC**: $^{1}J_{CH}$ delay optimized for 145 Hz, 4 scans, 8 ppm x 160 ppm spectral width, 2048 x 128 points, pulse program *hsqcedetgpsisp2.3*, sine square 90° apodization in both F1 and F2.
- ^{1}H-^{13}C gradient **HMBC**: $^{n}J_{CH}$ delay optimized for 8 Hz, 16 scans, 8 ppm x 210 ppm spectral width, pulse program *hmbcetgpl3nd*, magnitude mode phase correction along F2, sine square 90° apodization in F1 and sine bell 45° apodization in F2.

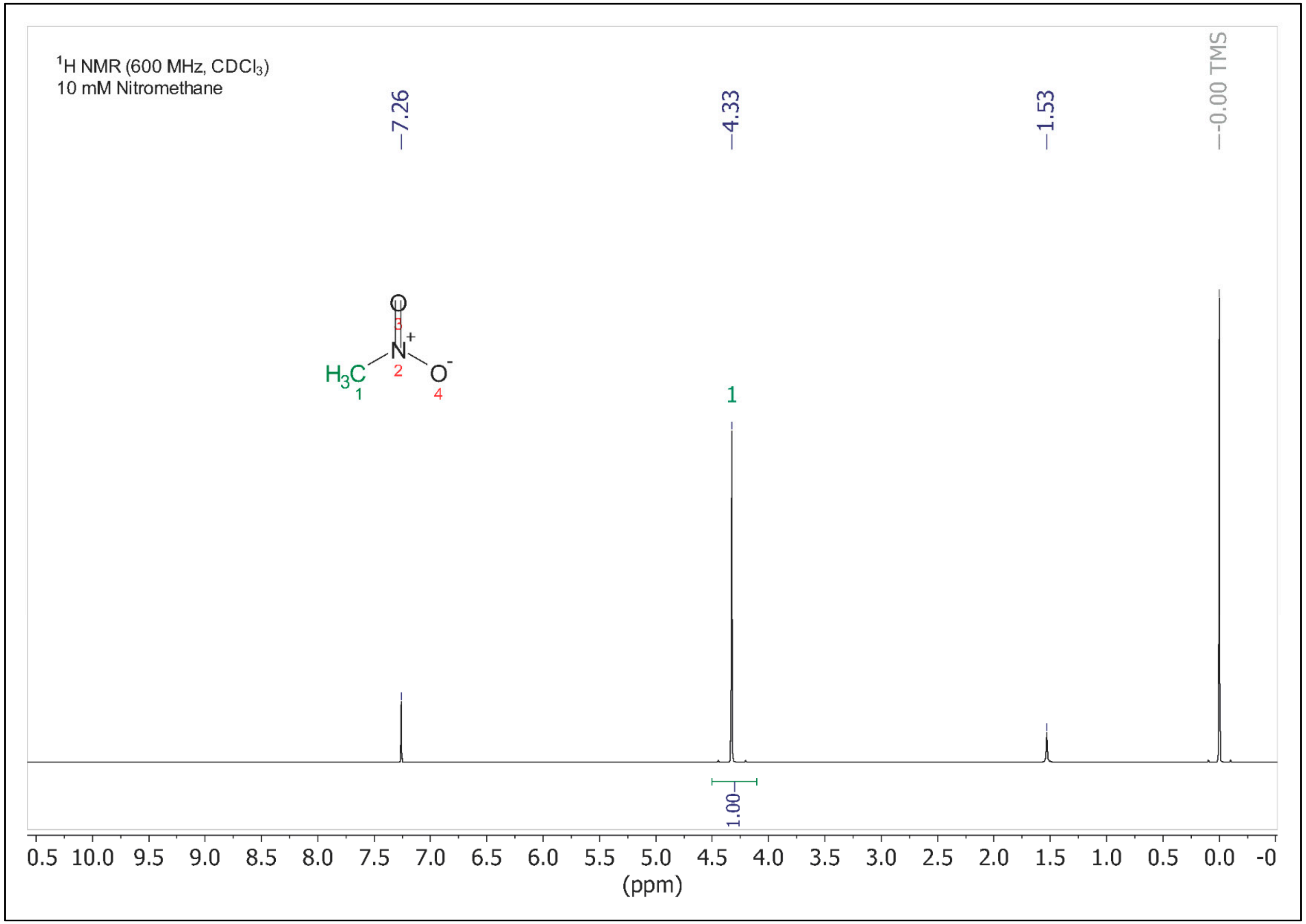

1H NMR (600 MHz, CDCl3)
10 mM Nitromethane
7.26
4.33
1.53
-0.00 TMS
H3C
1
2
3
4
N+
O
O-
1
1.00
0.5 10.0 9.5 9.0 8.5 8.0 7.5 7.0 6.5 6.0 5.5 5.0 4.5 4.0 3.5 3.0 2.5 2.0 1.5 1.0 0.5 0.0 -0
(ppm)

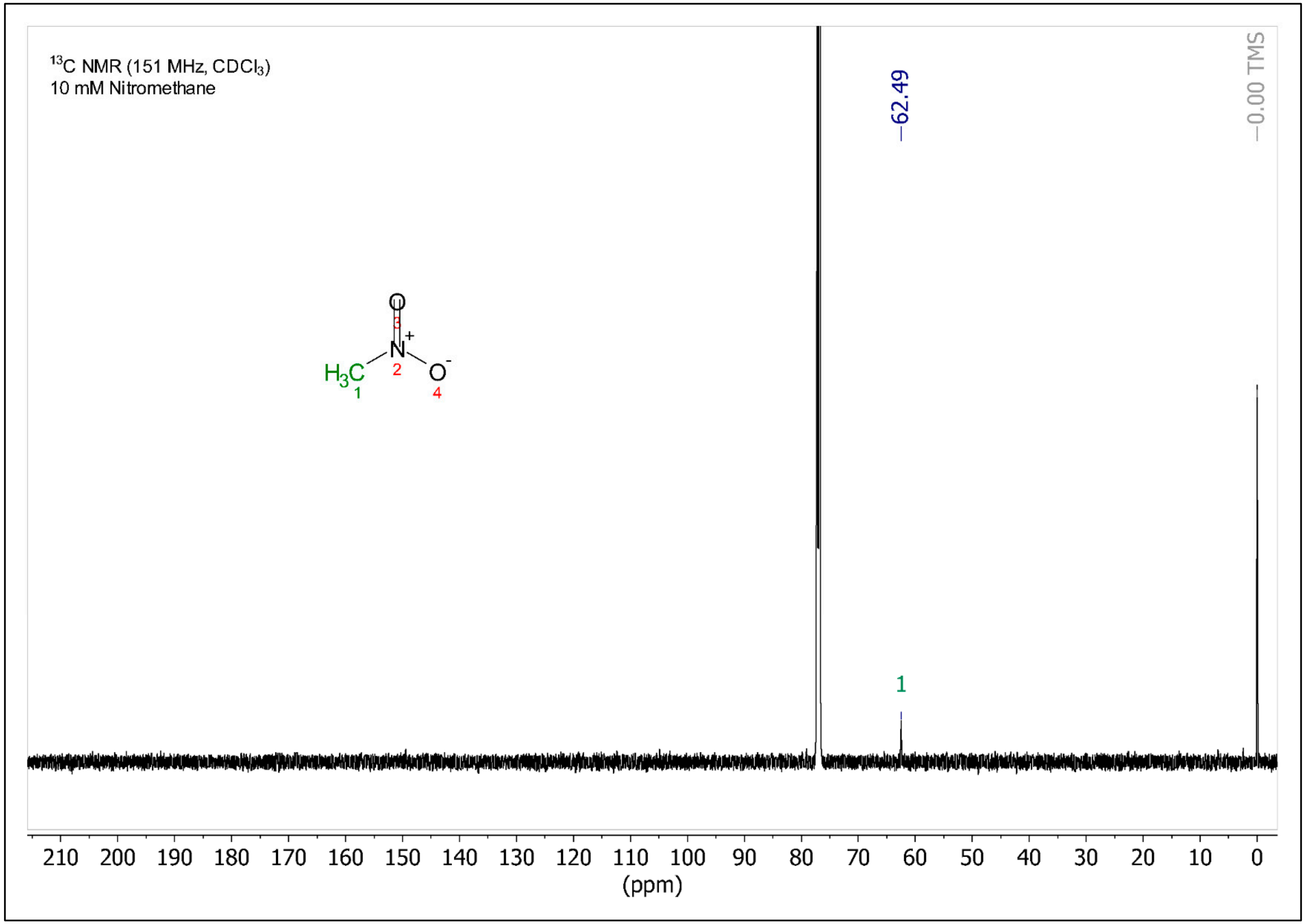

13C NMR (151 MHz, CDCl3)
10 mM Nitromethane
62.49
0.00 TMS
H3C
N
O
O
1
2
3
4
1
210 200 190 180 170 160 150 140 130 120 110 100 90 80 70 60 50 40 30 20 10 0
(ppm)

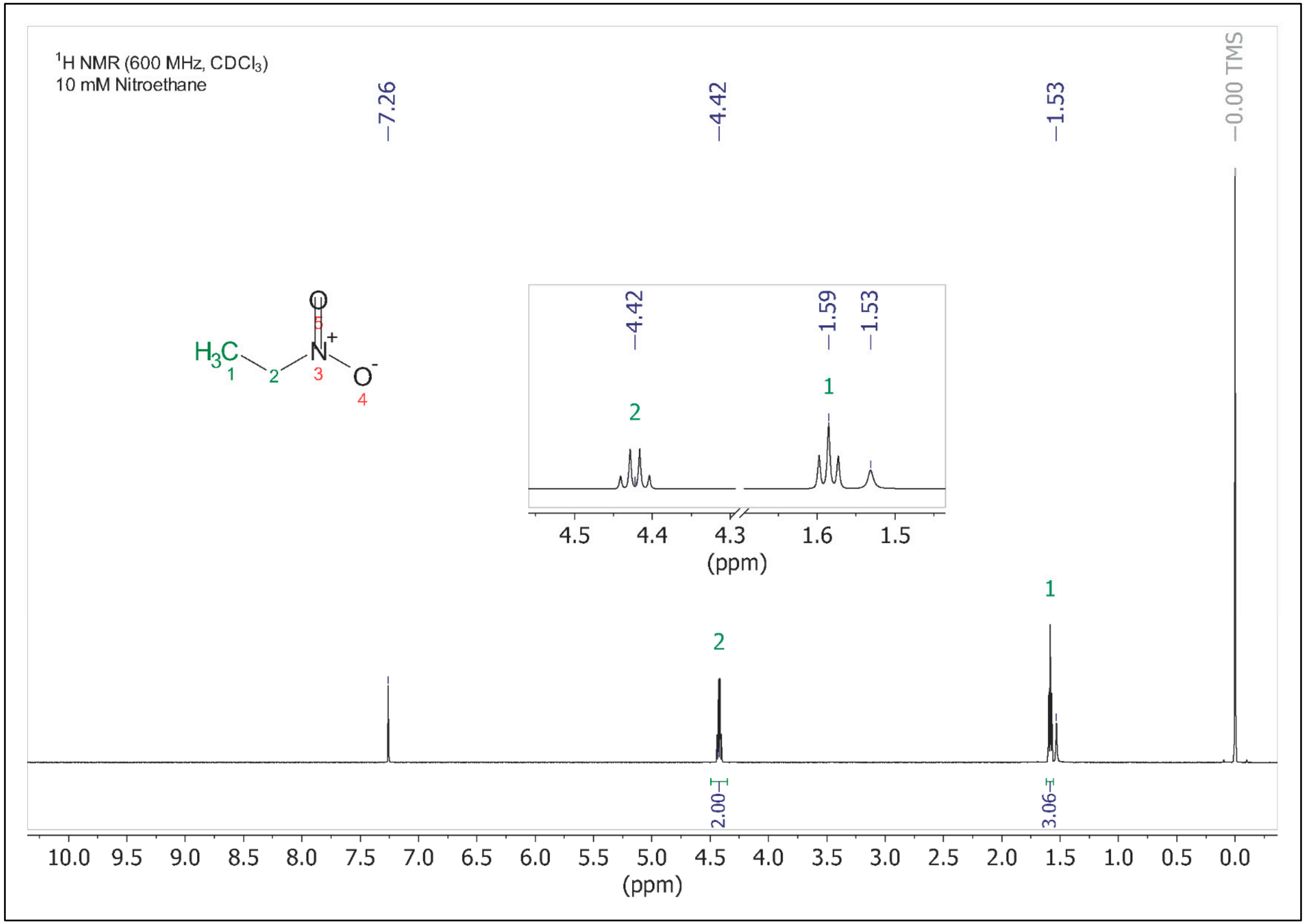

^{1}H NMR (600 MHz, CDCl$_3$)
10 mM Nitroethane
7.26
4.42
1.53
0.00 TMS
4.42
1.59
1.53
1
2
1
2
2.00
3.06
4.5 4.4 4.3 1.6 1.5
(ppm)
10.0 9.5 9.0 8.5 8.0 7.5 7.0 6.5 6.0 5.5 5.0 4.5 4.0 3.5 3.0 2.5 2.0 1.5 1.0 0.5 0.0
(ppm)

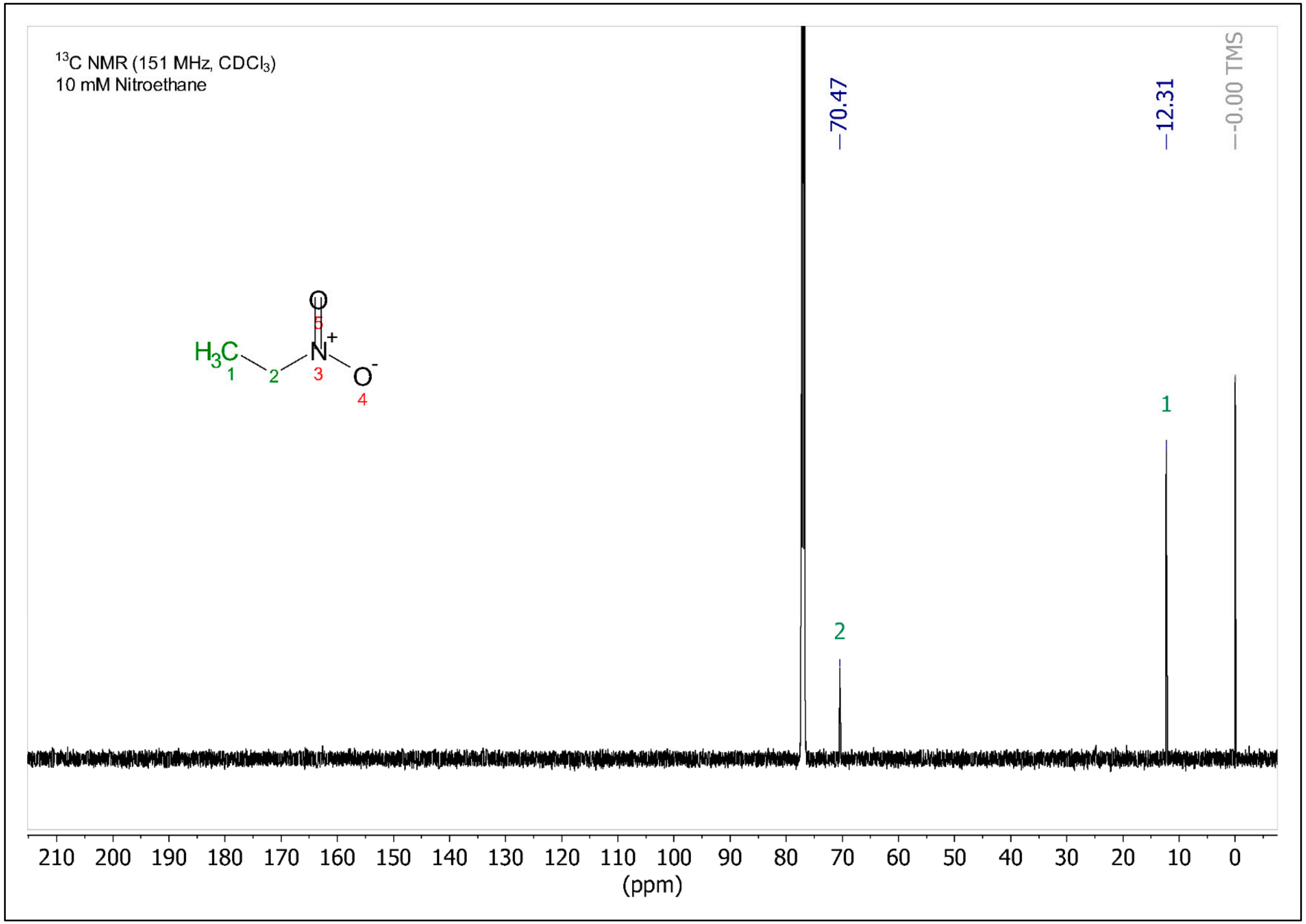

13C NMR (151 MHz, CDCl3)
10 mM Nitroethane
H3C
1
2
3
4
5
N
O
+
-
70.47
12.31
-0.00 TMS
210 200 190 180 170 160 150 140 130 120 110 100 90 80 70 60 50 40 30 20 10 0
(ppm)

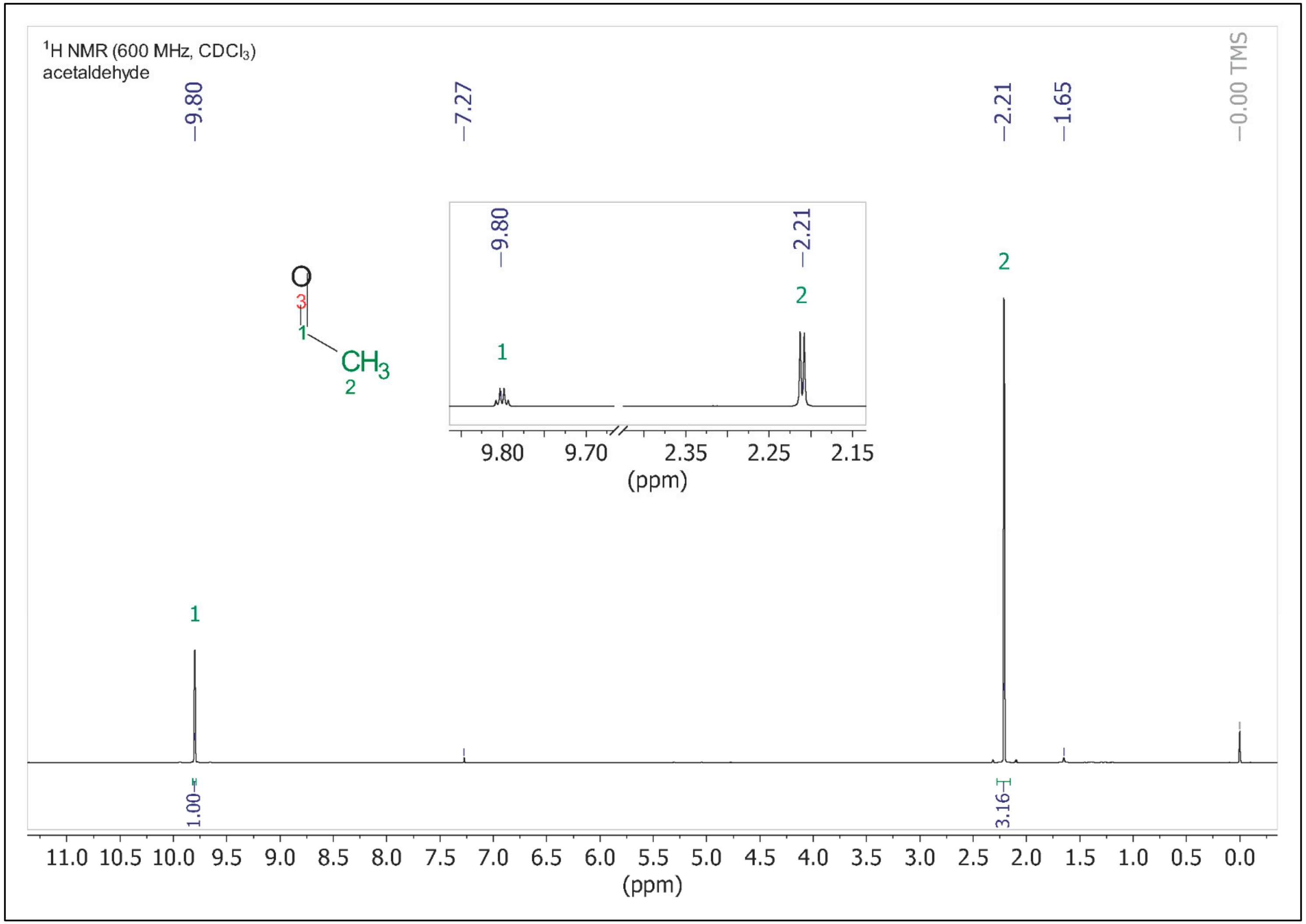

^{1}H NMR (600 MHz, CDCl$_3$)
acetaldehyde
9.80
7.27
2.21
1.65
0.00 TMS
O
3
1
CH$_3$
2
9.80
2.21
1
2
9.80 9.70 2.35 2.25 2.15
(ppm)
2
1
1.00
3.16
11.0 10.5 10.0 9.5 9.0 8.5 8.0 7.5 7.0 6.5 6.0 5.5 5.0 4.5 4.0 3.5 3.0 2.5 2.0 1.5 1.0 0.5 0.0
(ppm)

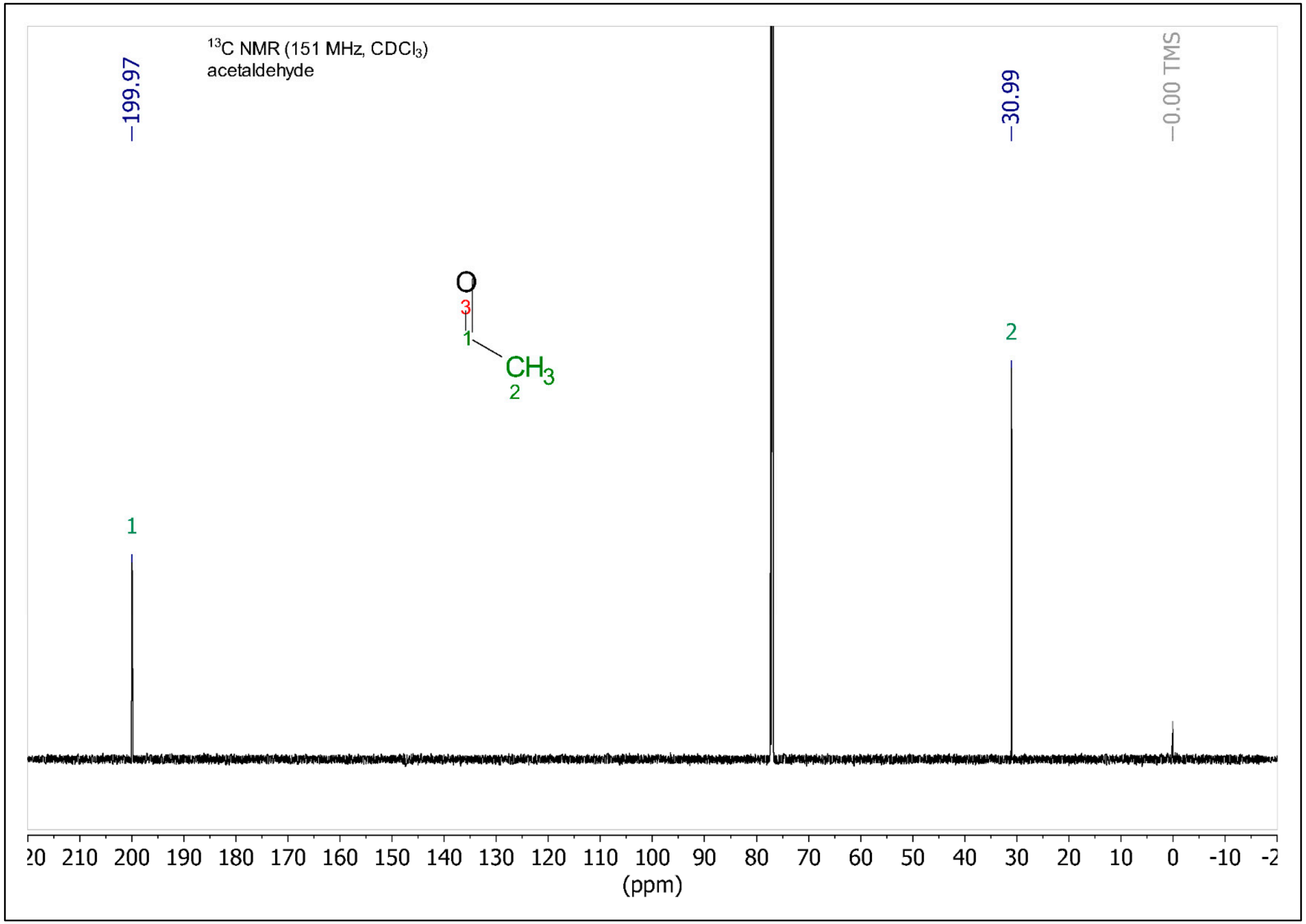

13C NMR (151 MHz, CDCl3)
acetaldehyde
199.97
30.99
0.00 TMS
O
3
1
CH3
2
20 210 200 190 180 170 160 150 140 130 120 110 100 90 80 70 60 50 40 30 20 10 0 -10 -2
(ppm)

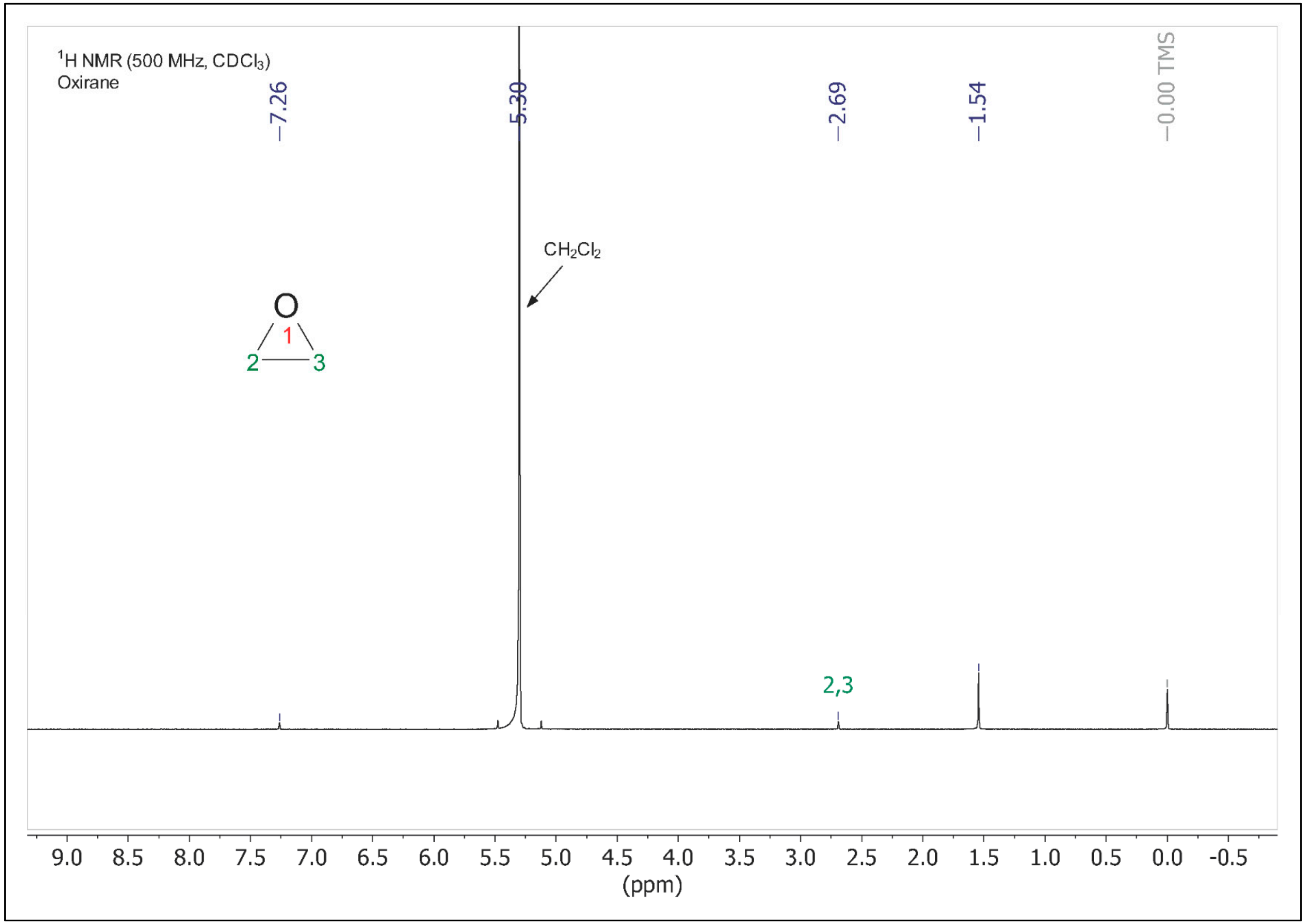

Note: Oxirane was obtained as a 2 mg/mL standard pre-dissolved in CH_2Cl_2.

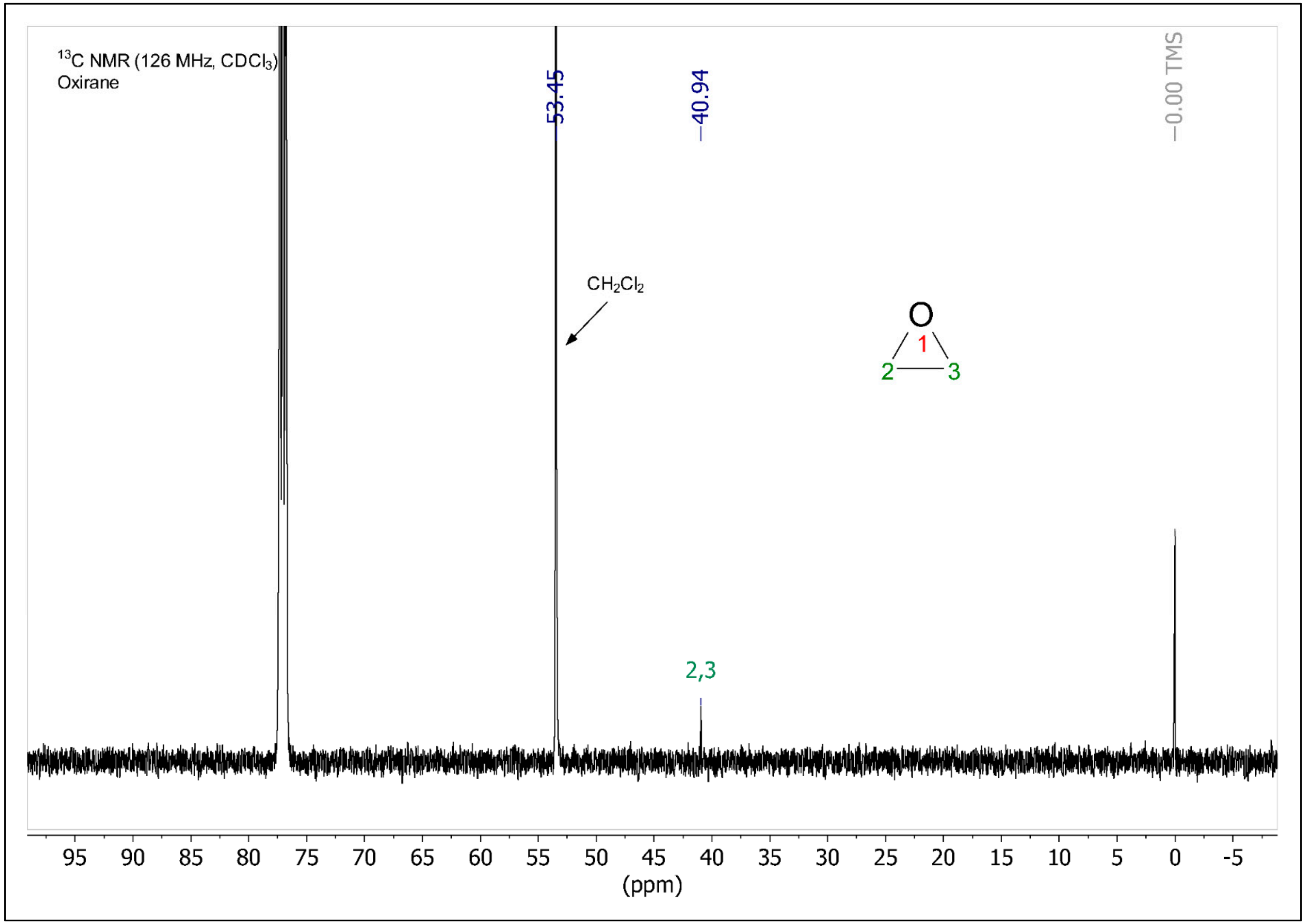

Note: Oxirane was obtained as a 2 mg/mL standard pre-dissolved in CH_2Cl_2.

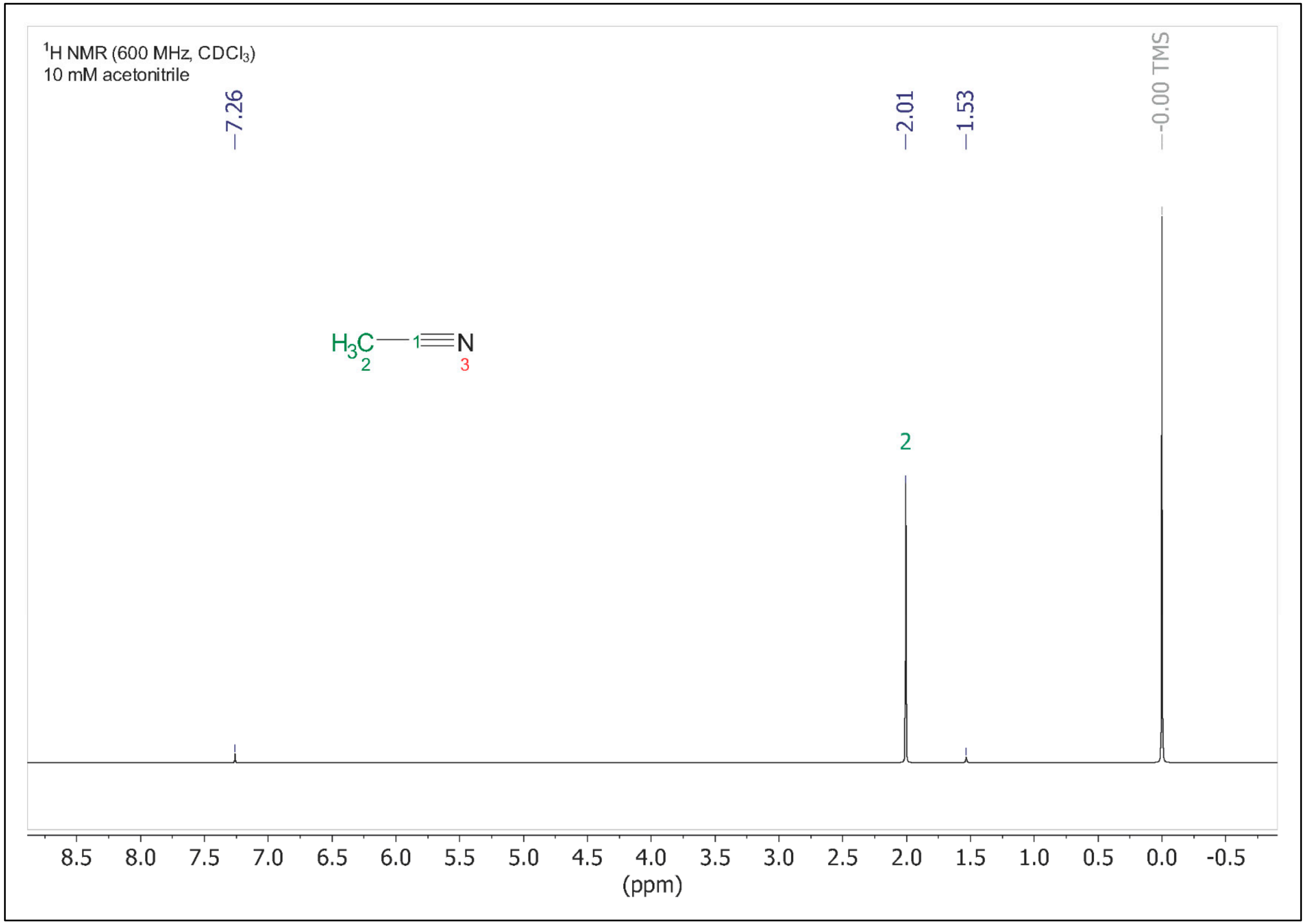

^{1}H NMR (600 MHz, CDCl$_3$)
10 mM acetonitrile
7.26
2.01
1.53
0.00 TMS
H$_3$C 2 — 1 ≡ N 3
2
8.5 8.0 7.5 7.0 6.5 6.0 5.5 5.0 4.5 4.0 3.5 3.0 2.5 2.0 1.5 1.0 0.5 0.0 -0.5
(ppm)

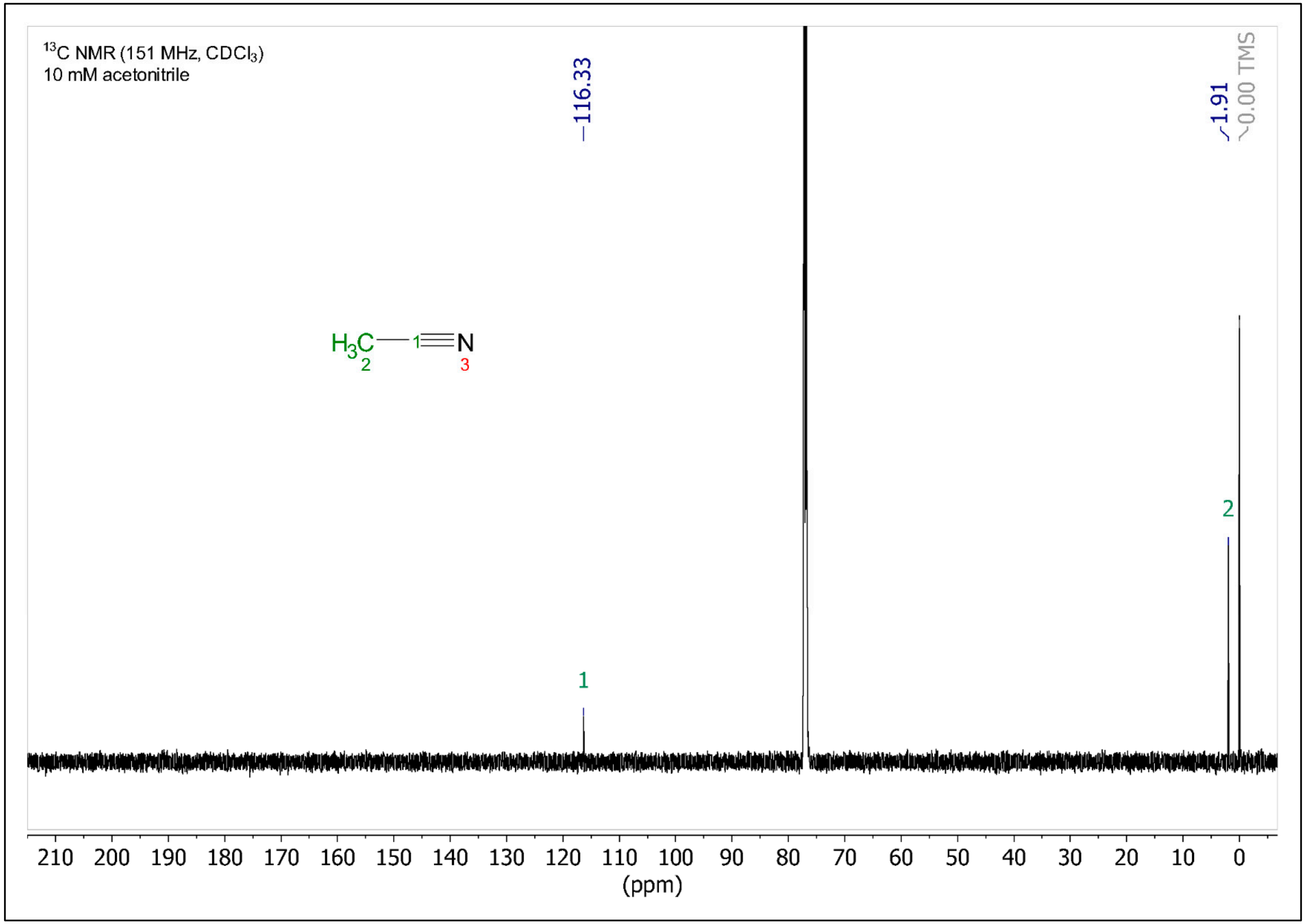

^{13}C NMR (151 MHz, CDCl$_3$)
10 mM acetonitrile
116.33
1.91
0.00 TMS
H3C
1
N
2
3
1
2
210 200 190 180 170 160 150 140 130 120 110 100 90 80 70 60 50 40 30 20 10 0
(ppm)

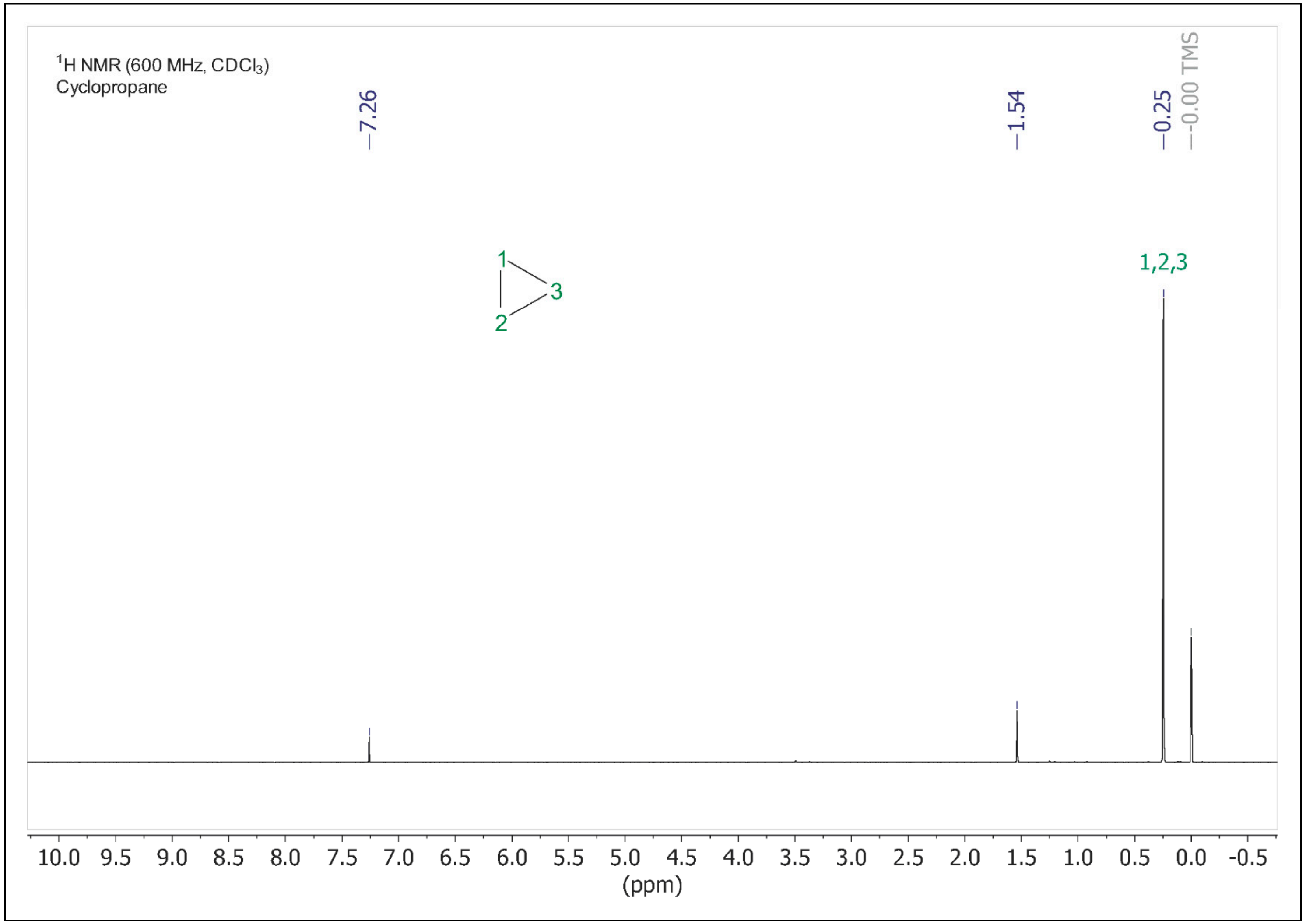

^{1}H NMR (600 MHz, $CDCl_3$)
Cyclopropane
7.26
1.54
0.25
0.00 TMS
1
2
3
1,2,3
10.0 9.5 9.0 8.5 8.0 7.5 7.0 6.5 6.0 5.5 5.0 4.5 4.0 3.5 3.0 2.5 2.0 1.5 1.0 0.5 0.0 -0.5
(ppm)

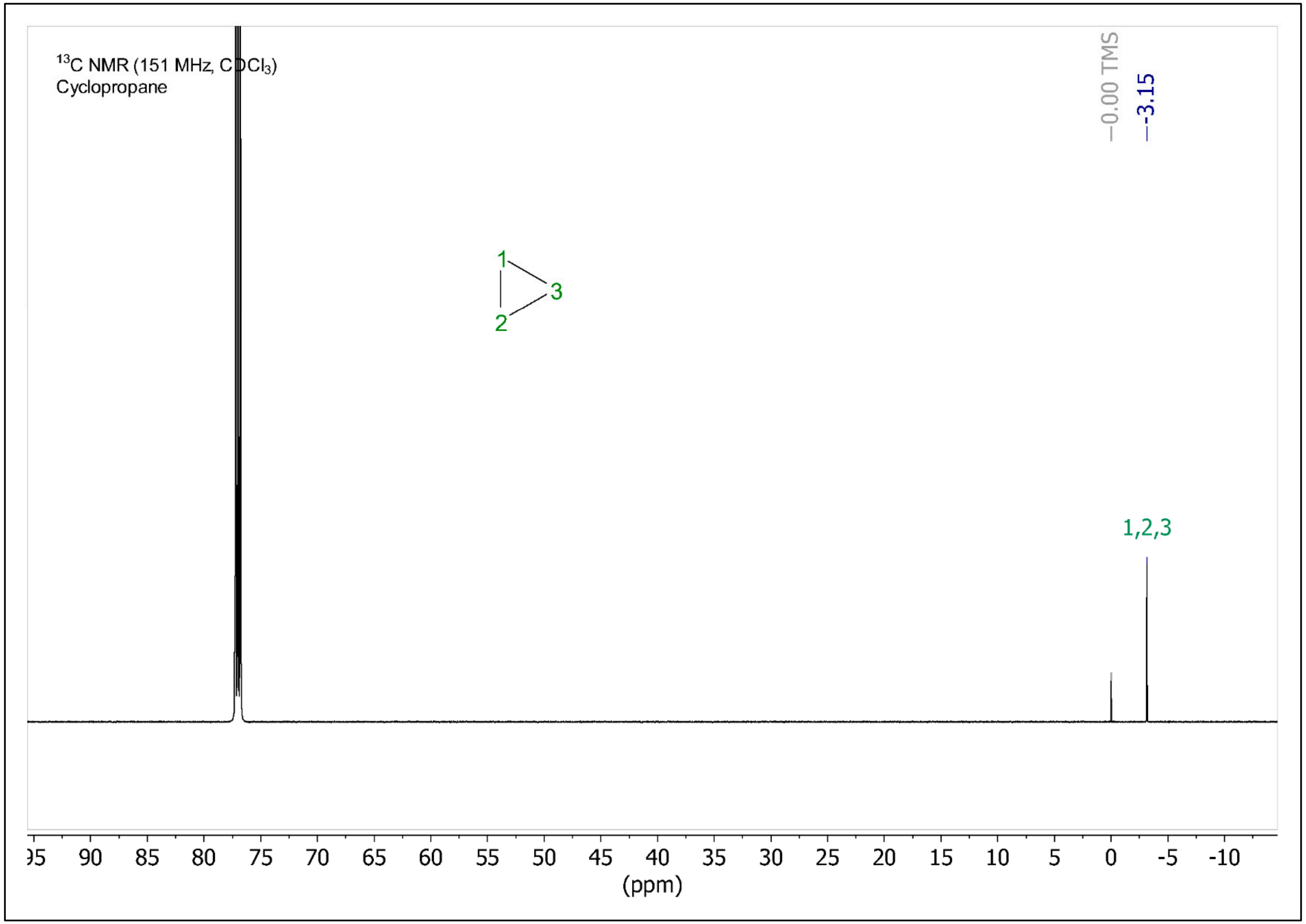

^{13}C NMR (151 MHz, CDCl$_3$)
Cyclopropane
1
2
3
—0.00 TMS
—-3.15
1,2,3
95 90 85 80 75 70 65 60 55 50 45 40 35 30 25 20 15 10 5 0 -5 -10
(ppm)

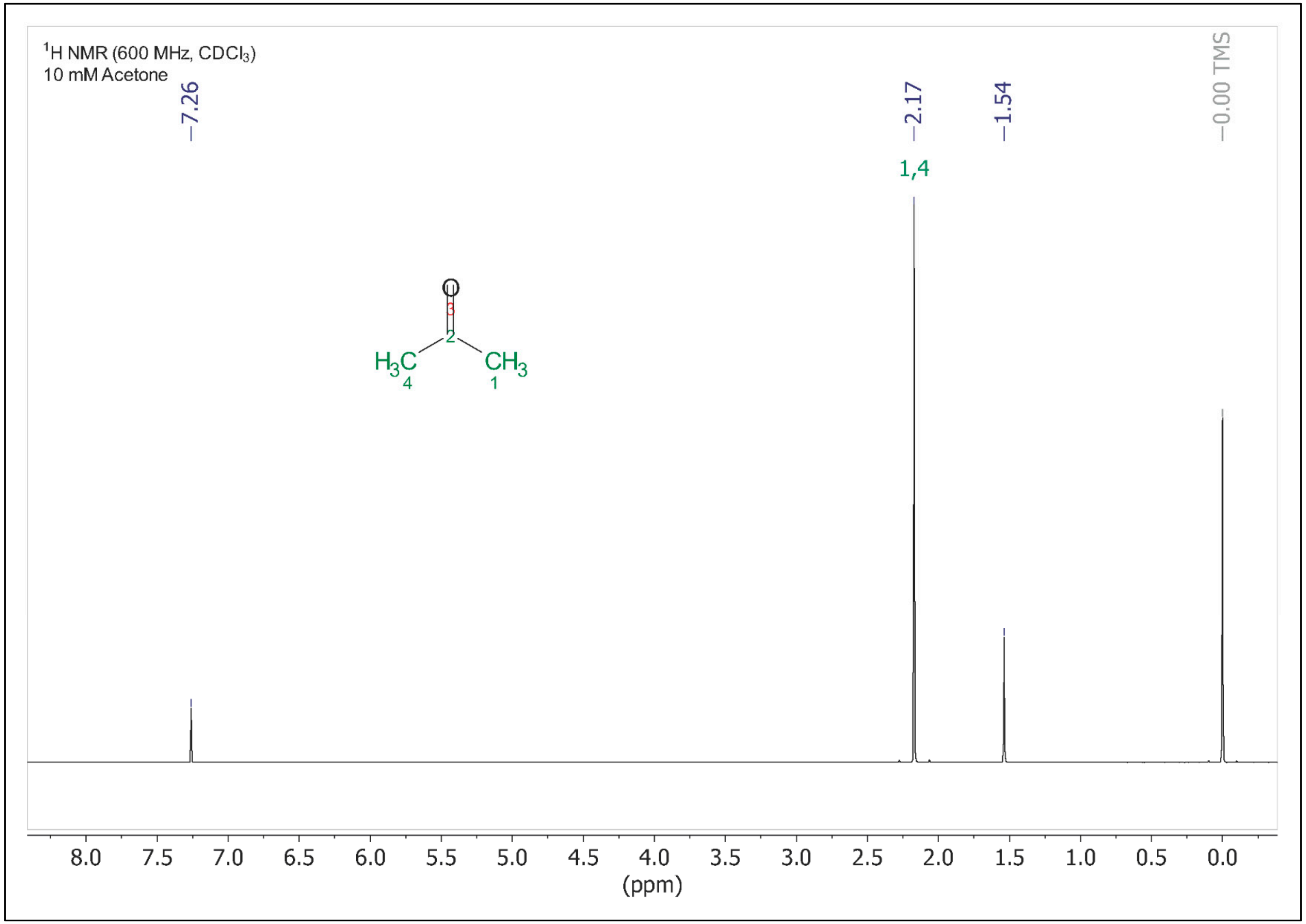

^1H NMR (600 MHz, $CDCl_3$)
10 mM Acetone
7.26
2.17
1.54
0.00 TMS
1,4
O
3
2
H_3C
4
CH_3
1
8.0 7.5 7.0 6.5 6.0 5.5 5.0 4.5 4.0 3.5 3.0 2.5 2.0 1.5 1.0 0.5 0.0
(ppm)

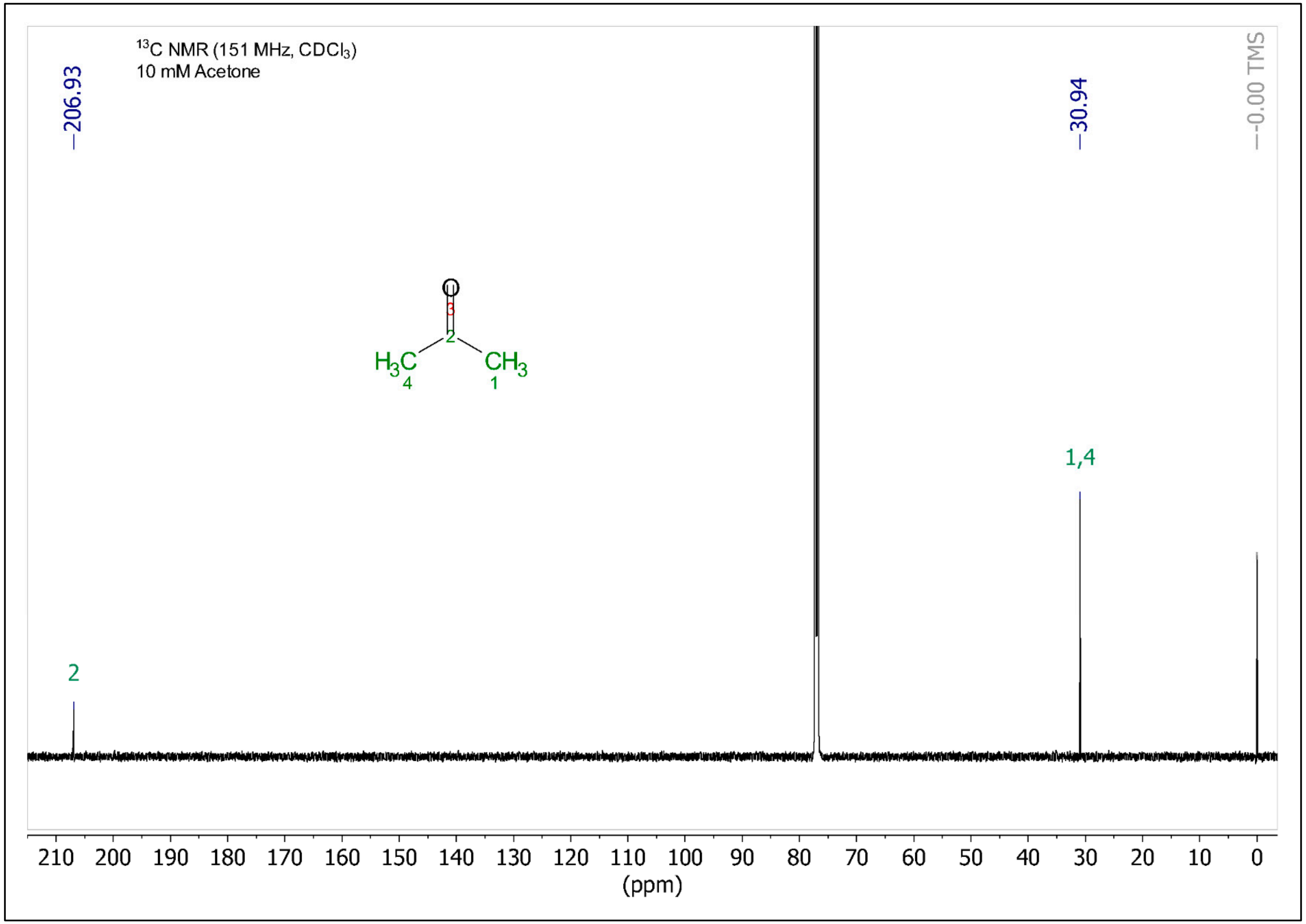

13C NMR (151 MHz, CDCl3)
10 mM Acetone
206.93
30.94
0.00 TMS
O
3
2
H3C
4
CH3
1
1,4
2
210 200 190 180 170 160 150 140 130 120 110 100 90 80 70 60 50 40 30 20 10 0
(ppm)

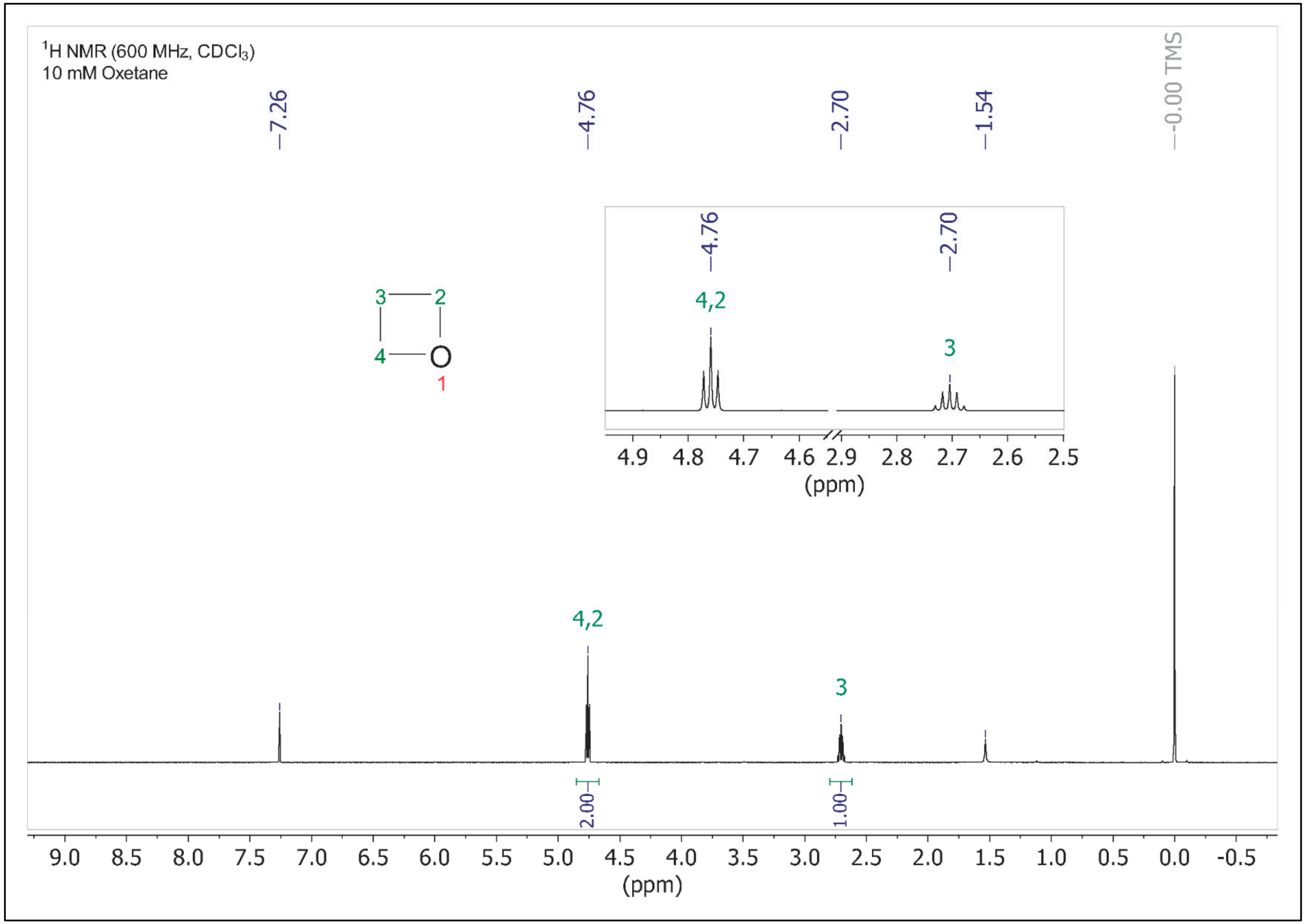

1H NMR (600 MHz, CDCl3)
10 mM Oxetane
7.26
4.76
2.70
1.54
-0.00 TMS
3
2
4
O
1
4,2
3
2.00
1.00
9.0 8.5 8.0 7.5 7.0 6.5 6.0 5.5 5.0 4.5 4.0 3.5 3.0 2.5 2.0 1.5 1.0 0.5 0.0 -0.5
(ppm)
4.9 4.8 4.7 4.6 2.9 2.8 2.7 2.6 2.5
(ppm)

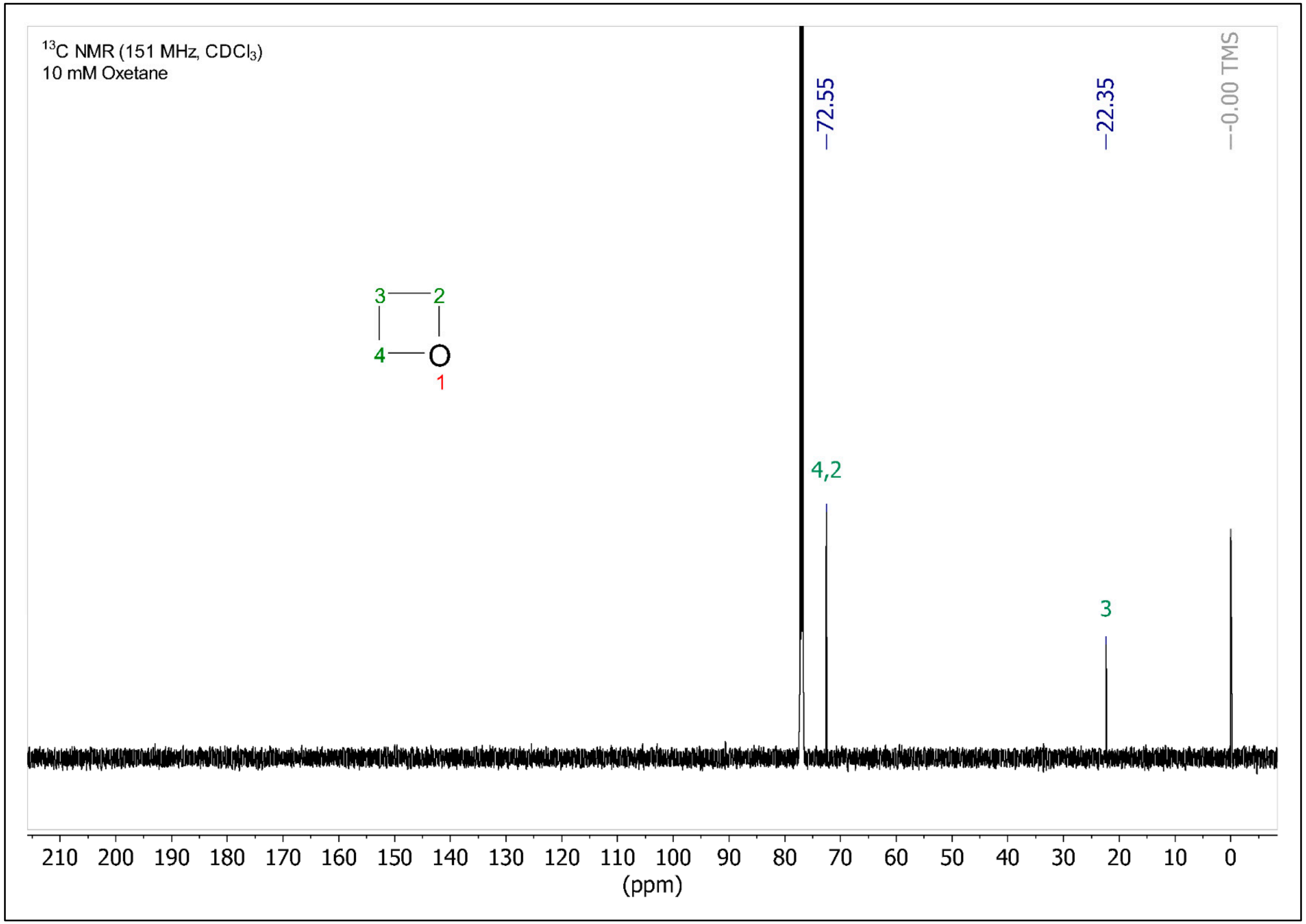

^{13}C NMR (151 MHz, CDCl$_3$)
10 mM Oxetane
72.55
22.35
-0.00 TMS
3
2
4
O
1
4,2
3
210 200 190 180 170 160 150 140 130 120 110 100 90 80 70 60 50 40 30 20 10 0
(ppm)

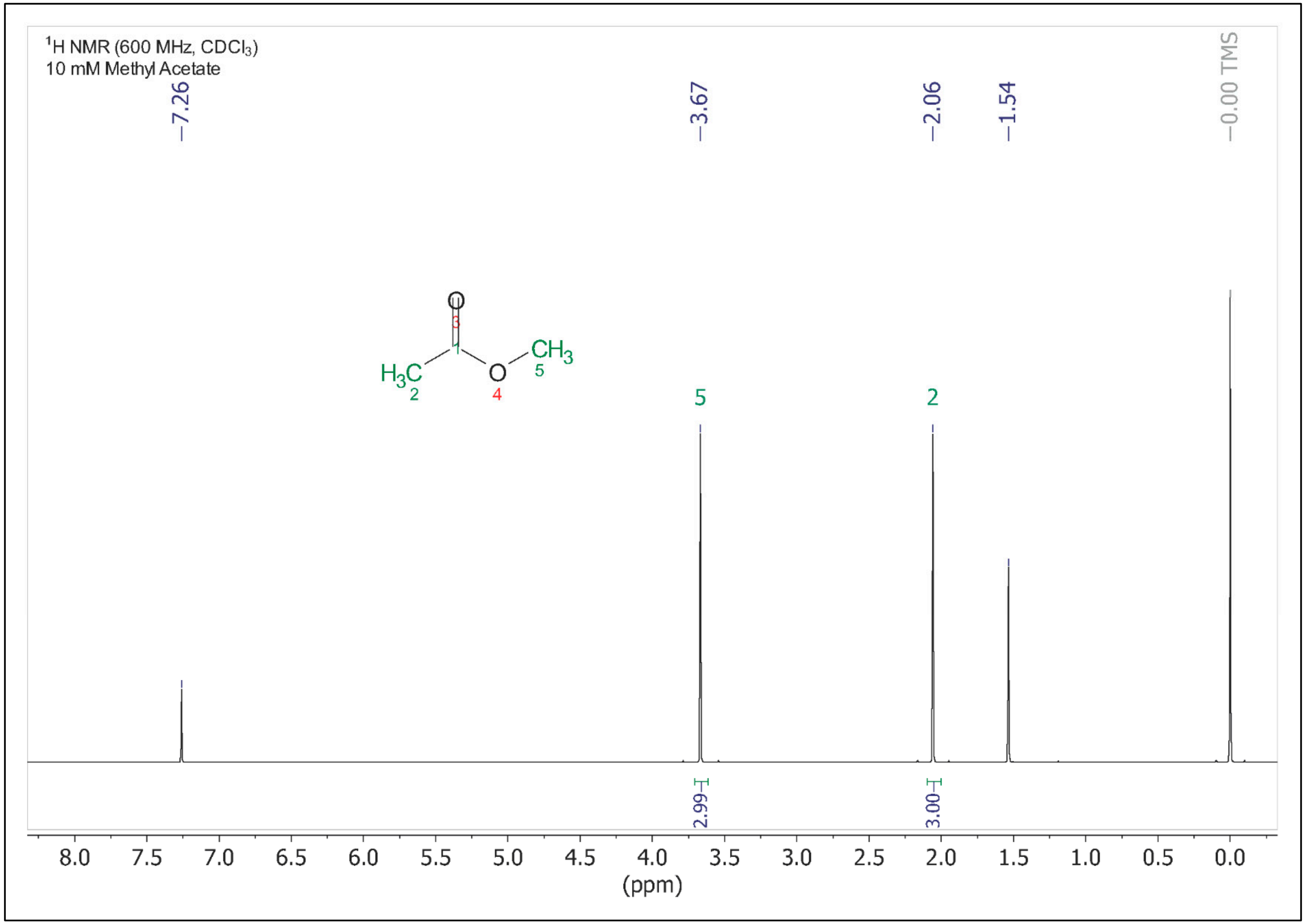

1H NMR (600 MHz, CDCl3)
10 mM Methyl Acetate
7.26
3.67
2.06
1.54
0.00 TMS
O
H3C
O
CH3
1
2
3
4
5
5
2
2.99
3.00
8.0 7.5 7.0 6.5 6.0 5.5 5.0 4.5 4.0 3.5 3.0 2.5 2.0 1.5 1.0 0.5 0.0
(ppm)

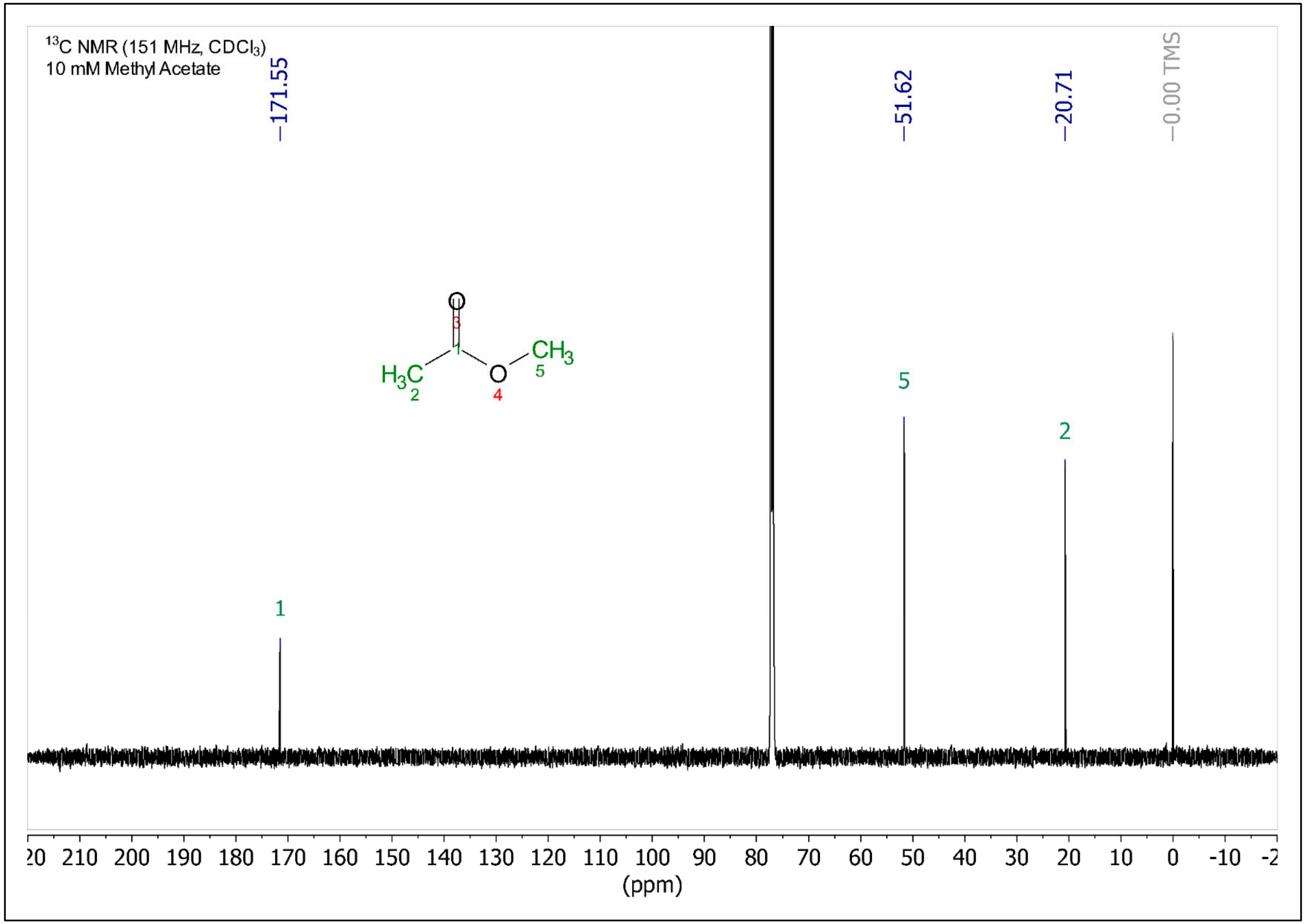

13C NMR (151 MHz, CDCl3)
10 mM Methyl Acetate
171.55
51.62
20.71
0.00 TMS
H3C
O
CH3
1
2
3
4
5
(ppm)

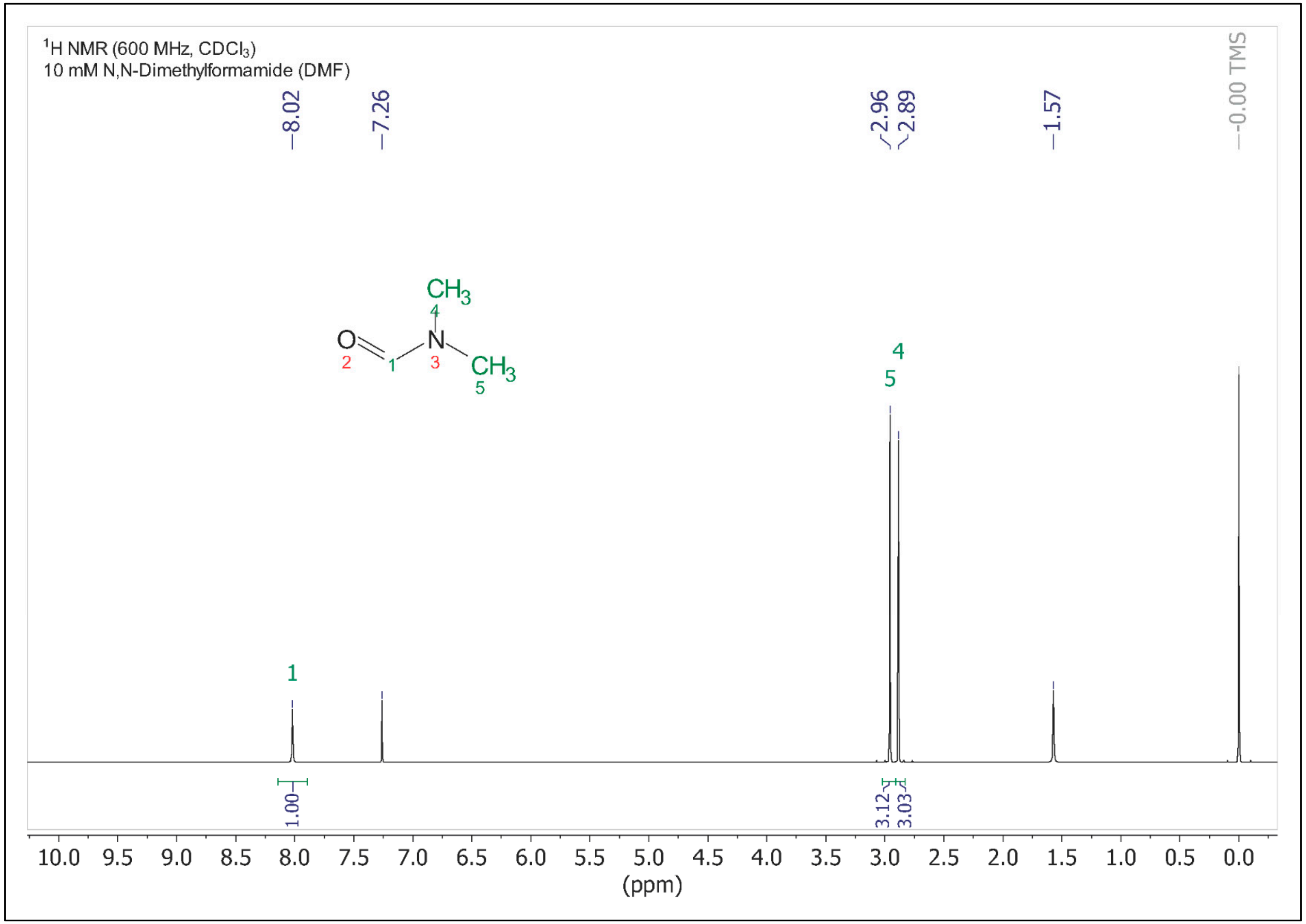

^{1}H NMR (600 MHz, CDCl$_3$)
10 mM N,N-Dimethylformamide (DMF)
8.02
7.26
2.96
2.89
1.57
-0.00 TMS
CH3
4
O
N
2
1
3
CH3
5
4
5
1
1.00
3.12
3.03
10.0 9.5 9.0 8.5 8.0 7.5 7.0 6.5 6.0 5.5 5.0 4.5 4.0 3.5 3.0 2.5 2.0 1.5 1.0 0.5 0.0
(ppm)

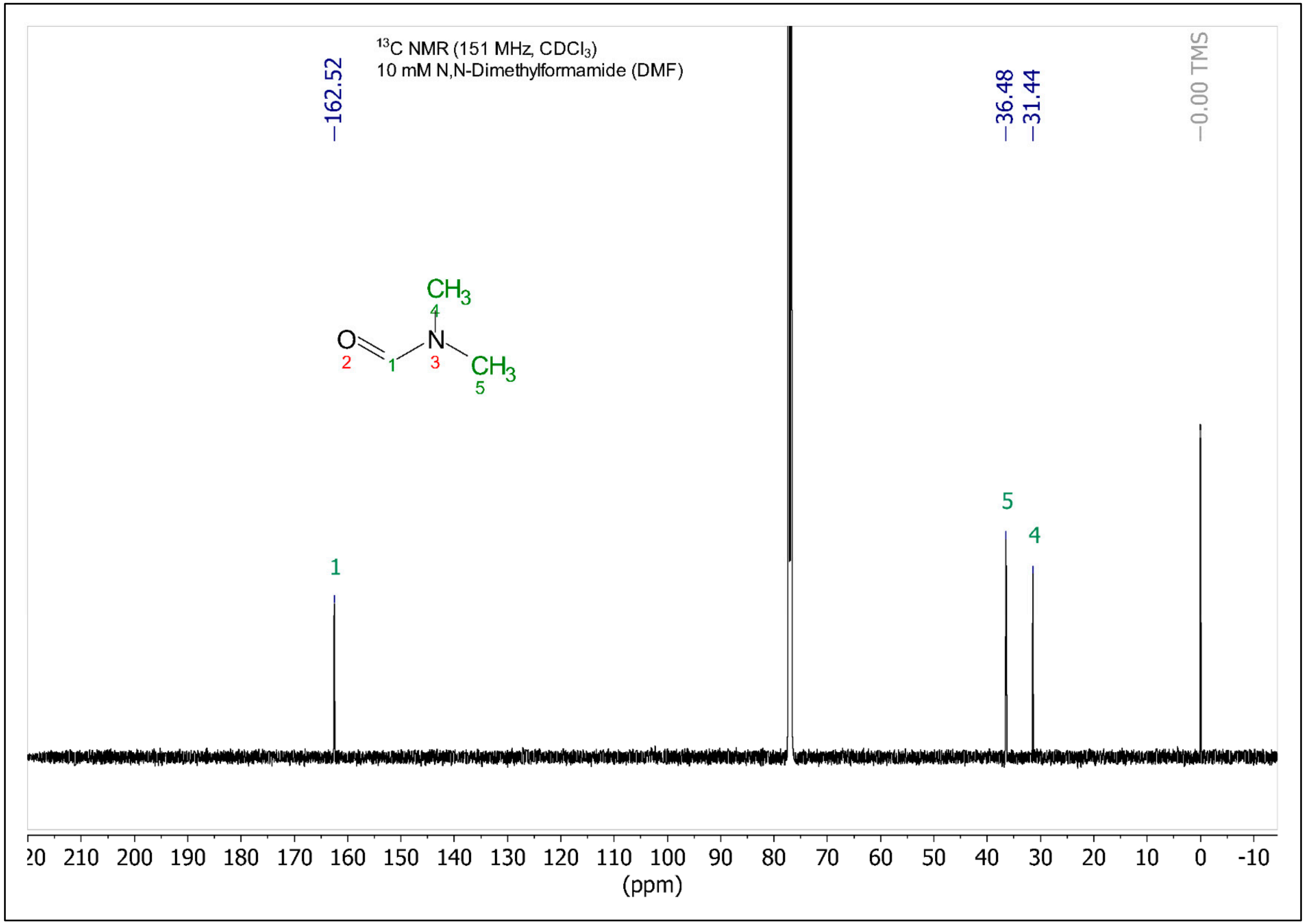

13C NMR (151 MHz, CDCl3)
10 mM N,N-Dimethylformamide (DMF)
162.52
36.48
31.44
-0.00 TMS
CH3
O
N
CH3
1
2
3
4
5
20 210 200 190 180 170 160 150 140 130 120 110 100 90 80 70 60 50 40 30 20 10 0 -10
(ppm)

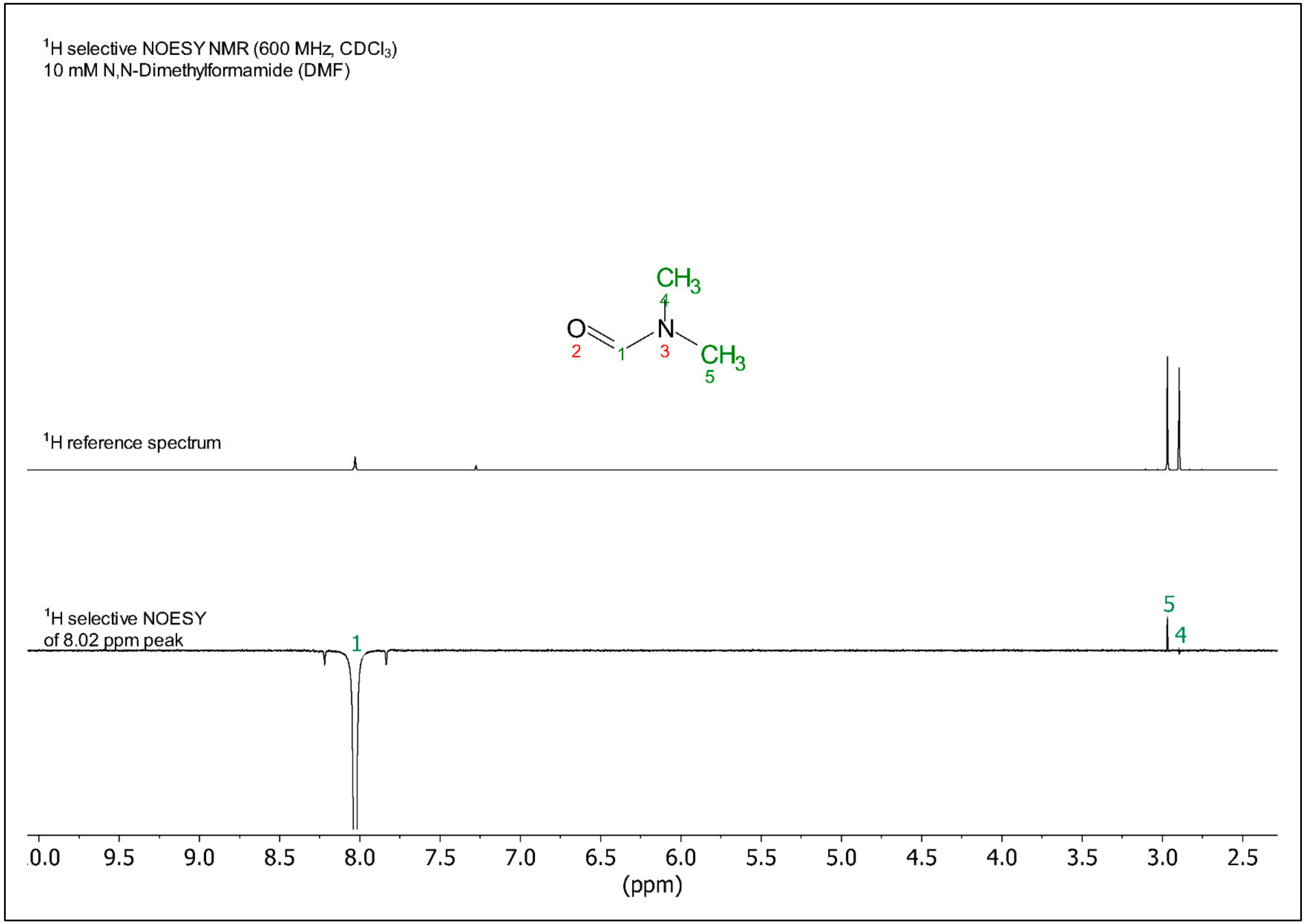

1H selective NOESY NMR (600 MHz, CDCl3)
10 mM N,N-Dimethylformamide (DMF)
CH3
4
O
N
2
1
3
CH3
5
1H reference spectrum
1H selective NOESY
of 8.02 ppm peak
1
5
4
10.0
9.5
9.0
8.5
8.0
7.5
7.0
6.5
6.0
5.5
5.0
4.5
4.0
3.5
3.0
2.5
(ppm)

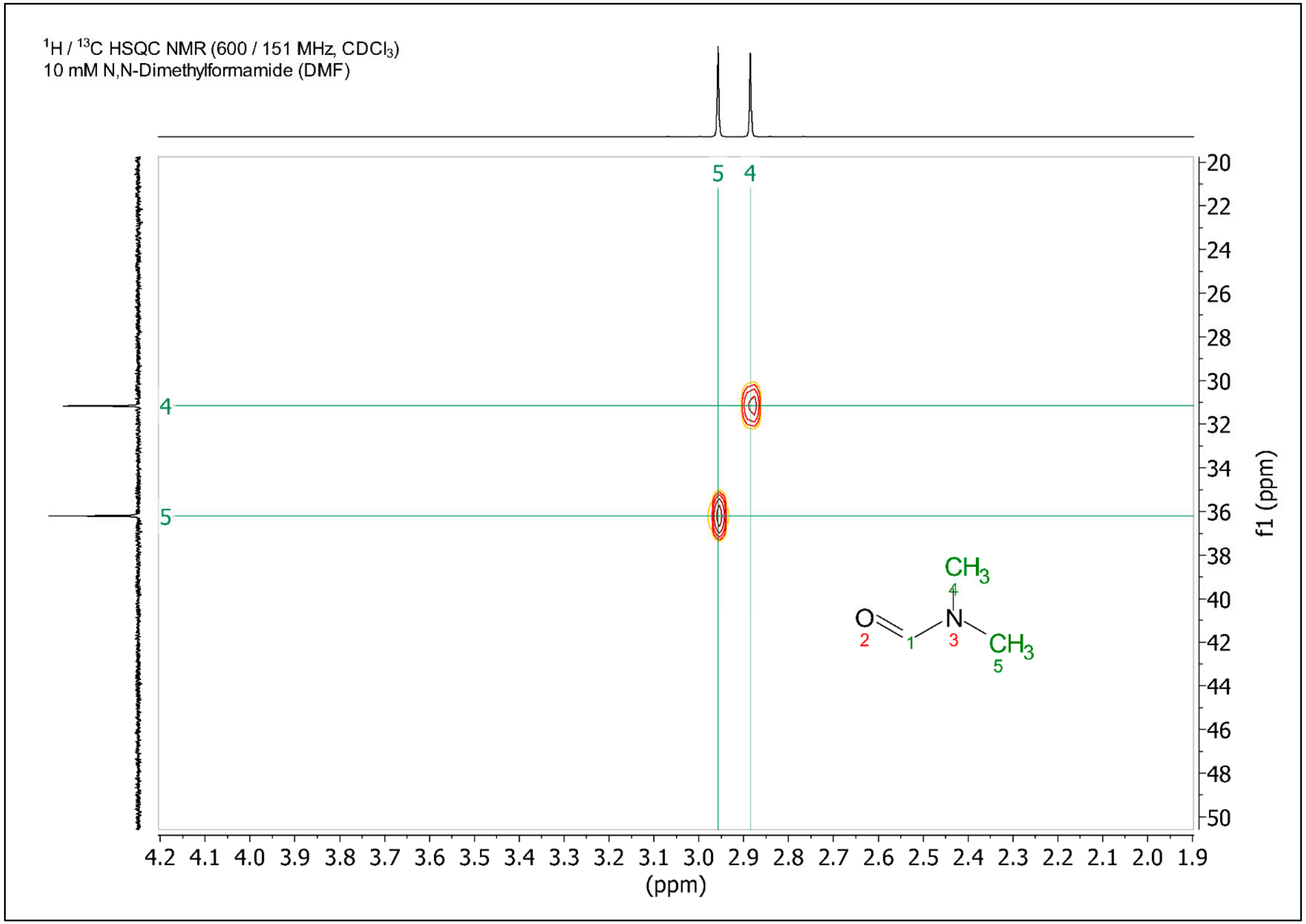

1H / 13C HSQC NMR (600 / 151 MHz, CDCl3)
10 mM N,N-Dimethylformamide (DMF)
f1 (ppm)
(ppm)

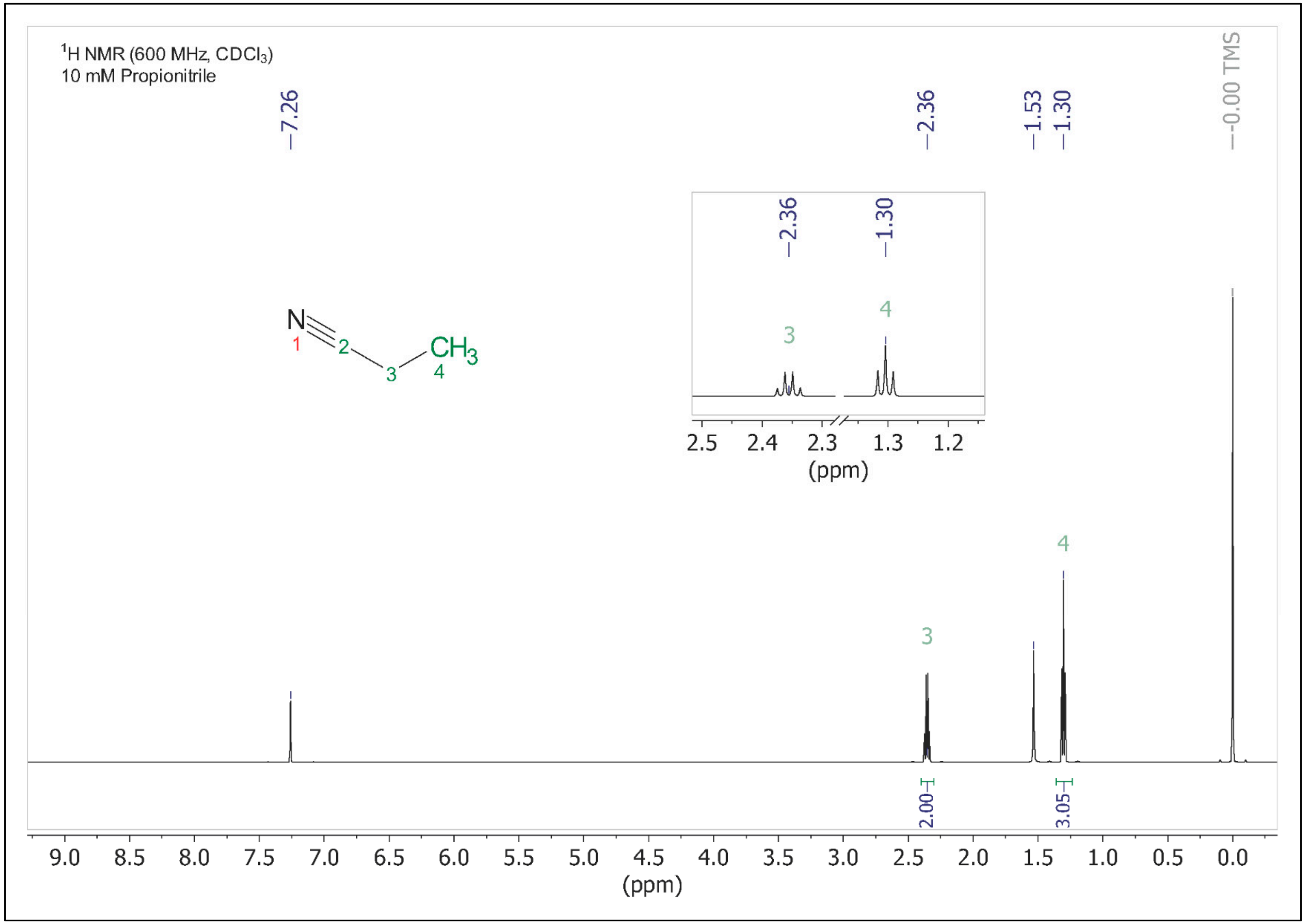

1H NMR (600 MHz, CDCl3)
10 mM Propionitrile
7.26
2.36
1.53
1.30
0.00 TMS
N
1
2
3
CH3
4
2.00
3.05
9.0 8.5 8.0 7.5 7.0 6.5 6.0 5.5 5.0 4.5 4.0 3.5 3.0 2.5 2.0 1.5 1.0 0.5 0.0
(ppm)
2.5 2.4 2.3 1.3 1.2

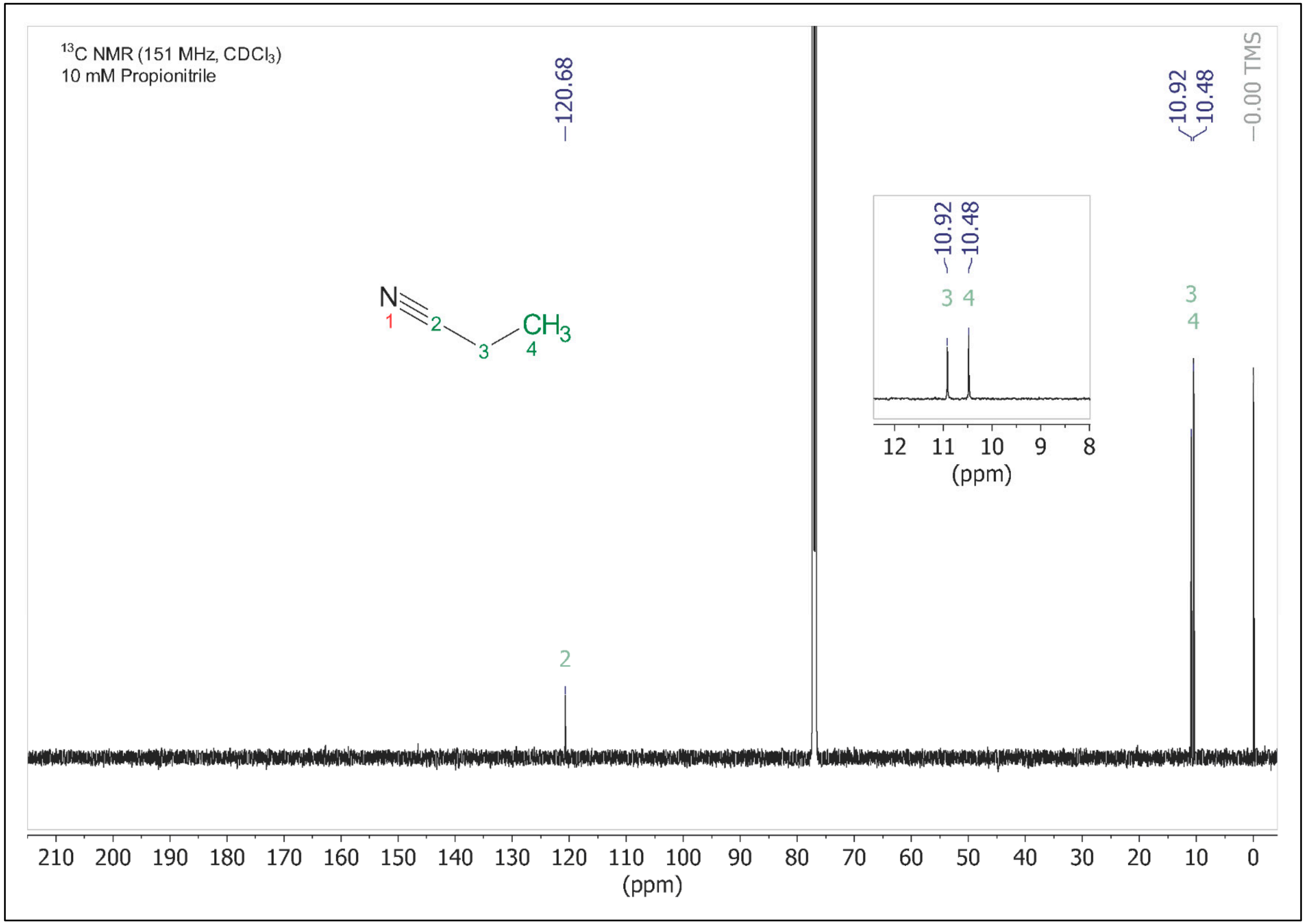

^{13}C NMR (151 MHz, CDCl$_3$)
10 mM Propionitrile
120.68
10.92
10.48
0.00 TMS
N
1
2
3
CH3
4
3 4
12 11 10 9 8
(ppm)
210 200 190 180 170 160 150 140 130 120 110 100 90 80 70 60 50 40 30 20 10 0
(ppm)

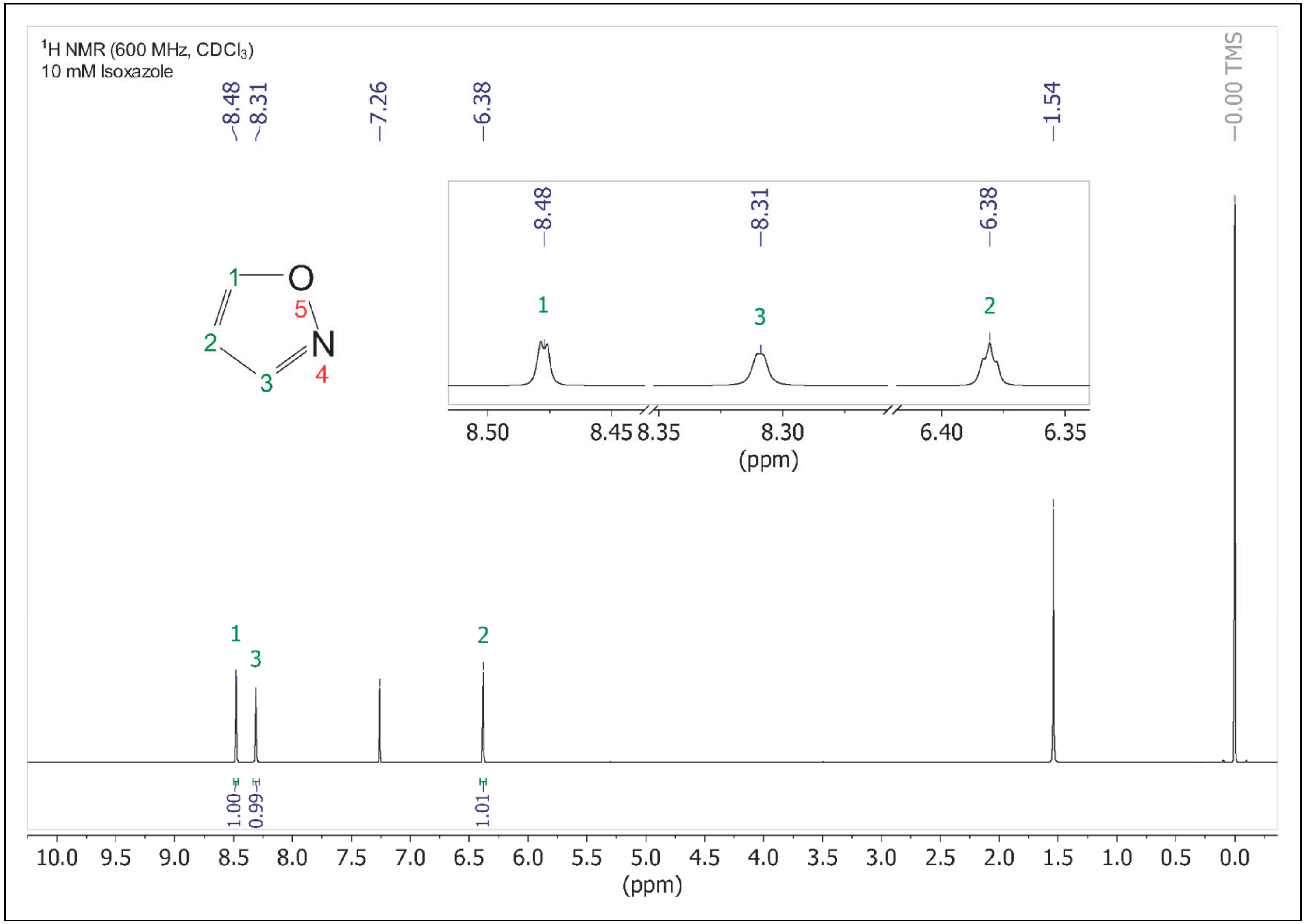

^{1}H NMR (600 MHz, CDCl$_3$)
10 mM Isoxazole
8.48
8.31
7.26
6.38
1.54
0.00 TMS
1
2
3
4
5
O
N
8.50
8.45
8.35
8.30
6.40
6.35
(ppm)
1.00
0.99
1.01
10.0 9.5 9.0 8.5 8.0 7.5 7.0 6.5 6.0 5.5 5.0 4.5 4.0 3.5 3.0 2.5 2.0 1.5 1.0 0.5 0.0

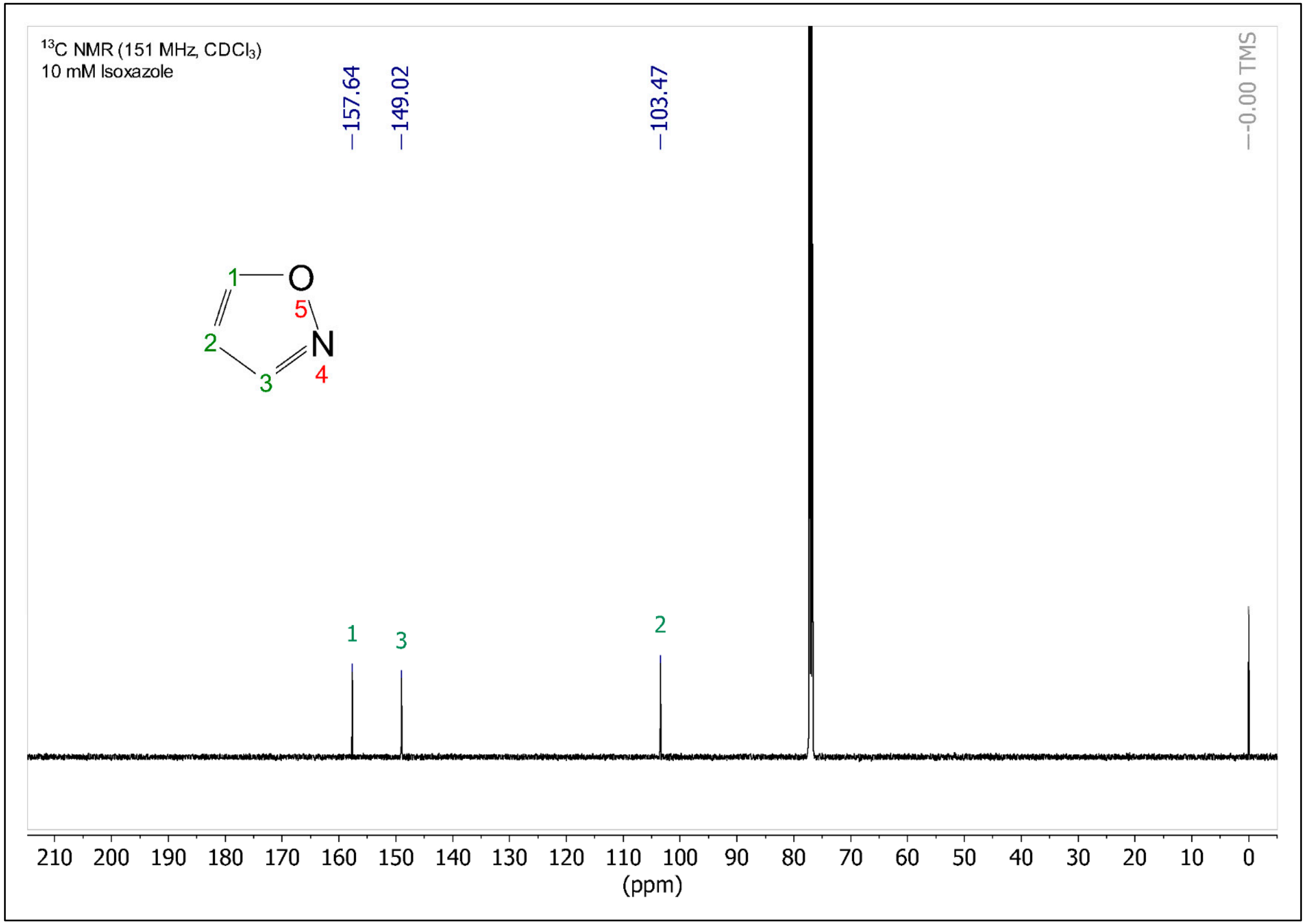

^{13}C NMR (151 MHz, CDCl$_3$)
10 mM Isoxazole
157.64
149.02
103.47
-0.00 TMS
1
O
5
2
N
3
4
1
3
2
210 200 190 180 170 160 150 140 130 120 110 100 90 80 70 60 50 40 30 20 10 0
(ppm)

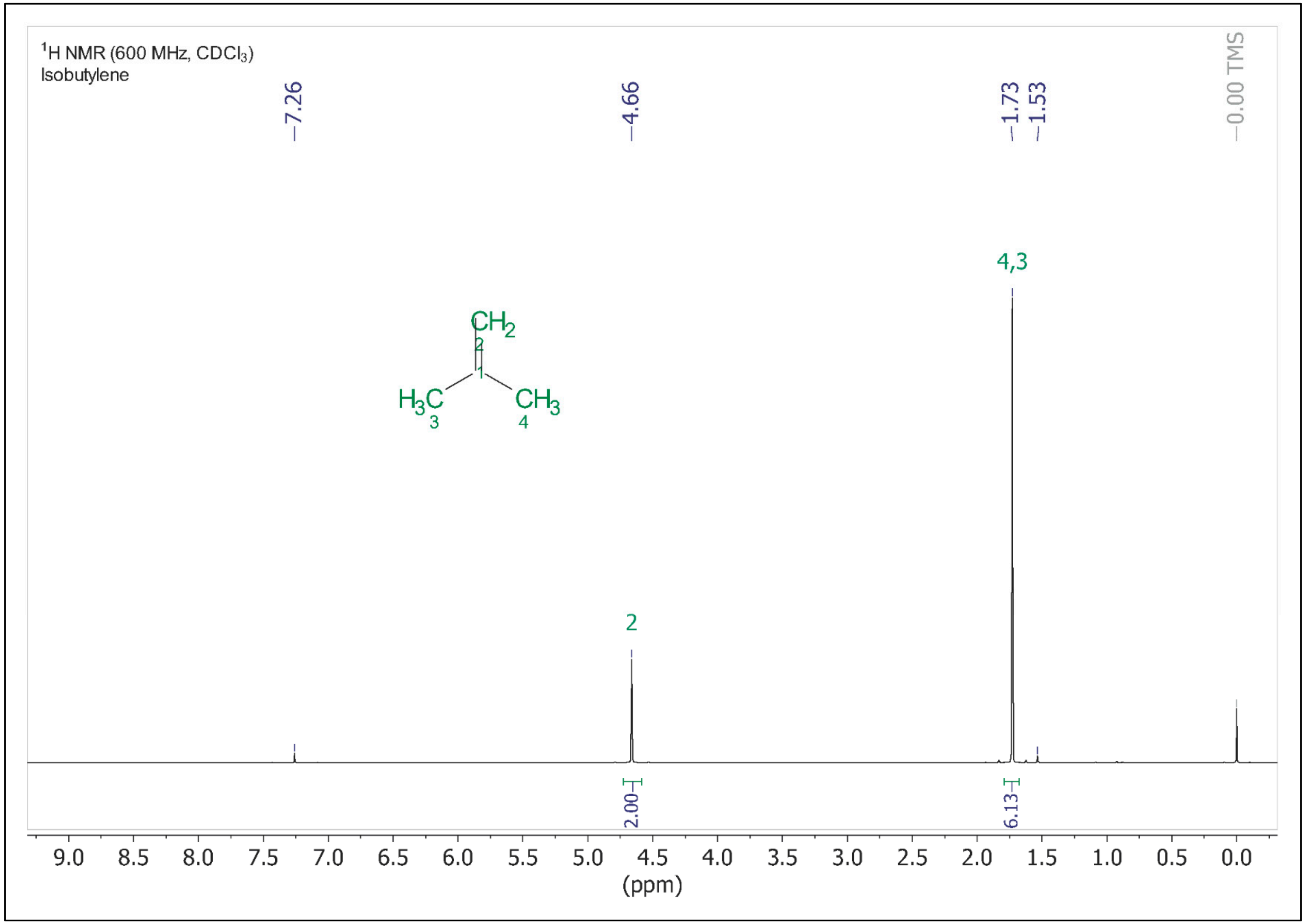

^{1}H NMR (600 MHz, CDCl$_3$)
Isobutylene
7.26
4.66
1.73
1.53
0.00 TMS
4,3
2
CH2
H3C
CH3
2.00
6.13
9.0 8.5 8.0 7.5 7.0 6.5 6.0 5.5 5.0 4.5 4.0 3.5 3.0 2.5 2.0 1.5 1.0 0.5 0.0
(ppm)

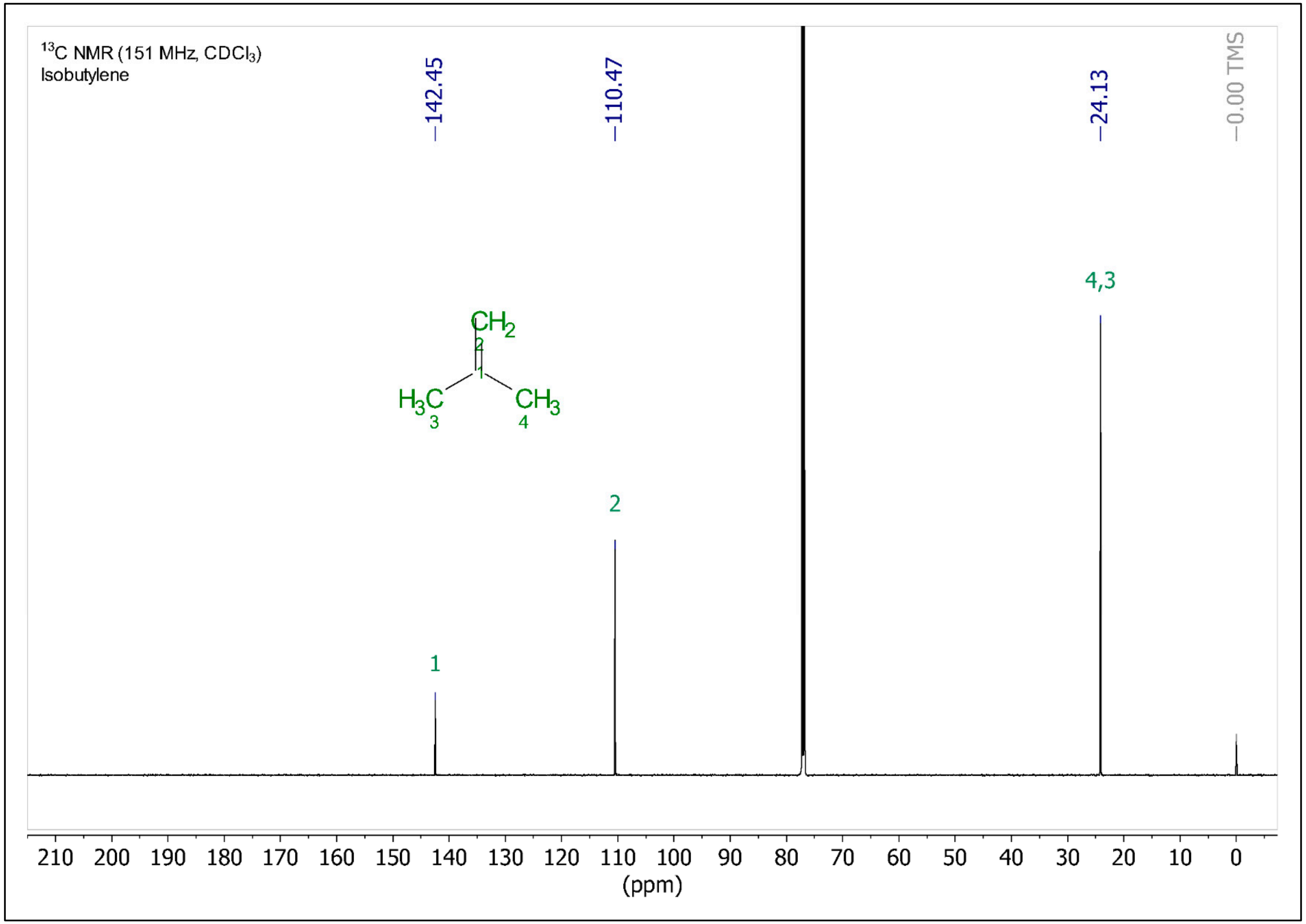

13C NMR (151 MHz, CDCl3)
Isobutylene
142.45
110.47
24.13
-0.00 TMS
1
2
4,3
CH2
H3C
CH3
210 200 190 180 170 160 150 140 130 120 110 100 90 80 70 60 50 40 30 20 10 0
(ppm)

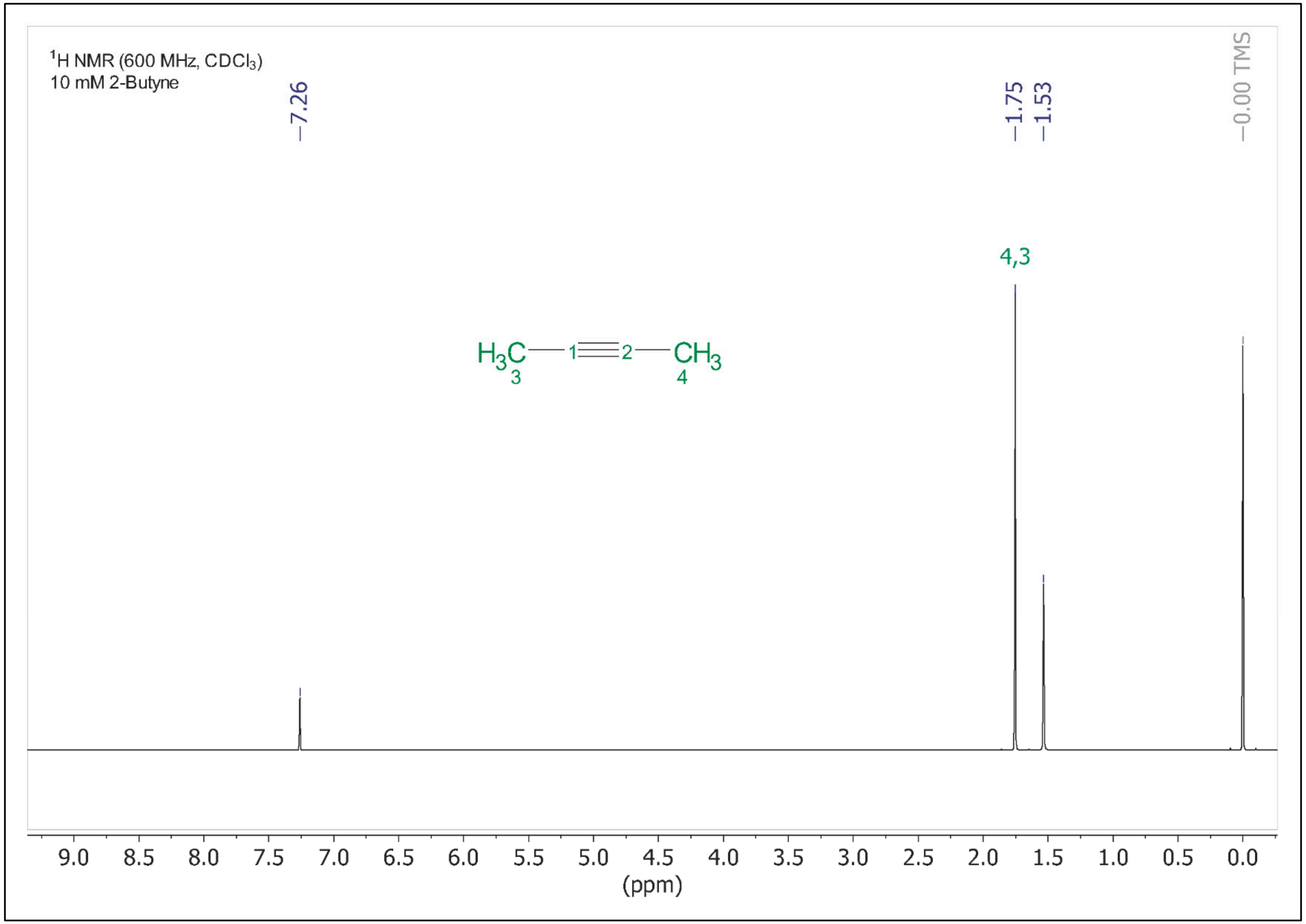

¹H NMR (600 MHz, CDCl3)
10 mM 2-Butyne
7.26
1.75
1.53
0.00 TMS
4,3
H3C
1
2
CH3
3
4
9.0 8.5 8.0 7.5 7.0 6.5 6.0 5.5 5.0 4.5 4.0 3.5 3.0 2.5 2.0 1.5 1.0 0.5 0.0
(ppm)

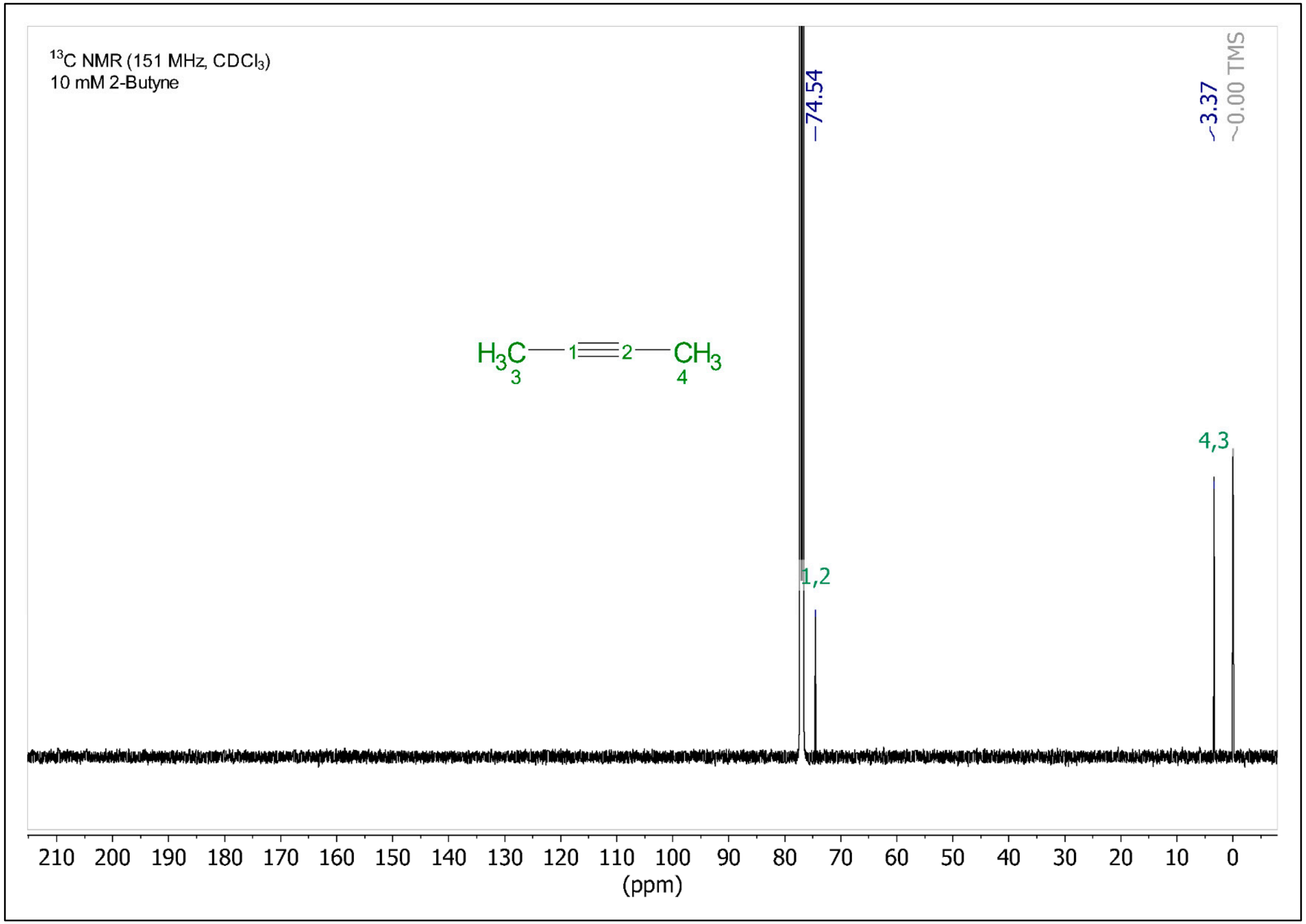

^{13}C NMR (151 MHz, $CDCl_3$)
10 mM 2-Butyne
74.54
3.37
0.00 TMS
H_3C 1 2 CH_3
3 4
1,2
4,3
210 200 190 180 170 160 150 140 130 120 110 100 90 80 70 60 50 40 30 20 10 0
(ppm)

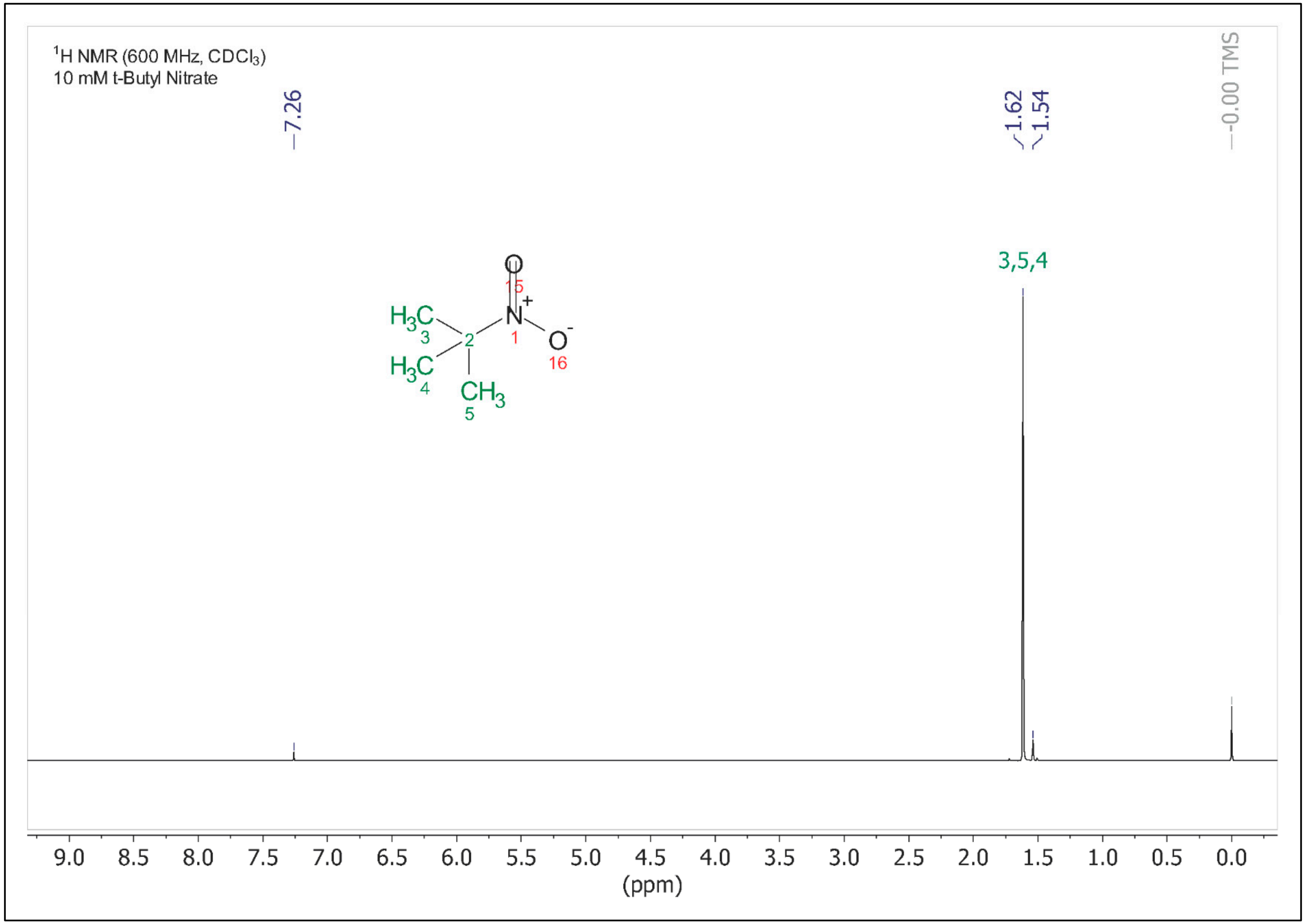

1H NMR (600 MHz, CDCl3)
10 mM t-Butyl Nitrate
7.26
1.62
1.54
0.00 TMS
3,5,4
9.0
8.5
8.0
7.5
7.0
6.5
6.0
5.5
5.0
4.5
4.0
3.5
3.0
2.5
2.0
1.5
1.0
0.5
0.0
(ppm)

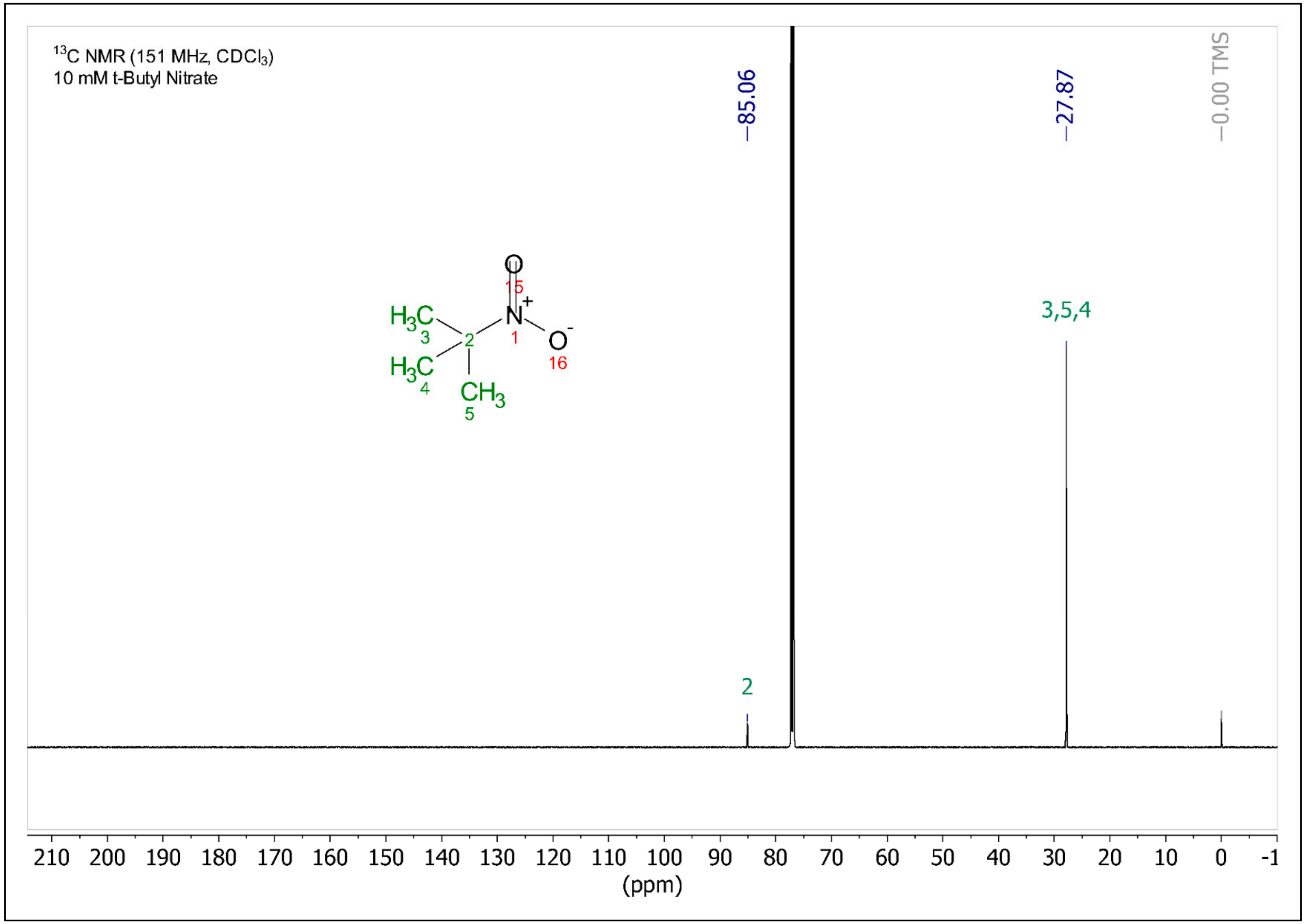

13C NMR (151 MHz, CDCl3)
10 mM t-Butyl Nitrate
85.06
27.87
0.00 TMS
2
3,5,4
210 200 190 180 170 160 150 140 130 120 110 100 90 80 70 60 50 40 30 20 10 0 -1
(ppm)

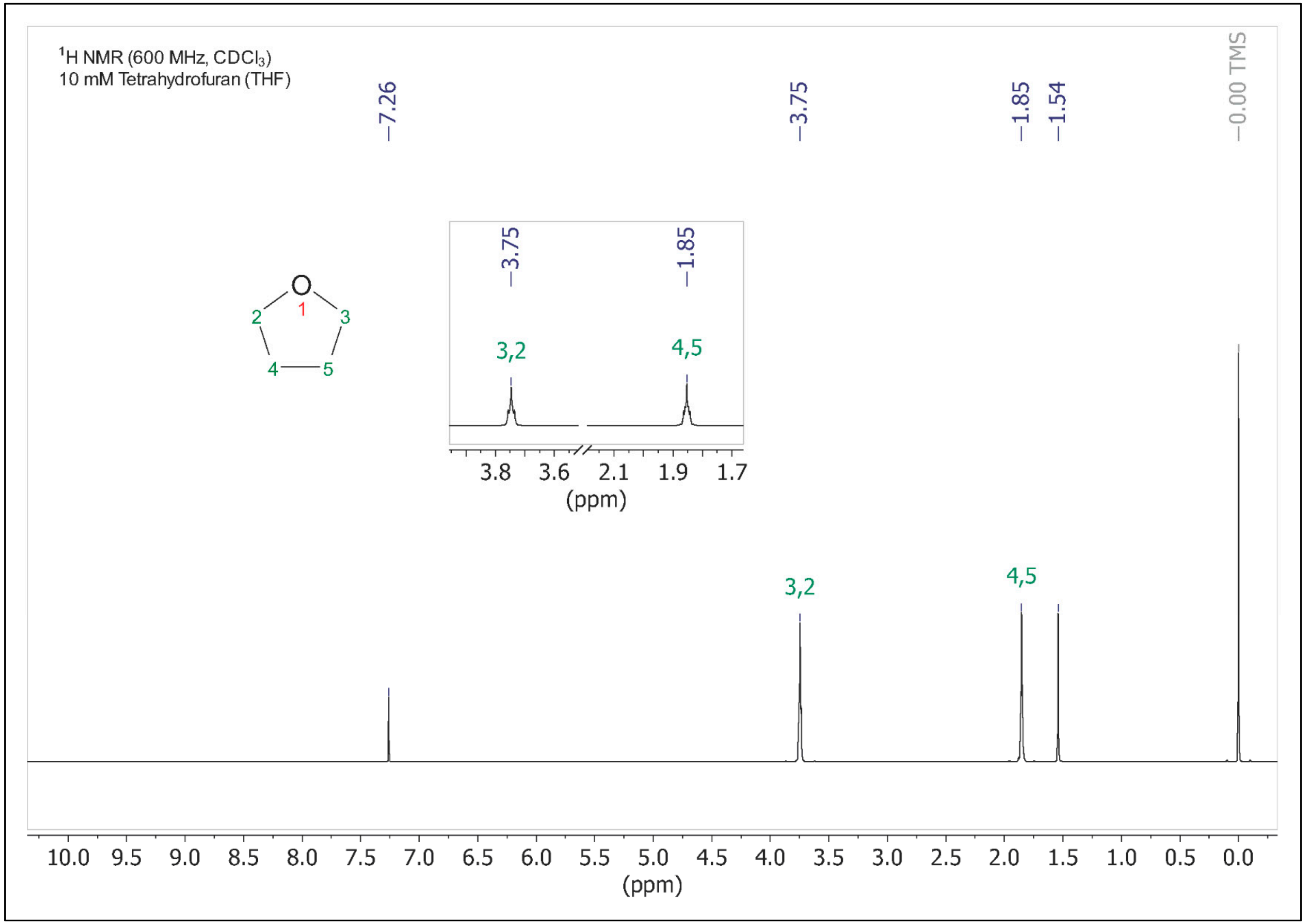

^{1}H NMR (600 MHz, CDCl$_3$)
10 mM Tetrahydrofuran (THF)
7.26
3.75
1.85
1.54
0.00 TMS
O
1
2
3
4
5
3,2
4,5
3.8 3.6 2.1 1.9 1.7
(ppm)
10.0 9.5 9.0 8.5 8.0 7.5 7.0 6.5 6.0 5.5 5.0 4.5 4.0 3.5 3.0 2.5 2.0 1.5 1.0 0.5 0.0
(ppm)

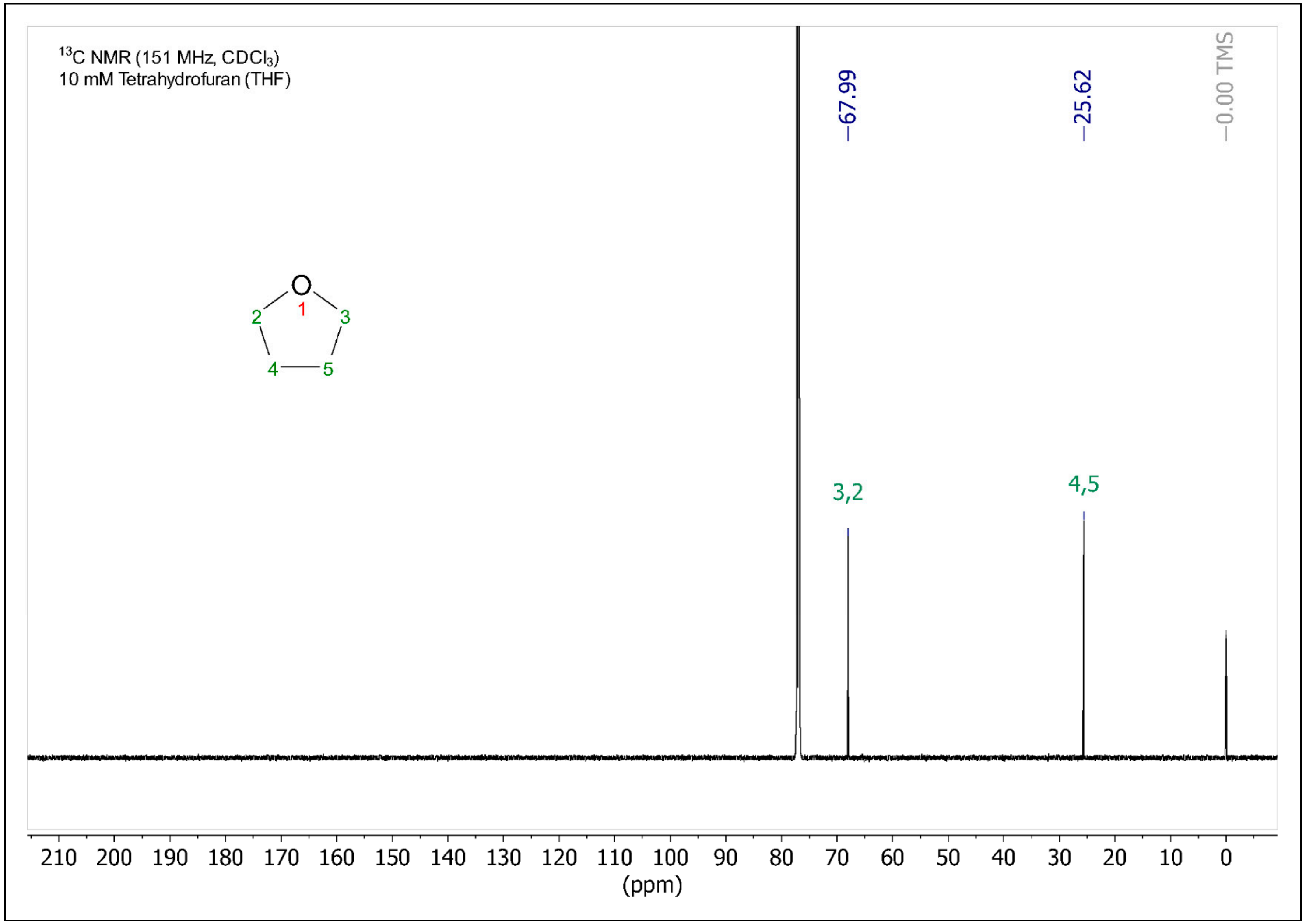

13C NMR (151 MHz, CDCl3)
10 mM Tetrahydrofuran (THF)
O
1
2
3
4
5
—67.99
—25.62
—0.00 TMS
3,2
4,5
210 200 190 180 170 160 150 140 130 120 110 100 90 80 70 60 50 40 30 20 10 0
(ppm)

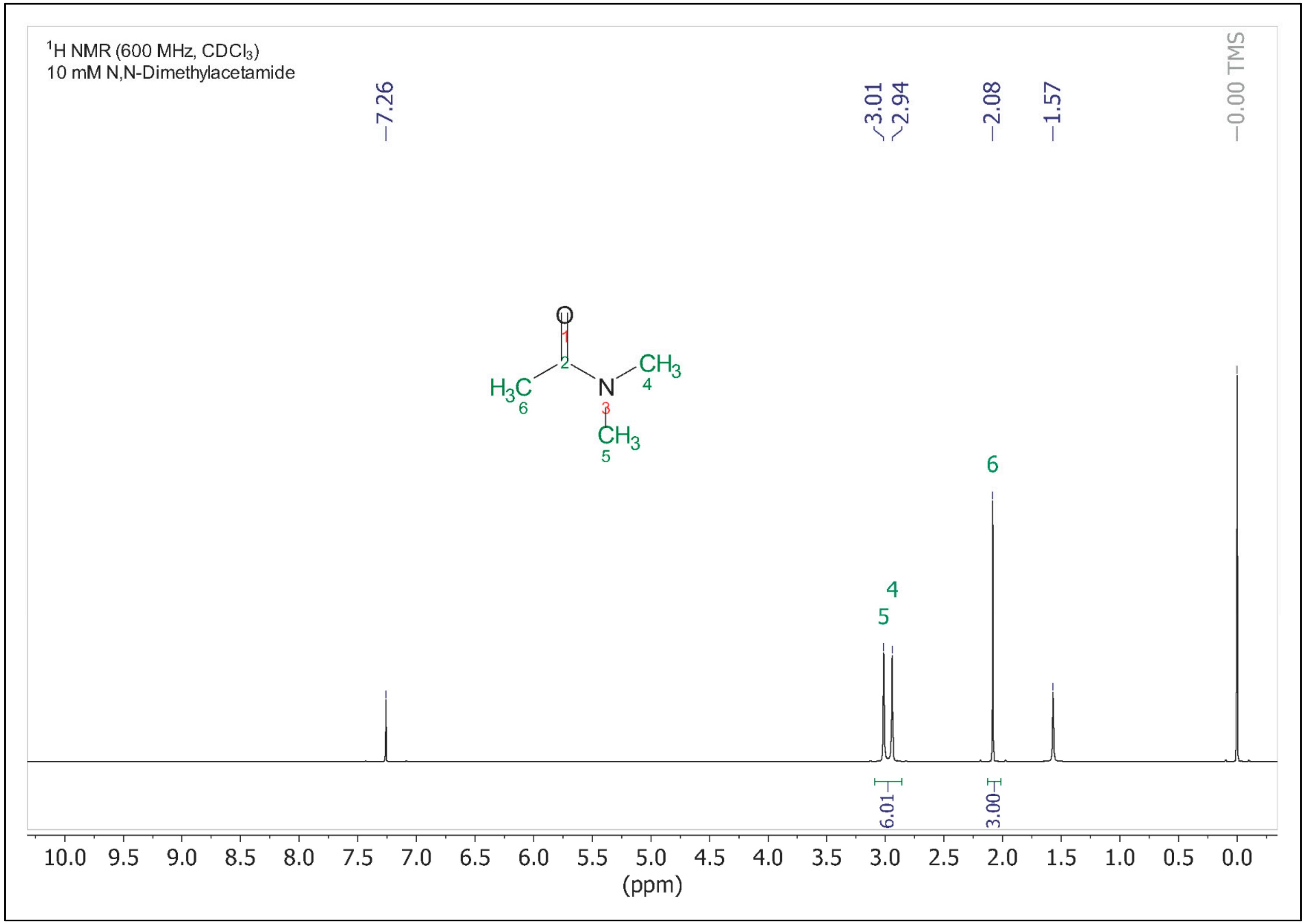

1H NMR (600 MHz, CDCl3)
10 mM N,N-Dimethylacetamide
7.26
3.01
2.94
2.08
1.57
0.00 TMS
O
H3C
N
CH3
CH3
1
2
3
4
5
6
6.01
3.00
10.0 9.5 9.0 8.5 8.0 7.5 7.0 6.5 6.0 5.5 5.0 4.5 4.0 3.5 3.0 2.5 2.0 1.5 1.0 0.5 0.0
(ppm)

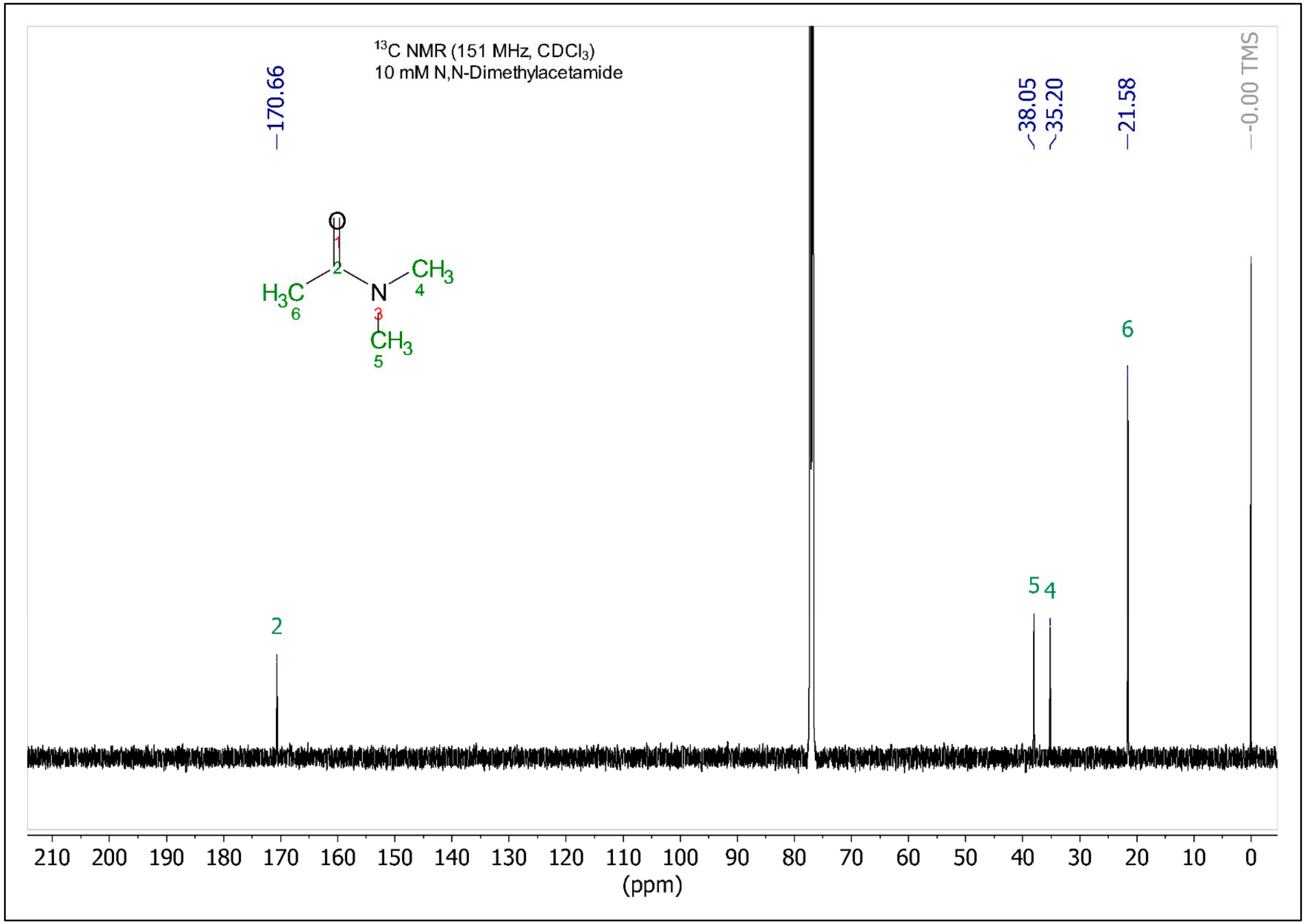

13C NMR (151 MHz, CDCl3)
10 mM N,N-Dimethylacetamide
170.66
38.05
35.20
21.58
-0.00 TMS
H3C
CH3
CH3
N
O
2
5
4
6
(ppm)

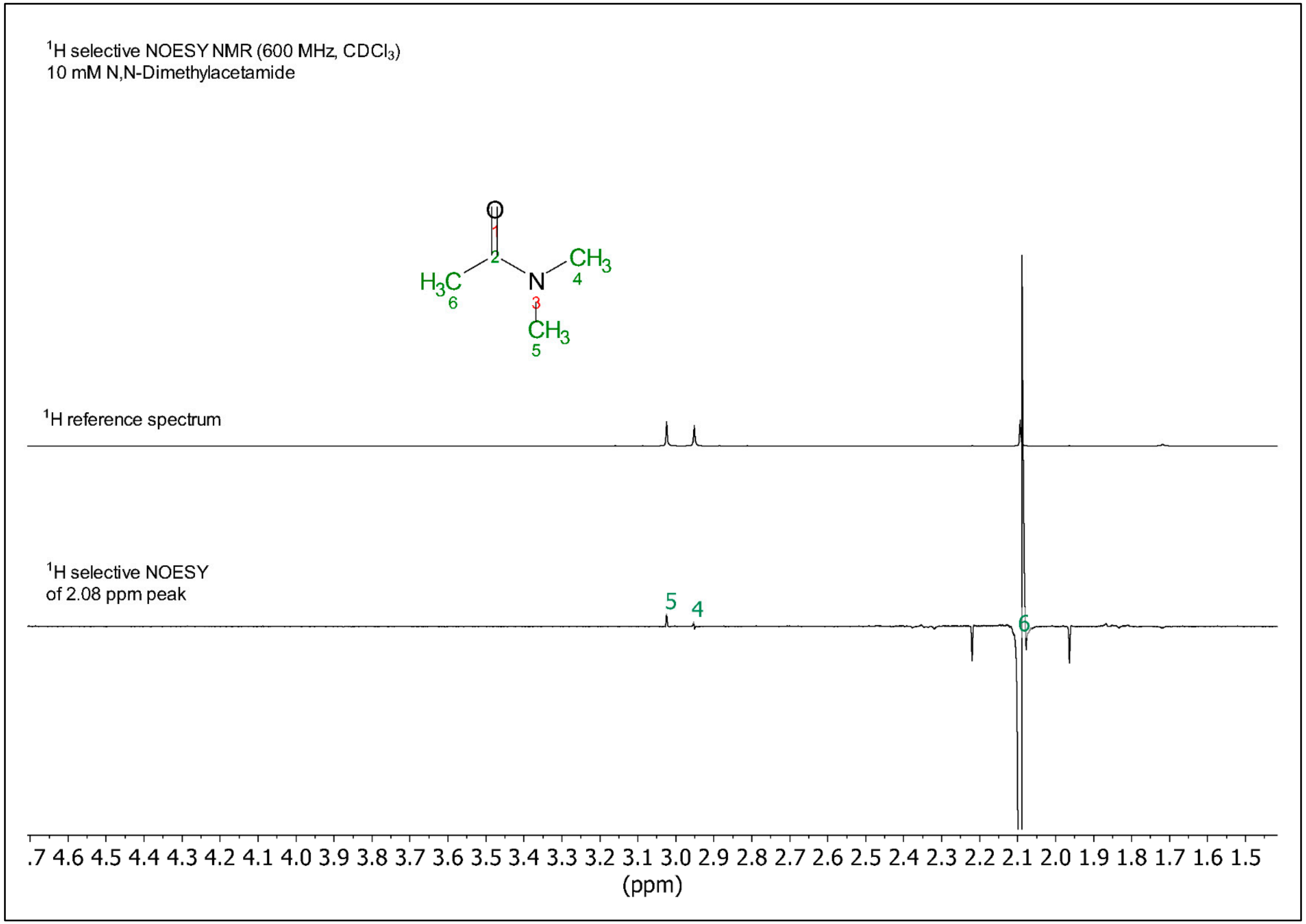

1H selective NOESY NMR (600 MHz, CDCl3)
10 mM N,N-Dimethylacetamide
O
H3C
N
CH3
CH3
1H reference spectrum
1H selective NOESY
of 2.08 ppm peak
5
4
6
.7 4.6 4.5 4.4 4.3 4.2 4.1 4.0 3.9 3.8 3.7 3.6 3.5 3.4 3.3 3.2 3.1 3.0 2.9 2.8 2.7 2.6 2.5 2.4 2.3 2.2 2.1 2.0 1.9 1.8 1.7 1.6 1.5
(ppm)

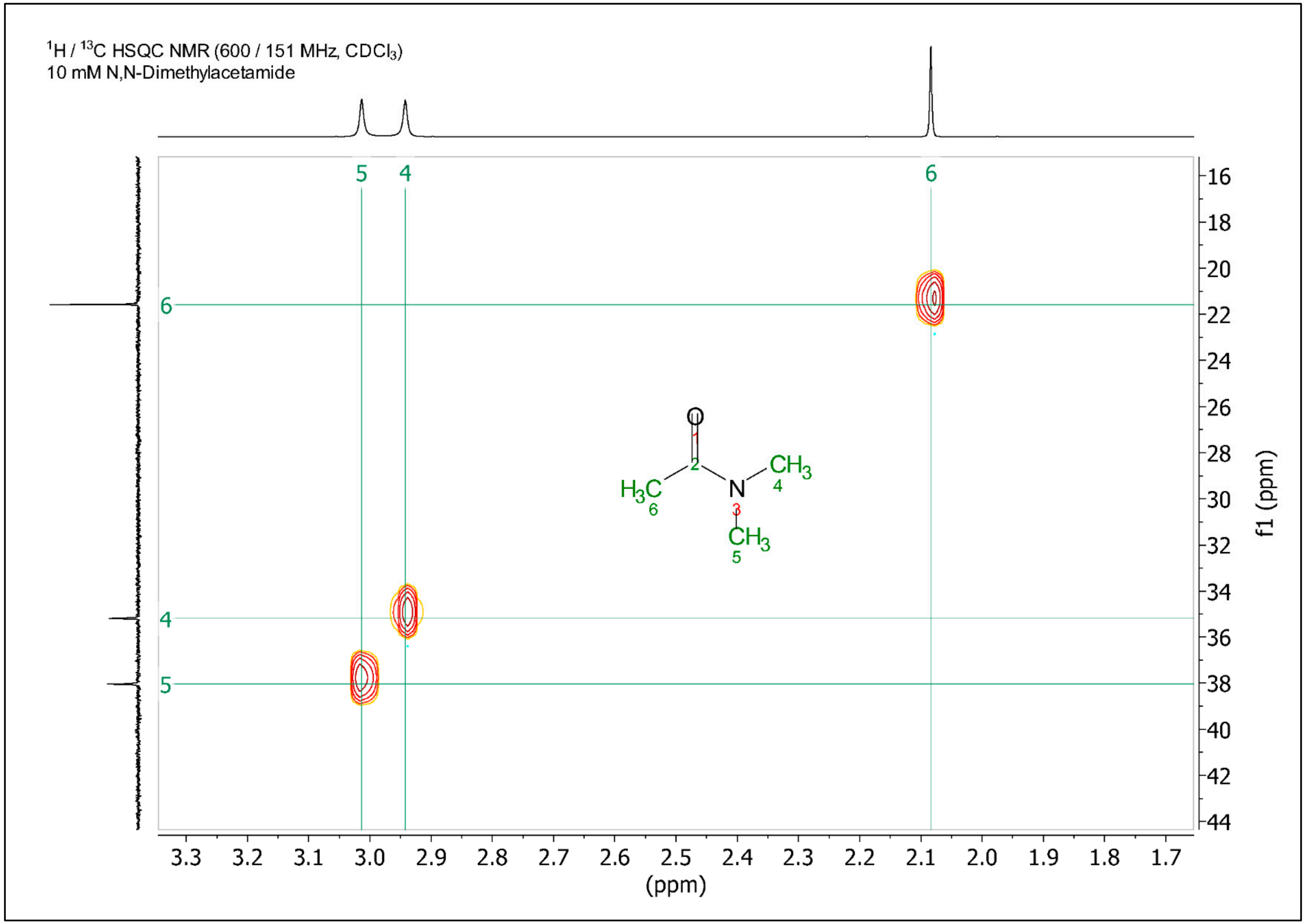

1H / 13C HSQC NMR (600 / 151 MHz, CDCl3)
10 mM N,N-Dimethylacetamide
f1 (ppm)
(ppm)

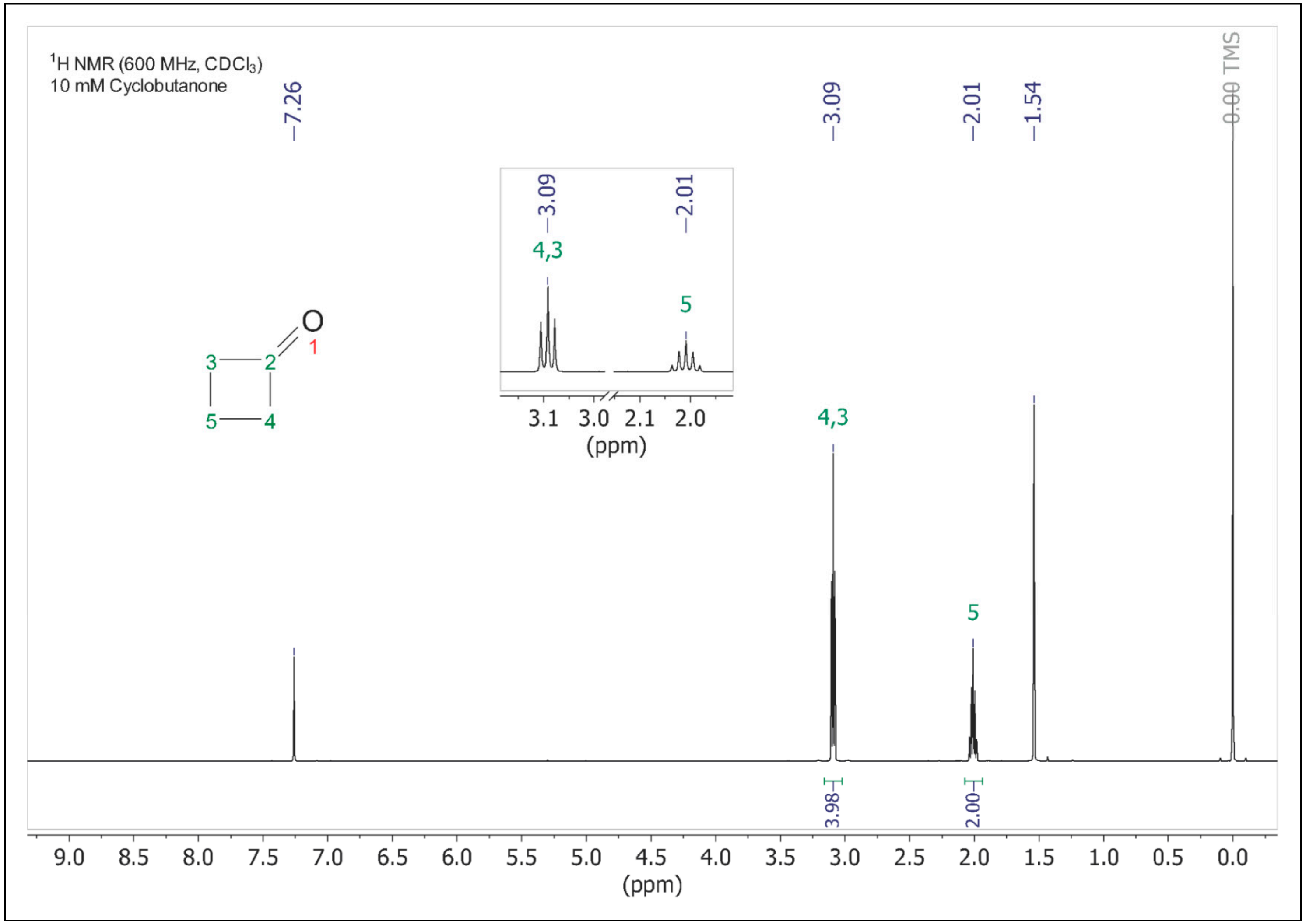

^{1}H NMR (600 MHz, CDCl$_3$)
10 mM Cyclobutanone
7.26
3.09
2.01
1.54
0.00 TMS
4,3
5
3.98
2.00
9.0 8.5 8.0 7.5 7.0 6.5 6.0 5.5 5.0 4.5 4.0 3.5 3.0 2.5 2.0 1.5 1.0 0.5 0.0
(ppm)
3.1 3.0 2.1 2.0
(ppm)

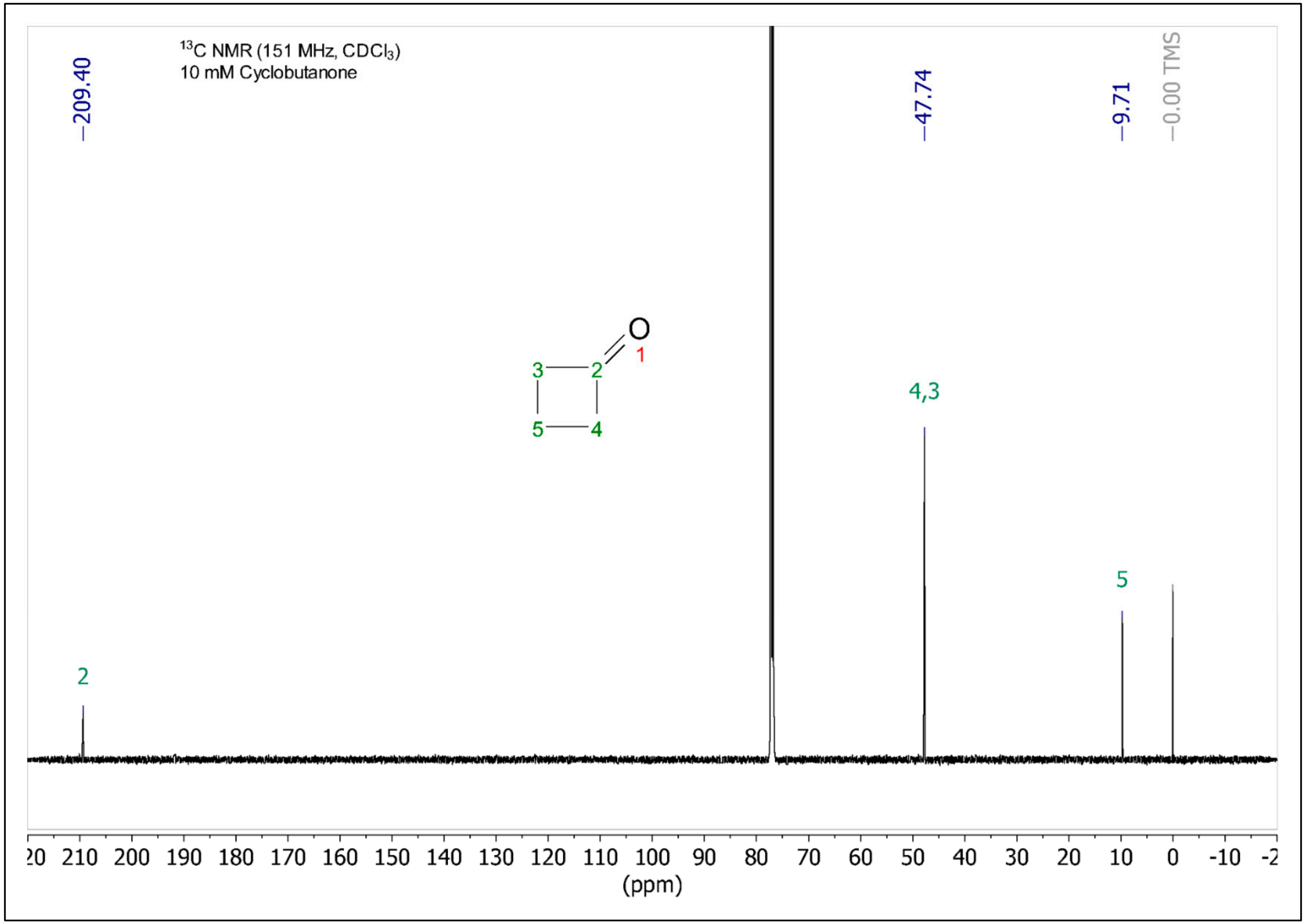

13C NMR (151 MHz, CDCl3)
10 mM Cyclobutanone
209.40
47.74
9.71
0.00 TMS
2
4,3
5
(ppm)

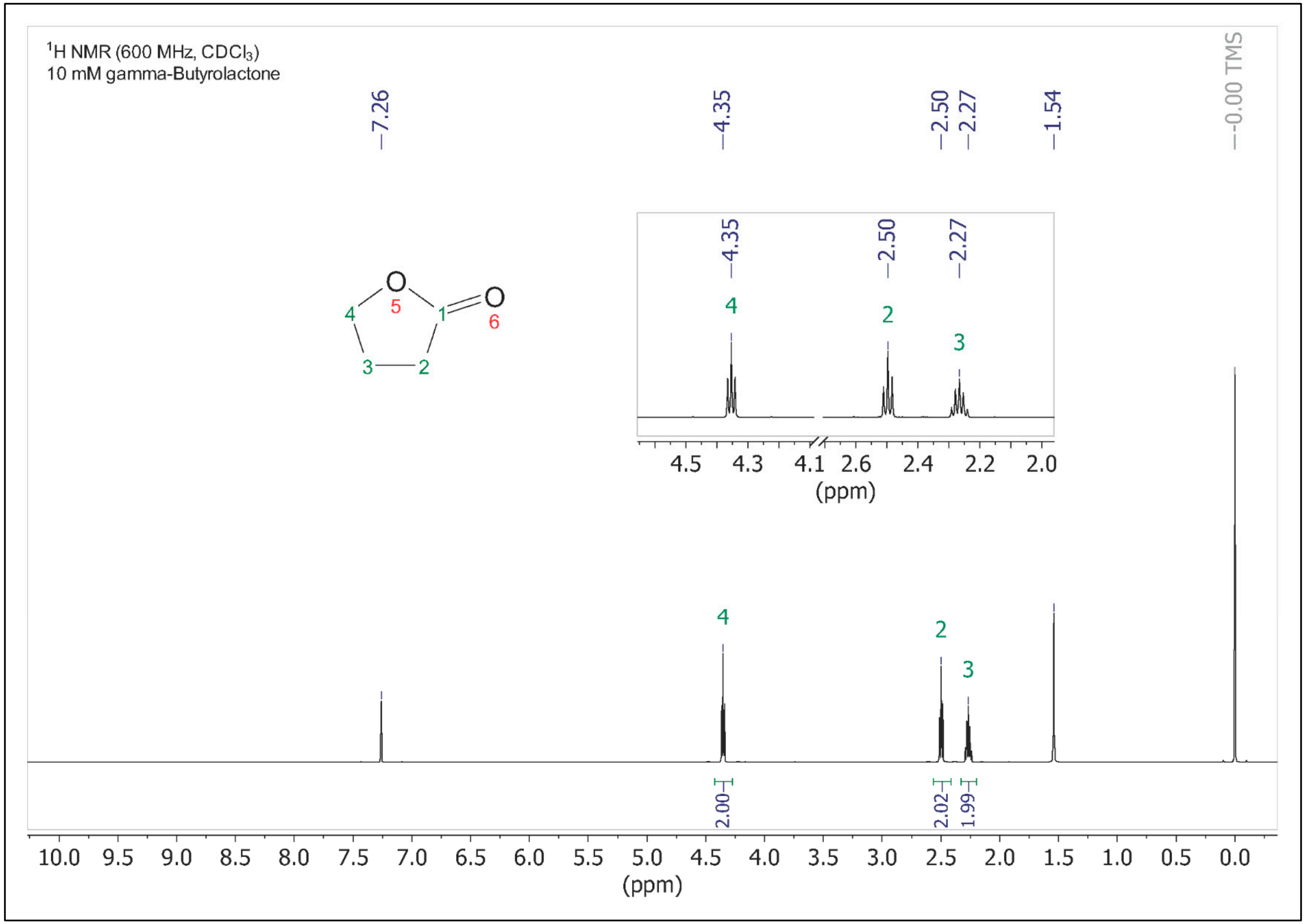

^{1}H NMR (600 MHz, CDCl3)
10 mM gamma-Butyrolactone
7.26
4.35
2.50
2.27
1.54
0.00 TMS
4
2
3
2.00
2.02
1.99
(ppm)

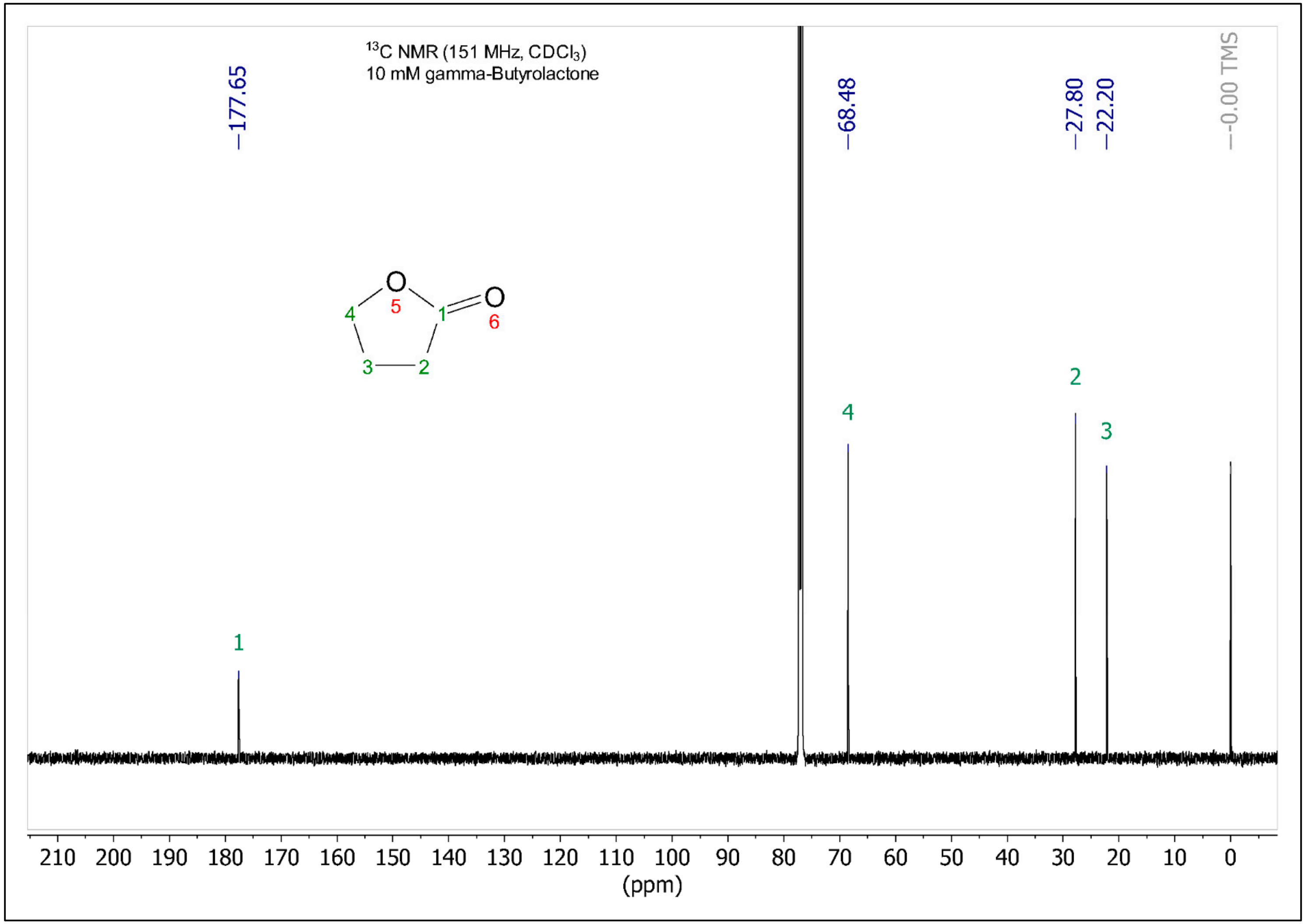

^{13}C NMR (151 MHz, $CDCl_3$)
10 mM gamma-Butyrolactone
177.65
68.48
27.80
22.20
-0.00 TMS
O
O
1
2
3
4
5
6
210 200 190 180 170 160 150 140 130 120 110 100 90 80 70 60 50 40 30 20 10 0
(ppm)

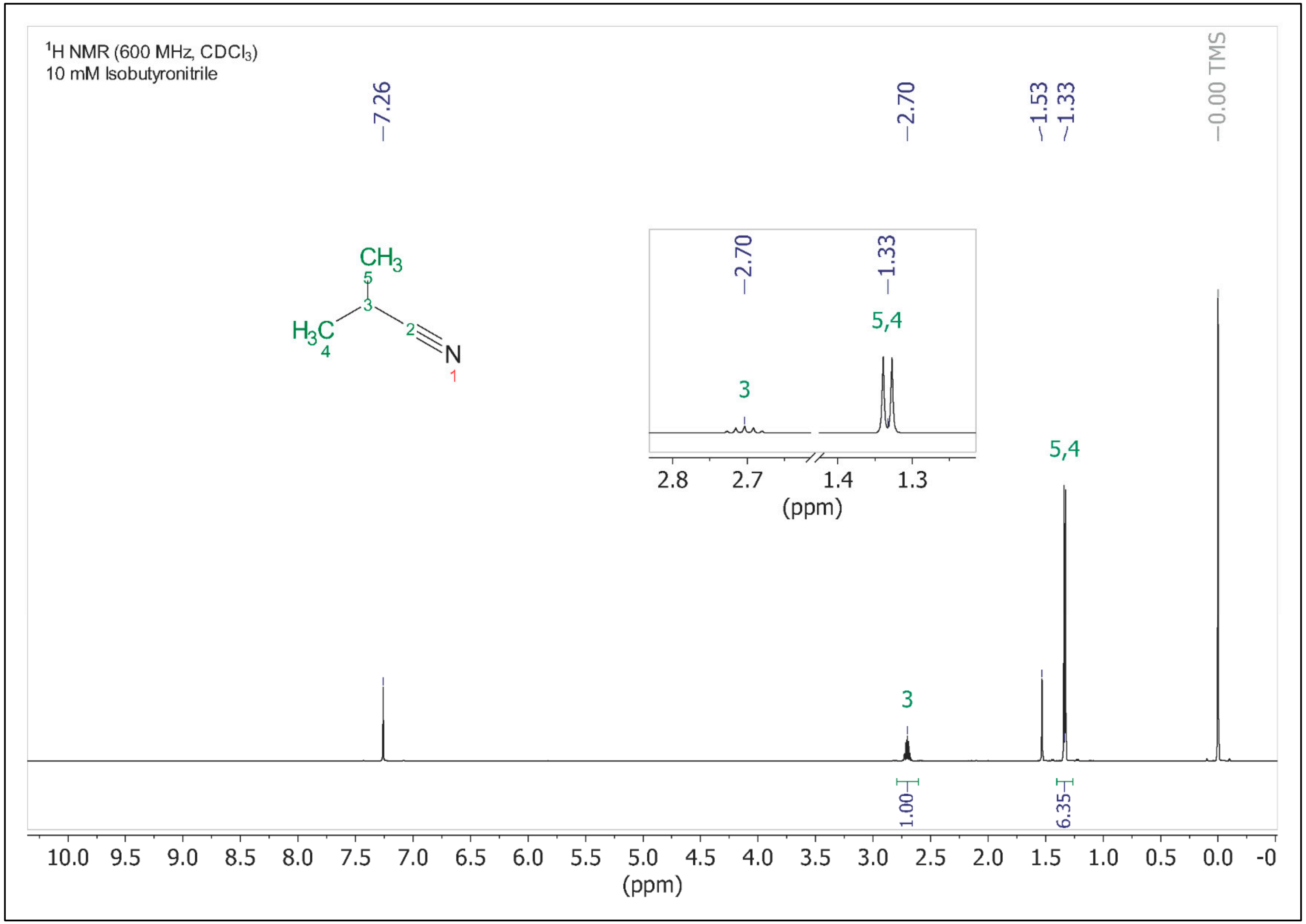

1H NMR (600 MHz, CDCl3)
10 mM Isobutyronitrile
7.26
2.70
1.53
1.33
0.00 TMS
CH3
H3C
N
1
2
3
4
5
5,4
3
2.8
2.7
1.4
1.3
(ppm)
1.00
6.35
10.0 9.5 9.0 8.5 8.0 7.5 7.0 6.5 6.0 5.5 5.0 4.5 4.0 3.5 3.0 2.5 2.0 1.5 1.0 0.5 0.0 -0
(ppm)

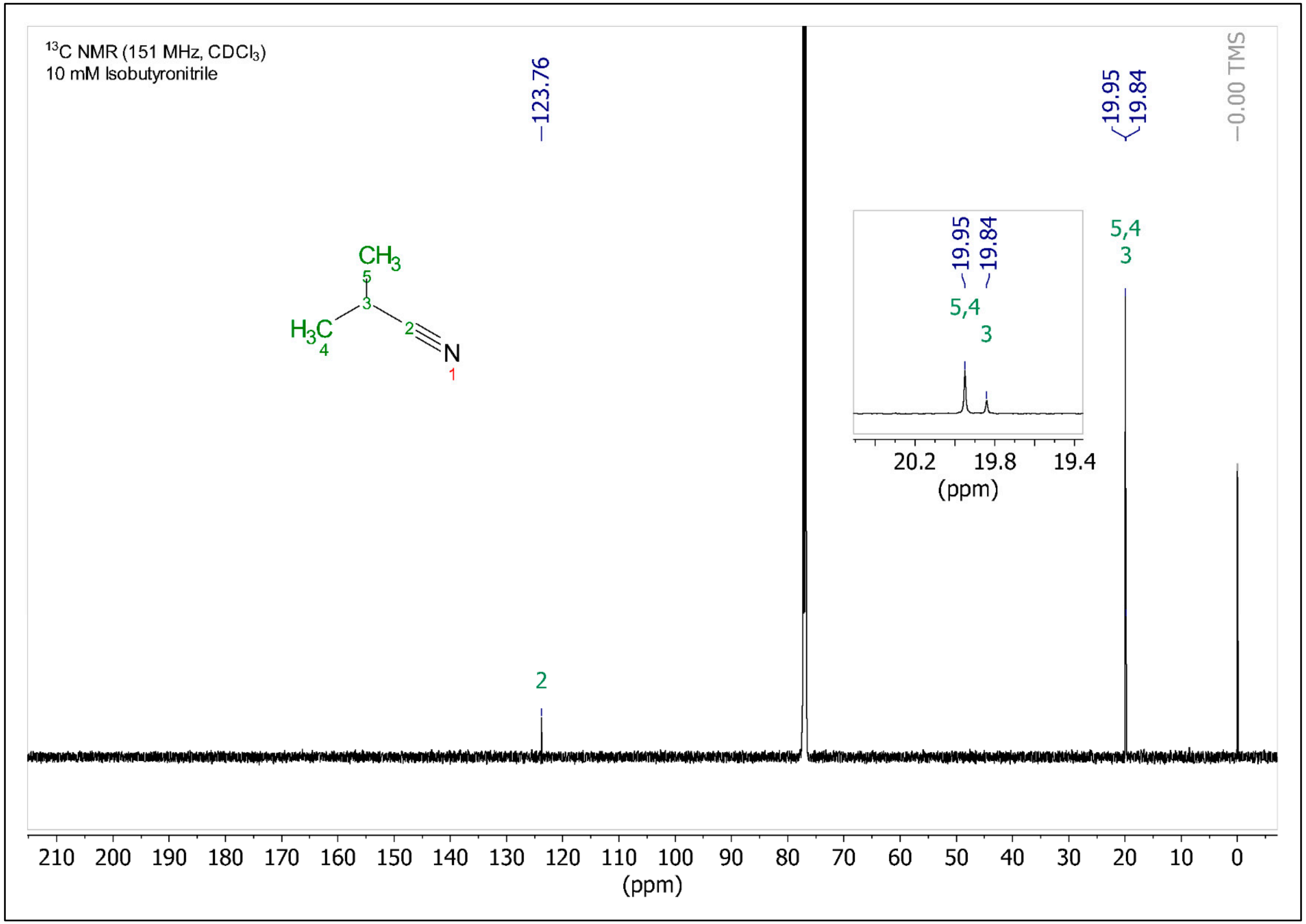

13C NMR (151 MHz, CDCl3)
10 mM Isobutyronitrile
123.76
19.95
19.84
0.00 TMS
CH3
H3C
N
5,4
3
2
20.2
19.8
19.4
(ppm)
210
200
190
180
170
160
150
140
130
120
110
100
90
80
70
60
50
40
30
20
10
0
(ppm)

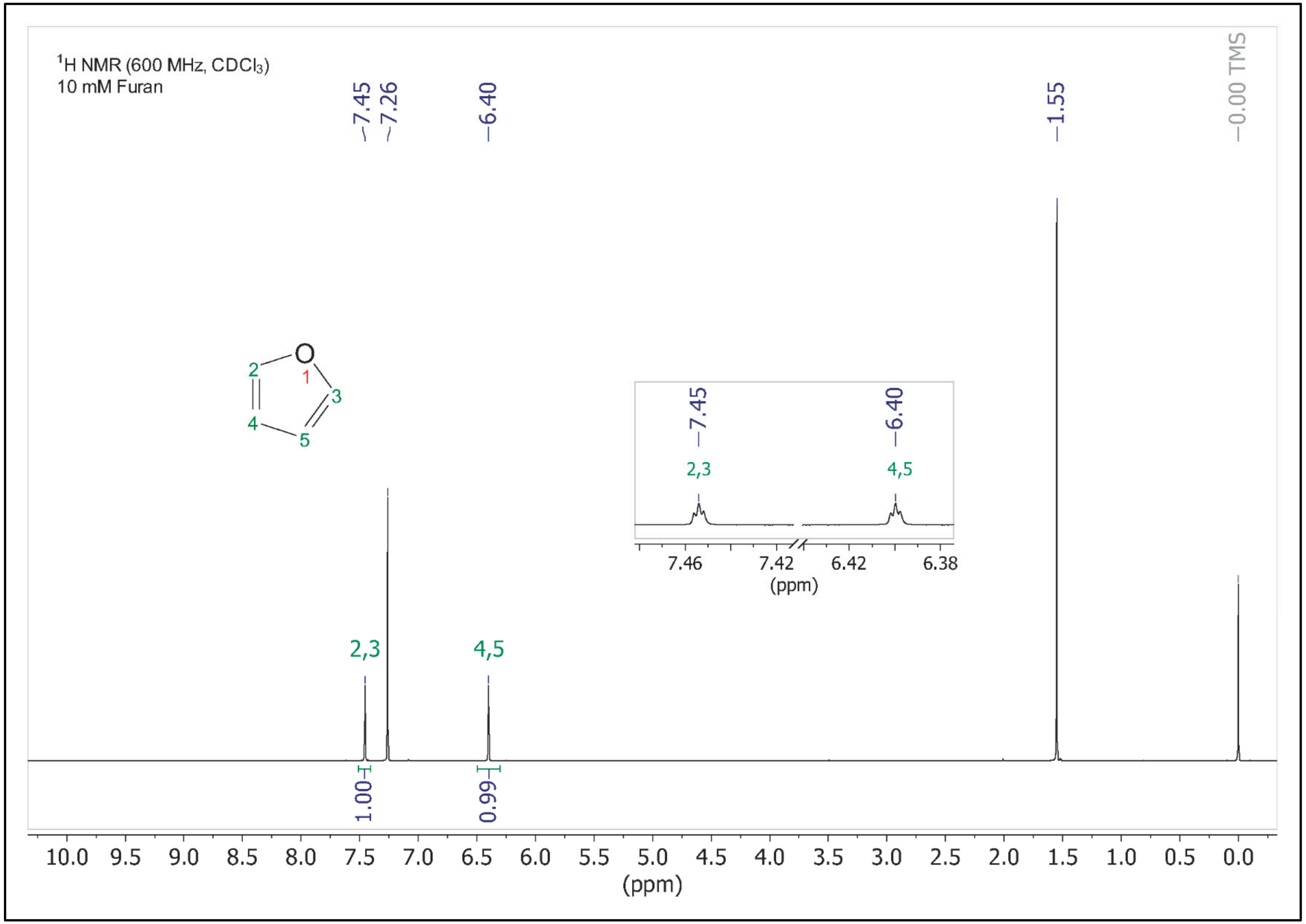

¹H NMR (600 MHz, CDCl3)
10 mM Furan
7.45
7.26
6.40
1.55
0.00 TMS
O
1
2
3
4
5
2,3
4,5
1.00
0.99
7.46
7.42
6.42
6.38
(ppm)
10.0
9.5
9.0
8.5
8.0
7.5
7.0
6.5
6.0
5.5
5.0
4.5
4.0
3.5
3.0
2.5
2.0
1.5
1.0
0.5
0.0
(ppm)

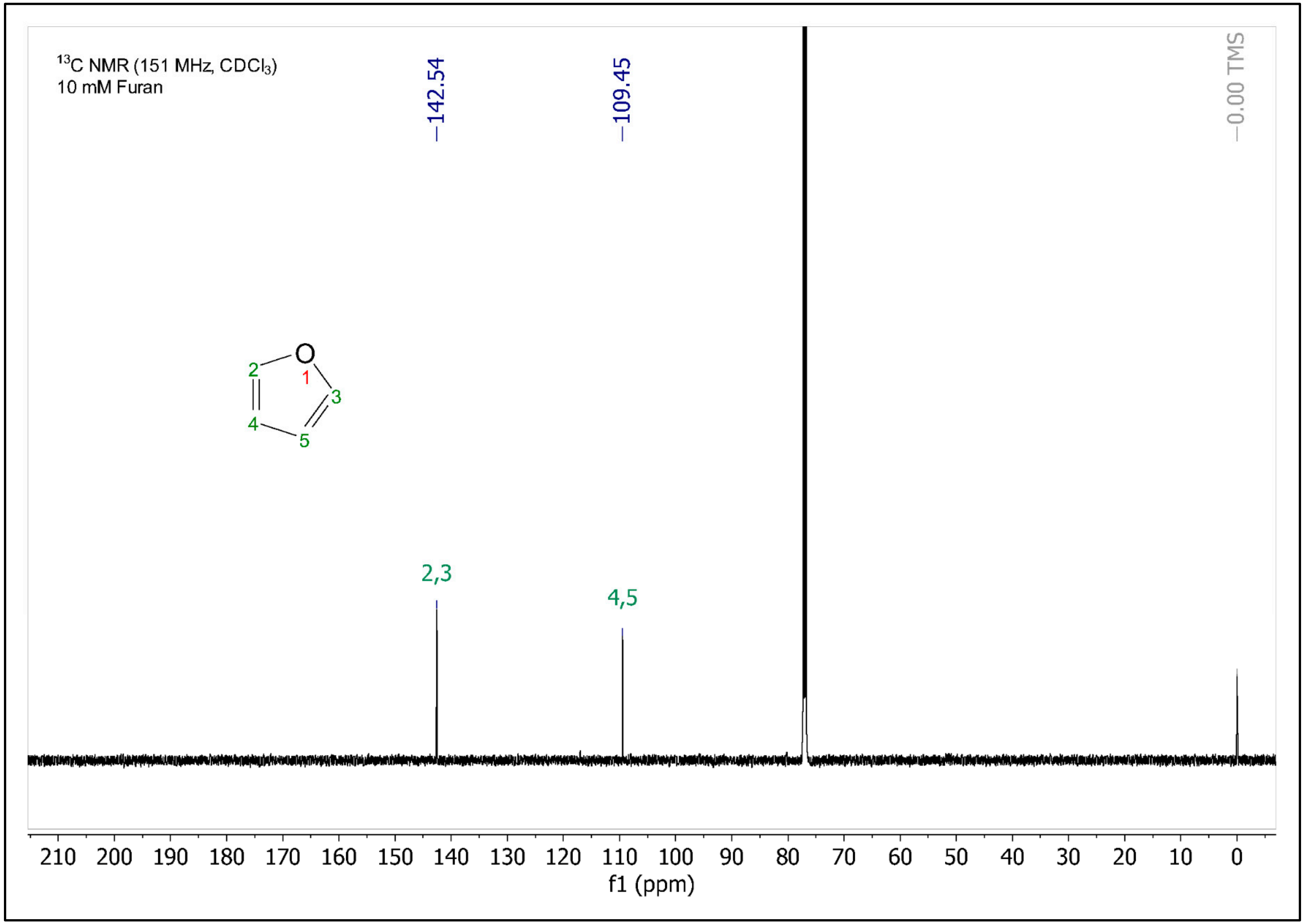

13C NMR (151 MHz, CDCl3)
10 mM Furan
—142.54
—109.45
—0.00 TMS
O
1
2
3
4
5
2,3
4,5
210 200 190 180 170 160 150 140 130 120 110 100 90 80 70 60 50 40 30 20 10 0
f1 (ppm)

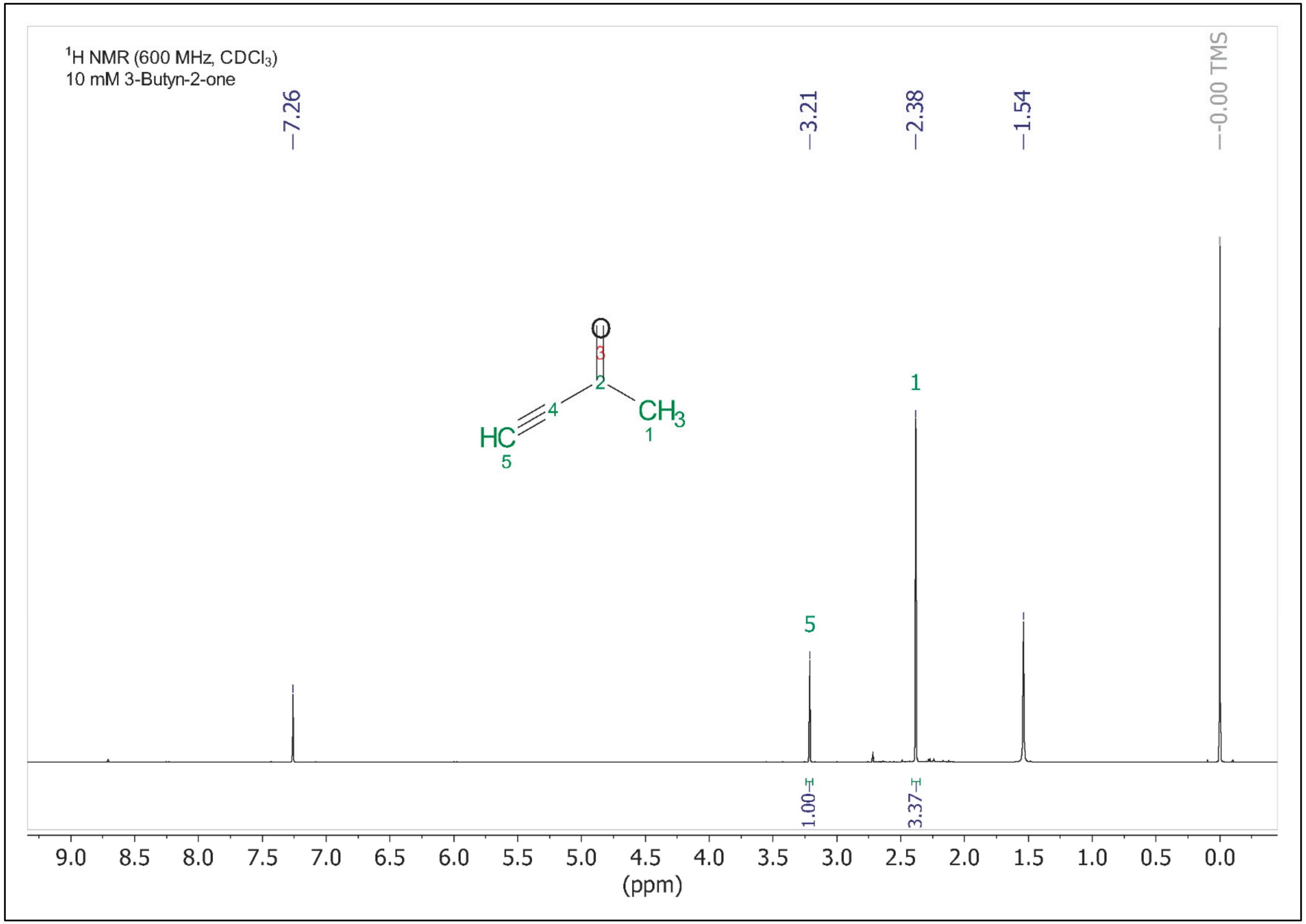

^{1}H NMR (600 MHz, $CDCl_3$)
10 mM 3-Butyn-2-one
7.26
3.21
2.38
1.54
0.00 TMS
HC
CH3
1
2
3
4
5
1.00
3.37
9.0 8.5 8.0 7.5 7.0 6.5 6.0 5.5 5.0 4.5 4.0 3.5 3.0 2.5 2.0 1.5 1.0 0.5 0.0
(ppm)

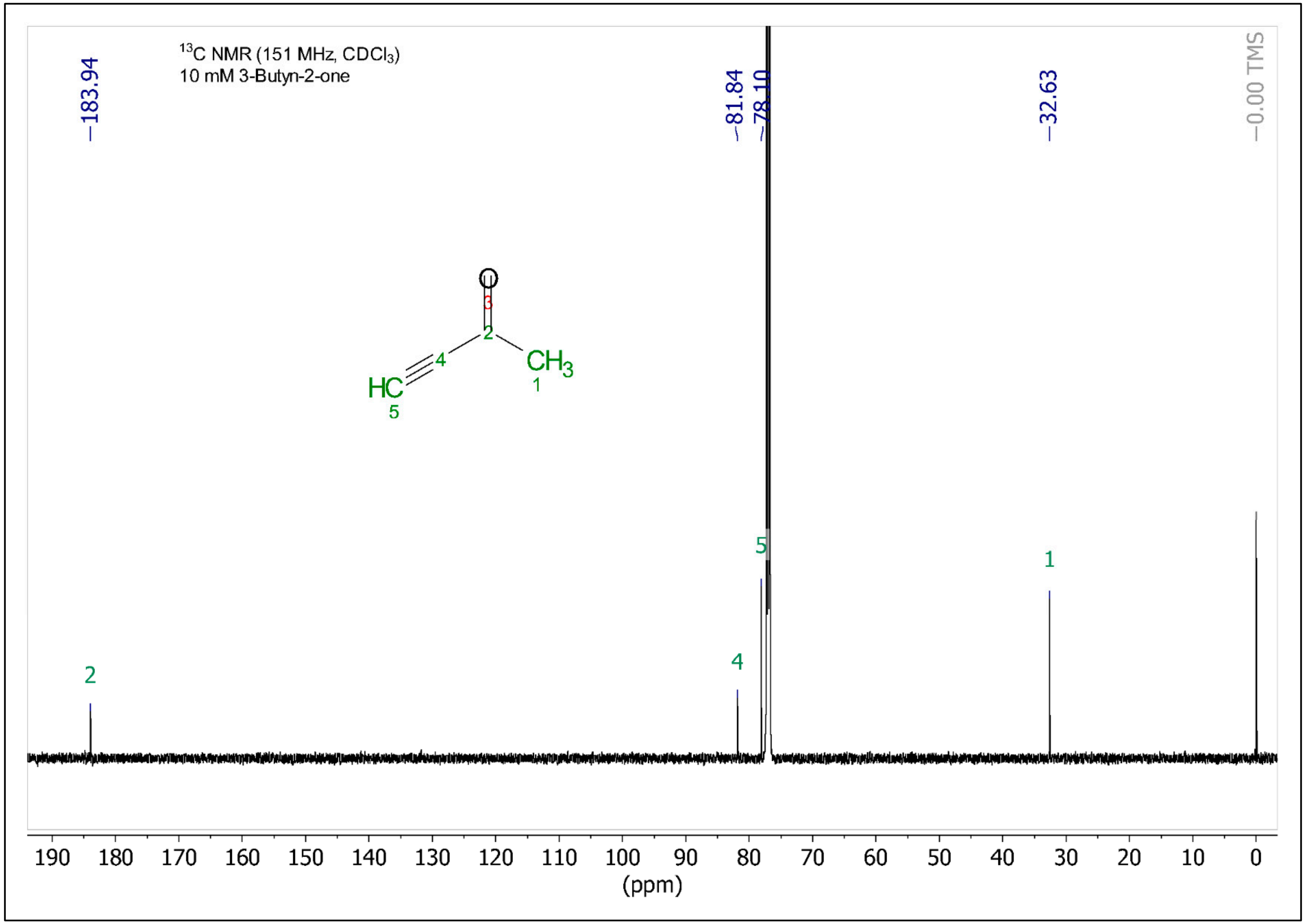

13C NMR (151 MHz, CDCl3)
10 mM 3-Butyn-2-one
183.94
81.84
78.10
32.63
0.00 TMS
HC
CH3
1
2
3
4
5
190 180 170 160 150 140 130 120 110 100 90 80 70 60 50 40 30 20 10 0
(ppm)

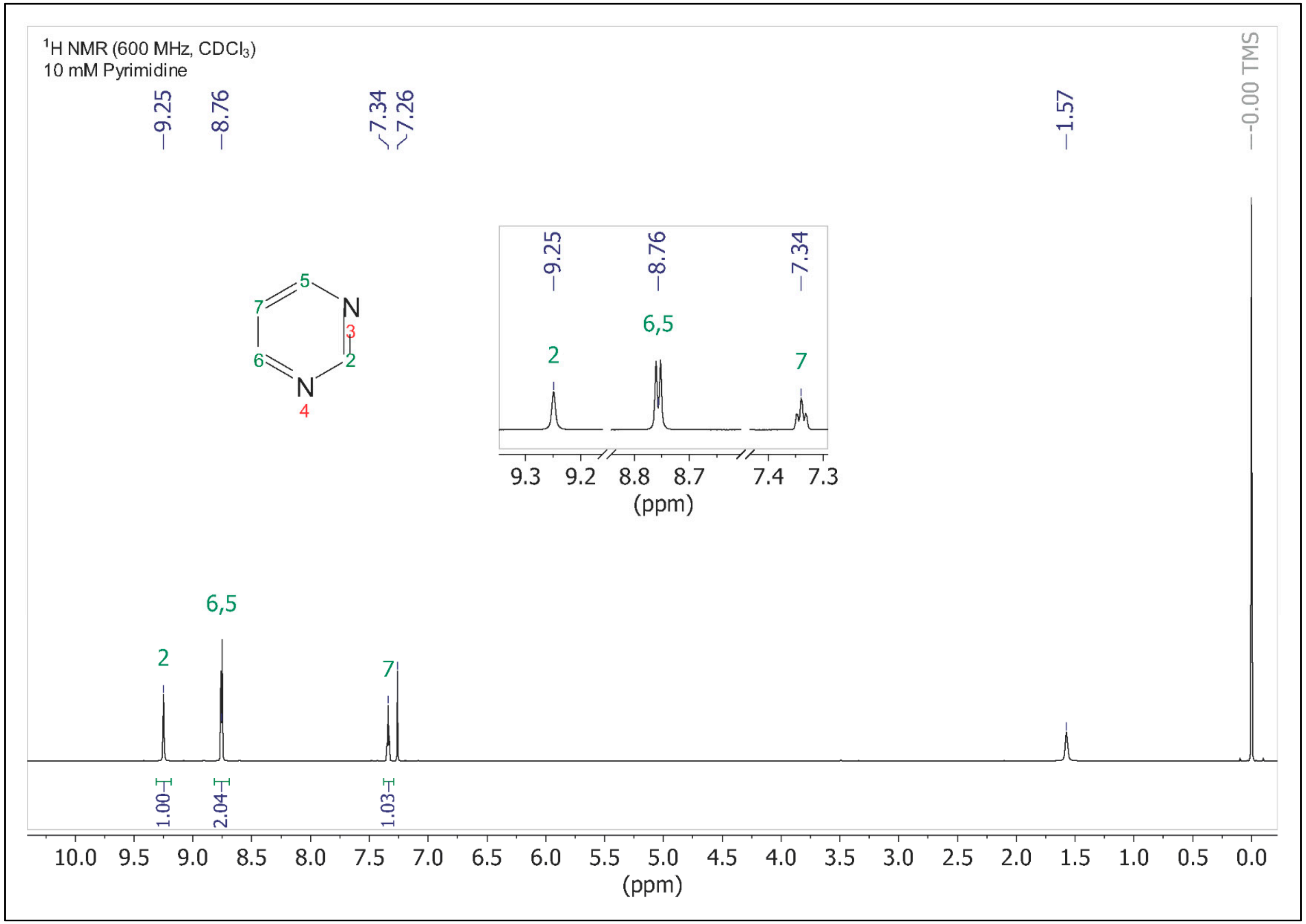

^{1}H NMR (600 MHz, $CDCl_3$)
10 mM Pyrimidine
9.25
8.76
7.34
7.26
1.57
0.00 TMS
2
6,5
7
1.00
2.04
1.03
(ppm)

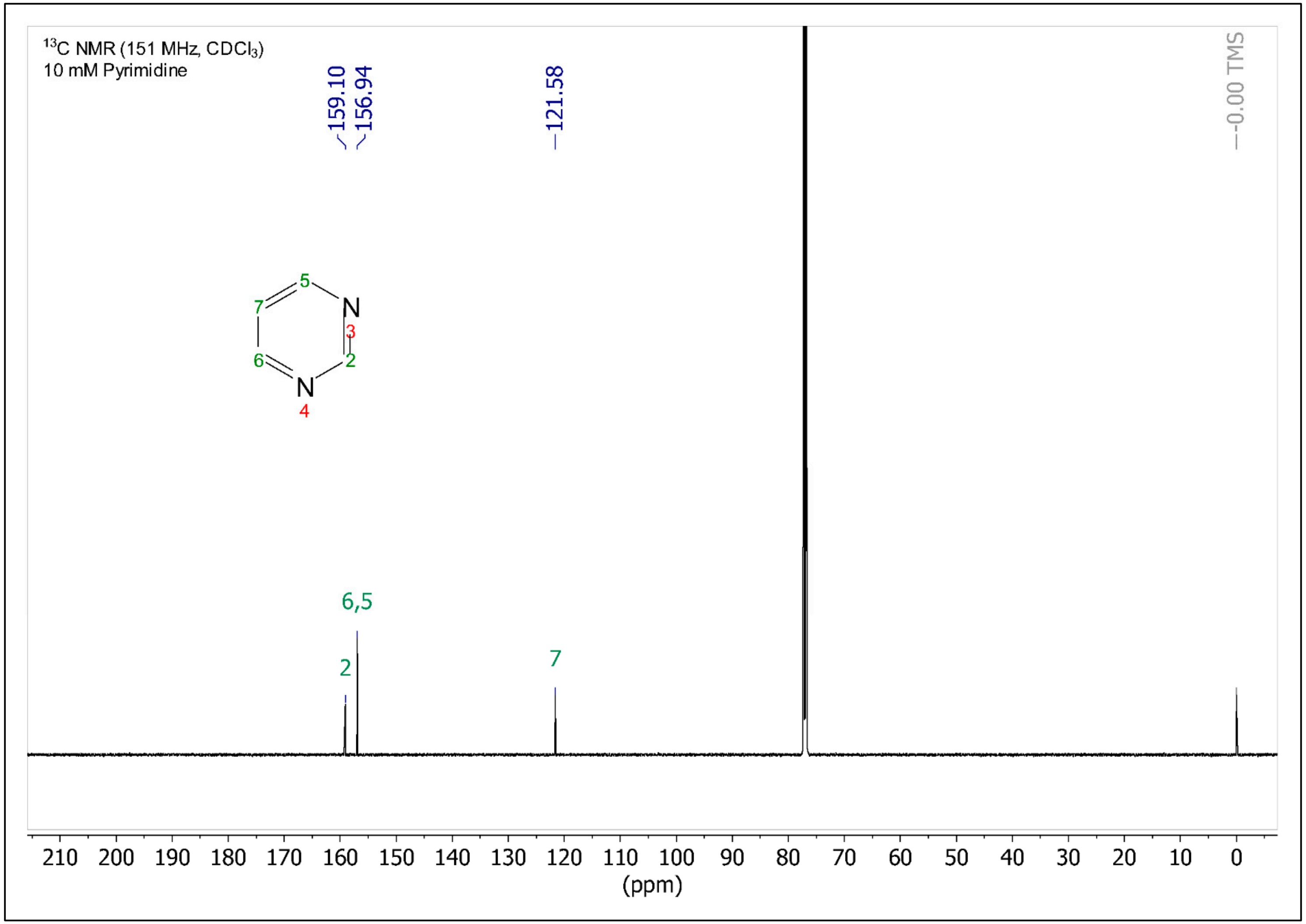

13C NMR (151 MHz, CDCl3)
10 mM Pyrimidine
159.10
156.94
121.58
-0.00 TMS
N
N
6,5
2
7
210 200 190 180 170 160 150 140 130 120 110 100 90 80 70 60 50 40 30 20 10 0
(ppm)

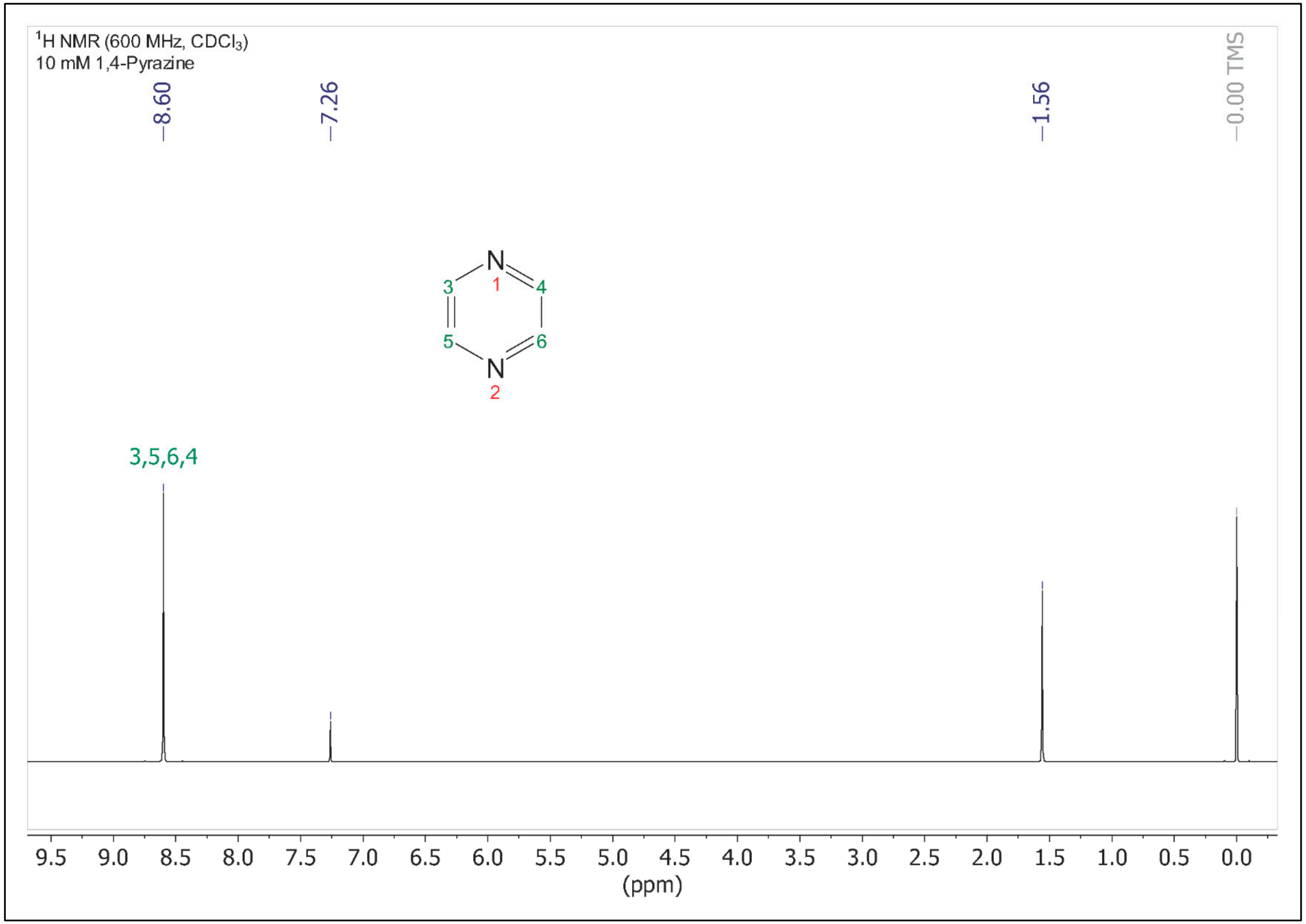

1H NMR (600 MHz, CDCl3)
10 mM 1,4-Pyrazine
8.60
7.26
1.56
0.00 TMS
N
1
N
2
3
4
5
6
3,5,6,4
9.5 9.0 8.5 8.0 7.5 7.0 6.5 6.0 5.5 5.0 4.5 4.0 3.5 3.0 2.5 2.0 1.5 1.0 0.5 0.0
(ppm)

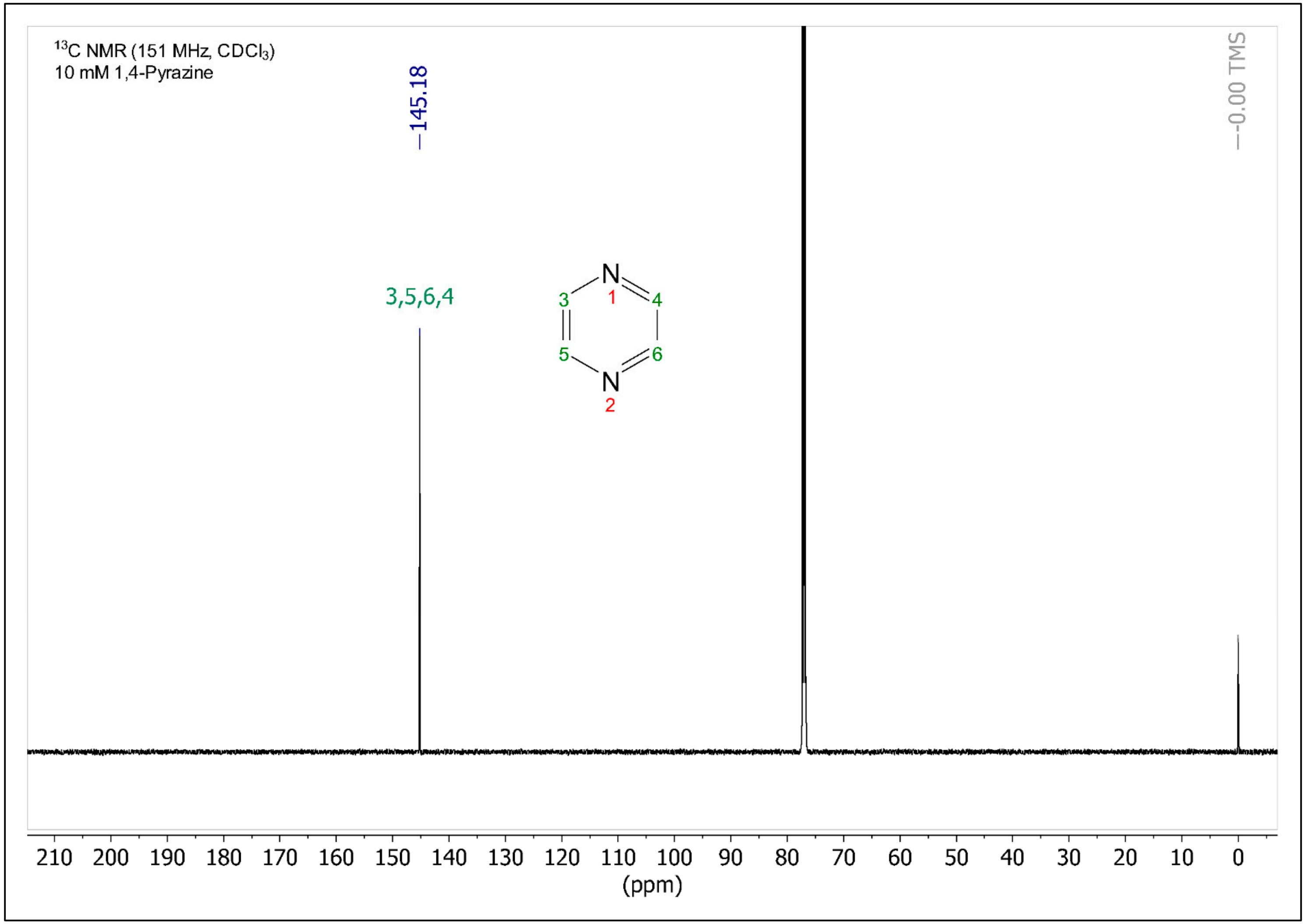

13C NMR (151 MHz, CDCl3)
10 mM 1,4-Pyrazine
145.18
-0.00 TMS
3,5,6,4
N
N
1
2
3
4
5
6
210 200 190 180 170 160 150 140 130 120 110 100 90 80 70 60 50 40 30 20 10 0
(ppm)

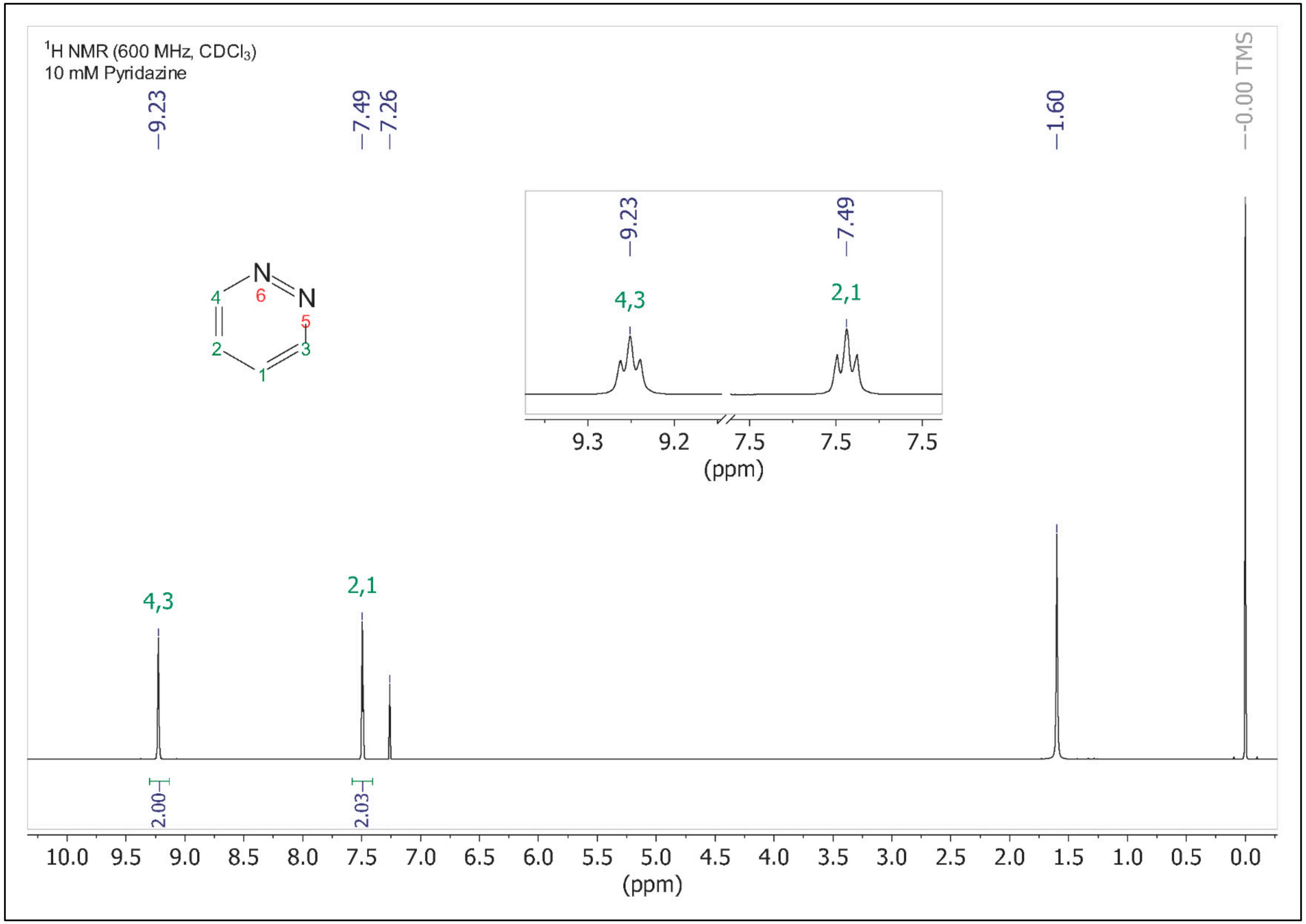

1H NMR (600 MHz, CDCl3)
10 mM Pyridazine
9.23
7.49
7.26
1.60
0.00 TMS
4,3
2,1
2.00
2.03
(ppm)

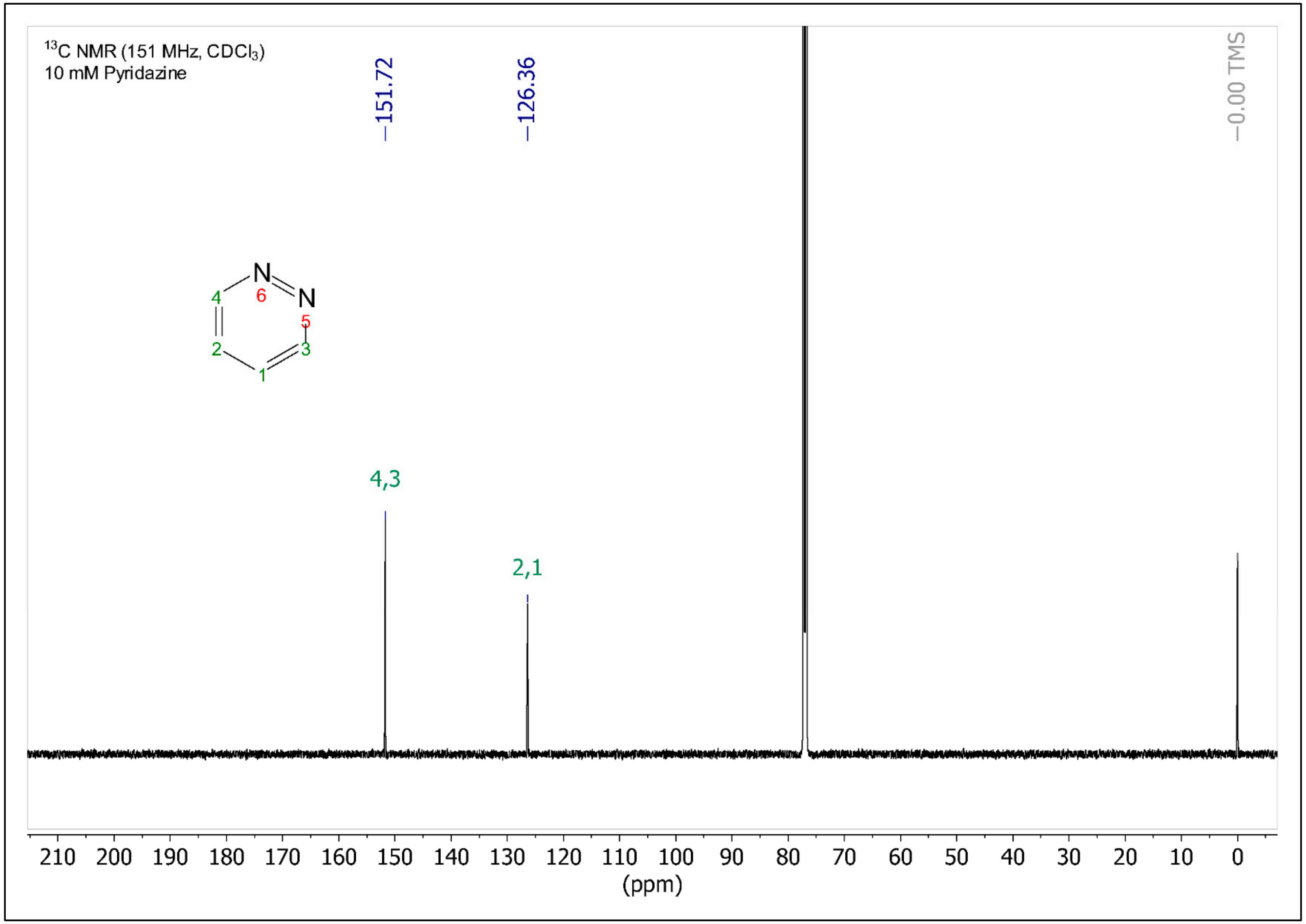

^{13}C NMR (151 MHz, $CDCl_3$)
10 mM Pyridazine
151.72
126.36
0.00 TMS
4,3
2,1
210 200 190 180 170 160 150 140 130 120 110 100 90 80 70 60 50 40 30 20 10 0
(ppm)

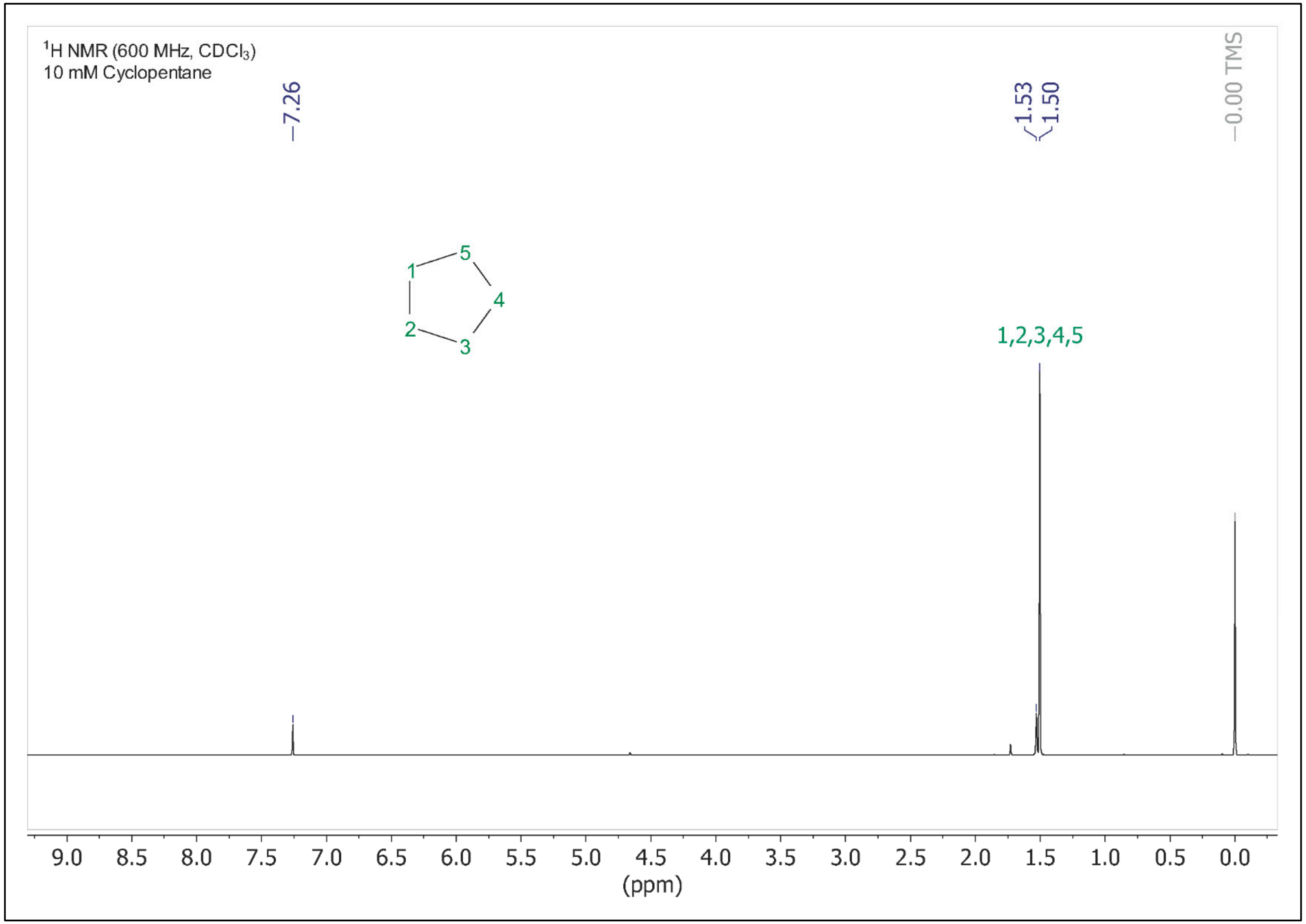

1H NMR (600 MHz, CDCl3)
10 mM Cyclopentane
7.26
1.53
1.50
0.00 TMS
1
2
3
4
5
1,2,3,4,5
9.0 8.5 8.0 7.5 7.0 6.5 6.0 5.5 5.0 4.5 4.0 3.5 3.0 2.5 2.0 1.5 1.0 0.5 0.0
(ppm)

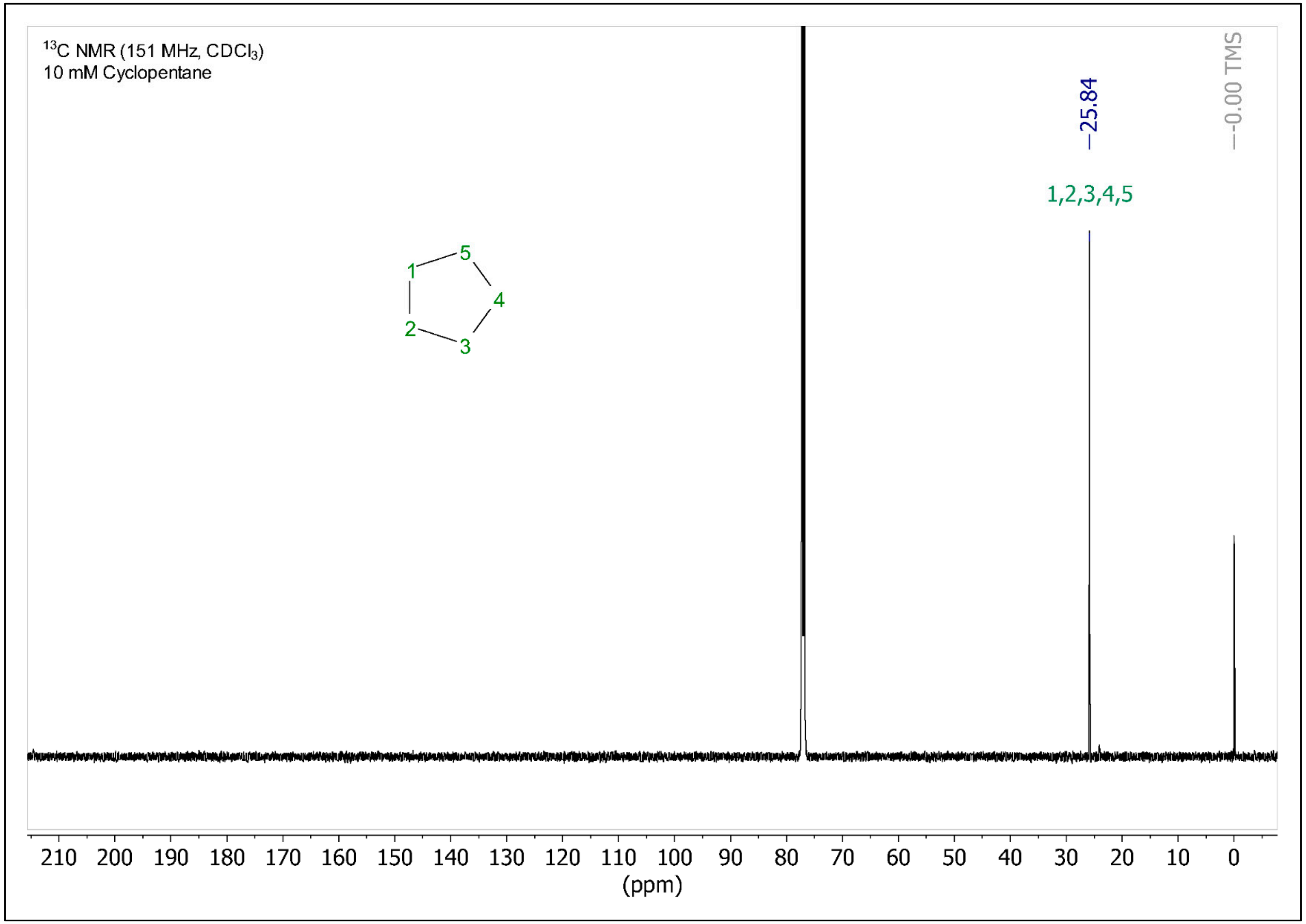

^{13}C NMR (151 MHz, CDCl$_3$)
10 mM Cyclopentane
—25.84
—0.00 TMS
1,2,3,4,5
1
2
3
4
5
210 200 190 180 170 160 150 140 130 120 110 100 90 80 70 60 50 40 30 20 10 0
(ppm)

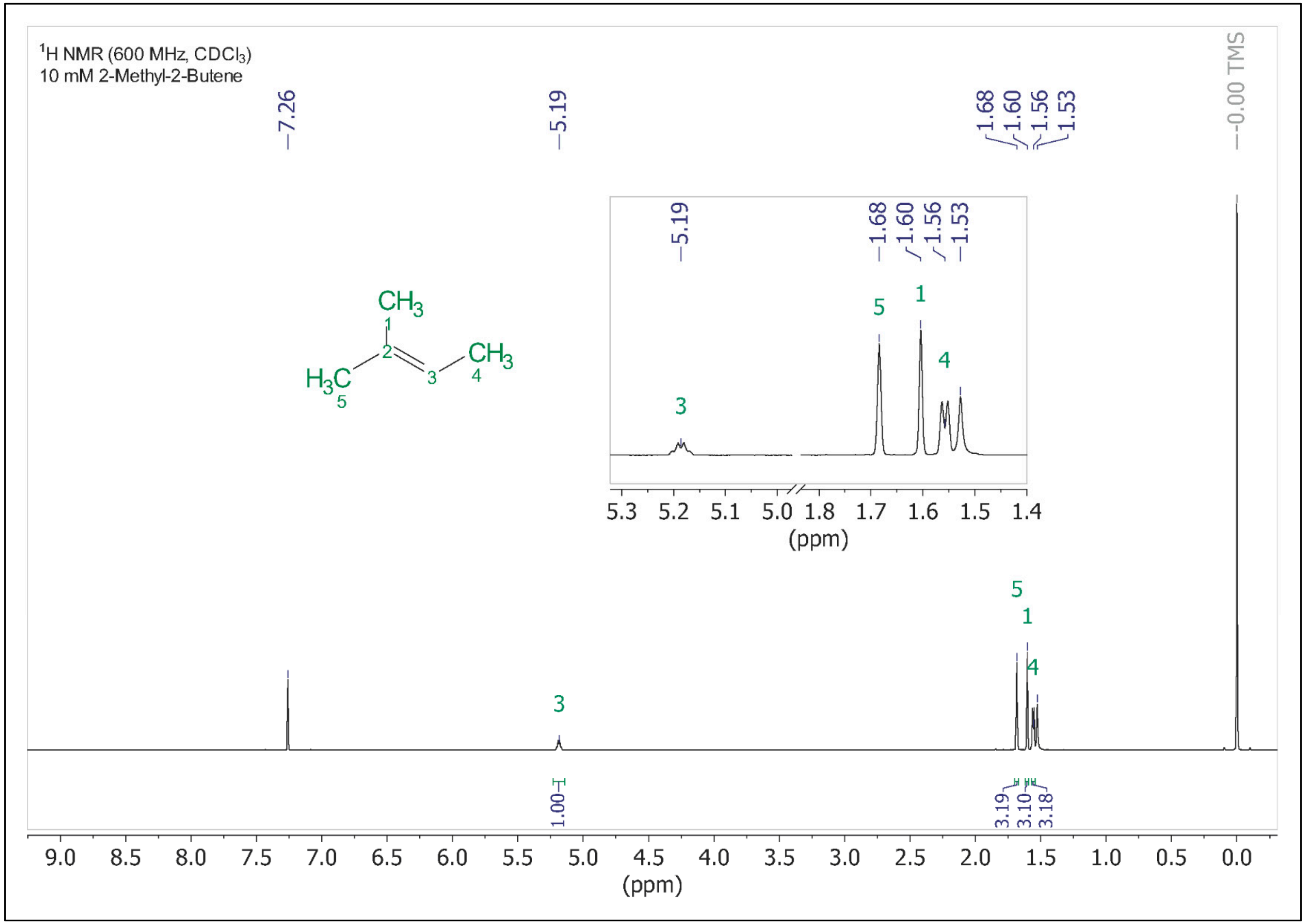

1H NMR (600 MHz, CDCl3)
10 mM 2-Methyl-2-Butene
7.26
5.19
1.68
1.60
1.56
1.53
0.00 TMS
CH3
H3C
1
2
3
4
5
1.00
3.19
3.10
3.18
9.0 8.5 8.0 7.5 7.0 6.5 6.0 5.5 5.0 4.5 4.0 3.5 3.0 2.5 2.0 1.5 1.0 0.5 0.0
(ppm)
5.3 5.2 5.1 5.0 1.8 1.7 1.6 1.5 1.4
(ppm)

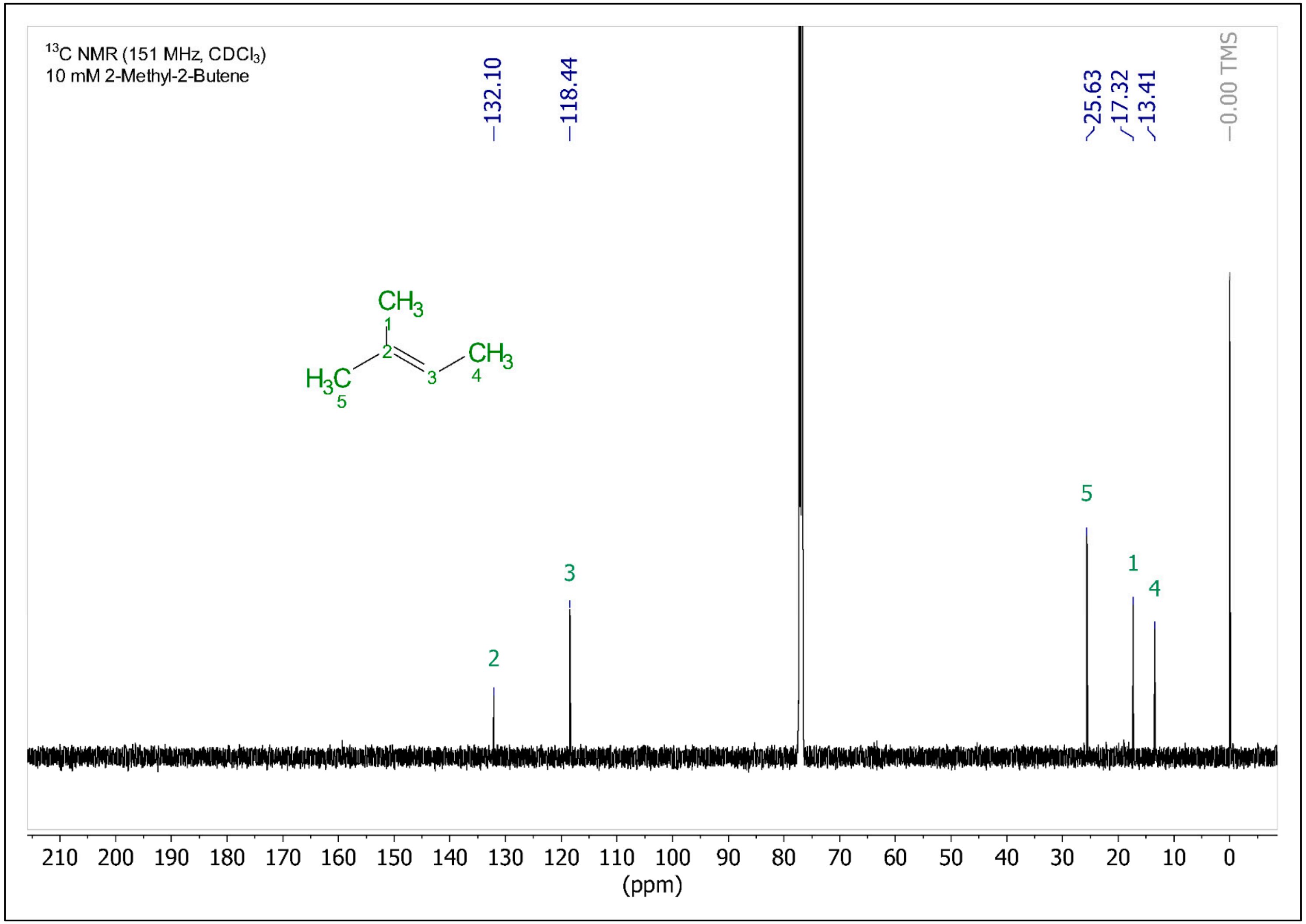

13C NMR (151 MHz, CDCl3)
10 mM 2-Methyl-2-Butene
132.10
118.44
25.63
17.32
13.41
0.00 TMS
CH3
H3C
CH3
1
2
3
4
5
210 200 190 180 170 160 150 140 130 120 110 100 90 80 70 60 50 40 30 20 10 0
(ppm)

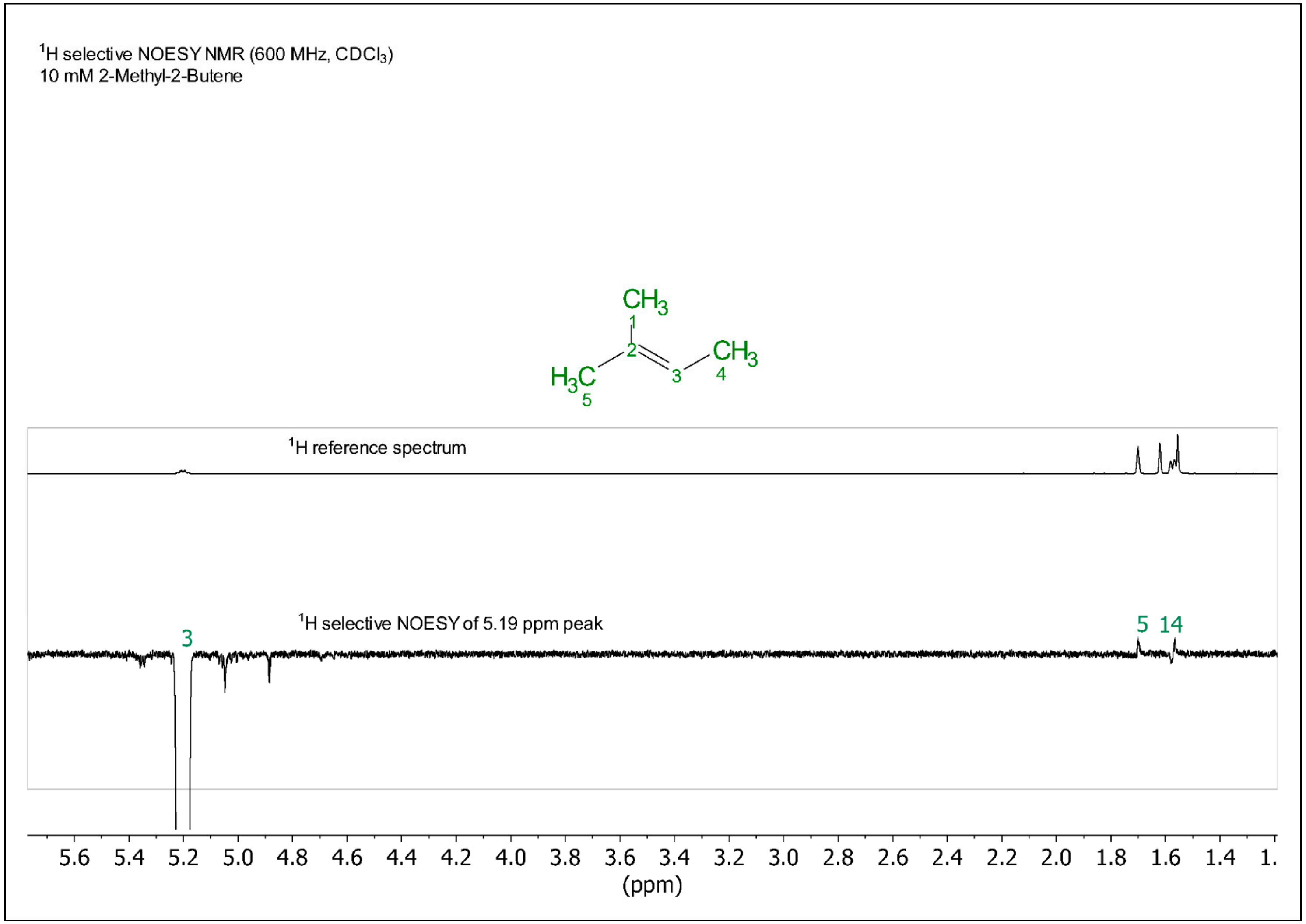

1H selective NOESY NMR (600 MHz, CDCl3)
10 mM 2-Methyl-2-Butene
CH3
1
2
H3C
5
3
CH3
4
1H reference spectrum
1H selective NOESY of 5.19 ppm peak
3
5 14
5.6 5.4 5.2 5.0 4.8 4.6 4.4 4.2 4.0 3.8 3.6 3.4 3.2 3.0 2.8 2.6 2.4 2.2 2.0 1.8 1.6 1.4 1.
(ppm)

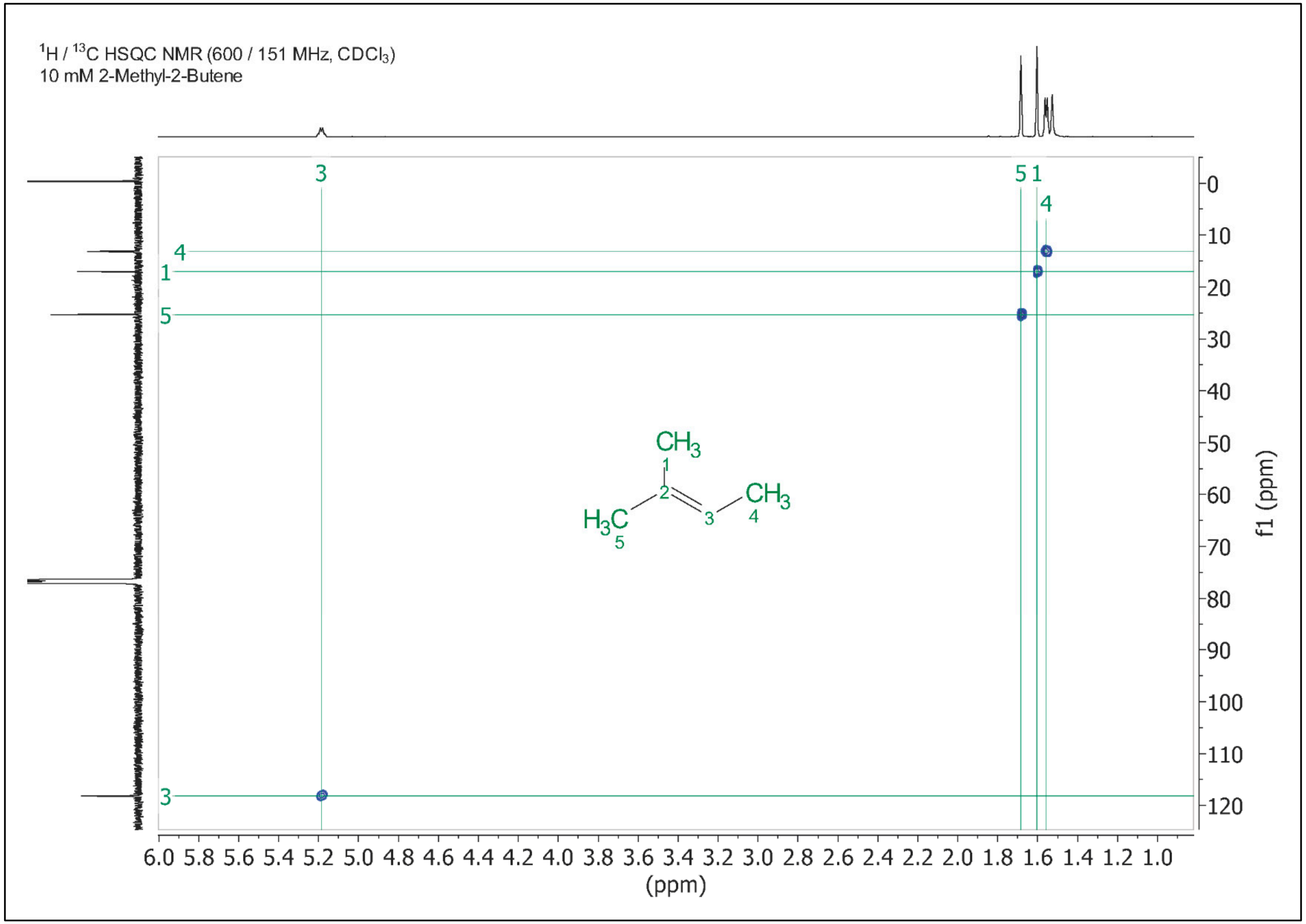

^{1}H / ^{13}C HSQC NMR (600 / 151 MHz, $CDCl_3$)
10 mM 2-Methyl-2-Butene
3
5 1
4
4
1
5
3
CH_3
1
2
CH_3
H_3C
3
4
5
0
10
20
30
40
50
60
70
80
90
100
110
120
f1 (ppm)
6.0 5.8 5.6 5.4 5.2 5.0 4.8 4.6 4.4 4.2 4.0 3.8 3.6 3.4 3.2 3.0 2.8 2.6 2.4 2.2 2.0 1.8 1.6 1.4 1.2 1.0
(ppm)

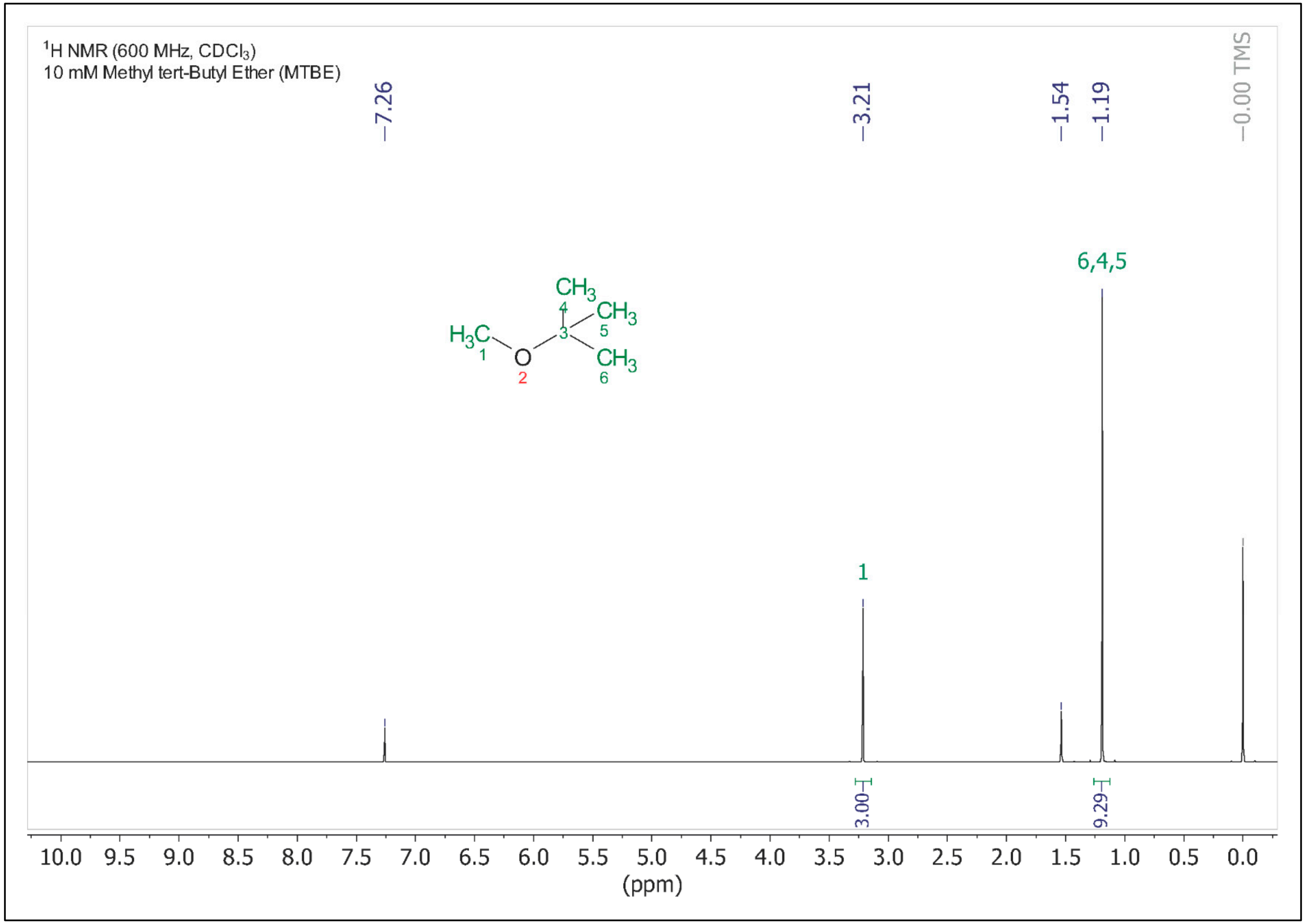

^{1}H NMR (600 MHz, CDCl$_3$)
10 mM Methyl tert-Butyl Ether (MTBE)
7.26
3.21
1.54
1.19
0.00 TMS
6,4,5
1
3.00
9.29
(ppm)

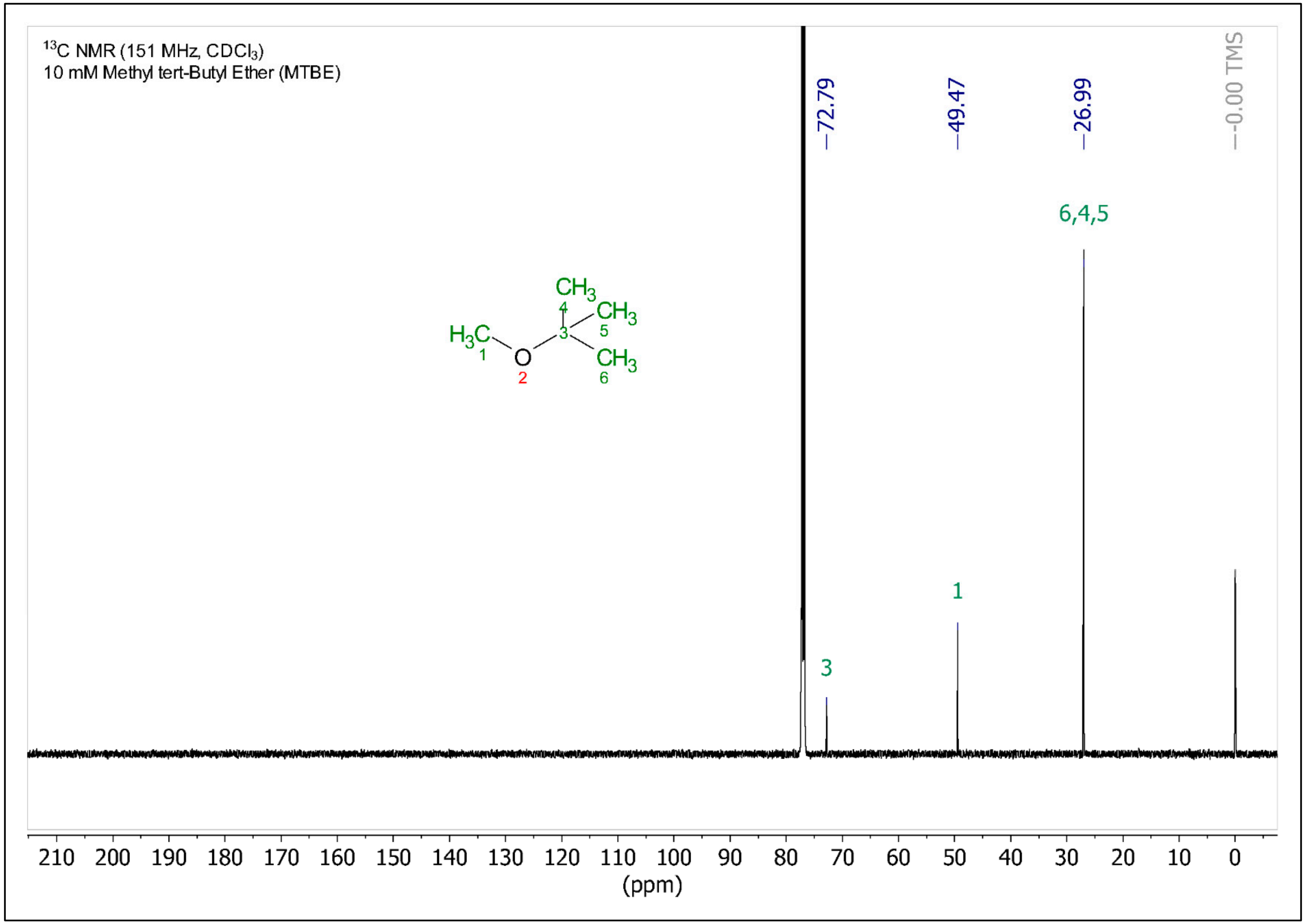

^{13}C NMR (151 MHz, $CDCl_3$)
10 mM Methyl tert-Butyl Ether (MTBE)
72.79
49.47
26.99
-0.00 TMS
6,4,5
1
3
H_3C
O
CH_3
CH_3
CH_3
210 200 190 180 170 160 150 140 130 120 110 100 90 80 70 60 50 40 30 20 10 0
(ppm)

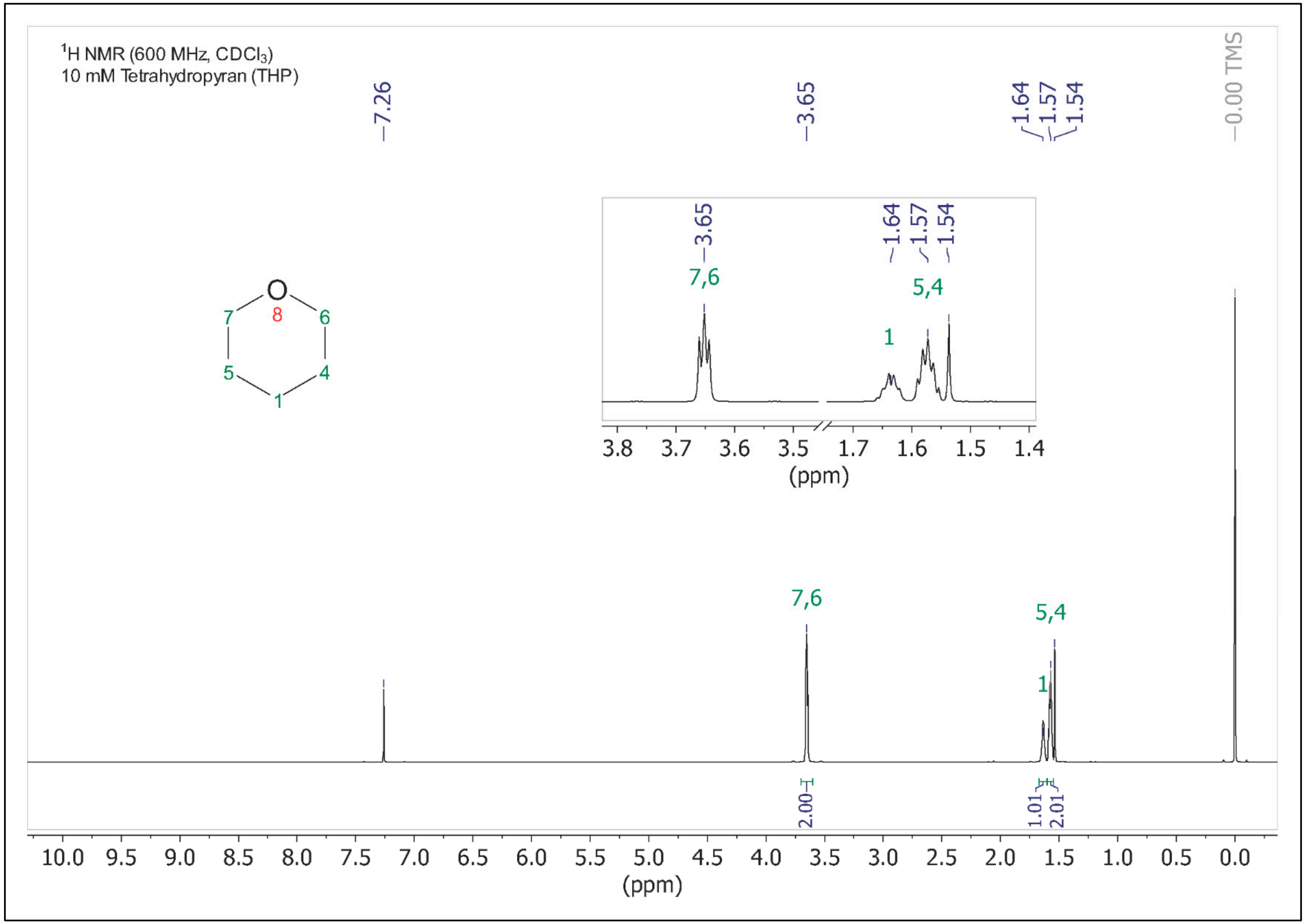

1H NMR (600 MHz, CDCl3)
10 mM Tetrahydropyran (THP)
7.26
3.65
1.64
1.57
1.54
0.00 TMS
7,6
5,4
1
2.00
1.01
2.01
(ppm)

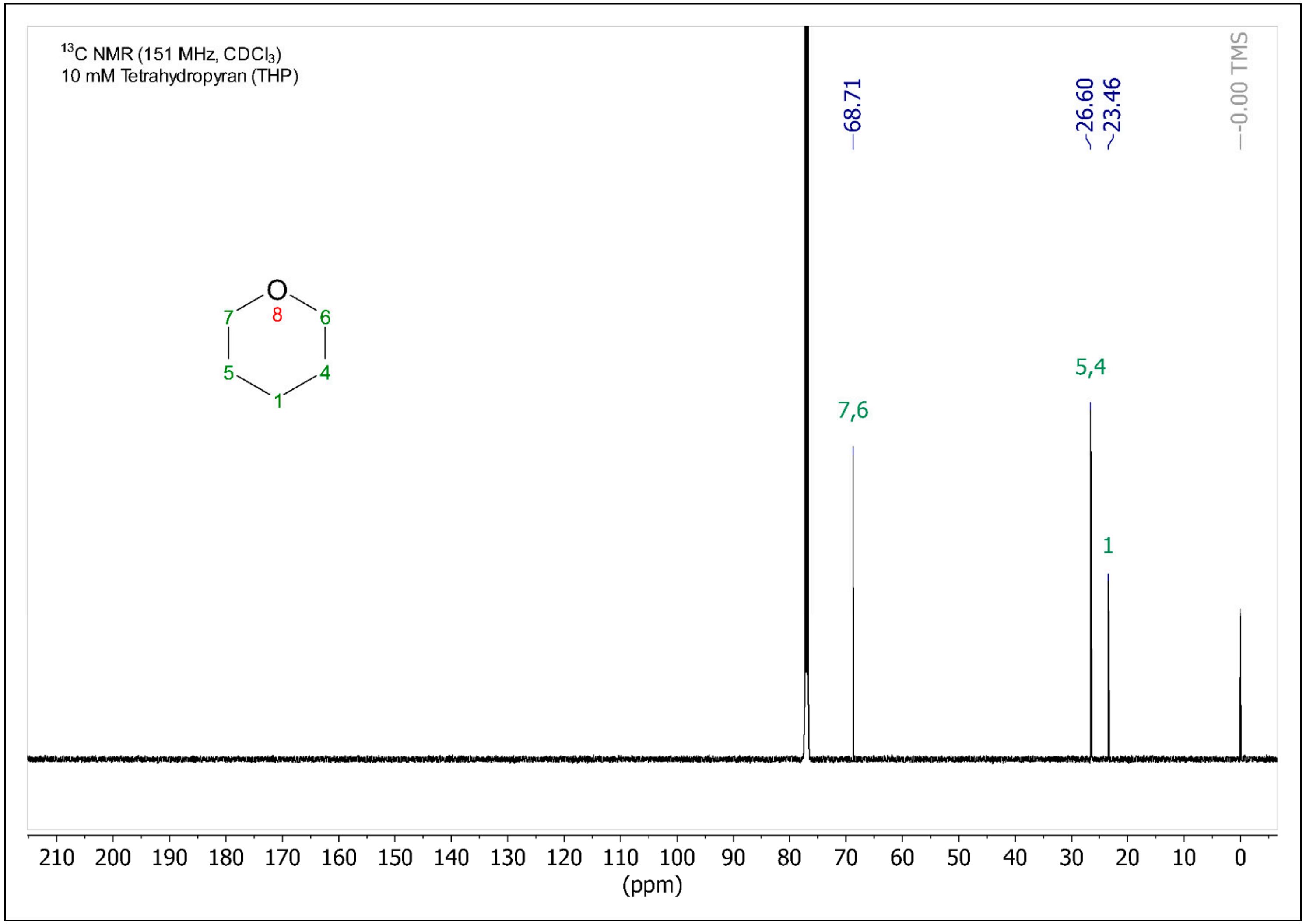
13C NMR (151 MHz, CDCl3)
10 mM Tetrahydropyran (THP)
O
7
8
6
5
4
1
68.71
26.60
23.46
-0.00 TMS
7,6
5,4
1
210 200 190 180 170 160 150 140 130 120 110 100 90 80 70 60 50 40 30 20 10 0
(ppm)

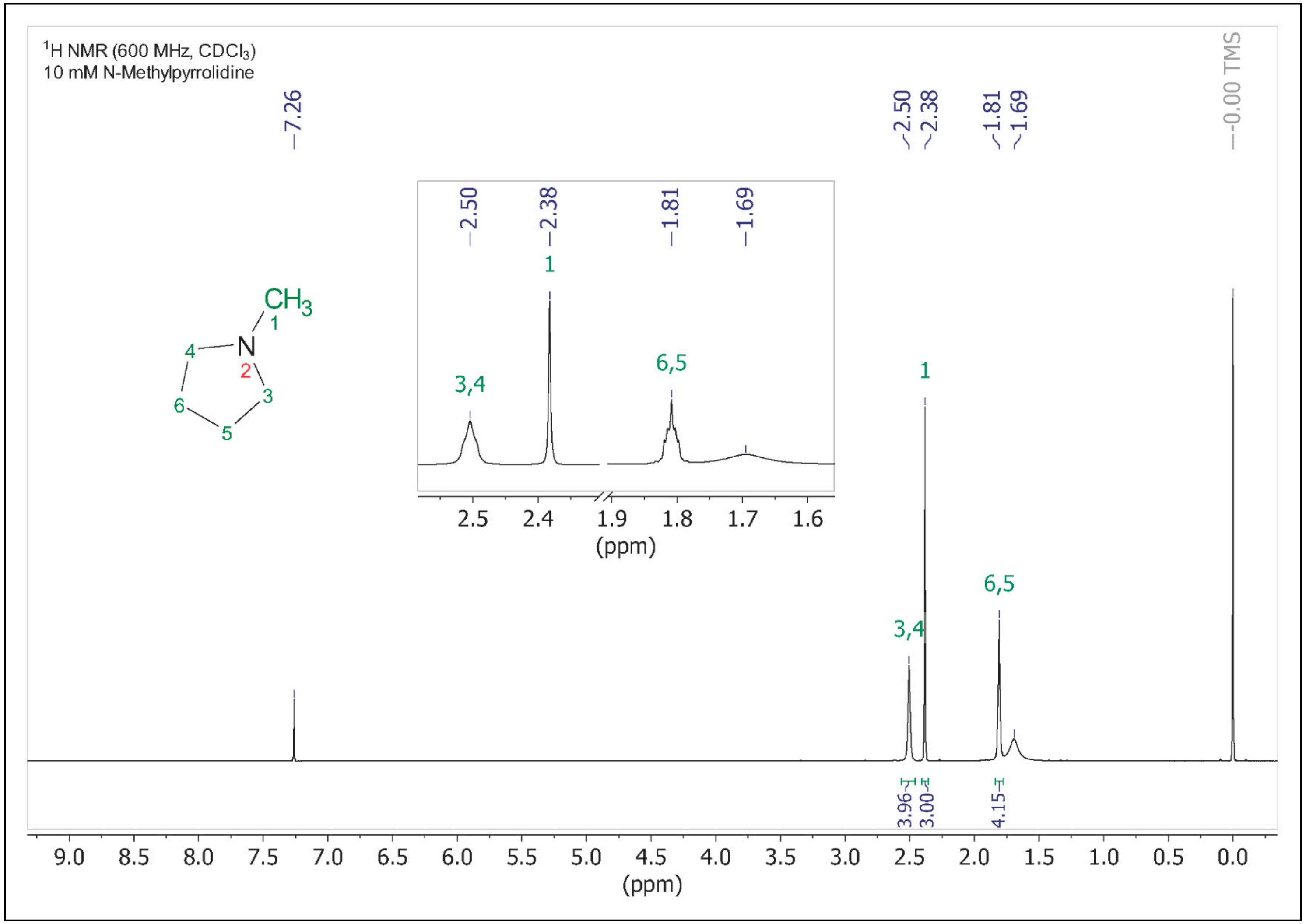

^{1}H NMR (600 MHz, $CDCl_3$)
10 mM N-Methylpyrrolidine
7.26
2.50
2.38
1.81
1.69
0.00 TMS
CH_3
N
1
2
3
4
5
6
3,4
6,5
3.96
3.00
4.15
9.0 8.5 8.0 7.5 7.0 6.5 6.0 5.5 5.0 4.5 4.0 3.5 3.0 2.5 2.0 1.5 1.0 0.5 0.0
(ppm)
2.5 2.4 1.9 1.8 1.7 1.6
(ppm)

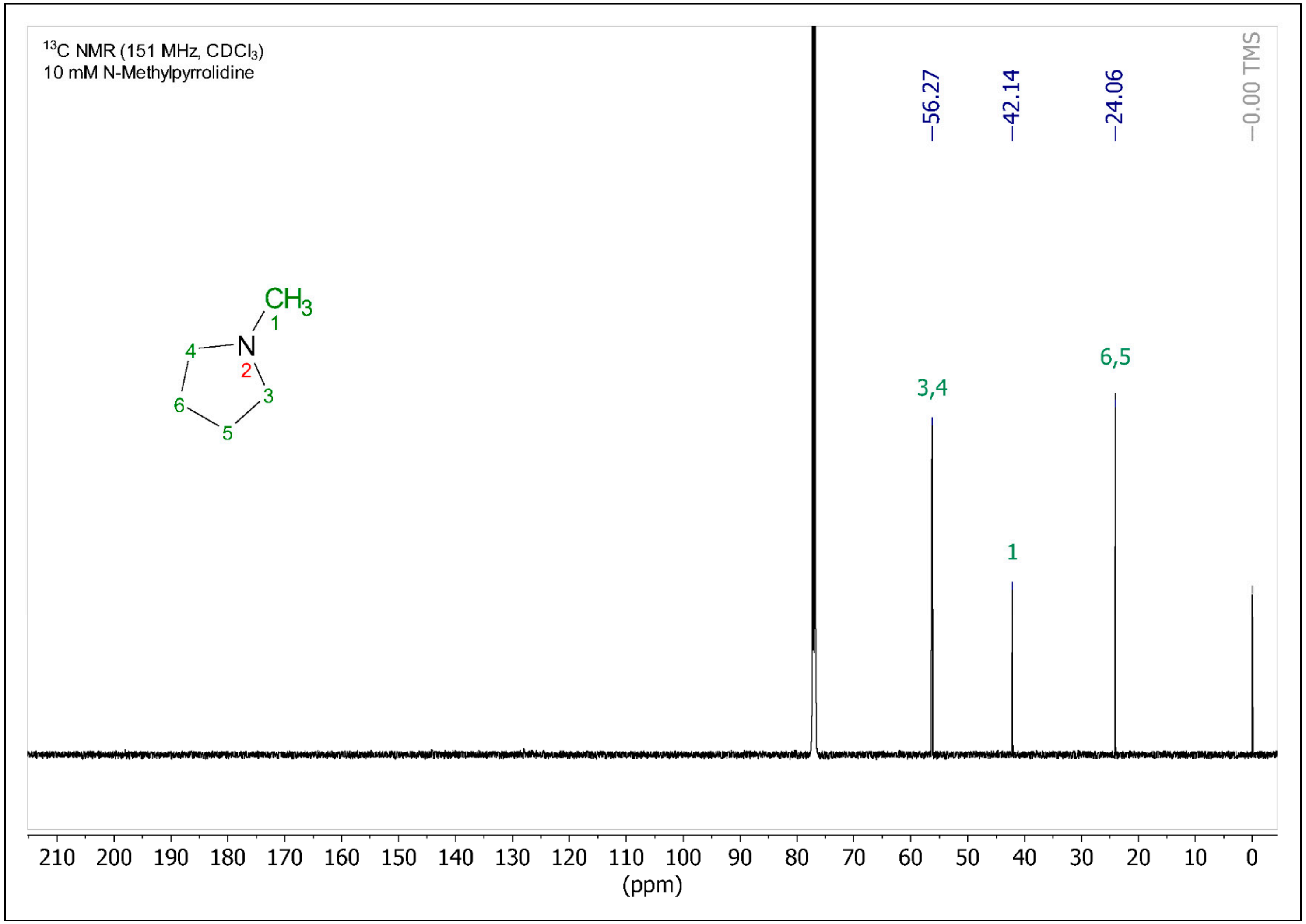

^{13}C NMR (151 MHz, CDCl$_3$)
10 mM N-Methylpyrrolidine
CH3
1
2
N
3
4
5
6
–56.27
–42.14
–24.06
–0.00 TMS
3,4
1
6,5
210 200 190 180 170 160 150 140 130 120 110 100 90 80 70 60 50 40 30 20 10 0
(ppm)

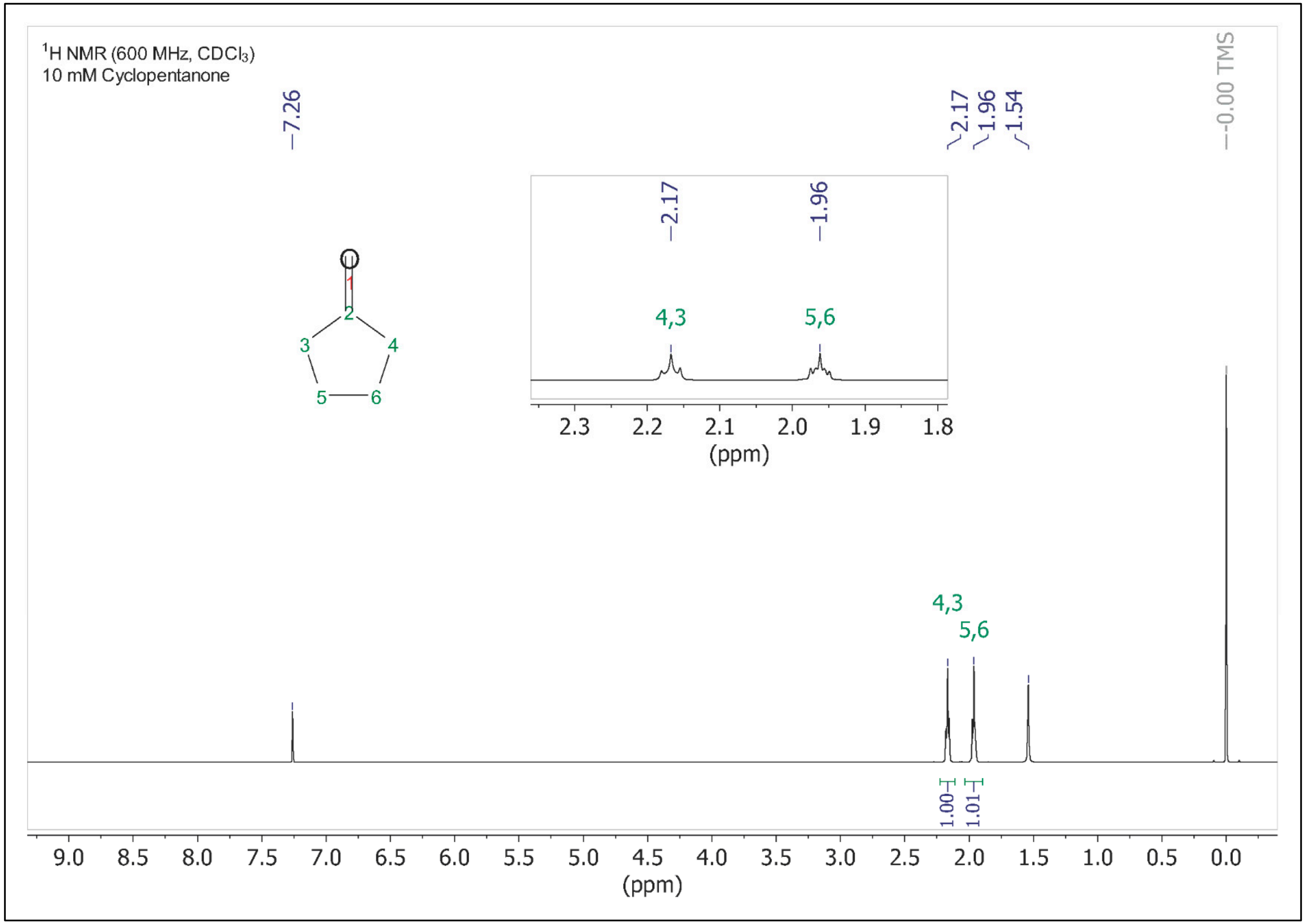

^{1}H NMR (600 MHz, CDCl$_3$)
10 mM Cyclopentanone
7.26
2.17
1.96
1.54
0.00 TMS
1
2
3
4
5
6
4,3
5,6
2.3 2.2 2.1 2.0 1.9 1.8
(ppm)
1.00
1.01
9.0 8.5 8.0 7.5 7.0 6.5 6.0 5.5 5.0 4.5 4.0 3.5 3.0 2.5 2.0 1.5 1.0 0.5 0.0
(ppm)

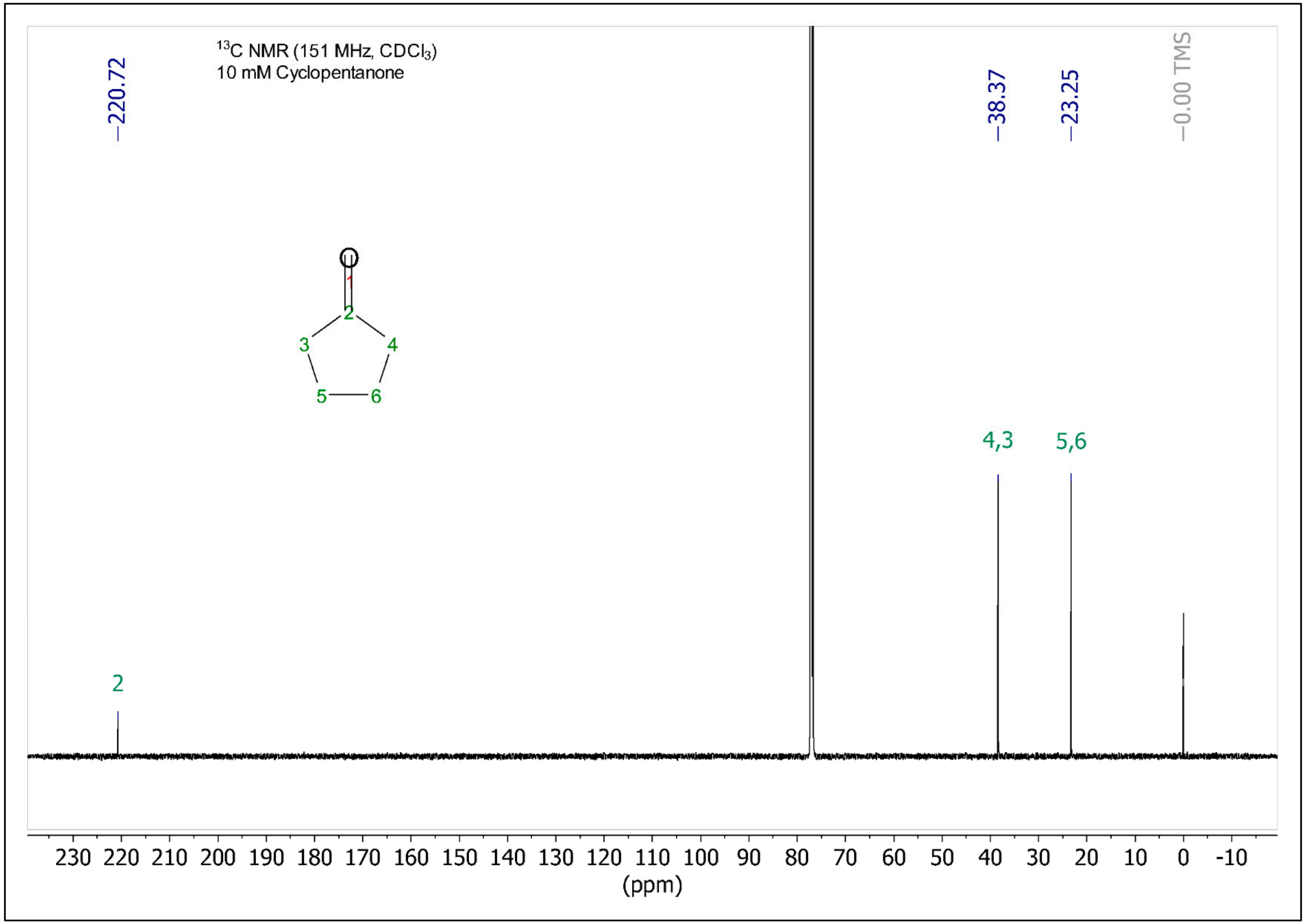

13C NMR (151 MHz, CDCl3)
10 mM Cyclopentanone
220.72
38.37
23.25
0.00 TMS
2
4,3
5,6
3
4
5
6
230 220 210 200 190 180 170 160 150 140 130 120 110 100 90 80 70 60 50 40 30 20 10 0 -10
(ppm)

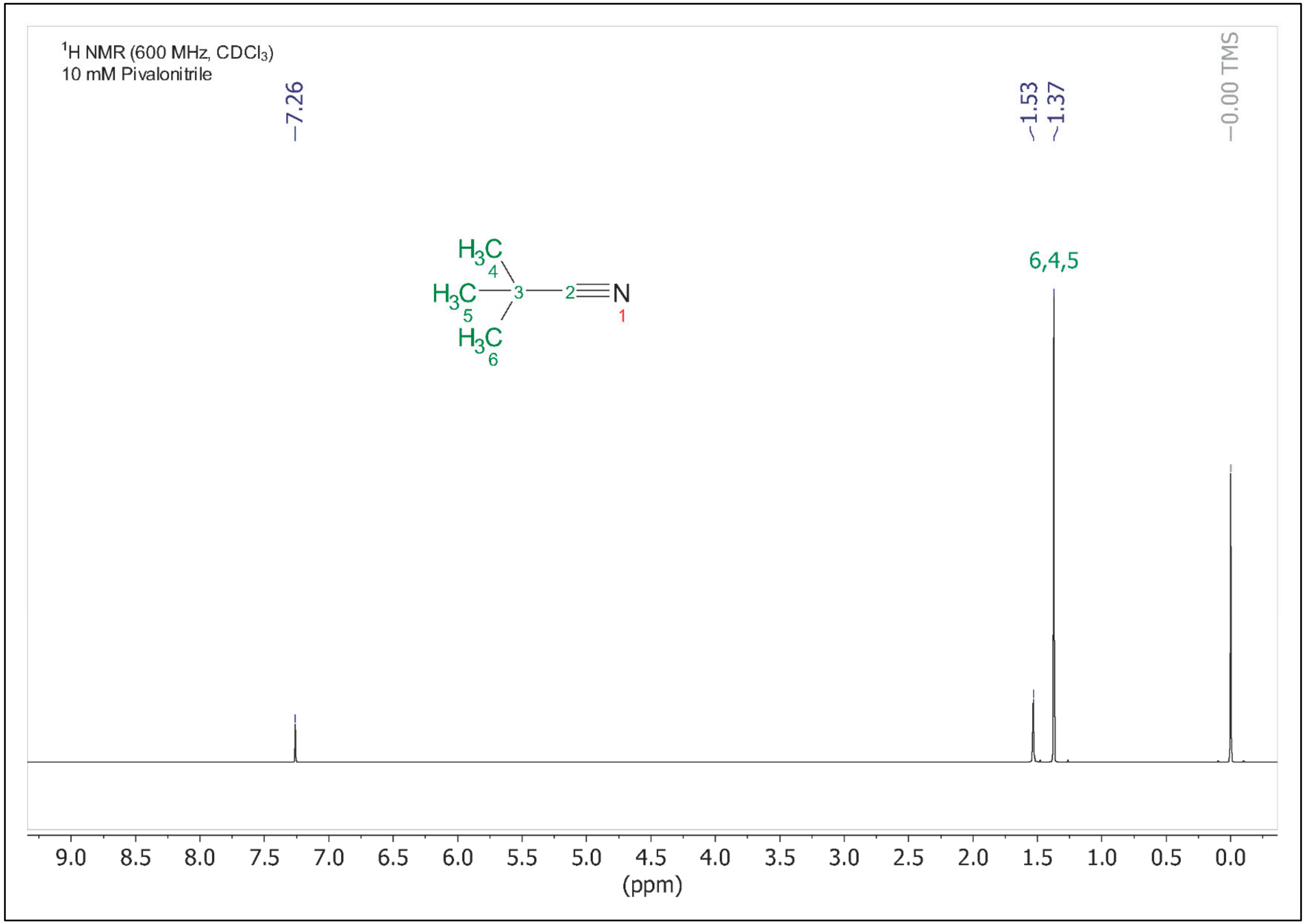

^{1}H NMR (600 MHz, $CDCl_3$)
10 mM Pivalonitrile
7.26
1.53
1.37
0.00 TMS
6,4,5
H_3C
N
9.0 8.5 8.0 7.5 7.0 6.5 6.0 5.5 5.0 4.5 4.0 3.5 3.0 2.5 2.0 1.5 1.0 0.5 0.0
(ppm)

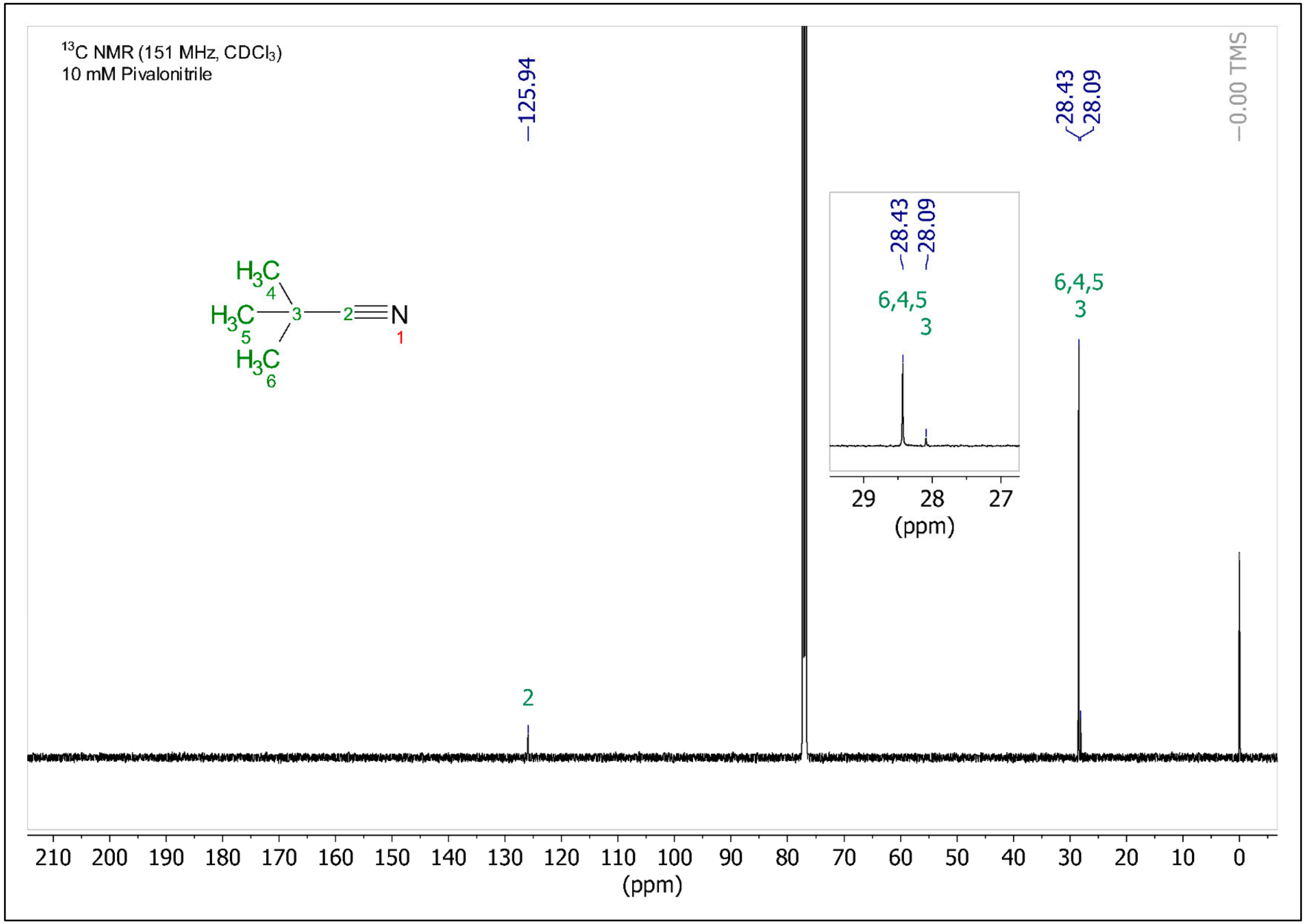

13C NMR (151 MHz, CDCl3)
10 mM Pivalonitrile
H3C 4
H3C 5
H3C 6
3
2
N 1
125.94
28.43
28.09
0.00 TMS
6,4,5
3
2
29
28
27
(ppm)
210 200 190 180 170 160 150 140 130 120 110 100 90 80 70 60 50 40 30 20 10 0
(ppm)

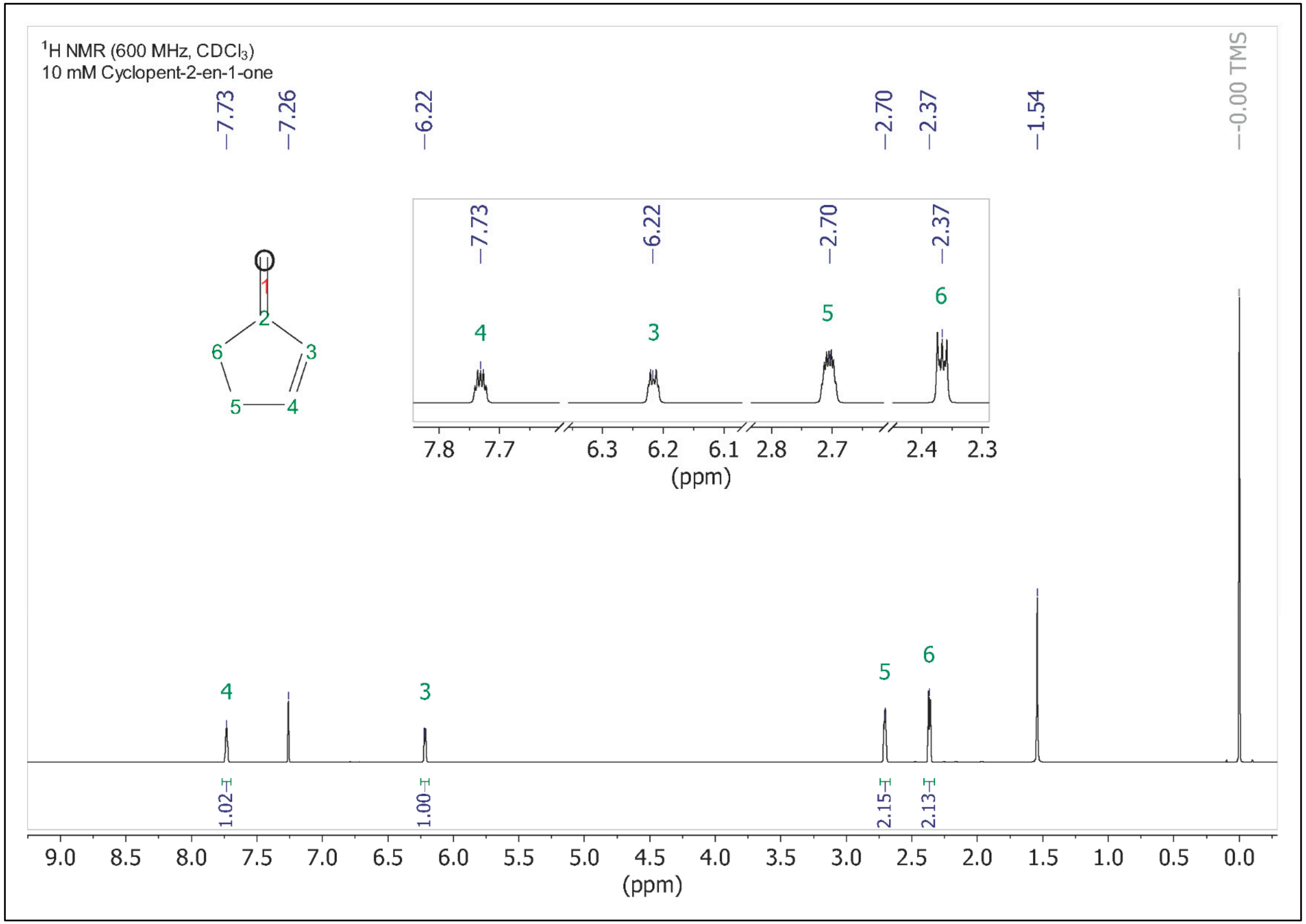

^{1}H NMR (600 MHz, CDCl$_3$)
10 mM Cyclopent-2-en-1-one
7.73
7.26
6.22
2.70
2.37
1.54
0.00 TMS
1.02
1.00
2.15
2.13
(ppm)

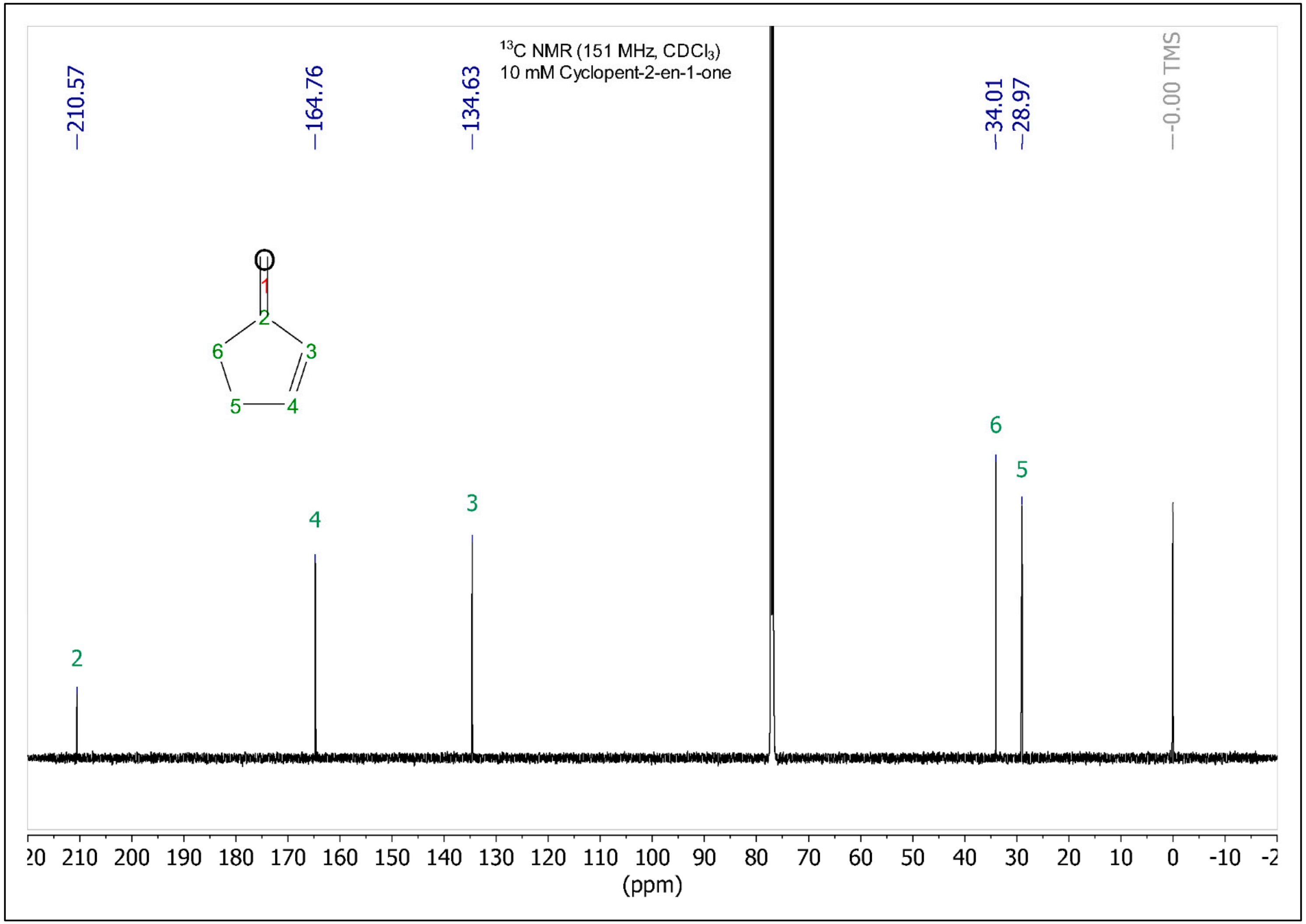
13C NMR (151 MHz, CDCl3)
10 mM Cyclopent-2-en-1-one
210.57
164.76
134.63
34.01
28.97
-0.00 TMS
O
1
2
3
4
5
6
20 210 200 190 180 170 160 150 140 130 120 110 100 90 80 70 60 50 40 30 20 10 0 -10 -2
(ppm)

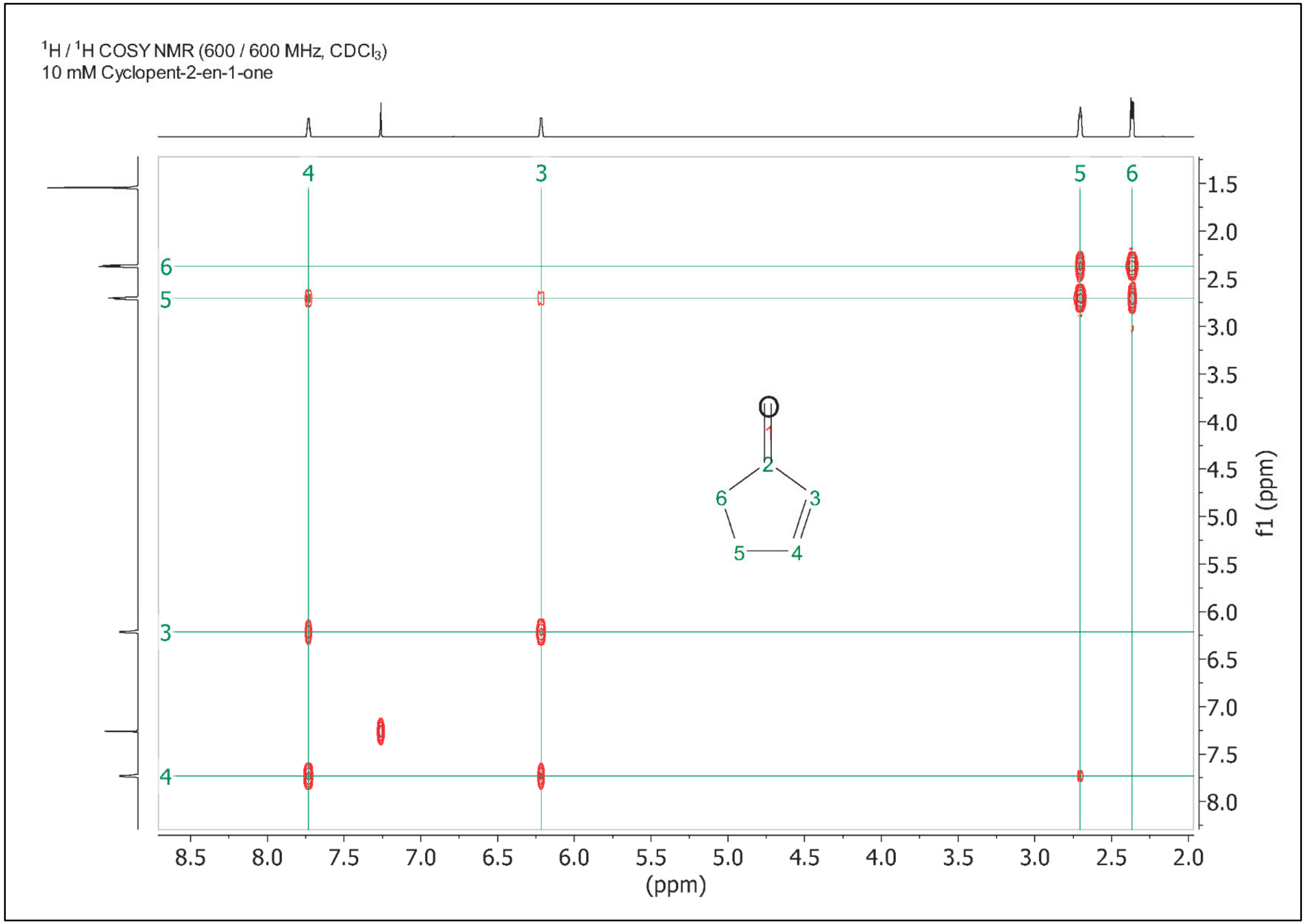

^1H / ^1H COSY NMR (600 / 600 MHz, $CDCl_3$)
10 mM Cyclopent-2-en-1-one
4
3
5
6
O
2
6
3
5
4
(ppm)
f1 (ppm)

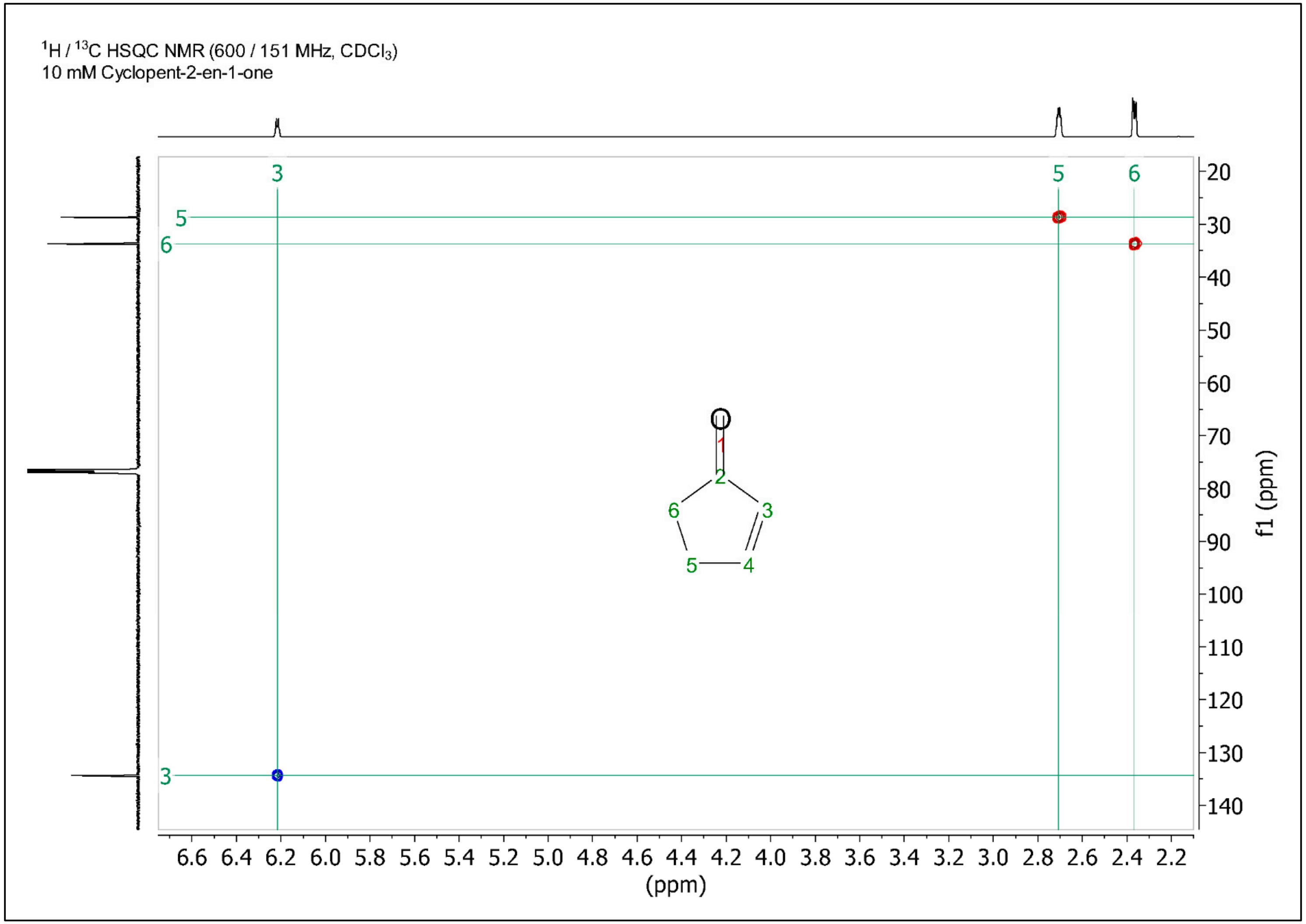

^1H / ^{13}C HSQC NMR (600 / 151 MHz, $CDCl_3$)
10 mM Cyclopent-2-en-1-one
3
5
6
O
1
2
3
4
5
6
20
30
40
50
60
70
80
90
100
110
120
130
140
f1 (ppm)
6.6 6.4 6.2 6.0 5.8 5.6 5.4 5.2 5.0 4.8 4.6 4.4 4.2 4.0 3.8 3.6 3.4 3.2 3.0 2.8 2.6 2.4 2.2
(ppm)

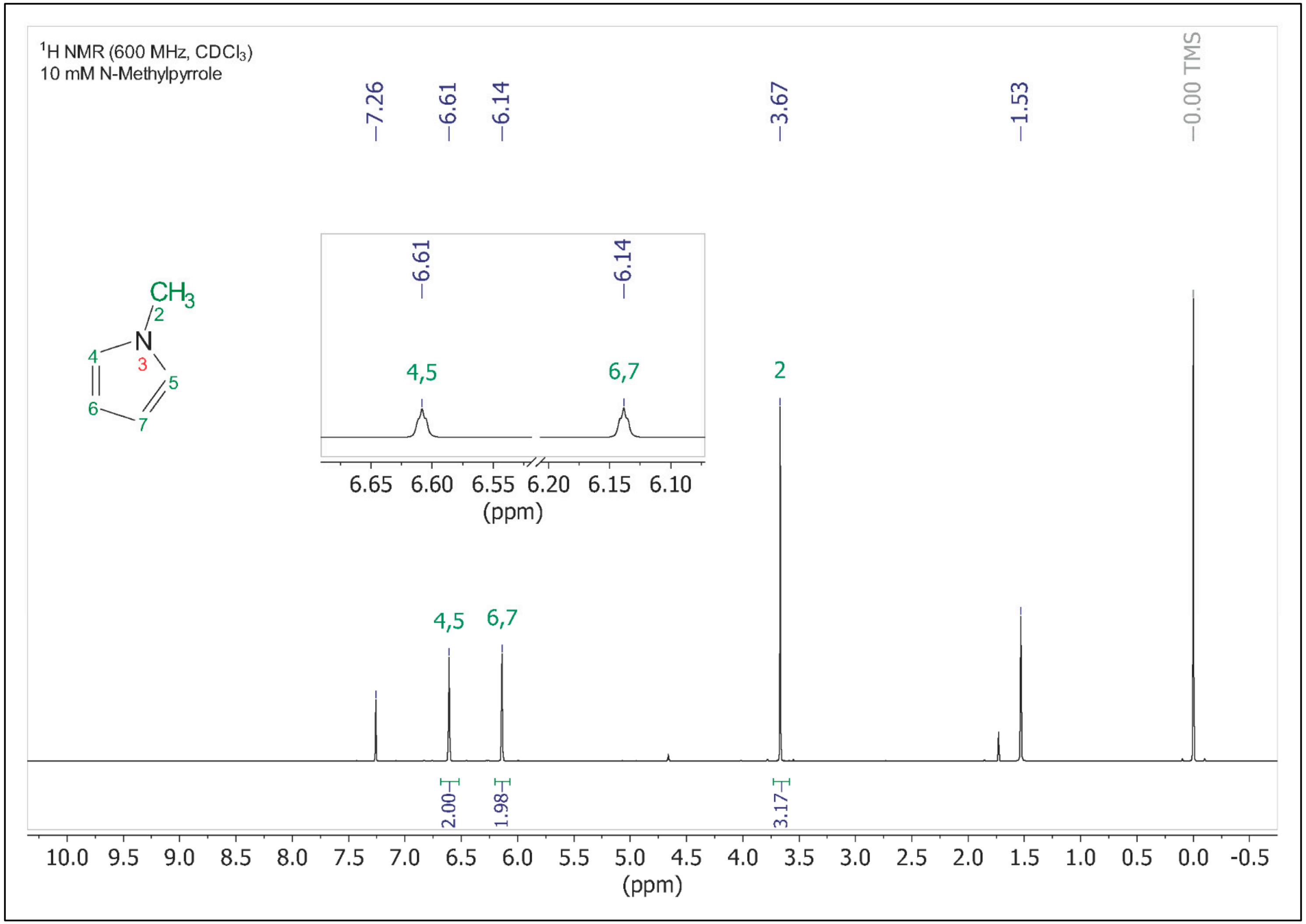

1H NMR (600 MHz, CDCl3)
10 mM N-Methylpyrrole
7.26
6.61
6.14
3.67
1.53
0.00 TMS
CH3
2
N
3
4
5
6
7
4,5
6,7
2
6.65 6.60 6.55 6.20 6.15 6.10
(ppm)
2.00
1.98
3.17
10.0 9.5 9.0 8.5 8.0 7.5 7.0 6.5 6.0 5.5 5.0 4.5 4.0 3.5 3.0 2.5 2.0 1.5 1.0 0.5 0.0 -0.5
(ppm)

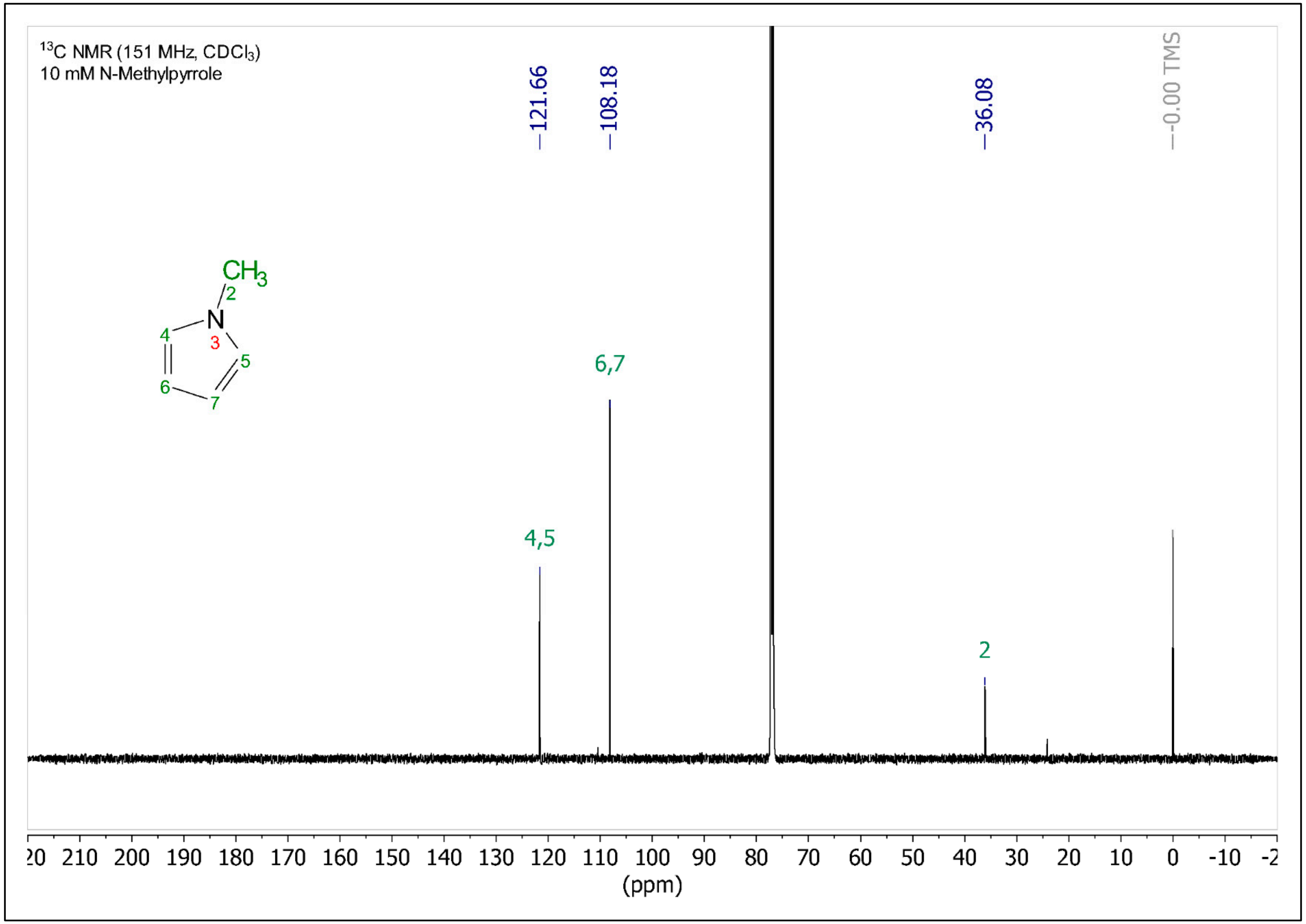

13C NMR (151 MHz, CDCl3)
10 mM N-Methylpyrrole
121.66
108.18
36.08
-0.00 TMS
CH3
2
N
3
4
5
6
7
4,5
6,7
2
20 210 200 190 180 170 160 150 140 130 120 110 100 90 80 70 60 50 40 30 20 10 0 -10 -2
(ppm)

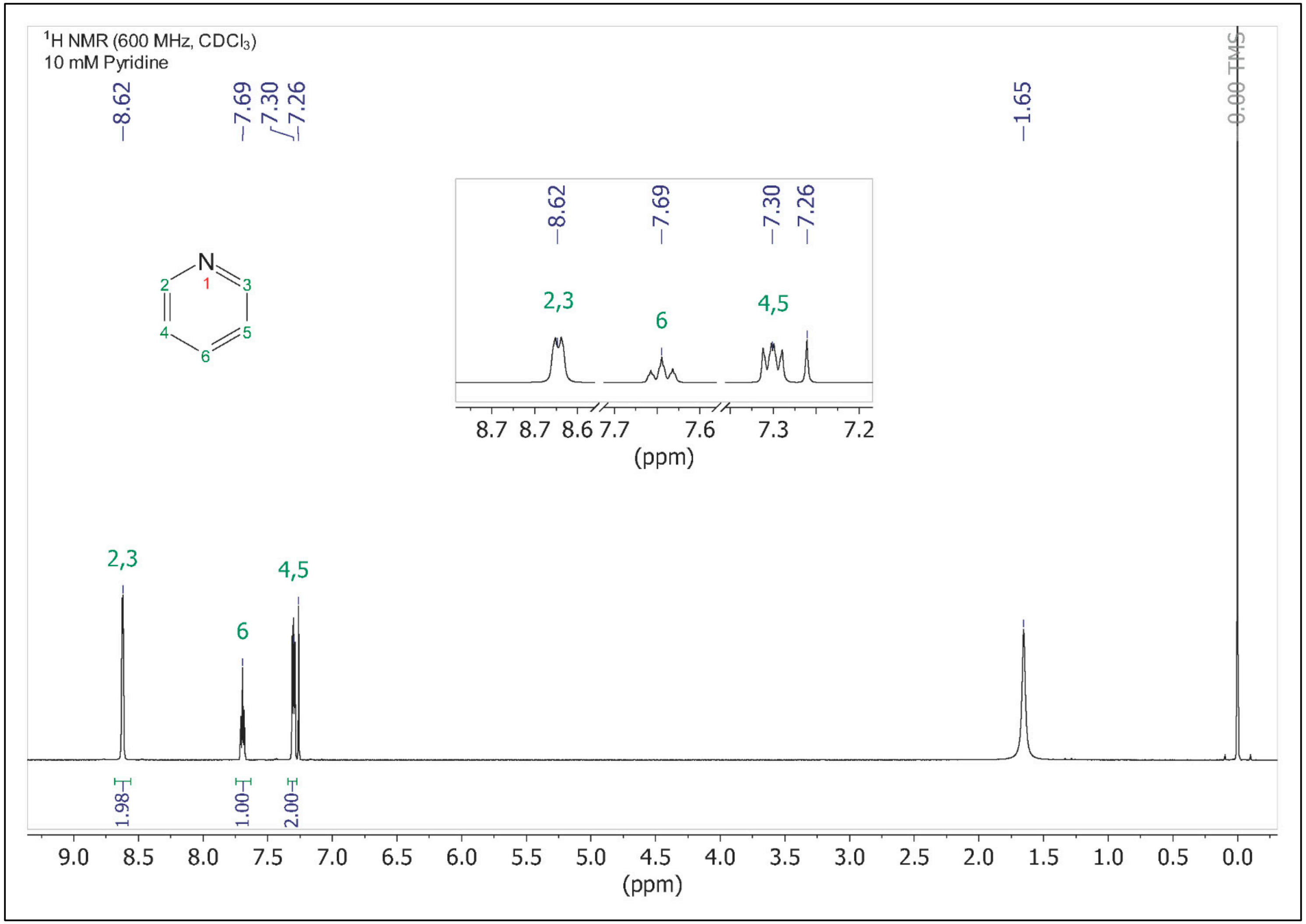

^{1}H NMR (600 MHz, CDCl$_3$)
10 mM Pyridine
8.62
7.69
7.30
7.26
1.65
0.00 TMS
N
1
2
3
4
5
6
2,3
6
4,5
8.7 8.7 8.6 7.7 7.6 7.3 7.2
(ppm)
1.98
1.00
2.00
9.0 8.5 8.0 7.5 7.0 6.5 6.0 5.5 5.0 4.5 4.0 3.5 3.0 2.5 2.0 1.5 1.0 0.5 0.0
(ppm)

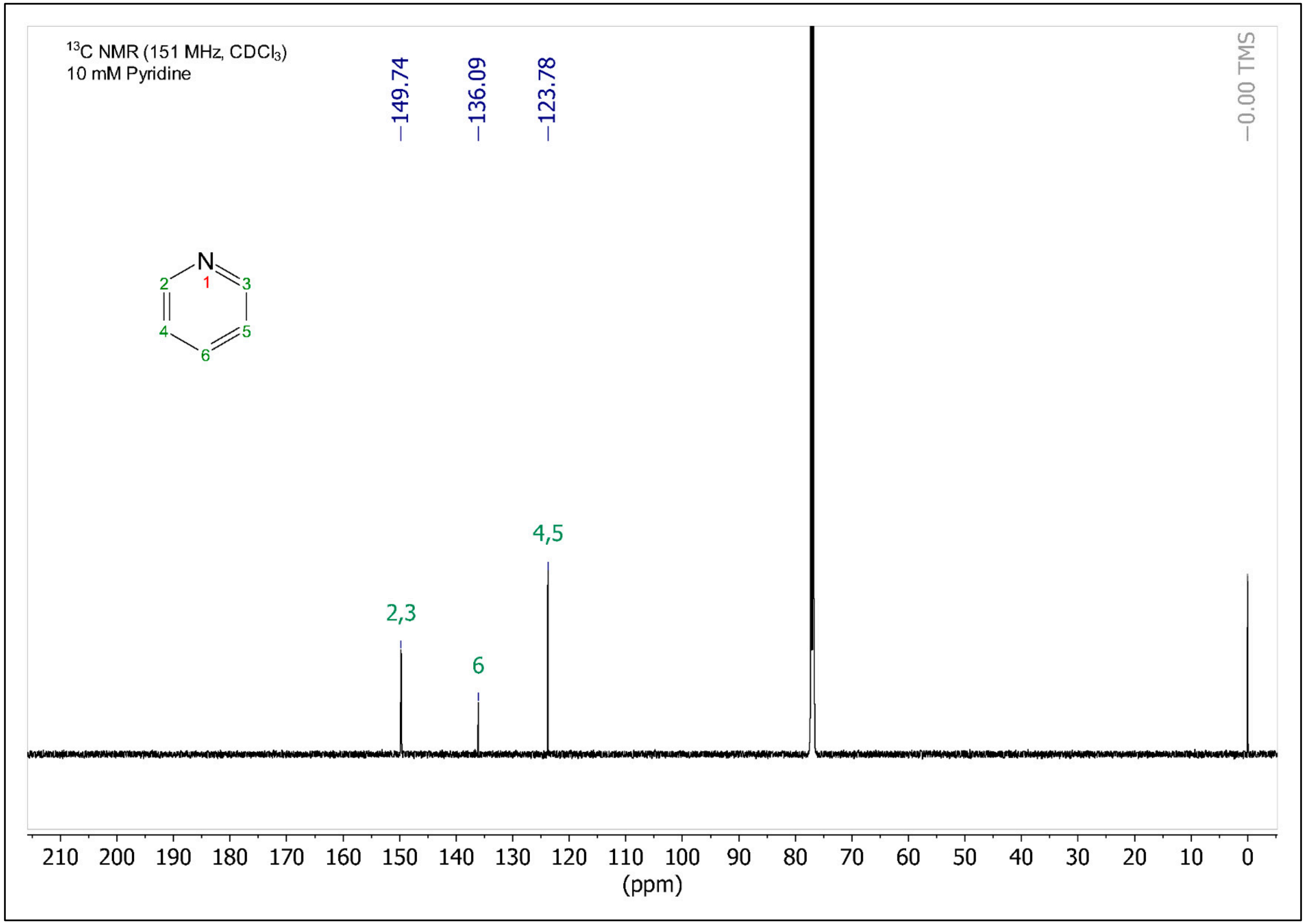

13C NMR (151 MHz, CDCl3)
10 mM Pyridine
149.74
136.09
123.78
0.00 TMS
N
1
2
3
4
5
6
2,3
6
4,5
210 200 190 180 170 160 150 140 130 120 110 100 90 80 70 60 50 40 30 20 10 0
(ppm)

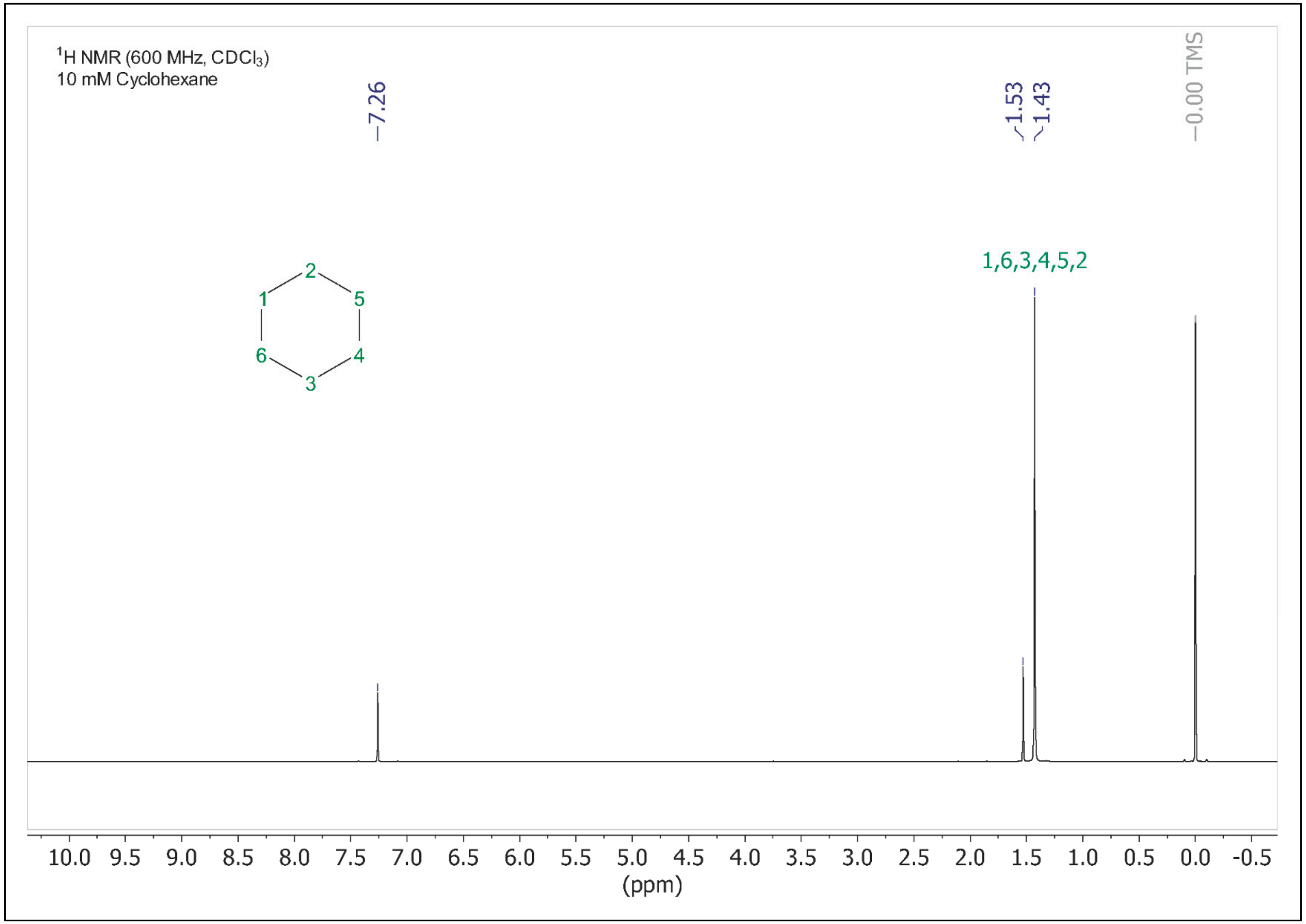

^1H NMR (600 MHz, $CDCl_3$)
10 mM Cyclohexane
7.26
1.53
1.43
0.00 TMS
1,6,3,4,5,2
2
1
5
6
4
3
10.0 9.5 9.0 8.5 8.0 7.5 7.0 6.5 6.0 5.5 5.0 4.5 4.0 3.5 3.0 2.5 2.0 1.5 1.0 0.5 0.0 -0.5
(ppm)

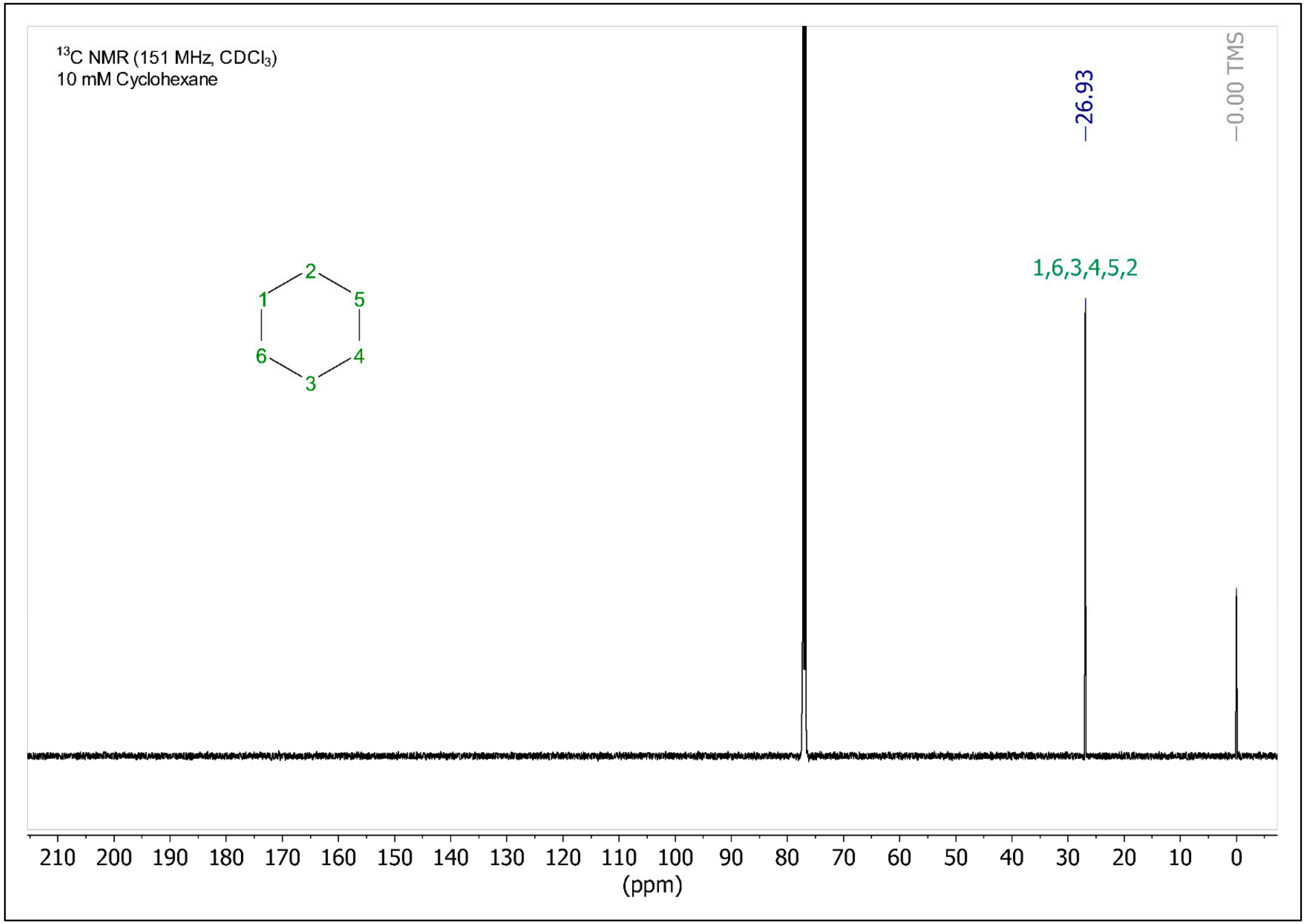

^{13}C NMR (151 MHz, CDCl$_3$)
10 mM Cyclohexane
26.93
0.00 TMS
1,6,3,4,5,2
1
2
5
6
4
3
210 200 190 180 170 160 150 140 130 120 110 100 90 80 70 60 50 40 30 20 10 0
(ppm)

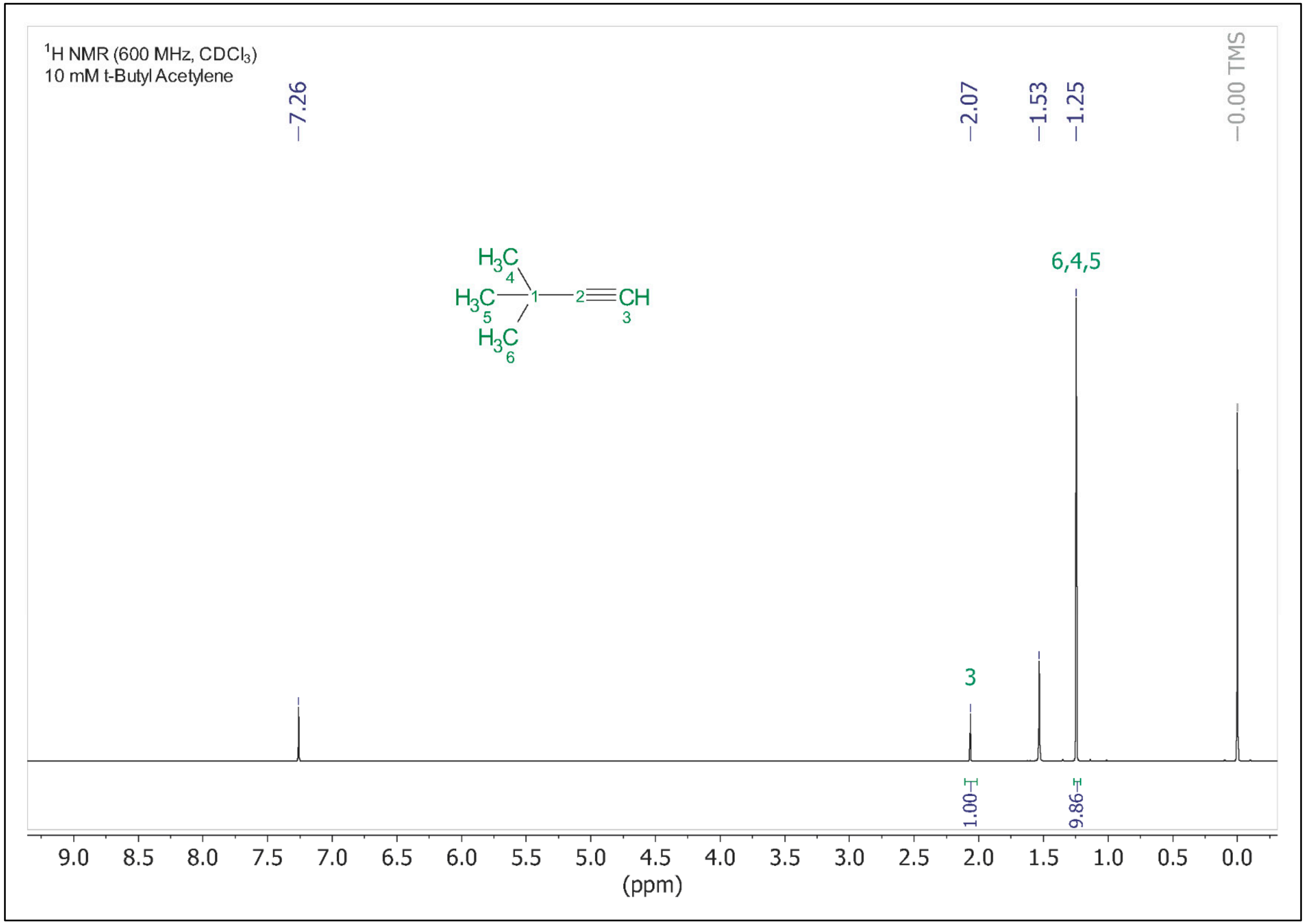

1H NMR (600 MHz, CDCl3)
10 mM t-Butyl Acetylene
7.26
2.07
1.53
1.25
0.00 TMS
H3C 4
H3C 5
H3C 6
1
2
CH 3
6,4,5
3
1.00
9.86
9.0 8.5 8.0 7.5 7.0 6.5 6.0 5.5 5.0 4.5 4.0 3.5 3.0 2.5 2.0 1.5 1.0 0.5 0.0
(ppm)

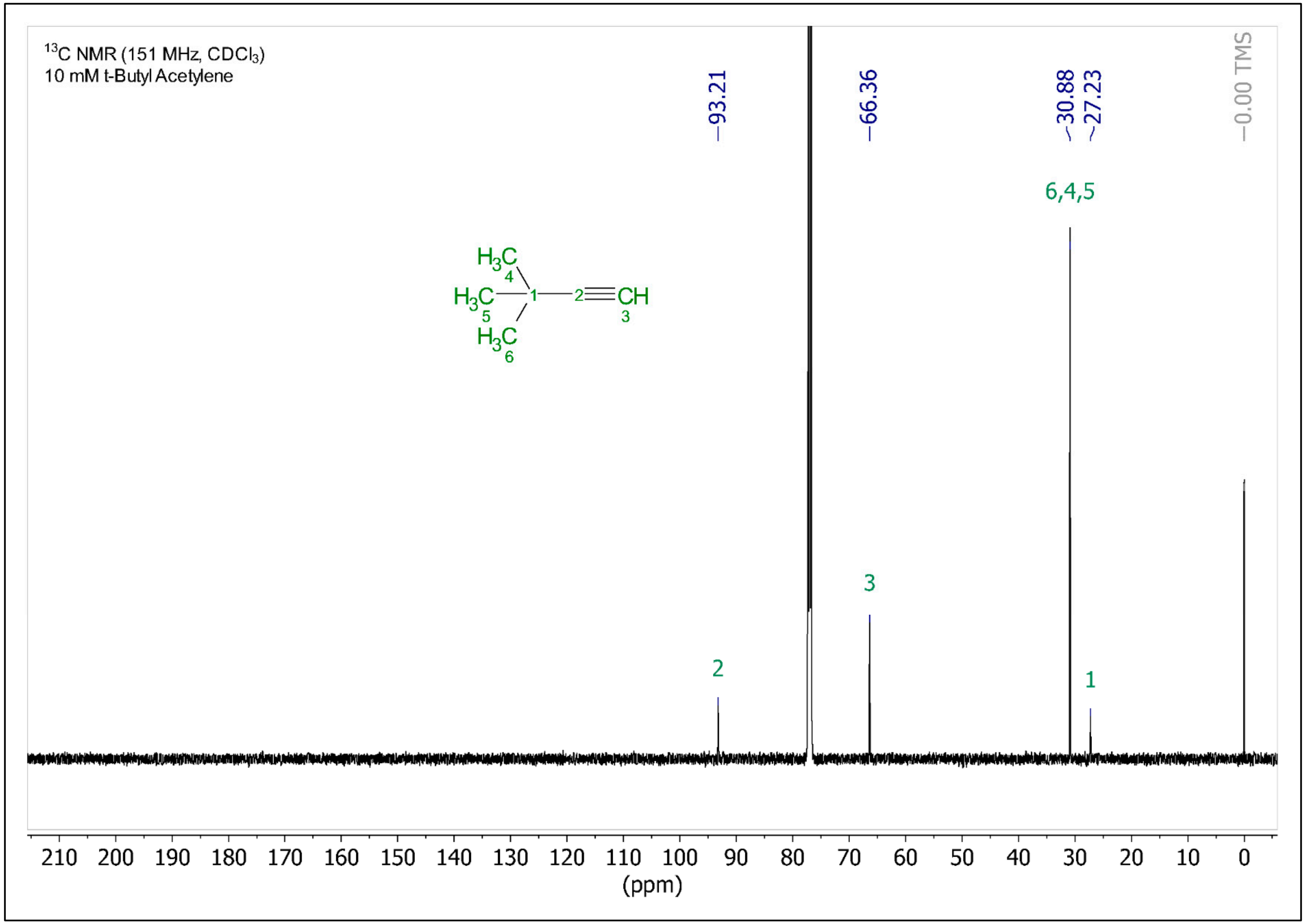

13C NMR (151 MHz, CDCl3)
10 mM t-Butyl Acetylene
93.21
66.36
30.88
27.23
0.00 TMS
6,4,5
3
2
1
H3C
H3C
H3C
CH
210 200 190 180 170 160 150 140 130 120 110 100 90 80 70 60 50 40 30 20 10 0
(ppm)

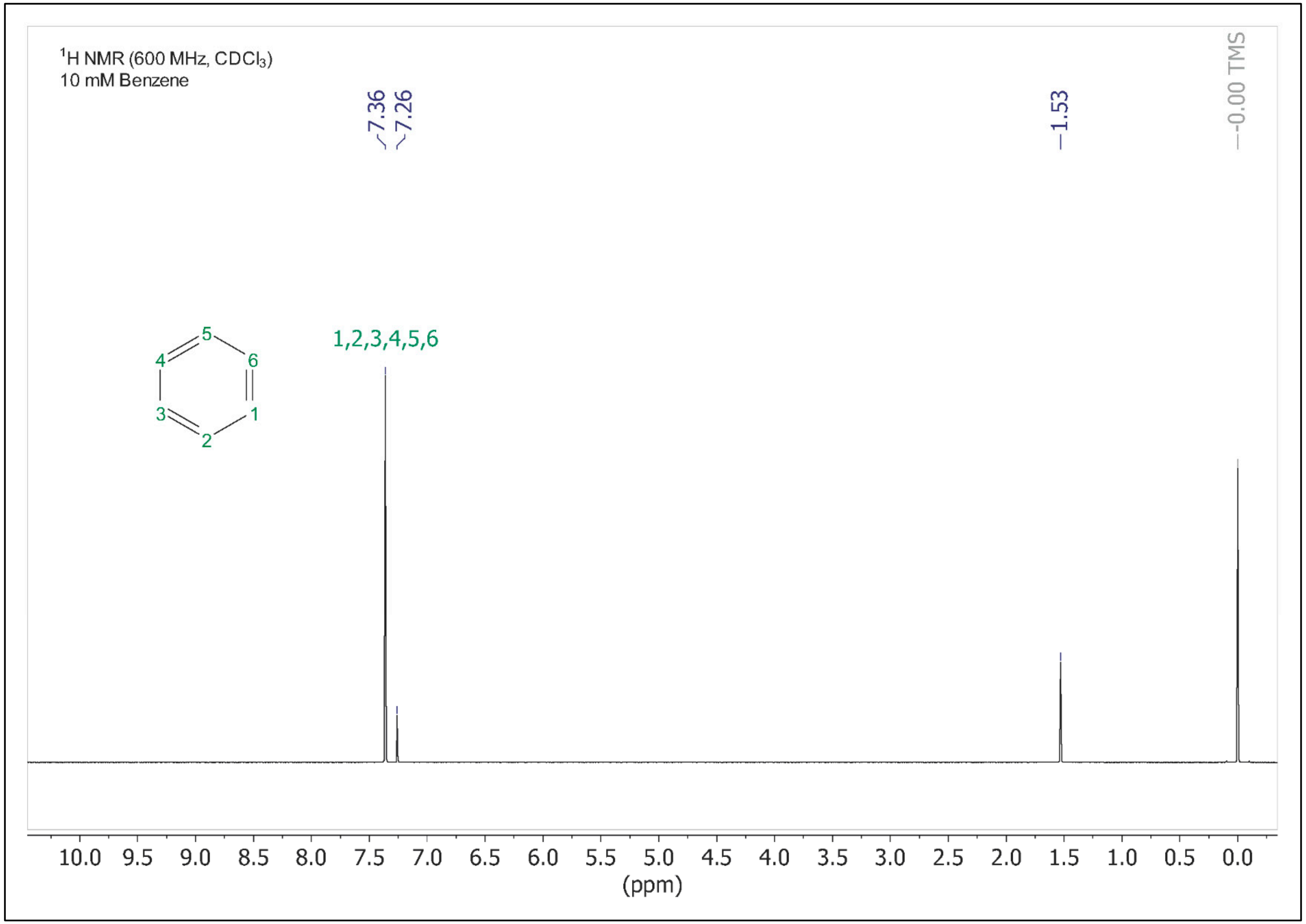

^{1}H NMR (600 MHz, CDCl$_3$)
10 mM Benzene
7.36
7.26
1.53
0.00 TMS
1,2,3,4,5,6
1
2
3
4
5
6
10.0 9.5 9.0 8.5 8.0 7.5 7.0 6.5 6.0 5.5 5.0 4.5 4.0 3.5 3.0 2.5 2.0 1.5 1.0 0.5 0.0
(ppm)

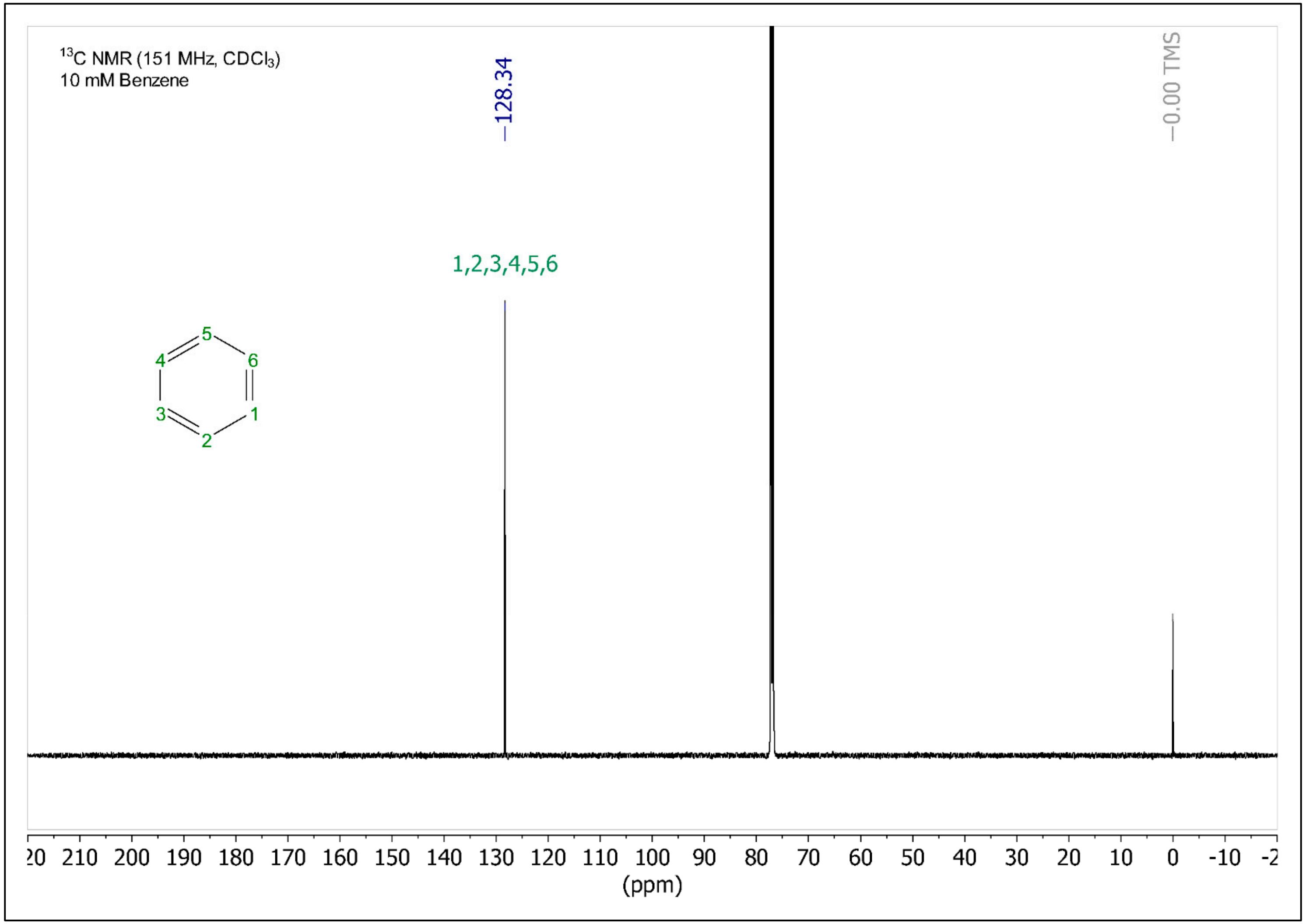

^{13}C NMR (151 MHz, CDCl$_3$)
10 mM Benzene
128.34
0.00 TMS
1,2,3,4,5,6
1
2
3
4
5
6
20 210 200 190 180 170 160 150 140 130 120 110 100 90 80 70 60 50 40 30 20 10 0 -10 -2
(ppm)

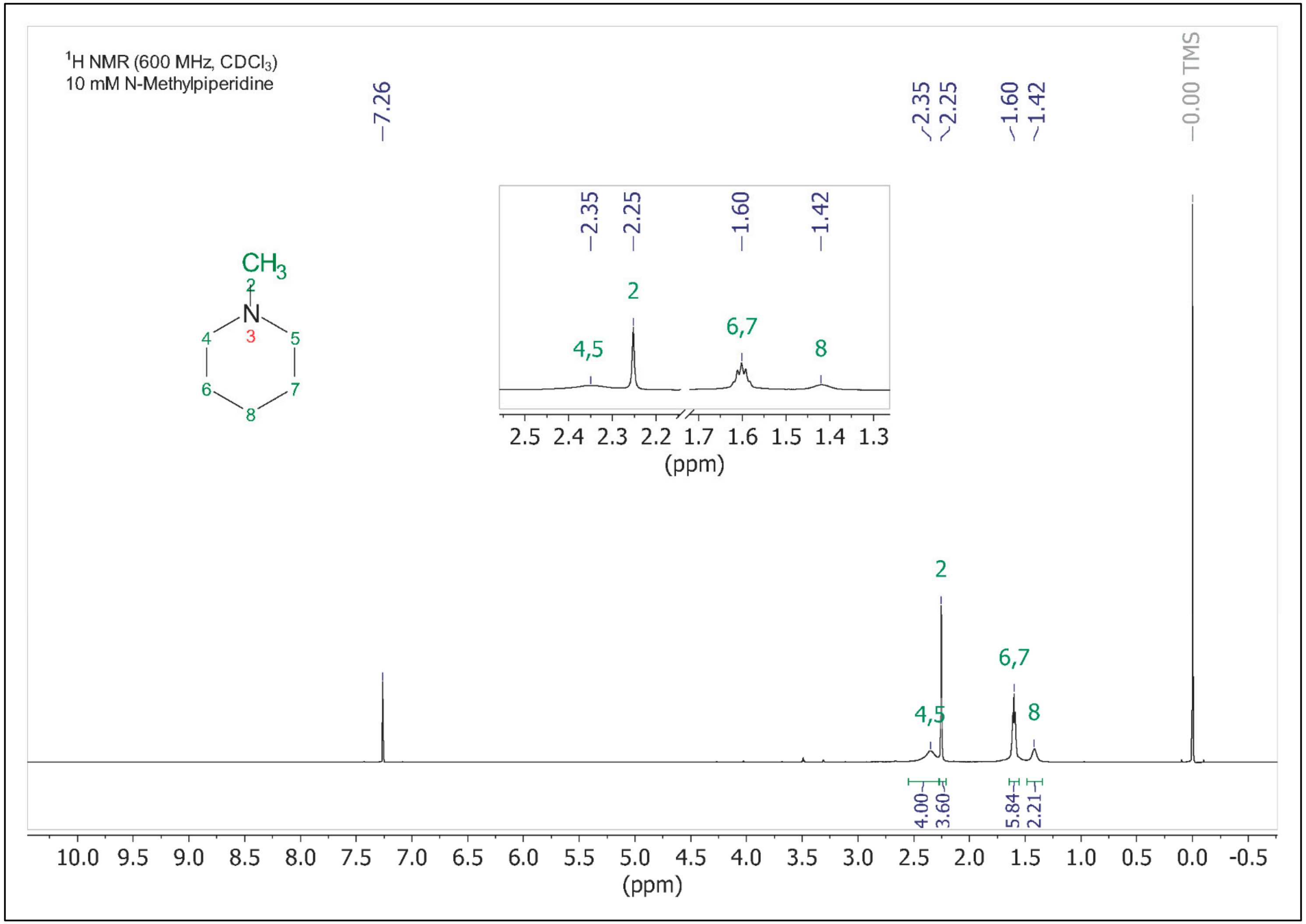

^{1}H NMR (600 MHz, $CDCl_3$)
10 mM N-Methylpiperidine
7.26
2.35
2.25
1.60
1.42
0.00 TMS
CH_3
N
2
3
4
5
6
7
8
4,5
6,7
4.00
3.60
5.84
2.21
(ppm)

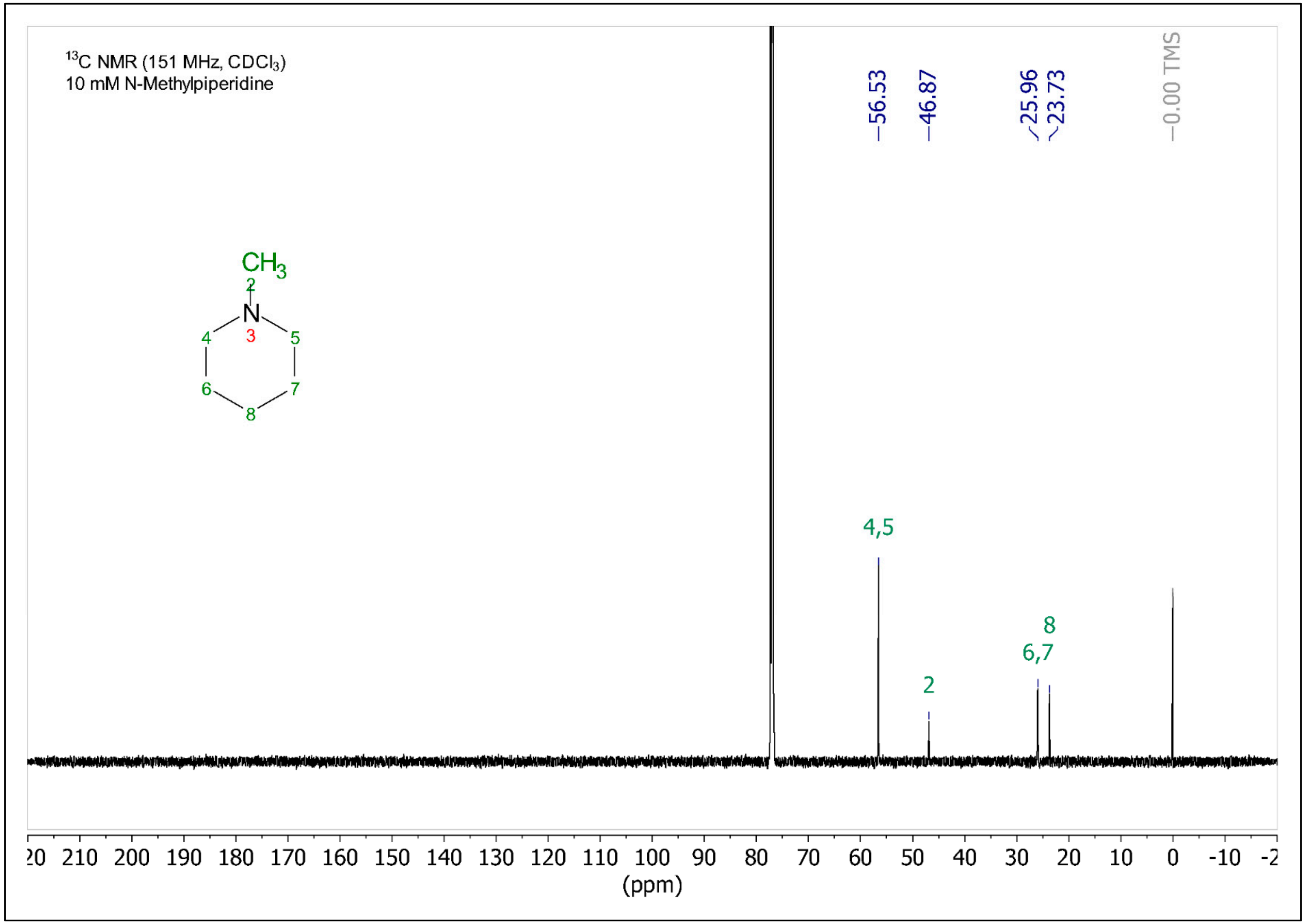

13C NMR (151 MHz, CDCl3)
10 mM N-Methylpiperidine
CH3
N
2
3
4
5
6
7
8
56.53
46.87
25.96
23.73
0.00 TMS
4,5
2
6,7
8
20 210 200 190 180 170 160 150 140 130 120 110 100 90 80 70 60 50 40 30 20 10 0 -10 -2
(ppm)

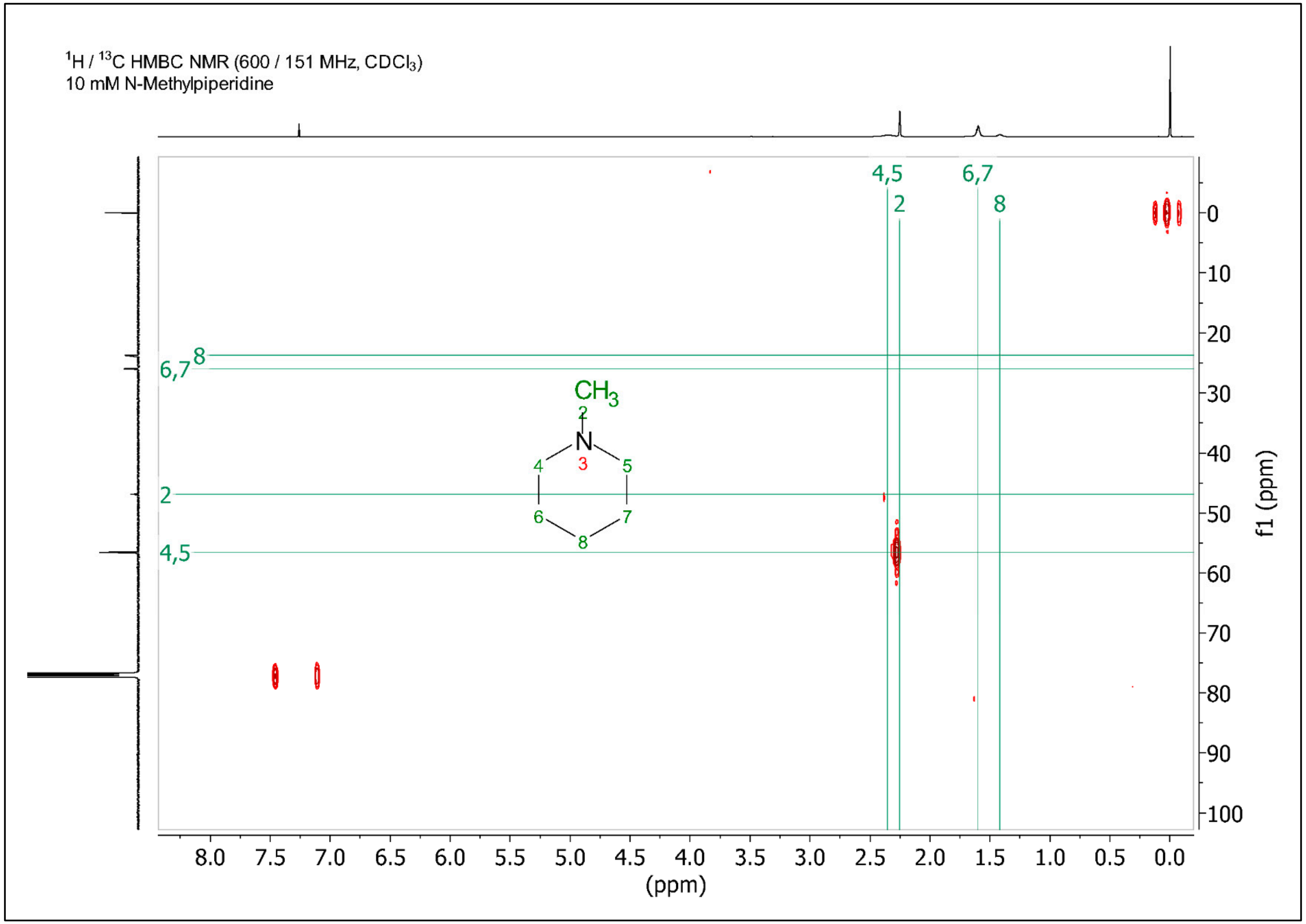

^1H / ^{13}C HMBC NMR (600 / 151 MHz, $CDCl_3$)
10 mM N-Methylpiperidine
4,5
2
6,7
8
6,7
8
2
4,5
CH3
N
f1 (ppm)
(ppm)

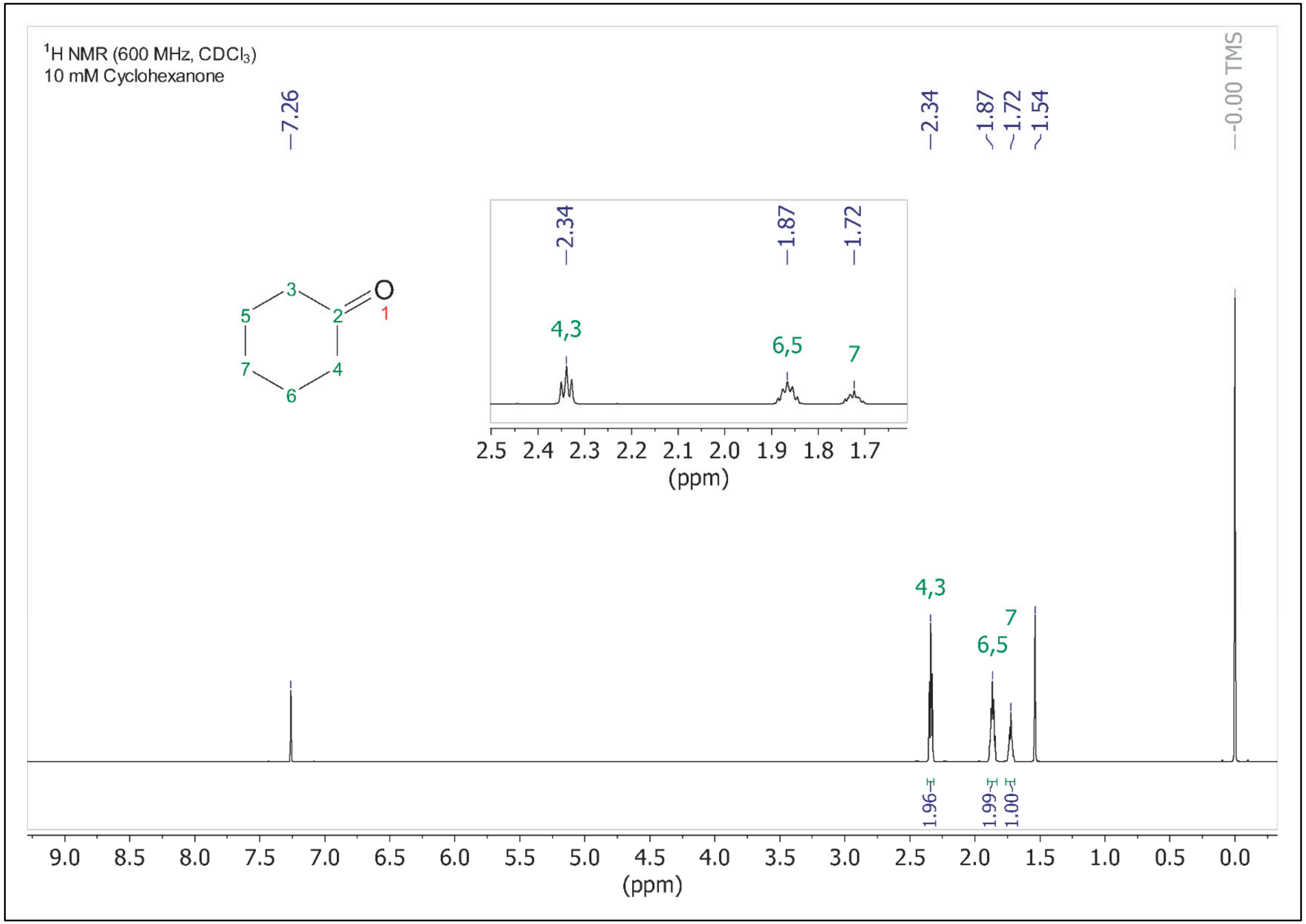

1H NMR (600 MHz, CDCl3)
10 mM Cyclohexanone
7.26
2.34
1.87
1.72
1.54
0.00 TMS
4,3
6,5
7
1.96
1.99
1.00
(ppm)

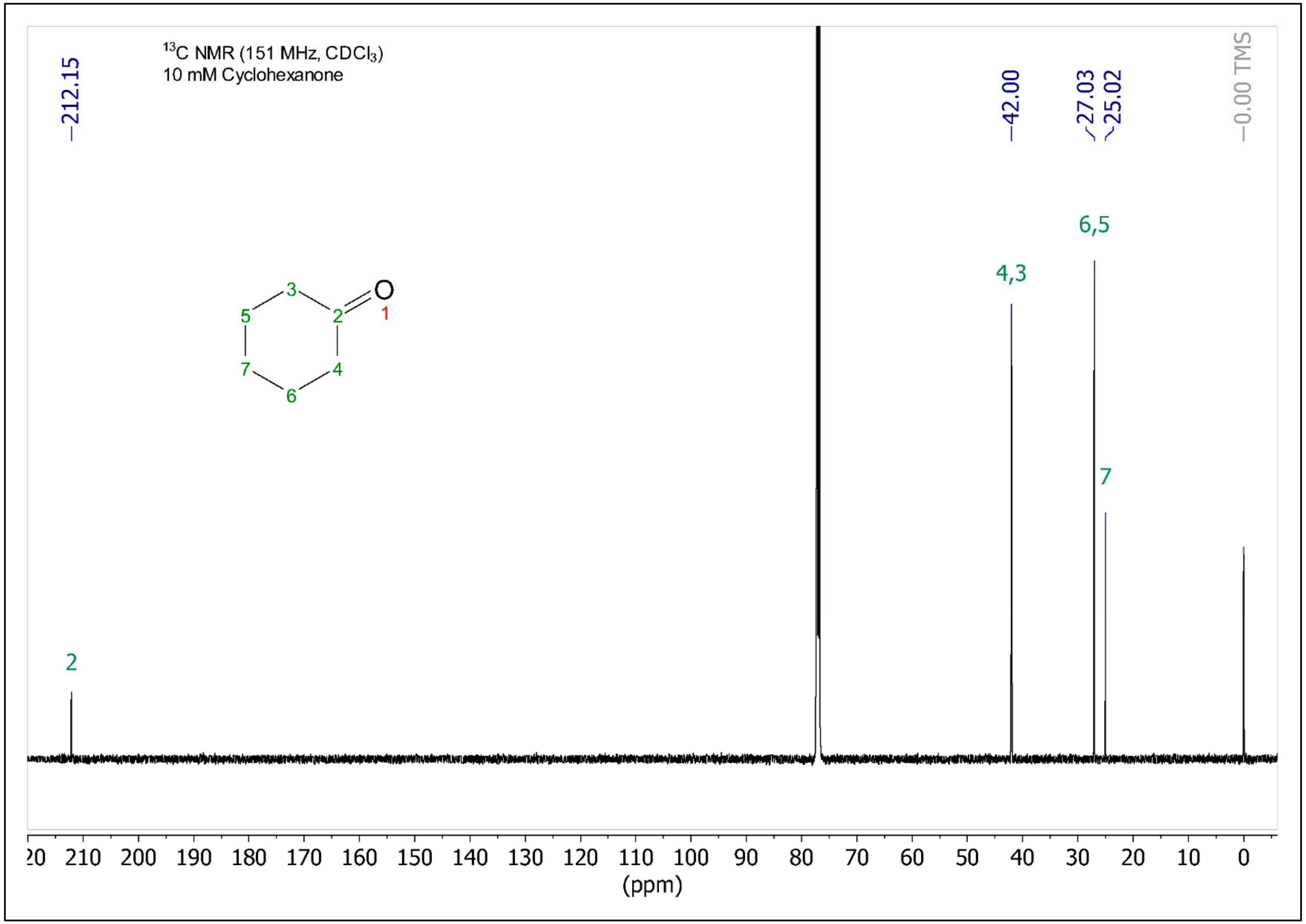

^{13}C NMR (151 MHz, CDCl$_3$)
10 mM Cyclohexanone
212.15
42.00
27.03
25.02
0.00 TMS
2
4,3
6,5
7
1
3
5
6
(ppm)

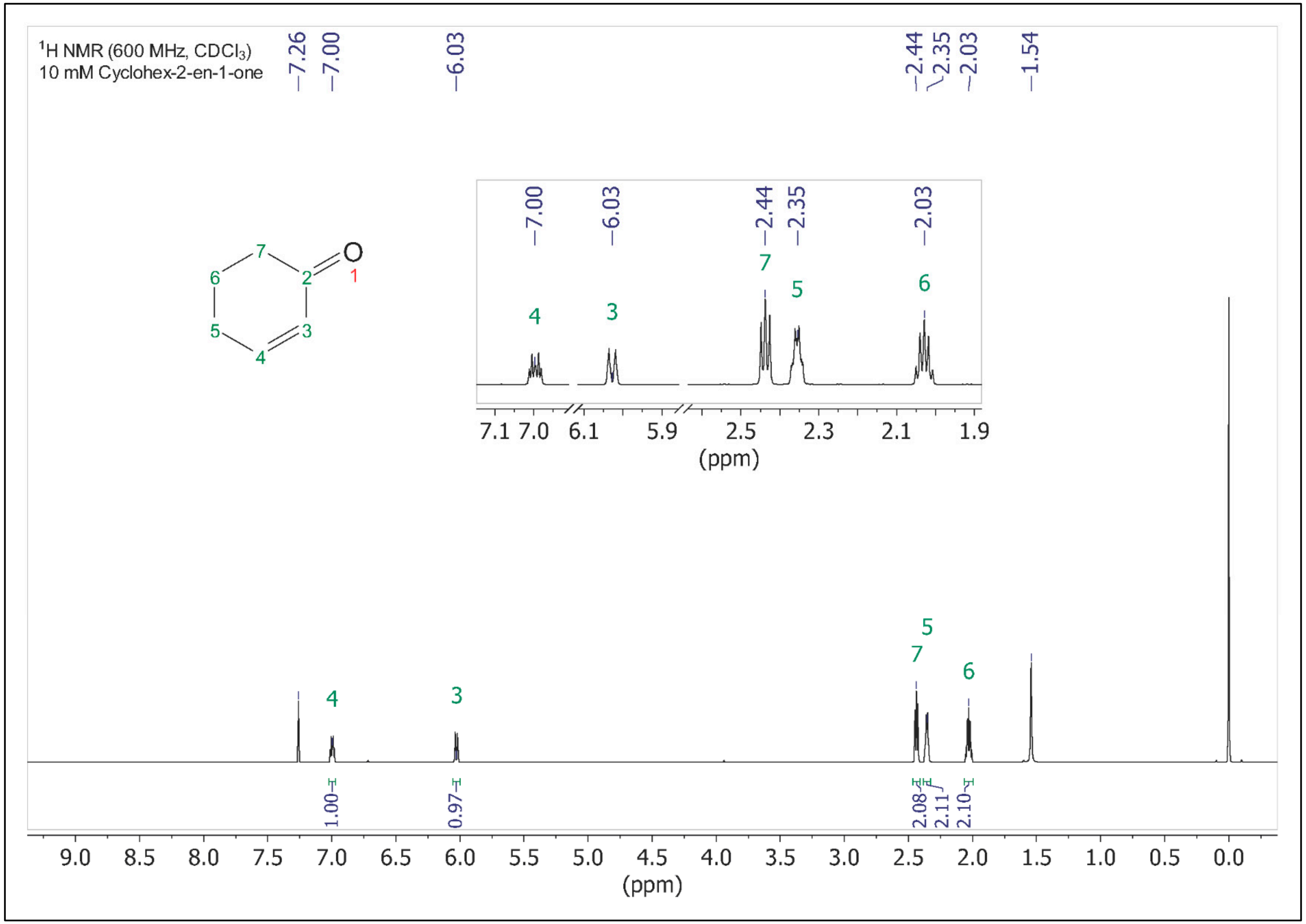

^{1}H NMR (600 MHz, CDCl$_3$)
10 mM Cyclohex-2-en-1-one
7.26
7.00
6.03
2.44
2.35
2.03
1.54
1.00
0.97
2.08
2.11
2.10
(ppm)

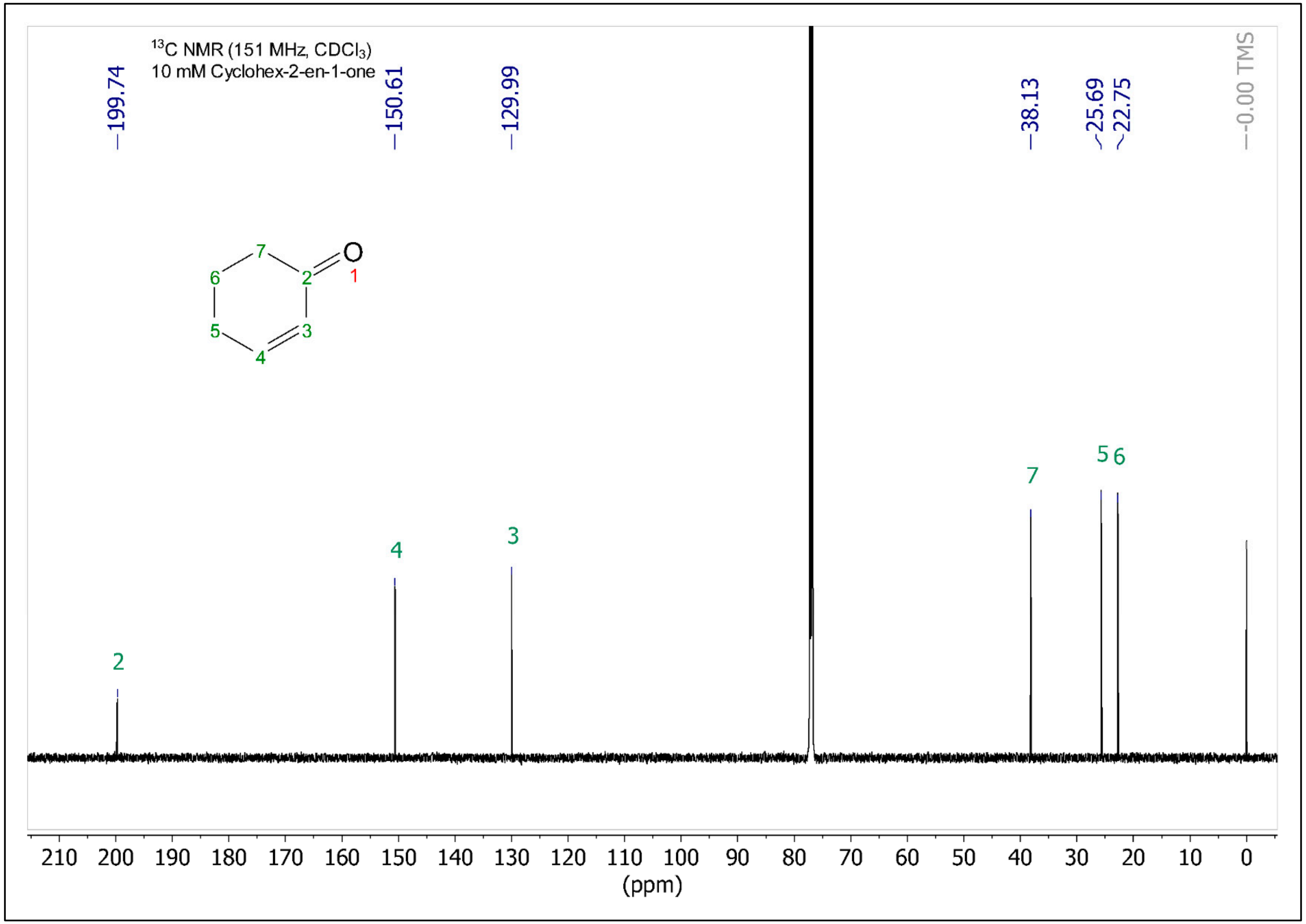

^{13}C NMR (151 MHz, CDCl$_3$)
10 mM Cyclohex-2-en-1-one
199.74
150.61
129.99
38.13
25.69
22.75
-0.00 TMS
(ppm)

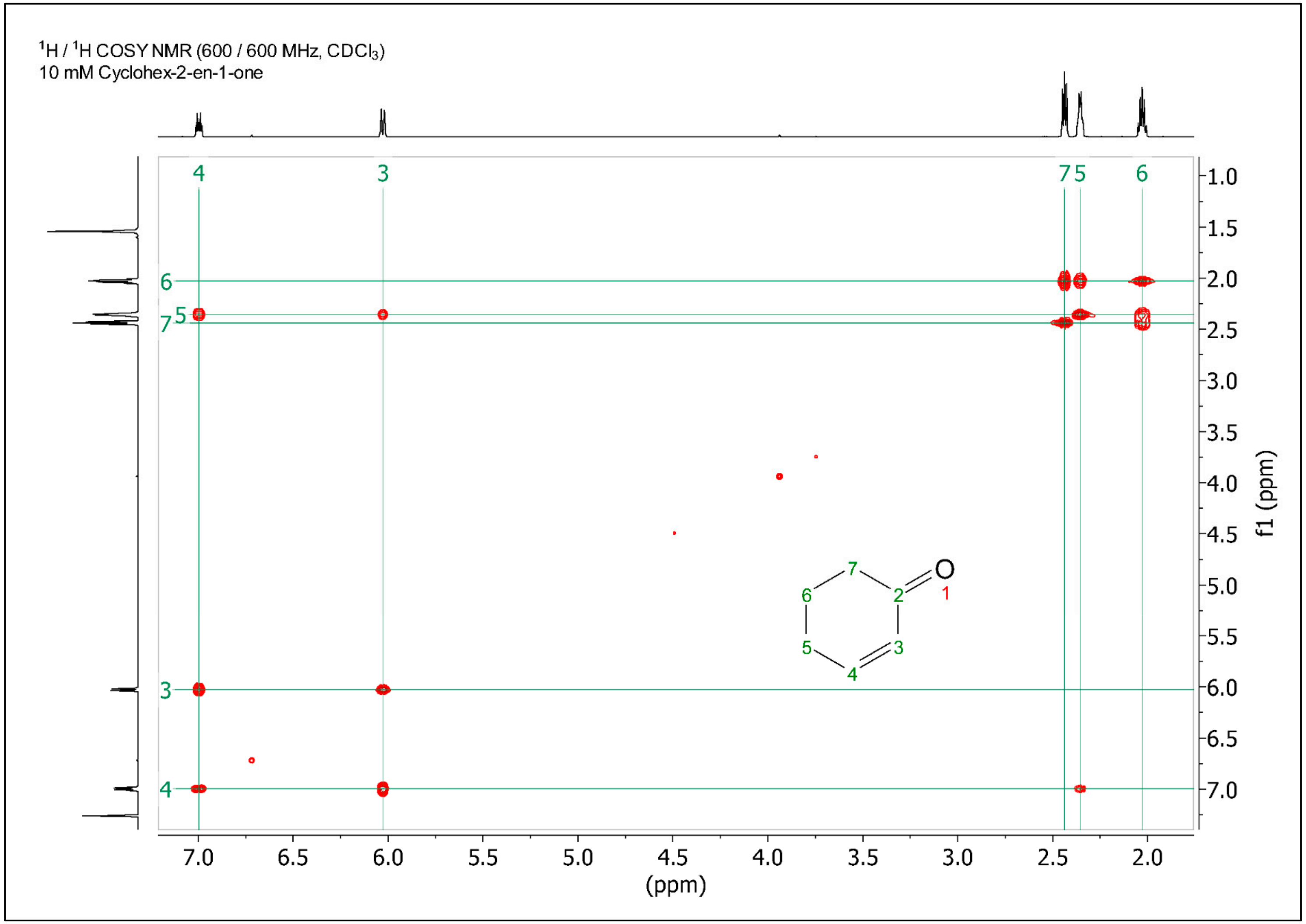
^1H / ^1H COSY NMR (600 / 600 MHz, $CDCl_3$)
10 mM Cyclohex-2-en-1-one
f1 (ppm)
(ppm)
O
1
2
3
4
5
6
7

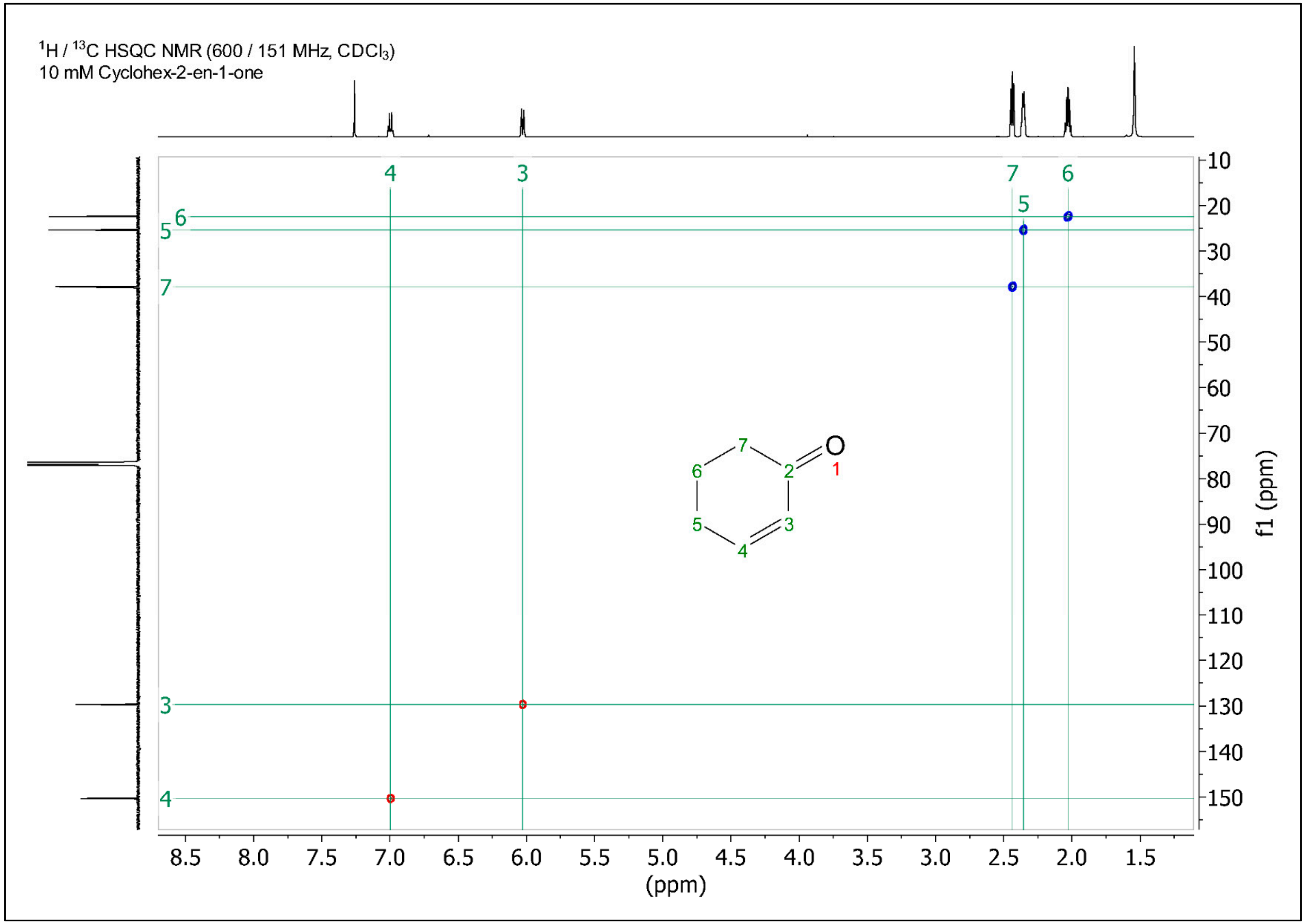

^1H / ^{13}C HSQC NMR (600 / 151 MHz, $CDCl_3$)
10 mM Cyclohex-2-en-1-one
f1 (ppm)
(ppm)

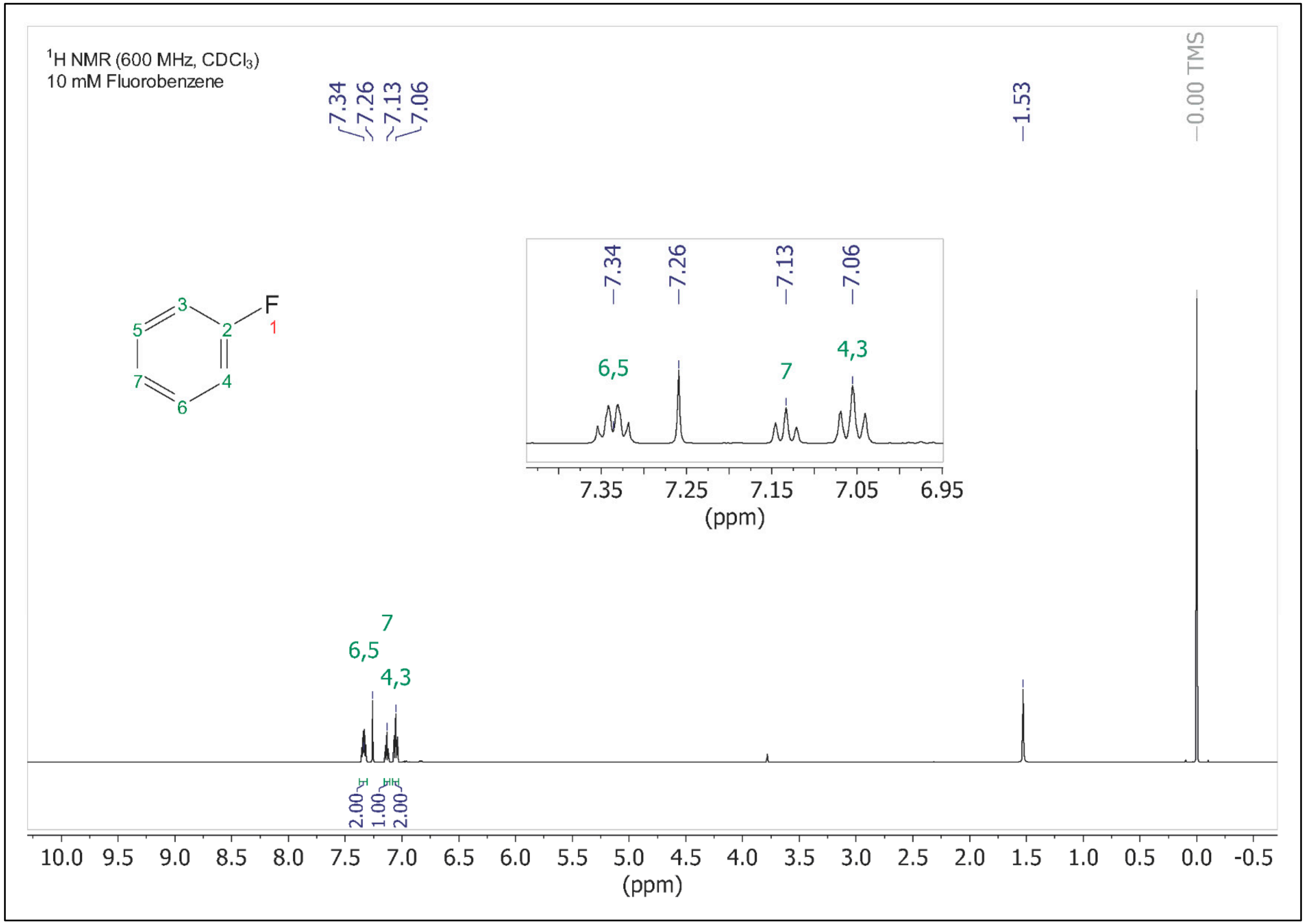

^{1}H NMR (600 MHz, CDCl$_3$)
10 mM Fluorobenzene
7.34
7.26
7.13
7.06
1.53
0.00 TMS
F
1
2
3
4
5
6
7
6,5
7
4,3
7.35
7.25
7.15
7.05
6.95
(ppm)
2.00
1.00
2.00
10.0 9.5 9.0 8.5 8.0 7.5 7.0 6.5 6.0 5.5 5.0 4.5 4.0 3.5 3.0 2.5 2.0 1.5 1.0 0.5 0.0 -0.5
(ppm)

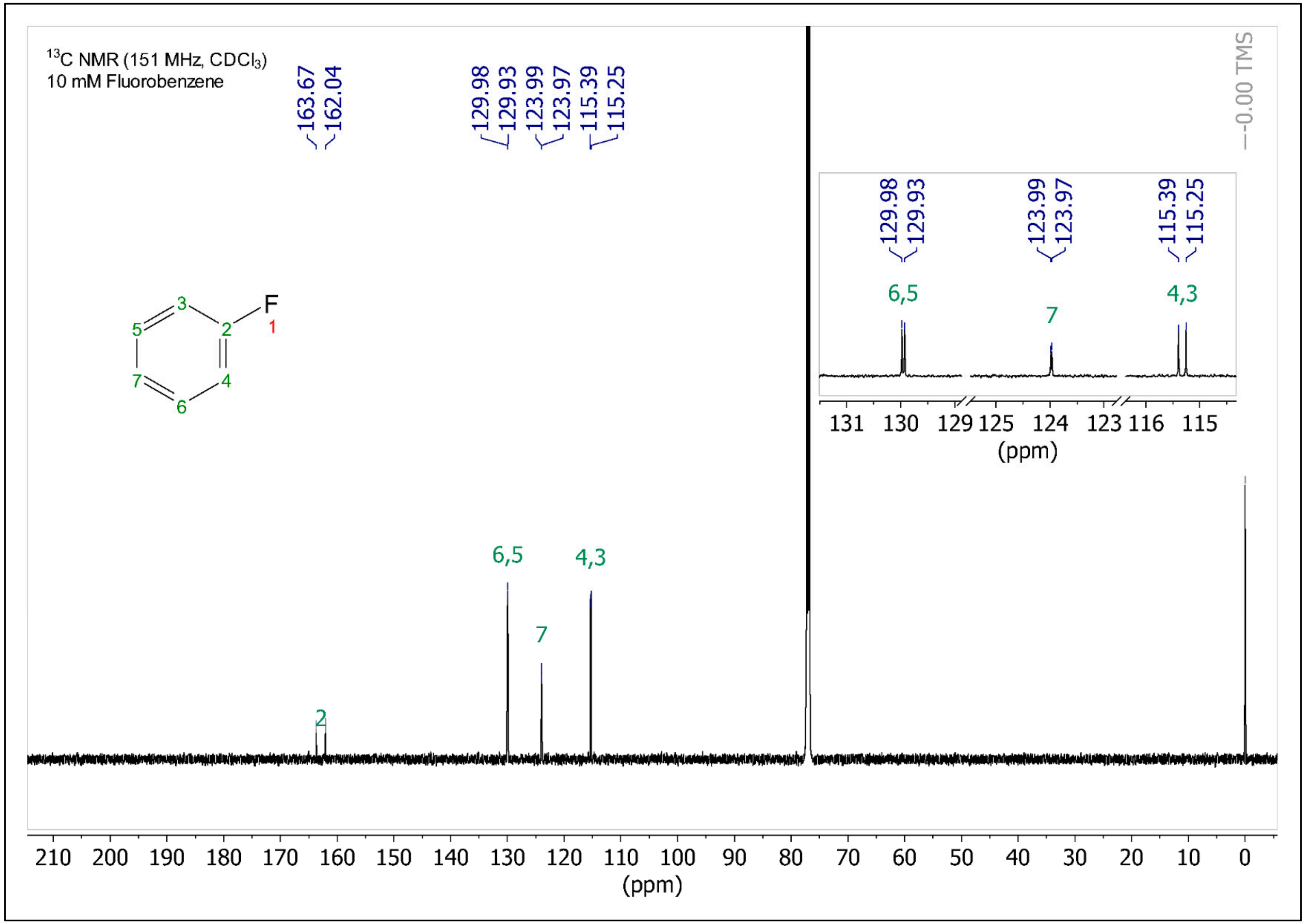

13C NMR (151 MHz, CDCl3)
10 mM Fluorobenzene
163.67
162.04
129.98
129.93
123.99
123.97
115.39
115.25
-0.00 TMS
F
1
2
3
4
5
6
7
6,5
7
4,3
2
131 130 129 125 124 123 116 115
(ppm)
210 200 190 180 170 160 150 140 130 120 110 100 90 80 70 60 50 40 30 20 10 0
(ppm)

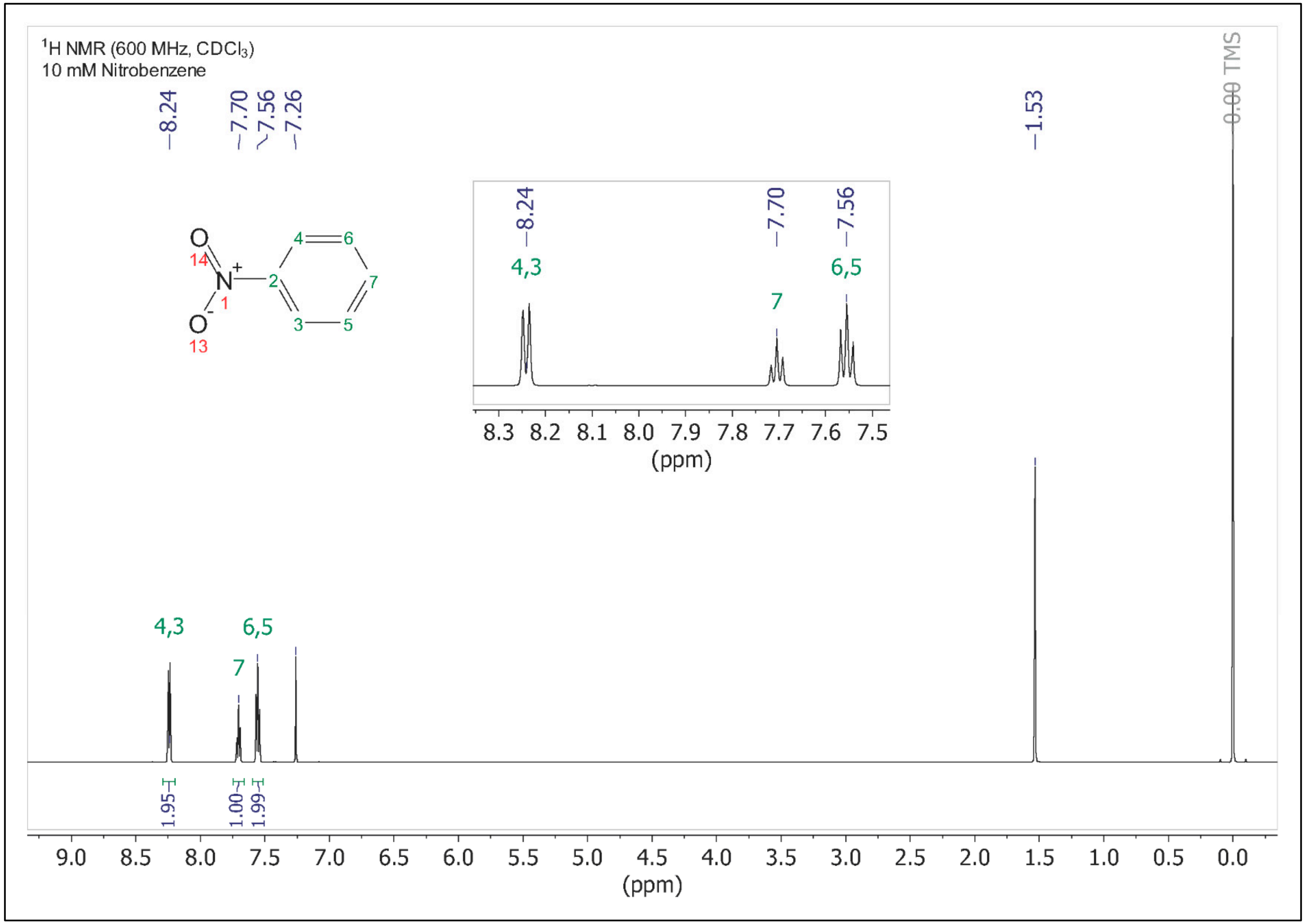

^{1}H NMR (600 MHz, CDCl$_3$)
10 mM Nitrobenzene
8.24
7.70
7.56
7.26
1.53
0.00 TMS
4,3
7
6,5
1.95
1.00
1.99
(ppm)

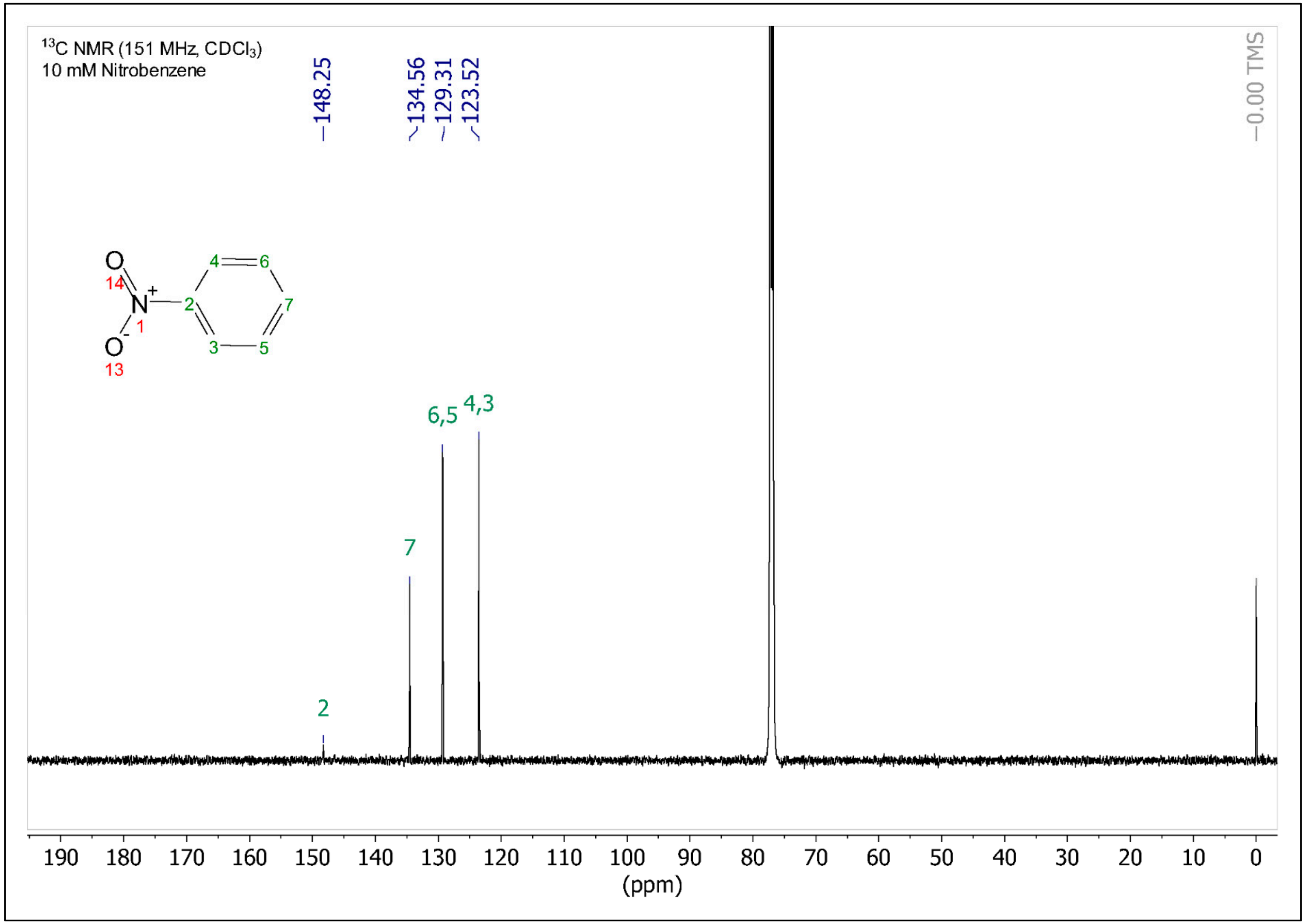

13C NMR (151 MHz, CDCl3)
10 mM Nitrobenzene
148.25
134.56
129.31
123.52
0.00 TMS
2
7
6,5
4,3
190 180 170 160 150 140 130 120 110 100 90 80 70 60 50 40 30 20 10 0
(ppm)

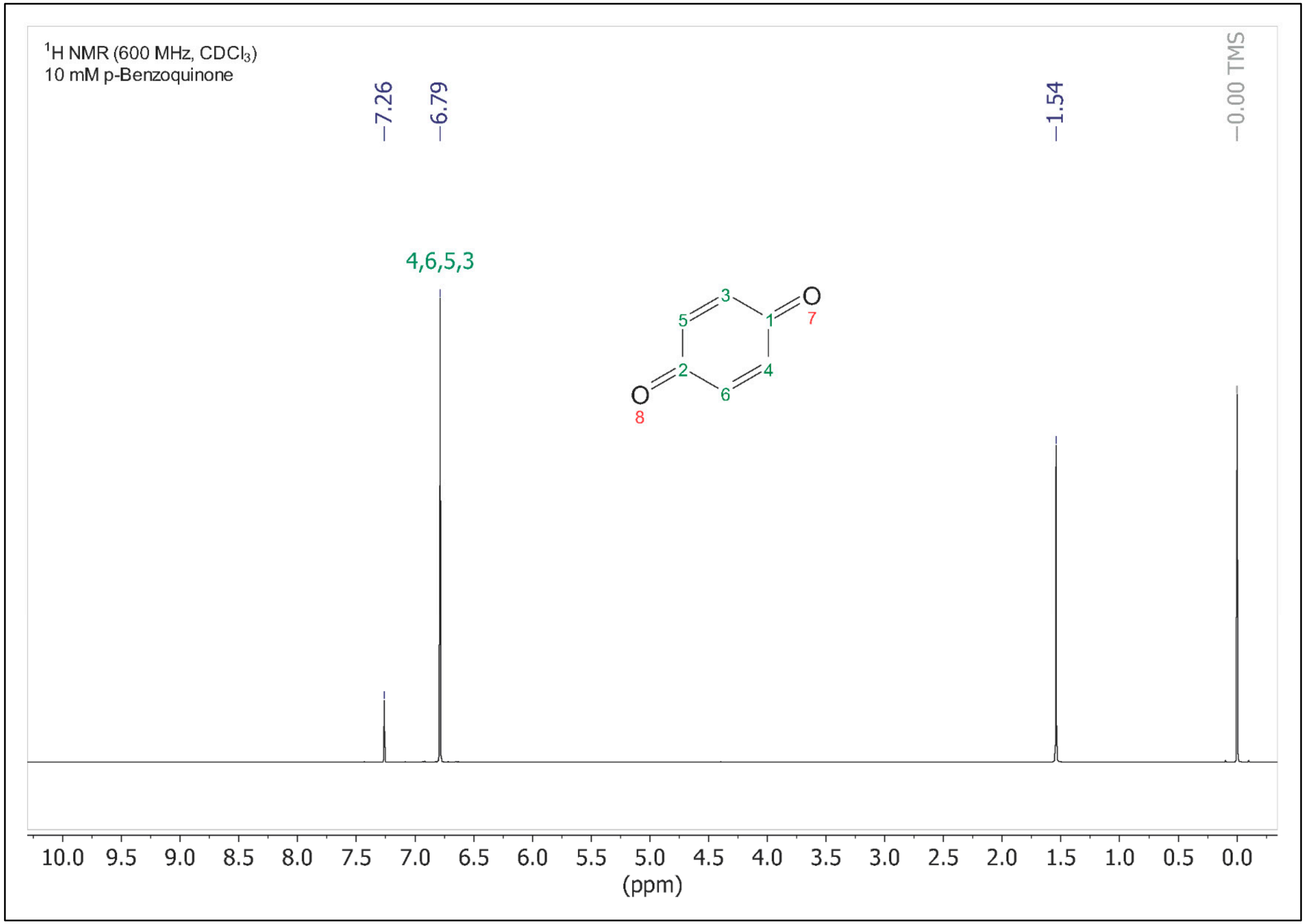

1H NMR (600 MHz, CDCl3)
10 mM p-Benzoquinone
7.26
6.79
1.54
0.00 TMS
4,6,5,3
3
5
1
7
2
4
8
6
O
O
10.0 9.5 9.0 8.5 8.0 7.5 7.0 6.5 6.0 5.5 5.0 4.5 4.0 3.5 3.0 2.5 2.0 1.5 1.0 0.5 0.0
(ppm)

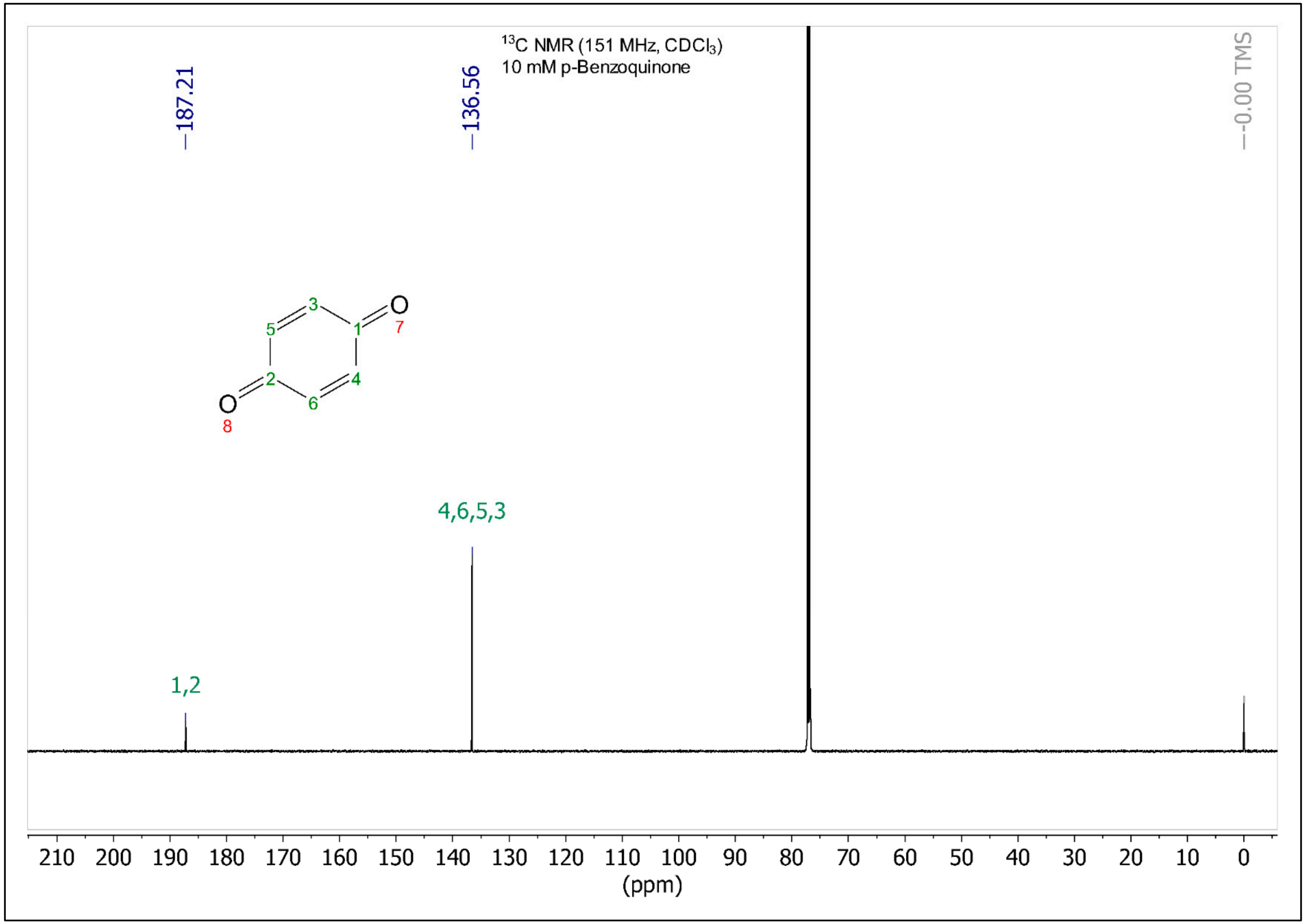

13C NMR (151 MHz, CDCl3)
10 mM p-Benzoquinone
187.21
136.56
-0.00 TMS
1,2
4,6,5,3
210 200 190 180 170 160 150 140 130 120 110 100 90 80 70 60 50 40 30 20 10 0
(ppm)

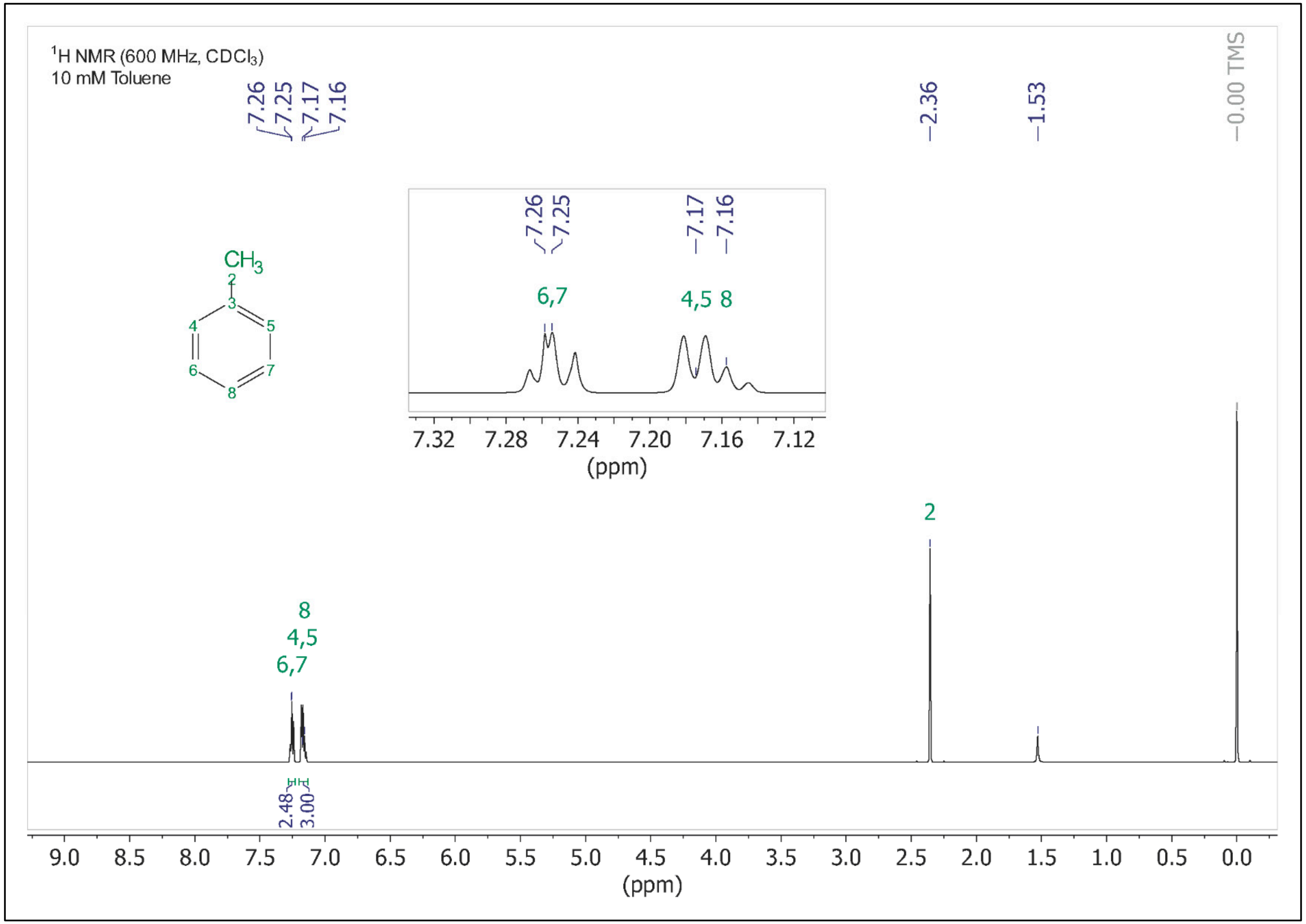

^{1}H NMR (600 MHz, CDCl$_3$)
10 mM Toluene
7.26
7.25
7.17
7.16
2.36
1.53
0.00 TMS
CH3
6,7
4,5 8
2
8
4,5
6,7
2.48
3.00
(ppm)

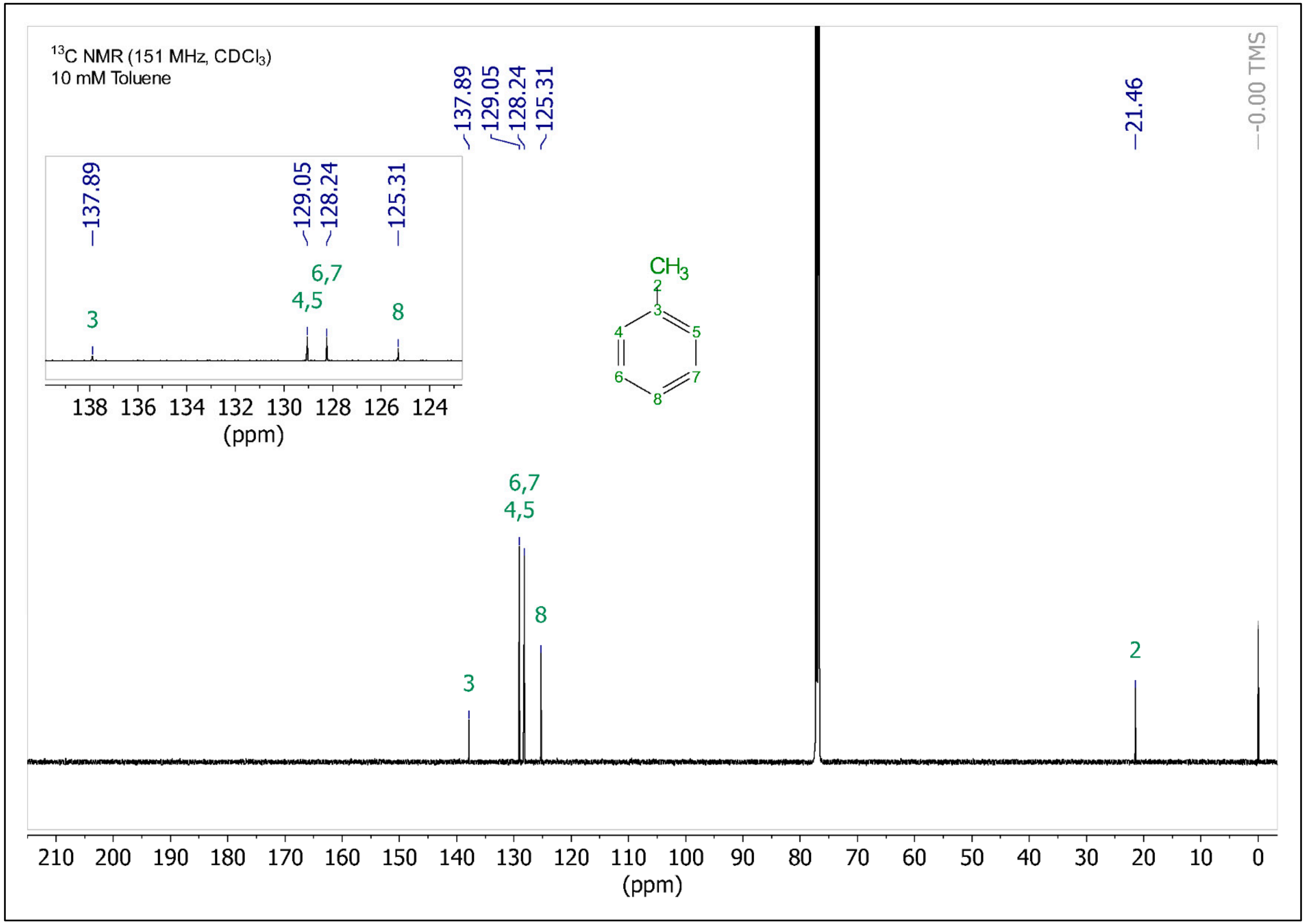

13C NMR (151 MHz, CDCl3)
10 mM Toluene
137.89
129.05
128.24
125.31
21.46
-0.00 TMS
3
4,5
6,7
8
2
CH3
138 136 134 132 130 128 126 124
(ppm)
210 200 190 180 170 160 150 140 130 120 110 100 90 80 70 60 50 40 30 20 10 0
(ppm)

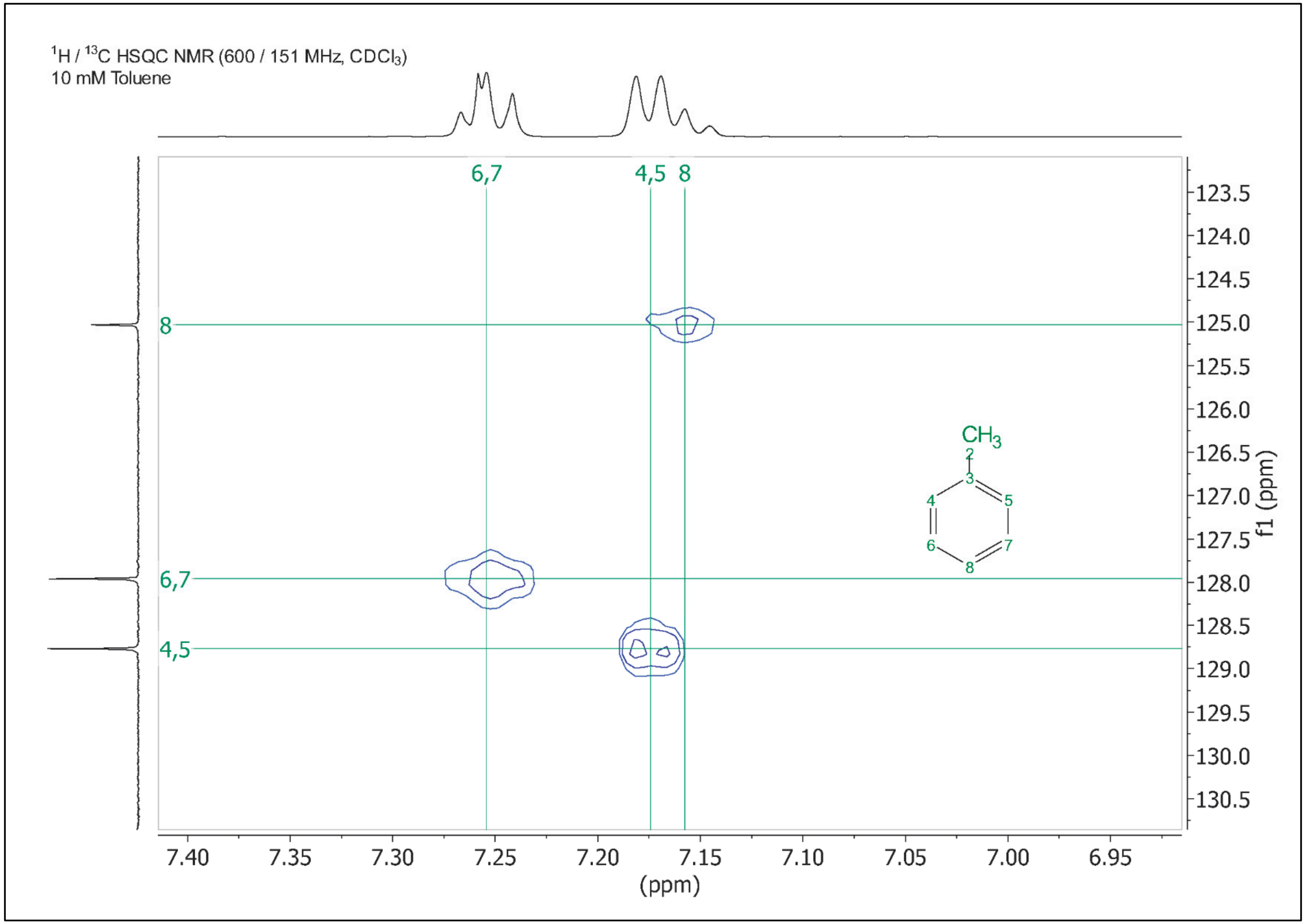

^1H / ^{13}C HSQC NMR (600 / 151 MHz, $CDCl_3$)
10 mM Toluene
6,7
4,5
8
CH_3
f1 (ppm)
(ppm)

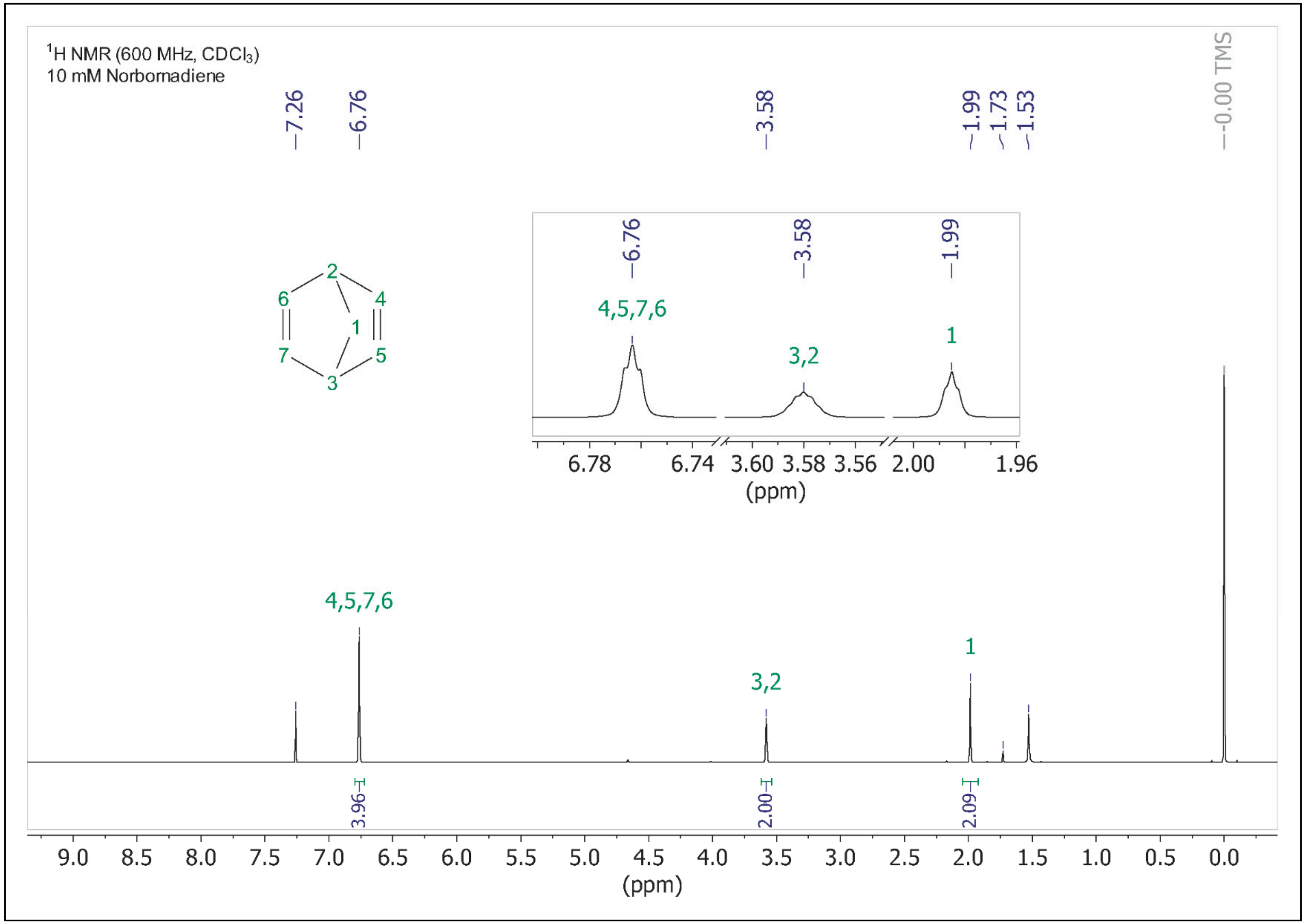

1H NMR (600 MHz, CDCl3)
10 mM Norbornadiene
7.26
6.76
3.58
1.99
1.73
1.53
0.00 TMS
4,5,7,6
3,2
1
6.78
6.74
3.60
3.58
3.56
2.00
1.96
(ppm)
3.96
2.00
2.09
9.0
8.5
8.0
7.5
7.0
6.5
6.0
5.5
5.0
4.5
4.0
3.5
3.0
2.5
2.0
1.5
1.0
0.5
0.0
(ppm)

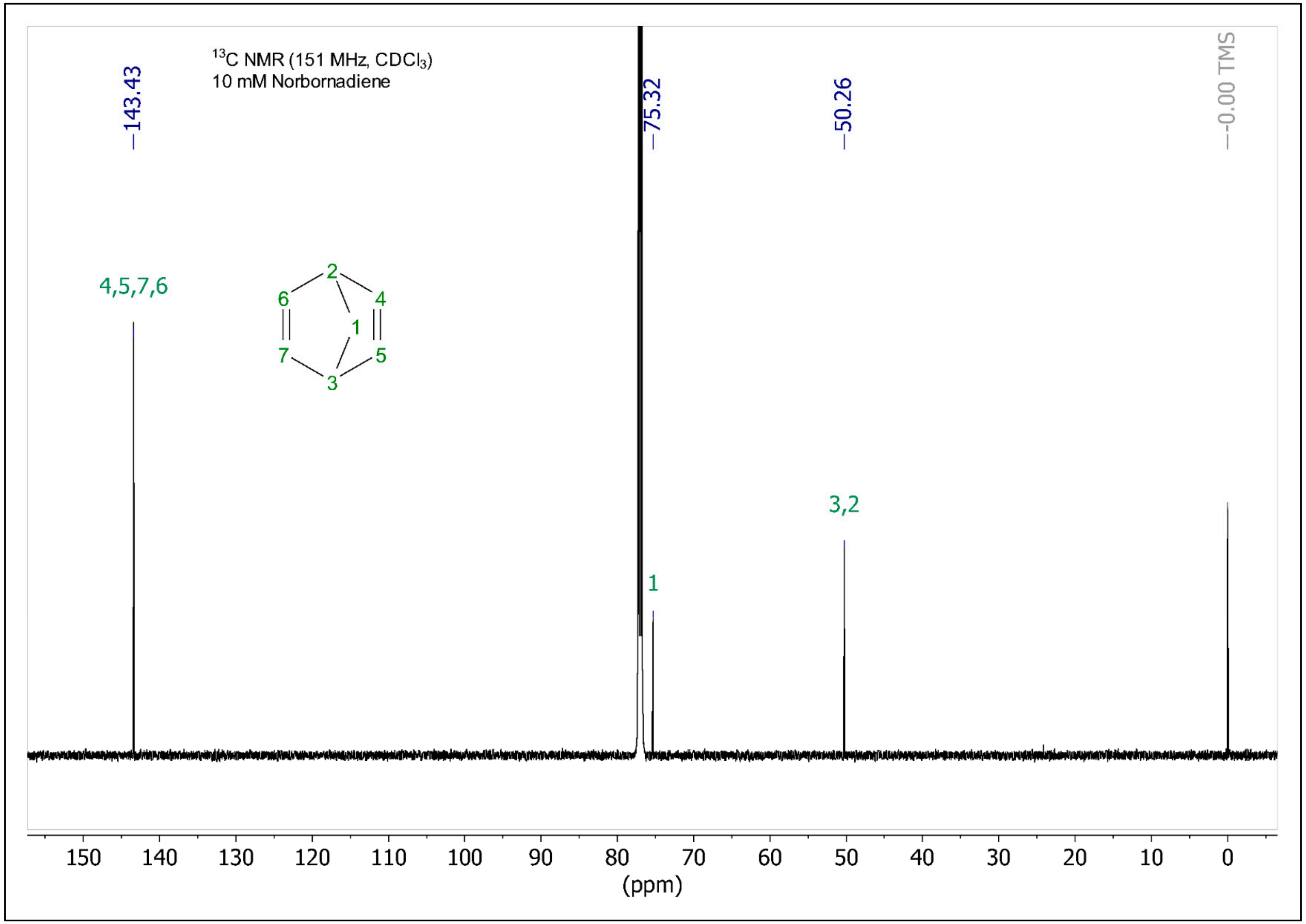

^{13}C NMR (151 MHz, CDCl$_3$)
10 mM Norbornadiene
143.43
75.32
50.26
-0.00 TMS
4,5,7,6
1
3,2
150 140 130 120 110 100 90 80 70 60 50 40 30 20 10 0
(ppm)

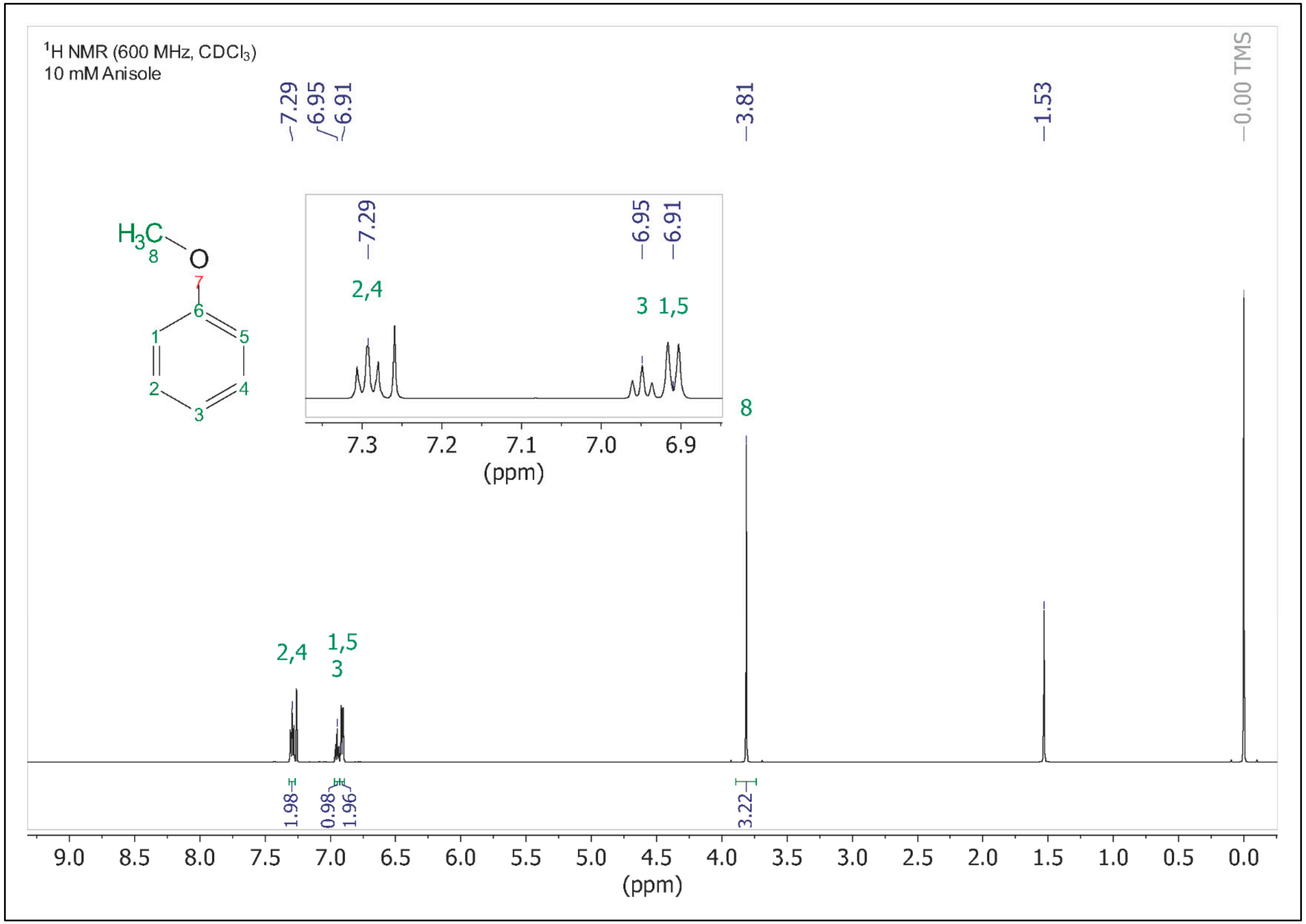

^{1}H NMR (600 MHz, CDCl$_3$)
10 mM Anisole
7.29
6.95
6.91
3.81
1.53
0.00 TMS
H_3C
O
8
7
6
1
2
3
4
5
2,4
3 1,5
8
1,5
3
1.98
0.98
1.96
3.22
7.3 7.2 7.1 7.0 6.9
(ppm)
9.0 8.5 8.0 7.5 7.0 6.5 6.0 5.5 5.0 4.5 4.0 3.5 3.0 2.5 2.0 1.5 1.0 0.5 0.0
(ppm)

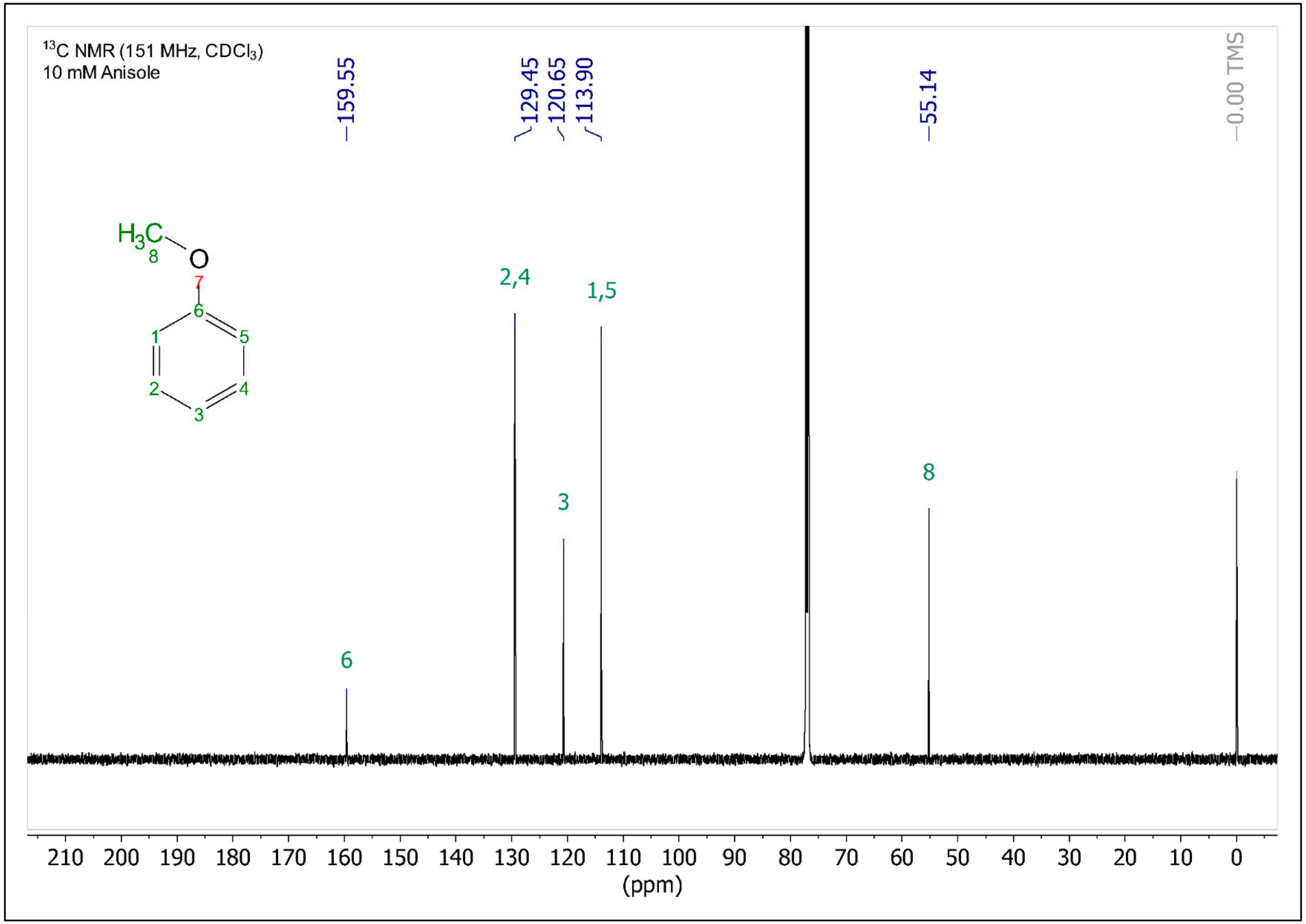

^{13}C NMR (151 MHz, CDCl$_3$)
10 mM Anisole
159.55
129.45
120.65
113.90
55.14
0.00 TMS
H3C
8
O
7
6
1
5
2
4
3
2,4
1,5
3
8
6
210 200 190 180 170 160 150 140 130 120 110 100 90 80 70 60 50 40 30 20 10 0
(ppm)

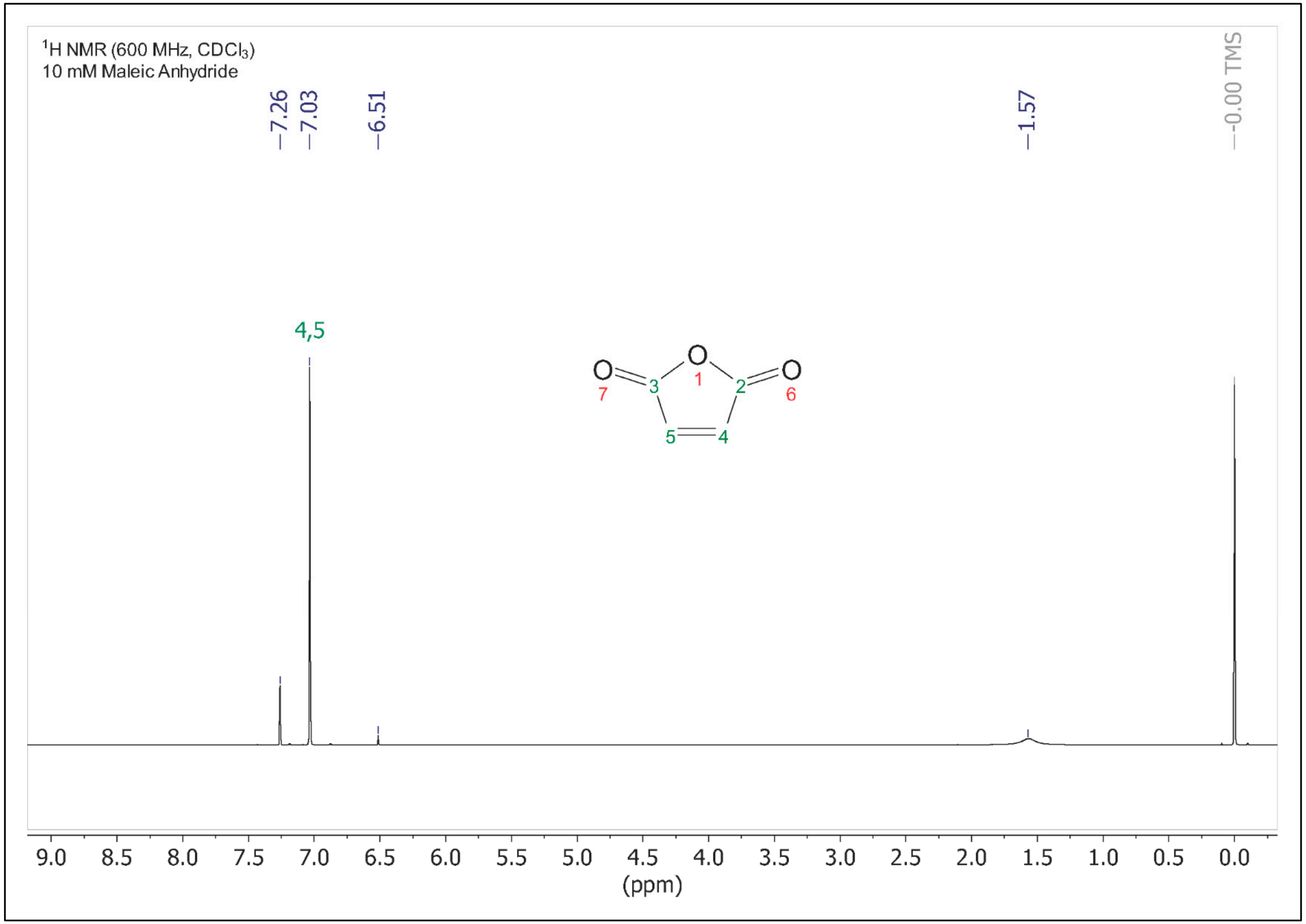

^{1}H NMR (600 MHz, CDCl$_3$)
10 mM Maleic Anhydride
7.26
7.03
6.51
1.57
-0.00 TMS
4,5
O
1
2
3
4
5
O
6
O
7
9.0
8.5
8.0
7.5
7.0
6.5
6.0
5.5
5.0
4.5
4.0
3.5
3.0
2.5
2.0
1.5
1.0
0.5
0.0
(ppm)

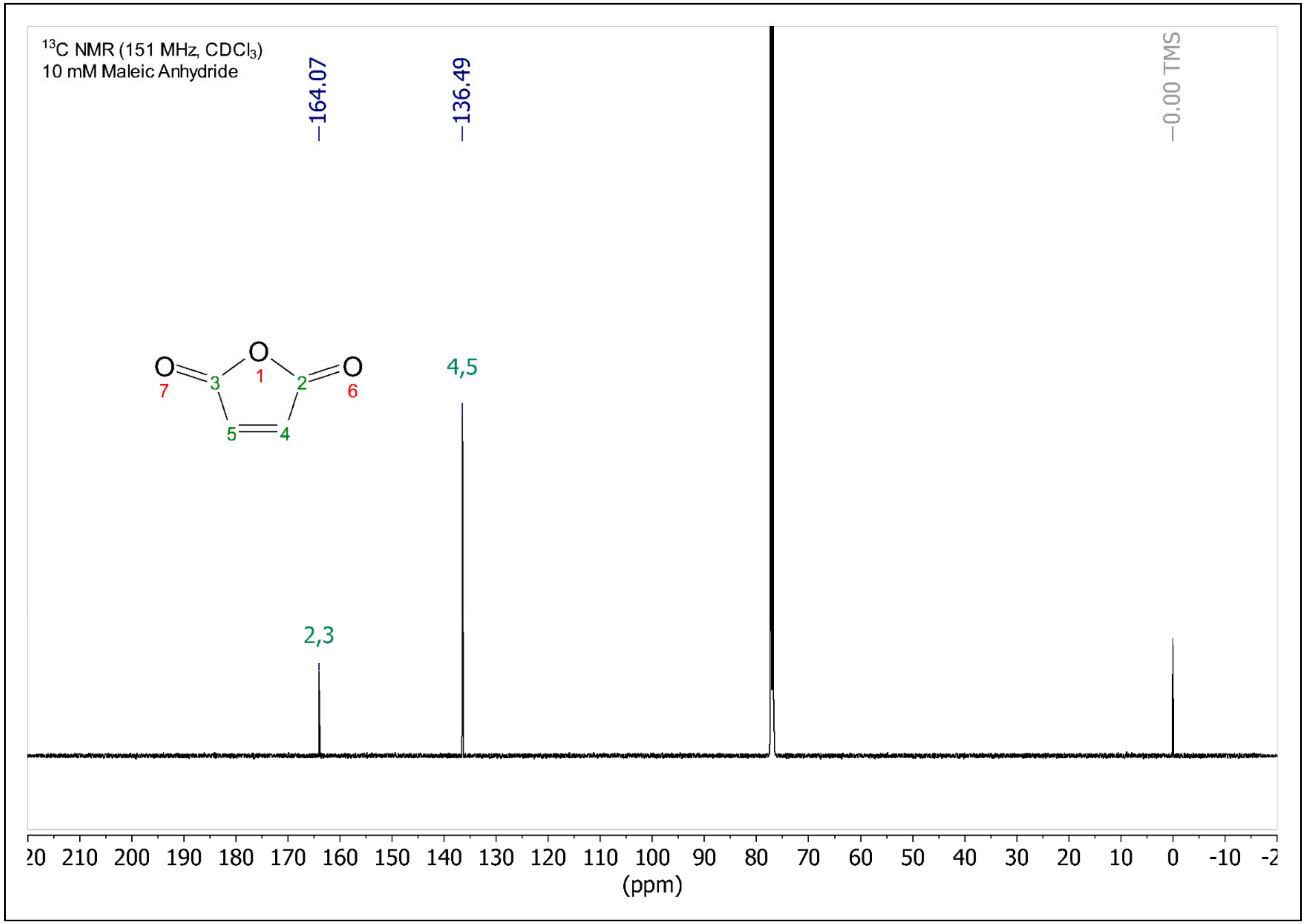

13C NMR (151 MHz, CDCl3)
10 mM Maleic Anhydride
164.07
136.49
0.00 TMS
4,5
2,3
O
1
O
7
3
O
6
2
5
4
20 210 200 190 180 170 160 150 140 130 120 110 100 90 80 70 60 50 40 30 20 10 0 -10 -2
(ppm)

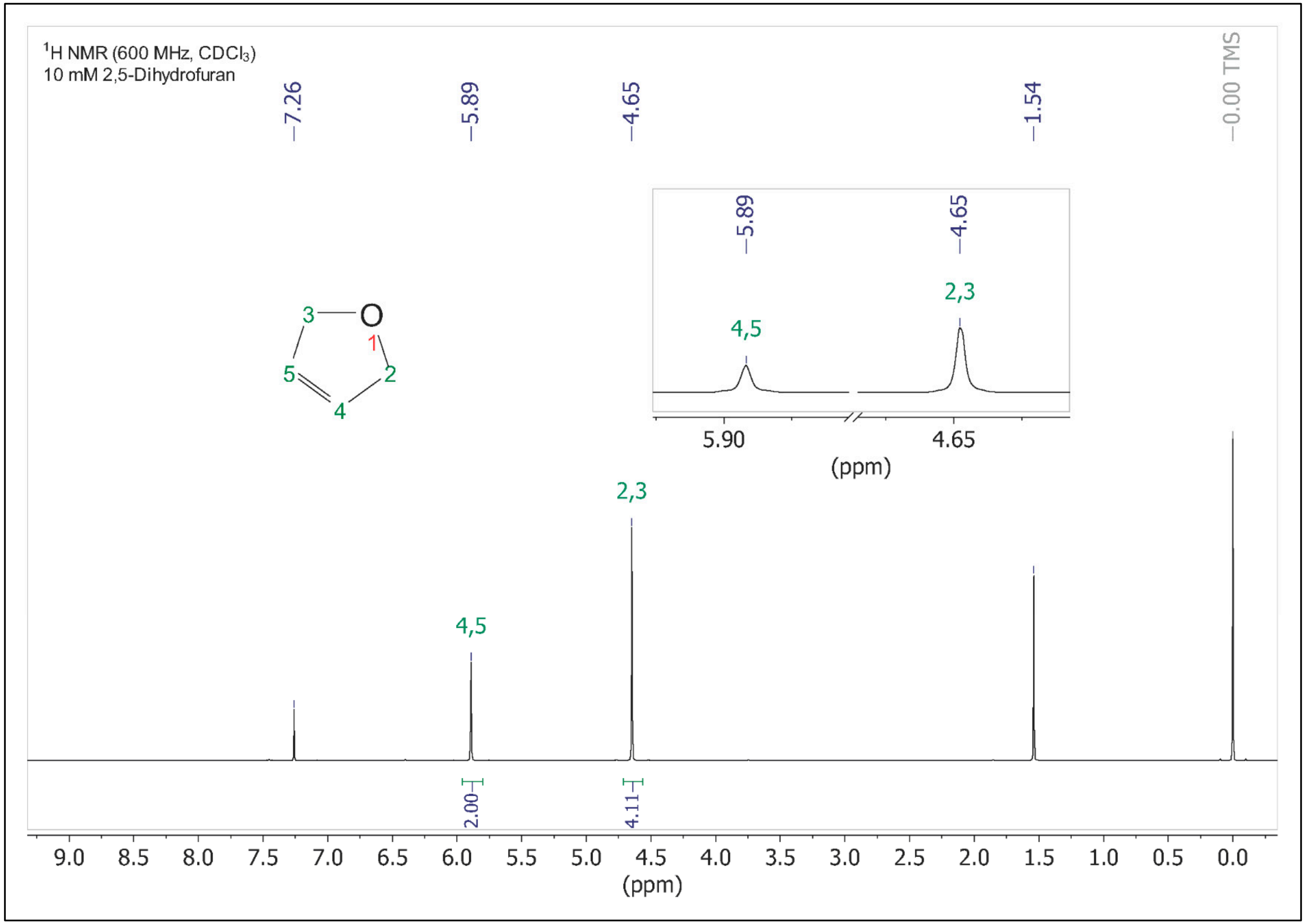

1H NMR (600 MHz, CDCl3)
10 mM 2,5-Dihydrofuran
7.26
5.89
4.65
1.54
0.00 TMS
3
O
1
5
2
4
5.89
4.65
2,3
4,5
5.90
4.65
(ppm)
2,3
4,5
2.00
4.11
9.0
8.5
8.0
7.5
7.0
6.5
6.0
5.5
5.0
4.5
4.0
3.5
3.0
2.5
2.0
1.5
1.0
0.5
0.0
(ppm)

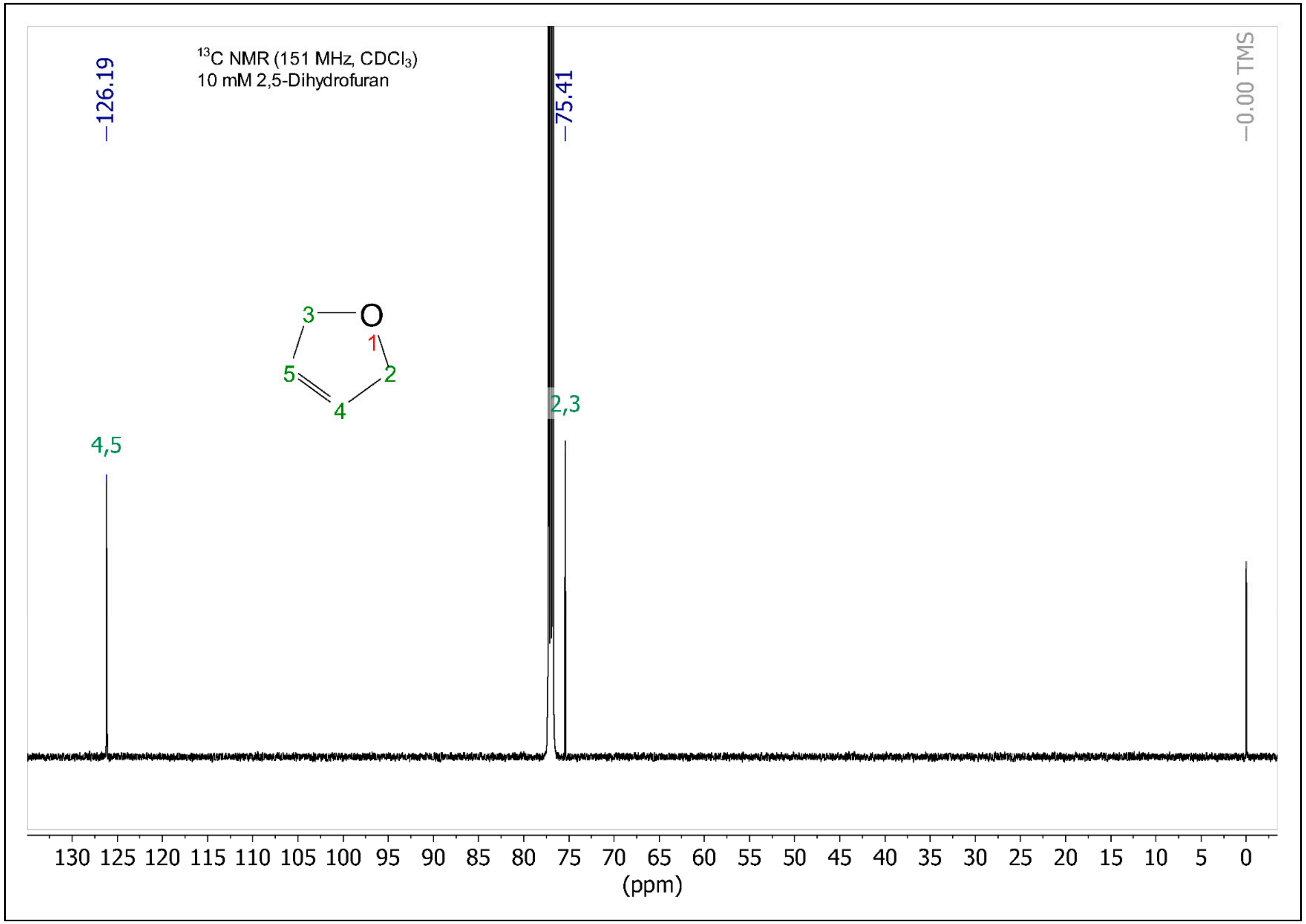

^{13}C NMR (151 MHz, CDCl$_3$)
10 mM 2,5-Dihydrofuran
–126.19
–75.41
–0.00 TMS
4,5
2,3
3
O
1
2
5
4
130 125 120 115 110 105 100 95 90 85 80 75 70 65 60 55 50 45 40 35 30 25 20 15 10 5 0
(ppm)

III. Probe Set NMR Data

The following small- to medium-sized synthetic organic compounds and natural products were included in the probe set: bicyclo[2.1.1]hexan-2-one, α-pinene, aquatolide, naupliolide, echinopine B, parthenolide, diepoxyguaianolide, cannabicitran (CBT-C), ingenane diterpene 8, artemisinin, nobilistine A, intricarene, strychnine, holstiine, colchicine, hexacyclinol, homodimericin A, strychnobaillonine, sungucine, and paclitaxel. Results from DFT calculations of ^{1}H and ^{13}C NMR chemical shifts of each compound are listed in Table S1 through Table S22.

Table S1. Comparison of DFT δ_H and δ_C predictions for **bicyclo[2.1.1]hexan-2-one**

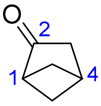

	Method: Expt[d]	1[a] DFT	2[b] DFT	Lit.[c] DFT	ML[e]		Method: Expt[d]	1[a] DFT	2[b] DFT	Lit.[c] DFT	ML[e]
Atom	δ_H (ppm)					Atom	δ_C (ppm)				
1	2.83	2.82	2.79	2.67	3.00	1	56.1	**55.3**	55.5	**57.8**	**45.9**
3	2.14	2.13	2.19	2.08	1.99	2	213.7	214.0	213.9	213.7	211.5
4	2.83	2.85	**2.71**	**2.63**	2.60	3	40.8	41.5	**41.5**	41.6	42.7
5a	1.62	1.65	1.71	1.59	1.63	4	36.0	35.4	35.5	37.4	31.9
5b	2.22	**2.27**	2.24	2.20	**1.89**	5	40.9	40.2	40.4	40.7	31.3
	RMSD:[f]	0.03	0.07	0.12	0.21		RMSD:[f]	0.6	0.5	1.1	6.7
	MD:[f]	0.05	0.12	0.20	0.33		MD:[f]	0.8	0.7	1.7	10.2

[a:] Data for method 1 (current study – see Table 7 of the main text for details on the model chemistry).
[b:] Data for method 2 (current study – see Table 7 of the main text for details on the model chemistry).
[c:] Data reported by Ref [4]. NMR calculations (both δ_H and δ_C) at the mPW1PW91/6-311+G(2d,p) level with CPCM/UAKS solvent model for chloroform on B3LYP/6-31+G(d,p) geometries. Linear scaling factors were used to convert isotropic magnetic shielding values, σ_H and σ_C, to chemical shifts, δ_H and δ_C, using the formulas, $\delta_H = (31.8486 - \sigma_H)/1.08225$ and $\delta_C = (186.060 - \sigma_C)/1.04478$.
[d:] Experimental data from Ref [5] obtained in $CDCl_3$ on a 400 MHz NMR spectrometer.
[e:] Results from Machine Learning (ML) / DFT approach that were calculated using an online app at http://nova.chem.colostate.edu/cascade; method details are from Ref [6].
[f:] RMSD = root mean square deviation, MD = maximum deviation (largest errors highlighted in blue).

Table S2. Comparison of DFT δ_H and δ_C predictions for **α-pinene**

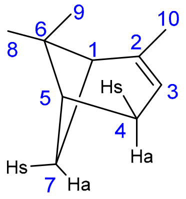

α-pinene

	Method: Expt[d]	1[a] DFT	2[b] DFT	Lit.[c] DFT		Method: Expt[d]	1[a] DFT	2[b] DFT	Lit.[c] DFT
Atom	δ_H (ppm)				Atom	δ_C (ppm)			
1	1.93	2.04	**2.08**	1.39	1	47.0	46.0	46.1	48.8
3	5.19	**5.38**	5.29	**4.05**	2	144.5	**148.0**	**148.1**	149.3
4a	2.23	2.23	2.24	1.60	3	116.0	119.1	118.5	120.5
4s	2.16	2.15	2.23	1.62	4	31.3	31.6	31.5	34.4
5	2.07	2.09	2.13	1.48	5	40.7	39.8	39.7	44.2
7a	1.15	1.19	1.11	0.81	6	38.0	38.6	38.4	**45.4**
7s	2.33	2.25	2.25	1.66	7	31.5	32.4	32.3	34.0
8	1.26	1.23	1.24	0.79	8	26.4	26.7	26.6	26.1
9	0.83	0.88	0.88	0.64	9	20.8	21.5	21.4	20.5
10	1.66	1.71	1.75	0.98	10	23.0	23.4	23.5	23.7
	RMSD:[e]	0.08	0.08	0.63		RMSD:[e]	1.6	1.5	3.6
	MD:[e]	0.19	0.15	1.14		MD:[e]	3.5	3.6	7.4

[a:] Data for method 1 (current study – see Table 7 of the main text for details on the model chemistry)
[b:] Data for method 2 (current study – see Table 7 of the main text for details on the model chemistry)
[c:] Calculated chemical shifts from [7]. GIAO NMR calculations from B3LYP (basis set not indicated). Chemical shift referencing using the equation, $\delta_{\alpha\text{-pinene}} = \sigma_{TMS} - \sigma_{\alpha\text{-pinene}}$
[d:] Experimental data from [8] at 0.1 M in $CDCl_3$ at 21-22°C; chemical shift referencing relative to TMS.
[e:] RMSD = root mean square deviation, MD = maximum deviation (largest errors highlighted in blue).

Table S3. Comparison of DFT δ_H and δ_C predictions for **aquatolide**

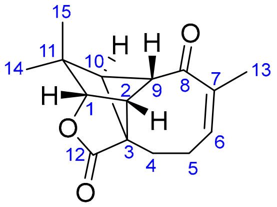

	Method: Expt[d]	1[a] DFT	2[b] DFT	Lit.[c] DFT		Method: Expt[d]	1[a] DFT	2[b] DFT	Lit.[c] DFT
Atom	δ_H (ppm)				Atom	δ_C (ppm)			
1	4.48	4.39	4.40	**4.21**	1	84.2	82.9	83.7	83.3
2	3.26	3.19	**3.15**	3.20	2	54.5	53.5	53.6	54.8
4a	2.52	2.49	2.54	2.48	3	62.8	61.5	61.6	62.8
4b	1.96	**1.70**	1.92	1.89	4	22.2	23.5	23.0	22.5
5a	2.35	2.31	2.30	2.29	5	28.6	29.0	28.8	30.5
5b	2.03	2.08	2.09	2.08	6	131.1	**135.2**	133.8	**135.2**
6	5.85	6.03	5.85	5.94	7	135.1	138.4	**138.3**	138.1
9	2.92	2.88	2.96	2.78	8	211.9	214.1	214.4	211.9
10	2.64	2.63	2.64	2.49	9	54.5	55.0	55.0	54.8
13	1.87	1.90	1.90	1.84	10	62.6	62.8	62.8	63.9
14	1.05	0.99	1.08	0.98	11	41.9	42.5	42.2	44.6
15	1.19	1.16	1.17	1.09	12	177.5	177.3	176.3	177.7
					13	22.2	24.4	24.0	23.2
					14	22.6	23.6	23.6	21.1
					15	22.8	23.2	23.2	20.8
	RMSD:[e]	0.10	0.05	0.11		RMSD:[e]	1.7	1.5	1.8
	MD:[e]	0.26	0.11	0.27		MD:[e]	4.1	3.2	4.1

[a:] Data for method 1 (current study – see Table 7 of the main text for details on the model chemistry).

[b:] Data for method 2 (current study – see Table 7 of the main text for details on the model chemistry).

[c:] Data reported by Ref [4]. NMR calculations (both δ_H and δ_C) at the mPW1PW91/6-311+G(2d,p) level with CPCM/UAKS solvent model for chloroform on B3LYP/6-31+G(d,p) geometries. Linear scaling factors were used to convert isotropic magnetic shielding values, σ_H and σ_C, to chemical shifts, δ_H and δ_C, using the formulas, $\delta_H = (31.8486 - \sigma_H)/1.08225$ and $\delta_C = (186.060 - \sigma_C)/1.04478$.

[d:] Experimental data from Ref [4] in $CDCl_3$.

[e:] RMSD = root mean square deviation, MD = maximum deviation (largest errors highlighted in blue).

Table S4. Comparison of DFT δ_H and δ_C predictions for **naupliolide**

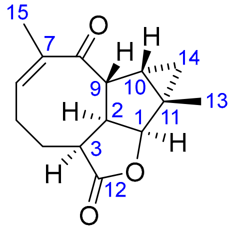

	Method: Expt[d]	1[a] DFT	2[b] DFT	Lit.[c] DFT		Method: Expt[d]	1[a] DFT	2[b] DFT	Lit.[c] DFT
Atom	δ_H (ppm)				Atom	δ_C (ppm)			
1	4.66	4.58	4.66	4.50	1	89.1	88.2	89.8	88.3
2	2.59	2.44	**2.39**	2.49	2	42.0	43.8	42.8	45.2
3	2.94	2.76	2.94	2.85	3	43.4	44.8	43.8	45.2
4a	1.79	1.70	1.68	1.60	4	23.0	24.8	24.2	25.0
4b	2.39	2.37	2.32	2.34	5	25.3	26.1	24.9	25.0
5a	2.30	2.16	2.40	2.30	6	138.7	**144.2**	**144.3**	**146.5**
5b	2.54	2.60	2.60	2.59	7	136.1	138.2	137.3	140.1
6	6.34	6.54	6.53	6.74	8	203.8	206.7	203.6	203.5
9	3.66	3.64	3.74	3.87	9	51.9	50.0	51.9	48.1
10	1.61	1.47	1.50	1.33	10	28.4	31.1	30.2	32.2
13[e]	1.34	1.29	1.34	1.28	11	25.3	26.3	26.4	28.0
14a	0.57	0.67	0.72	**1.15**	12	177.0	175.8	175.0	176.8
14b	0.64	**0.97**	0.71	0.61	13	16.1	17.1	17.1	15.5
15[e]	1.84	1.85	1.88	1.77	14	13.2	15.0	14.8	14.4
					15	21.0	24.3	23.8	21.7
	RMSD:[e]	0.14	0.10	0.23		RMSD:[e]	2.3	1.9	3.0
	MD:[e]	0.33	0.20	0.58		MD:[e]	5.5	5.6	7.8

a: Data for method 1 (current study – see Table 7 of the main text for details on the model chemistry).
b: Data for method 2 (current study – see Table 7 of the main text for details on the model chemistry).
c: Data reported by Ref [4]. NMR calculations (both δ_H and δ_C) at the mPW1PW91/6-311+G(2d,p) level with CPCM/UAKS solvent model for chloroform on B3LYP/6-31+G(d,p) geometries. Linear scaling factors were used to convert isotropic magnetic shielding values, σ_H and σ_C, to chemical shifts, δ_H and δ_C, using the formulas, $\delta_H = (31.8486 - \sigma_H)/1.08225$ and $\delta_C = (186.060 - \sigma_C)/1.04478$.
d: Experimental data from Ref [9] in $CDCl_3$.
e: Note that DFT predictions suggest a misassignment of the chemical shifts, H13 and H15.
e: RMSD = root mean square deviation, MD = maximum deviation (largest errors highlighted in blue).

Table S5. Comparison of DFT δ_H and δ_C predictions for **echinopine B**

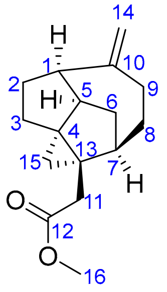

Atom	Method: Expt[d]	1[a] DFT	2[b] DFT	Lit[c] DFT	Atom	Method: Expt[d]	1[a] DFT	2[b] DFT	Lit[c] DFT
	δ_H (ppm)					δ_C (ppm)			
1	2.80	2.72	2.82	2.86	1	48.5	50.0	50.0	51.2
2a	2.14	2.10	2.13	2.14	2	30.9	31.8	31.7	31.6
2b	1.65	1.55	1.71	1.59	3	25.4	27.4	27.2	27.7
3a	1.93	2.01	1.95	1.92	4	**41.0**[e]	43.6	43.4	**46.5**
3b	1.51	1.51	1.53	1.57	5	48.4	49.8	49.8	50.0
5	2.25	2.29	2.31	2.24	6	30.3	31.5	31.3	30.7
6a	1.44	1.37	1.41	1.45	7	40.4	41.4	41.1	42.7
6b	1.35	1.31	1.40	1.40	8	32.5	32.9	32.9	33.1
7	2.39	2.39	2.32	2.22	9	29.3	30.0	29.7	30.5
8a	1.93	2.00	2.09	1.92	10	154.3	155.6	156.1	158.6
8b	1.26	1.22	1.33	1.29	11	35.1	36.5	36.4	35.6
9a	2.11	2.07	2.11	2.08	12	173.7	173.7	173.0	174.9
9b	1.82	**1.99**	**2.04**	**2.04**	13	**29.8**[e]	**32.0**	**32.3**	34.9
11a	2.65	2.62	2.70	2.63	14	111.9	113.6	113.2	111.4
11b	1.72	1.84	1.84	1.57	15	15.7	17.2	17.2	15.1
14a	4.63	4.70	4.73	4.68	16	51.3	51.7	52.3	50.7
14b	4.60	4.66	4.66	4.65					
15a	0.69	0.68	0.74	0.73					
15b	0.47	0.53	0.57	0.59					
16	3.68	3.65	3.64	3.46					
	RMSD:[f]	0.07	0.08	0.10		RMSD:[f]	1.4	1.4	2.5
	MD:[f]	0.17	0.22	0.22		MD:[f]	2.6	2.5	5.5

a: Data for method 1 (current study – see Table 7 of the main text for details on the model chemistry)

b: Data for method 2 (current study – see Table 7 of the main text for details on the model chemistry)

c: Calculated chemical shifts from Ref [4]. NMR calculations (both δ_H and δ_C) at the mPW1PW91/6-311+G(2d,p) level with CPCM/UAKS solvent model for chloroform on B3LYP/6-31+G(d,p) geometries. Linear scaling factors were used to convert isotropic magnetic shielding values, σ_H and σ_C, to chemical shifts, δ_H and δ_C, using the formulas, $\delta_H = (31.8486 - \sigma_H)/1.08225$ and $\delta_C = (186.060 - \sigma_C)/1.04478$.

d: Experimental ^{1}H and ^{13}C NMR data acquired in $CDCl_3$ on a 500 MHz NMR spectrometer [10].

e: The δ_C chemical shifts for atoms 4 and 13 appear to be misassigned in the original experimental report [10]. They were swapped here based on the DFT predictions, which was also performed in the previous DFT paper from Ref [4].

f: RMSD = root mean square deviation, MD = maximum deviation (largest errors highlighted in blue).

Table S6. Comparison of DFT δ_H and δ_C predictions for **parthenolide**

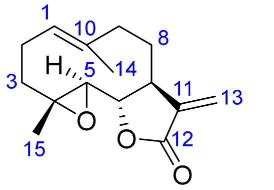

	Method: Expt[c]	1[a] DFT	2[b] DFT		Method: Expt[c]	1[a] DFT	2[b] DFT
Atom	δ_H (ppm)			Atom	δ_C (ppm)		
1	5.21	5.23	5.21	1	125.3	126.7	126.5
2a	2.11-2.21[d]	2.20	2.25	2	24.1	25.3	25.1
2b	2.32-2.49[d]	2.36	2.42	3	36.3	37.2	36.8
3a	1.20-1.28[d]	1.29	1.30	4	61.5	61.3	62.2
3b	2.11-2.21[d]	2.01	2.09	5	66.4	66.9	67.0
5	2.79	**2.65**	2.76	6	82.4	82.6	83.7
6	3.86	3.73	**3.90**	7	47.7	49.3	48.8
7	2.74-2.82[d]	2.74	2.77	8	30.6	32.5	32.1
8a	2.11-2.21[d]	2.01	2.01	9	41.2	41.9	41.9
8b	1.70-1.77[d]	1.69	1.81	10	134.6	137.2	136.8
9a	2.11-2.21[d]	2.14	2.13	11	139.2	141.2	141.2
9b	2.32-2.44[d]	2.37	2.33	12	169.3	168.1	167.6
13a	6.34	6.41	6.34	13	121.3	**124.3**	**124.1**
13b	5.62	5.65	5.60	14	16.9	17.9	18.2
14	1.72	1.73	1.73	15	17.3	18.2	18.3
15	1.31	1.23	1.29				
	RMSD:[e]	0.08	0.02		RMSD:[e]	1.5	1.4
	MD:[e]	0.14	0.04		MD:[e]	3.0	2.8

[a:] Data for method 1 (current study – see Table 7 of the main text for details on the model chemistry)
[b:] Data for method 2 (current study – see Table 7 of the main text for details on the model chemistry)
[c:] Experimental NMR data obtained in $CDCl_3$ on a 300 MHz NMR spectrometer [11].
[d:] Proton chemical shift multiplets were reported as a range, and thus they are not included in RMSD and MD calculations.
[e:] RMSD = root mean square deviation, MD = maximum deviation (largest errors highlighted in blue).

Table S7. Comparison of DFT δ_H and δ_C predictions for **diepoxyguainaolide**

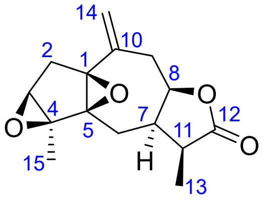

Method:		1[a]	2[b]	Lit[c]	Method:		1[a]	2[b]	Lit[c]
	Expt[d]	DFT	DFT	DFT		Expt[d]	DFT	DFT	DFT
Atom	δ_H (ppm)				Atom	δ_C (ppm)			
2a	2.11	**1.90**	2.08	**1.67**	1	77.2	78.5	78.8	77.6
2b	2.15	2.05	2.11	2.01	2	27.9	27.5	27.4	26.9
3	3.50	3.35	3.45	3.32	3	66.7	67.1	67.8	65.8
6a	2.35	2.32	2.32	2.20	4	61.3	62.0	62.7	60.9
6b	1.79	1.71	1.81	1.83	5	67.5	69.4	69.9	68.4
7	2.55	2.44	2.46	2.27	6	21.0	23.7	23.1	23.4
8	4.36	4.16	4.34	4.22	7	41.1	42.2	42.0	42.4
9a	2.60	2.51	2.57	2.62	8	79.5	79.3	80.4	78.7
9b	2.40	2.30	**2.52**	2.55	9	32.2	33.6	33.5	33.3
11	2.88	2.73	2.88	2.59	10	139.1	**143.2**	**143.2**	**141.7**
13	1.25	1.21	1.25	1.25	11	38.8	40.7	40.4	39.8
14a	5.30	5.35	5.27	5.41	12	177.5	176.3	175.7	175.7
14b	5.26	5.21	5.24	5.29	13	10.2	12.0	11.9	11.2
15	1.57	1.51	1.50	1.48	14	119.1	119.6	119.7	117.6
					15	16.3	17.2	17.2	16.8
	RMSD:[e]	0.12	0.05	0.18		RMSD:[e]	1.7	1.8	1.3
	MD:[e]	0.21	0.12	0.44		MD:[e]	4.1	4.1	2.6

[a:] Data for method 1 (current study – see Table 7 of the main text for details on the model chemistry)
[b:] Data for method 2 (current study – see Table 7 of the main text for details on the model chemistry)
[c:] Calculated chemical shifts from ref. [12] using parametric/DFT hybrid method, DU8+, which is wB97X-D/6-31G(d)//B3LYP/6-31G(d) calculations and conversion of shieldings to chemical shifts according to ref. [13].
[d:] Experimental NMR data from ref. [14] at 400 MHz / 100 MHz (^{1}H / ^{13}C) in $CDCl_3$; chemical shift referencing relative to TMS.
[e:] RMSD = root mean square deviation, MD = maximum deviation (largest errors highlighted in blue).

Table S8. Comparison of DFT δ_H and δ_C predictions for **cannabicitran (CBT-C)**

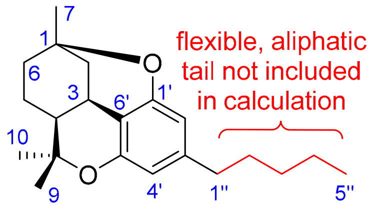

	Method: Expt[d]	1[a] DFT	2[b] DFT	Lit.[c] DFT		Method: Expt[d]	1[a] DFT	2[b] DFT	Lit.[c] DFT
Atom	δ_H (ppm)				Atom	δ_C (ppm)			
2 eq	2.23	2.10	2.21	2.17	1	74.4	73.8	74.7	73.7
2 ax	1.83	1.82	1.84	1.71	2	35.4	35.6	35.5	34.3
3	2.86	2.73	2.84	2.78	3	28.1	**30.3**	**30.3**	28.5
4	2.03	2.20	2.16	1.93	4	46.8	48.0	47.9	47.2
5 eq	1.24	1.14	1.19	1.13	5	22.1	23.2	22.9	22.7
5 ax	0.61	0.52	0.56	**0.92**	6	37.3	37.2	37.0	37.3
6 eq	1.77	1.67	1.71	1.71	7	29.1	30.2	30.0	27.2
6 ax	1.42	1.41	1.48	1.37	8	83.5	83.3	84.5	83.2
7	1.38	1.33	1.35	1.26	9	23.7	25.0	24.8	**21.7**
9	1.02	1.00	1.00	0.98	10	29.7	31.1	30.7	**27.7**
10	1.53	1.47	1.47	1.39	1’	156.5	156.7	157.8	156.8
2’	6.33	**6.16**	6.28	6.31	2’	108.9	109.0	109.0	109.4
4’	6.29	6.14	**6.12**	6.31	4’	109.7	109.8	110.0	109.9
					5’	156.9	157.5	157.8	158.0
					6’	114.0	114.1	114.4	112.8
	RMSD:[e]	0.11	0.07	0.12		RMSD:[e]	0.9	1.0	1.1
	MD:[e]	0.17	0.17	0.31		MD:[e]	2.2	2.2	2.0

a: Data for method 1 (current study – see Table 7 of the main text for details on the model chemistry).
b: Data for method 2 (current study – see Table 7 of the main text for details on the model chemistry).
b: Calculated results by Wood, *et. al.* [15]. The best performance for δ_H was obtained from NMR calculations *in vacuu* at the B3LYP/6-31+G(d,p) level, while the best performance for δ_C was provided from NMR calculations *in vacuu* at the mPW1PW91/6-311+G(d,p) level. B3LYP/6-31+G(d,p) was used for geometry optimizations in the gas phase for both δ_H and δ_C. Linear scaling factors were used to convert isotropic magnetic shielding values, σ_H and σ_C, to chemical shifts, δ_H and δ_C, using the scaling factors provided by Lodewyk, *et. al.* [16].
d: Experimental data obtained at 25°C on a 600 MHz NMR spectrometer from Ref [15] for a 2.0 mg sample dissolved in 0.15 mL $CDCl_3$.
e: RMSD = root mean square deviation, MD = maximum deviation (largest errors highlighted in blue).

Table S9. Comparison of DFT δ_H and δ_C predictions for **ingenane diterpene 8**

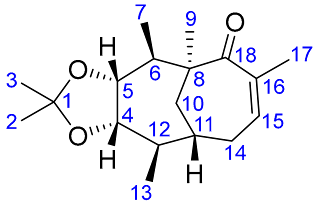

Method: Expt[d]		1[a] DFT	2[b] DFT	Lit[c] DFT	Method: Expt[d]		1[a] DFT	2[b] DFT	Lit[c] DFT
Atom		δ_H (ppm)			Atom		δ_C (ppm)		
2	1.48 (s, 3H)	1.43	1.47	1.49	1	106.8	107.5	107.7	106.9
3	1.34 (s, 3H)	1.27	1.33	1.23	2	28.5[e]	29.3	29.2	24.6
4	3.95 (t, 4.5 Hz, 1H)	3.68	3.87	3.95	3	25.3[e]	26.2	26.2	24.2
5	4.10 (dd, 8.5, 5.0 Hz, 1H)	4.04	4.14	**3.90**	4	87.2	84.2	85.7	86.8
6	2.25 (m, 1H)	2.19	2.22	2.15	5	84.0	84.5	83.9	83.6
7	1.39 (d, 7.5 Hz, 3H)	1.29	1.40	1.35	6	47.1[e]	46.6	47.2	51.6
9	1.09 (s, 3H)	1.25	1.18	1.03	7	15.9	19.2	17.9	13.5
10	1.74[e,f] (m, 2H)	1.83[f]	1.89[f]	-	8	55.3	56.8	56.5	**59.2**
11	1.27[e] (m, 2H)	1.24	1.22	-	9	28.5[e]	30.7	29.1	27.2
12	1.86[e] (m, 1H)	1.72	1.90	1.86	10	42.9[e]	44.3	44.7	44.1
13	1.01 (d, 7.0 Hz, 3H)	0.96	1.00	0.98	11	41.4[e]	39.8	41.0	41.6
14	1.93[e,f] (m, 2H)	2.11[f]	2.13[f]	1.89[f]	12	46.0[e]	45.5	43.7	45.3
15	6.06 (m, 1H)	**6.45**	**6.20**	6.04	13	18.9	19.5	19.8	17.6
17	1.89 (s, 3H)	1.92	1.96	1.92	14	36.0	37.2	36.7	37.4
					15	138.0	**142.5**	140.2	141.7
					16	135.3	139.5	**140.0**	138.5
					17	23.5[e]	26.2	25.7	23.5
					18	211.3	214.5	214.4	211.8
	RMSD:[f]	0.17	0.06	0.08		RMSD:[f]	2.7	2.2	2.2
	MD:[f]	0.39	0.14	0.20		MD:[f]	4.5	4.7	3.9

a: Data for method 1 (current study – see Table 7 of the main text for details on the model chemistry)
b: Data for method 2 (current study – see Table 7 of the main text for details on the model chemistry)
c: Calculated chemical shifts from [4]. NMR calculations (both δ_H and δ_C) at the mPW1PW91/6-311+G(2d,p) level with CPCM/UAKS solvent model for chloroform on B3LYP/6-31+G(d,p) geometries. Linear scaling factors were used to convert isotropic magnetic shielding values, σ_H and σ_C, to chemical shifts, δ_H and δ_C, using the formulas, $\delta_H = (31.8486 - \sigma_H)/1.08225$ and $\delta_C = (186.060 - \sigma_C)/1.04478$.
d: Experimental ^{1}H and ^{13}C NMR data in ppm for a sample dissolved in $CDCl_3$ [17].
e: Assignments are ambiguous and thus not included in RMSD and MD calculations.
f: CH_2 ^{1}H chemical shifts were averaged in experimental assignments; the same was done with DFT predictions [4].
g: RMSD = root mean square deviation, MD = maximum deviation (largest errors highlighted in blue).

Table S10. Comparison of DFT δ_H and δ_C predictions for **artemisinin**

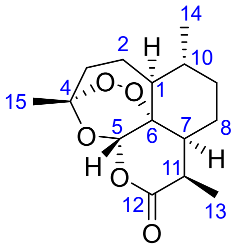

	Method: Expt[d]	1[a] DFT	2[b] DFT		Method: Expt[d]	1[a] DFT	2[b] DFT	Lit.[c] DFT
Atom	δ_H (ppm)			Atom	δ_C (ppm)			
1	1.40	1.47	1.57	1	50.3	50.5	50.1	-
2α	2.07	1.84	**1.83**	2	25.0	26.0	25.8	-
2β	1.45	1.46	1.67	3	36.1	35.9	35.4	-
3α	2.44	2.39	2.47	4	105.3	105.0	105.9	106.3
3β	2.03	2.03	2.08	5	93.8	92.5	93.7	93.5
5	5.86	**5.61**	5.75	6	79.6	**81.7**	**81.9**	**81.0**
7	1.78	1.82	1.83	7	45.1	46.0	45.7	46.0
8α	1.87	1.85	1.77	8	23.4	24.1	24.0	-
8β	1.09	1.04	1.22	9	33.8	33.8	33.7	-
9α	1.78	1.66	1.62	10	37.6	37.6	37.6	-
9β	1.09	1.05	1.09	11	33.0	34.7	34.3	32.5
10	1.45	1.38	1.50	12	171.5	170.5	170.2	171.9
11	3.40	3.27	3.34	13	12.5	14.5	14.5	-
13	1.21	1.19	1.20	14	19.8	21.2	21.2	-
14	1.00	0.96	1.04	15	25.2	25.8	25.4	-
15	1.45	1.39	1.46					
	RMSD:[e]	0.10	0.12		RMSD:[e]	1.1	1.1	0.8
	MD:[e]	0.25	0.24		MD:[e]	2.1	2.3	1.4

[a:] Data for method 1 (current study – see Table 7 of the main text for details on the model chemistry).
[b:] Data for method 2 (current study – see Table 7 of the main text for details on the model chemistry).
[c:] Calculated data from Ref [18]. NMR calculations (δ_C only) using CSGT method at the B3LYP/6-311+G(2d,p) level on B3LYP/6-31G(d,p) geometries. Isotropic shielding values were converted to chemical shifts by subtracting the shielding from the calculated value for TMS. Note that only six δ_C values were reported.
[d:] Experimental data acquired on a 400 MHz NMR spectrometer at 25°C in $CDCl_3$ for 0.14 M and 1 M samples for ^1H and ^{13}C, respectively [19].
[e:] RMSD = root mean square deviation, MD = maximum deviation (largest errors highlighted in blue).

Table S11. Comparison of DFT δ_H and δ_C predictions for **nobilisitine A**

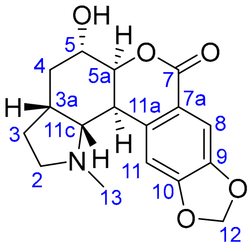

	Method: Expt[d]	1[a] DFT	2[b] DFT	Lit[c] DFT		Method: Expt[d]	1[a] DFT	2[b] DFT	Lit[c] DFT
Atom	δ_H (ppm)				Atom	δ_C (ppm)			
2α	3.24	3.10	3.09	**2.59**	2	54.9	53.3	53.4	54.3
2β	2.30	2.40	2.46	2.78	3	30.1	32.2	32.1	31.3
3α	2.00	1.93	2.00	2.22	3a	34.7	35.3	35.3	36.9
3β	1.62	1.79	1.78	1.64	4	33.7	31.8	31.4	30.8
3a	2.27	2.11	2.21	2.14	5	68.6	67.5	67.8	67.9
4α	1.62	1.54	1.66	1.88	5a	81.4	78.2	79.1	79.1
4β	2.02	2.17	2.17	1.84	7	164.0	163.1	162.8	162.6
5	3.96	4.00	4.03	3.88	7a	118.6	119.1	118.5	118.1
5a	4.65	4.52	4.67	4.37	8	109.7	110.8	110.7	109.6
8	7.53	7.42	7.38	7.35	9	147.2	146.0	146.2	146.2
11	7.05	**6.80**	6.85	6.82	10	152.6	152.0	152.1	151.9
11b	3.34	3.13	**3.09**	3.15	11	106.5	107.5	107.3	105.9
11c	2.67	2.51	2.61	2.72	11a	137.3	**140.9**	**140.8**	**140.4**
12α	6.05	6.16	6.11	6.01	11b	36.6	38.7	38.8	38.8
12β	6.05	6.11	6.11	5.77	11c	66.6	67.8	67.7	65.8
13	2.24	2.26	2.19	2.10	12	102.0	101.1	102.3	101.3
					13	41.8	41.0	41.3	39.7
	RMSD:[e]	0.14	0.12	0.27		RMSD:[e]	1.7	1.5	1.6
	MD:[e]	0.25	0.25	0.65		MD:[e]	3.6	3.5	3.1

a: Data for method 1 (current study – see Table 7 of the main text for details on the model chemistry)
b: Data for method 2 (current study – see Table 7 of the main text for details on the model chemistry)
c: Calculated GIAO chemical shifts from Ref [20] at the mPW1PW91/6-311+G(d,p) level using a CPCM/UAKS solvent model for chloroform on B3LYP/6-31+G(d,p) optimized geometries. Linear scaling factors were used to convert isotropic magnetic shielding values, σ_H and σ_C, to chemical shifts, δ_H and δ_C, using the formulas, $\delta_H = (31.8486 - \sigma_H)/1.08225$ and $\delta_C = (186.060 - \sigma_C)/1.04478$.
d: Experimental ^{1}H and ^{13}C NMR data in $CDCl_3$ referenced to TMS at an unspecified field strength from Ref [21].
e: RMSD = root mean square deviation, MD = maximum deviation (largest errors highlighted in blue).

Table S12. Comparison of DFT δ_H and δ_C predictions for **intricarene**

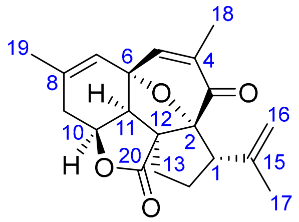

	Method: Expt[d]	1[a] DFT	2[b] DFT	Lit[c] DFT		Method: Expt[d]	1[a] DFT	2[b] DFT	Lit[c] DFT
Atom		δ_H (ppm)			Atom		δ_C (ppm)		
1	3.38	3.47	3.46	3.44	1	46.4	47.3	47.3	48.4
5	6.27	6.40	**6.48**	6.32	2	102.8	105.6	105.3	105.9
7	6.41	6.34	6.36	6.32	3	193.0	197.0	196.4	192.3
9a	2.82	2.79	2.79	2.77	4	137.2	138.9	138.8	138.3
9b	2.41	2.20	2.43	2.27	5	147.3	150.9	150.6	150.3
10	4.77	**4.53**	4.68	4.55	6	84.6	85.6	85.8	85.3
11	2.54	2.52	2.65	2.70	7	128.1	130.8	130.0	129.5
13a	2.01	2.10	2.07	2.02	8	135.2	**139.2**	**139.0**	**140.1**
13b	1.92	1.88	2.04	1.83	9	35.7	36.1	35.7	36.3
14a	2.11	2.13	2.09	2.10	10	70.7	70.4	71.8	69.8
14b	1.91	1.92	2.00	1.86	11	58.0	60.0	59.5	59.5
16a	4.92	4.94	4.84	4.94	12	64.2	66.8	66.4	66.3
16b	4.86	4.88	4.91	**5.13**	13	29.3	31.0	30.8	31.8
17	1.75	1.77	1.80	1.81	14	28.5	30.2	29.4	29.8
18	1.76	1.72	1.72	1.62	15	141.8	145.1	145.4	146.0
19	1.84	1.87	1.92	1.87	16	113.3	114.6	113.6	116.3
					17	23.1	23.9	23.7	21.6
					18	14.4	17.2	17.2	15.5
					19	23.0	23.9	23.8	23.1
					20	177.9	178.4	177.5	178.4
	RMSD:[e]	0.10	0.09	0.12		RMSD:[e]	2.3	2.1	2.2
	MD:[e]	0.24	0.21	0.27		MD:[e]	4.0	3.8	4.9

a: Data for method 1 (current study – see Table 7 of the main text for details on the model chemistry)

b: Data for method 2 (current study – see Table 7 of the main text for details on the model chemistry)

c: Calculated chemical shifts from Ref [4]. NMR calculations (both δ_H and δ_C) at the mPW1PW91/6-311+G(2d,p) level with CPCM/UAKS solvent model for chloroform on B3LYP/6-31+G(d,p) geometries. Linear scaling factors were used to convert isotropic magnetic shielding values, σ_H and σ_C, to chemical shifts, δ_H and δ_C, using the formulas, $\delta_H = (31.8486 - \sigma_H)/1.08225$ and $\delta_C = (186.060 - \sigma_C)/1.04478$.

d: Experimental ^{1}H and ^{13}C NMR were collected at 25°C in $CDCl_3$ using a 500 MHz NMR spectrometer and referenced to TMS [22].

e: RMSD = root mean square deviation, MD = maximum deviation (largest errors highlighted in blue).

Table S13. Comparison of DFT δ_H and δ_C predictions for **strychnine**

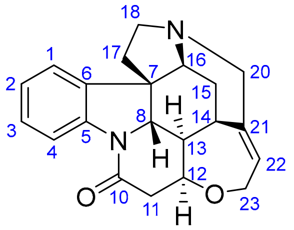

	Method: Expt[e]	1[a] DFT	2[b] DFT	Lit. 1[c] DFT	Lit. 2[d] DFT		Method: Expt[e]	1[a] DFT	2[b] DFT	Lit. 1[c] DFT	Lit. 2[d] DFT
Atom	δ_H (ppm)					Atom	δ_C (ppm)				
1	7.16	7.26	7.16	7.23	7.17	1	122.8	122.8	122.6	126.0	121.6
2	7.08	7.03	6.98	7.19	7.06	2	124.4	122.5	122.7	127.2	122.4
3	7.24	7.17	7.20	7.41	7.22	3	128.8	127.9	128.0	133.6	127.1
4	8.08	8.16	8.01	**8.60**	8.12	4	116.3	115.3	115.4	121.4	115.8
8	3.85	3.68	3.98	3.85	3.83	5	142.2	143.5	143.2	149.3	141.6
11a	3.11	2.92	3.01	3.01	3.02	6	132.9	134.0	134.3	138.0	133.6
11b	2.66	2.49	2.68	2.62	2.67	7	52.3	54.3	53.8	56.4	52.2
12	4.27	4.05	4.21	4.13	4.15	8	60.1	62.3	62.5	63.0	61.2
13	1.26	1.26	1.23	1.00	1.21	10	170.3	169.6	169.5	172.0	168.4
14	3.13	**2.72**	3.14	2.95	3.24	11	42.8	43.5	43.1	45.6	42.9
15a	1.44	1.28	1.41	1.34	1.49	12	77.6	77.0	77.1	81.6	77.1
15b	2.35	2.21	**2.10**	2.24	2.38	13	48.2	48.9	48.3	51.6	48.0
16	3.96	3.85	3.86	3.93	3.89	14	31.7	33.4	33.2	36.2	33.0
17a	1.89	1.93	1.84	1.87	2.00	15	26.9	28.6	28.4	30.0	27.1
17b	1.87	1.92	1.72	1.78	1.91	16	60.1	61.0	60.9	64.1	60.7
18a	3.21	3.07	3.01	3.09	**3.03**	17	42.7	44.4	44.3	46.1	42.8
18b	2.87	2.79	2.89	2.92	2.99	18	50.3	49.4	49.5	51.7	49.8
20a	3.71	3.64	3.65	3.75	3.65	20	52.9	51.7	51.8	54.8	51.7
20b	2.74	2.61	2.58	2.53	2.76	21	140.0	**144.0**	**143.8**	**151.4**	**146.7**
22	5.92	5.86	5.85	6.10	6.08	22	127.9	128.0	127.9	134.0	128.9
23a	4.14	4.03	4.16	4.10	4.02	23	64.8	63.5	63.9	67.3	63.3
23b	4.05	3.97	3.98	4.14	4.11						
	RMSD:[f]	0.15	0.10	0.16	0.08		RMSD:[f]	1.5	1.4	4.6	1.8
	MD:[f]	0.41	0.25	0.52	0.18		MD:[f]	4.0	3.8	11.4	6.7

a: Data for method 1 (current study – see Table 7 of the main text for details on the model chemistry)

b: Data for method 2 (current study – see Table 7 of the main text for details on the model chemistry)

c: Data for literature method 1 reported by Ref [23]. The best δ_H and δ_C methods were B3LYP/cc-pVTZ and PBE0/cc-pVTZ, respectively, both with PCM solvation. Geometries were optimized at B3LYP/6-31G(d,p). Isotropic shielding values were converted to chemical shifts by subtracting from the calculated shielding values for TMS.

d: Data for literature method 2 reported by Semenov and Krivdin [24] with PCM solvation model. Geometry optimized at MP2/aug-cc-pVTZ, including solvent effects for chloroform from PCM. Calculated isotropic shielding values were converted to chemical shifts using the calculated value for TMS and then linearly scaled according to $\delta_{recalc} = (\delta_{calc} – b) / a$, where a and b were obtained via regression analysis.

e: Experimental data from Ref [25] performed on a 500 MHz NMR in $CDCl_3$.

f: RMSD = root mean square deviation, MD = maximum deviation (largest errors highlighted in blue).

Table S14. Comparison of DFT δ_H and δ_C predictions for **holstiine**

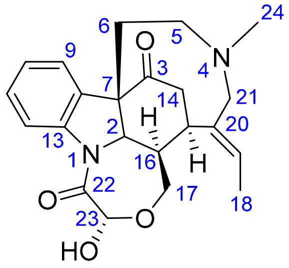

	Method: Expt[d]	1[a] DFT	2[b] DFT	Lit[c] DFT		Method: Expt[d]	1[a] DFT	2[b] DFT	Lit[c] DFT
Atom	δ_H (ppm)				Atom	δ_C (ppm)			
2	4.84	4.58	4.90	**5.31**	2	67.2	68.6	69.0	68.5
5a	2.80	2.56	**2.57**	2.50	3	191.0	**198.7**	**196.1**	192.0
5b	2.66	2.55	2.64	2.72	5	53.5	53.2	52.4	52.9
6a	3.10	3.13	3.11	3.19	6	45.8	47.1	47.1	47.1
6b	1.95	1.87	1.93	1.83	7	55.8	59.2	58.8	57.8
9	7.52	7.55	7.48	7.45	8	132.7	134.0	134.3	134.4
10	7.14	6.99	7.07	7.09	9	124.9	124.8	124.8	124.8
11	7.31	7.15	7.22	7.23	10	125.4	124.0	124.1	124.6
12	8.22	8.39	8.16	8.19	11	128.8	128.4	128.4	128.0
14a	2.49	2.53	2.56	2.41	12	116.9	116.5	116.4	116.8
14b	2.27	2.30	2.38	2.25	13	139.9	140.9	140.8	140.1
15	3.21	3.10	3.16	3.09	14	43.5	45.7	44.9	45.0
16	2.33	2.28	2.32	2.45	15	34.1	35.8	35.4	37.5
17a	4.15	3.88	3.93	4.06	16	42.4	43.5	42.5	43.8
17b	3.85	3.60	3.83	3.41	17	74.3	72.1	72.4	**63.0**
18	1.61	1.68	1.78	1.72	18	13.0	14.7	14.6	14.6
19	5.51	5.48	5.40	5.75	19	125.8	129.3	128.9	128.3
21a	3.56	3.37	3.46	3.33	20	135.6	138.5	138.0	139.3
21b	3.42	3.23	3.52	3.85	21	69.4	68.7	68.2	70.2
23	5.34	**5.04**	5.33	5.41	22	167.9	170.8	170.0	165.7
24	1.91	1.91	2.05	1.92	23	92.8	92.9	93.2	94.1
					24	40.0	38.5	38.7	38.0
	RMSD:[e]	0.16	0.10	0.21		RMSD:[e]	2.4	1.9	2.9
	MD:[e]	0.30	0.23	0.47		MD:[e]	7.7	5.1	11.3

[a:] Data for method 1 (current study – see Table 7 of the main text for details on the model chemistry)
[b:] Data for method 2 (current study – see Table 7 of the main text for details on the model chemistry)
[c:] Calculated chemical shifts at the PBE0/pcSseg-2//pcseg-2 level using linear scaling factors [26].
[d:] Experimental data from a 30 mg sample dissolved in 0.5 mL $CDCl_3$ acquired on a 300 MHz NMR spectrometer [27].
[e:] RMSD = root mean square deviation, MD = maximum deviation (largest errors highlighted in blue).

Table S15. Comparison of DFT δ_H and δ_C predictions for **colchicine**

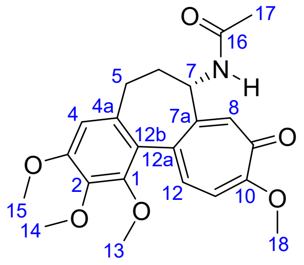

Atom	Method: Expt[e]	1[a] DFT	2[b] DFT	Lit[c] DFT	Lit[d] DFT
	δ_H (ppm)				
4	6.52	6.49	6.56	6.41	6.37
5α	2.52	2.48	2.57	2.44	2.39
5β	2.41	2.34	2.43	2.38	2.37
6α	2.22	2.13	2.24	2.13	2.12
6β	1.76	1.81	1.89	1.69	1.65
7	4.63	4.58	4.46	4.48	4.48
8	7.34	**7.13**	7.15	7.19	7.17
11	6.79	6.68	6.83	6.56	**6.51**
12	7.27	7.08	7.13	7.06	7.04
13	3.63	3.58	3.51	3.56	3.60
14	3.93	3.85	3.84	**3.68**	3.72
15	3.89	3.87	3.86	3.70	3.75
17	1.99	1.86	**1.79**	1.78	1.76
18	3.98	4.01	3.99	3.75	3.78
	RMSD:[f]	0.10	0.11	0.16	0.17
	MD:[f]	0.21	0.20	0.25	0.28

Atom	Method: Expt[e]	1[a] DFT	2[b] DFT	Lit[c] DFT	Lit[d] DFT
	δ_C (ppm)				
1	151.2	153.5	153.7	150.4	151.1
2	141.6	142.9	143.5	140.5	141.3
3	153.6	154.2	154.4	152.7	153.4
4	107.3	109.0	110.1	104.4	103.9
4a	134.2	136.8	136.3	135.4	136.1
5	29.9	30.9	30.4	31.0	31.6
6	36.5	38.4	37.7	38.9	40.1
7	52.6	52.5	54.4	52.1	52.8
7a	152.3	153.6	153.1	150.6	151.1
8	130.5	133.6	**134.5**	130.9	130.4
9	179.5	179.0	179.9	**174.5**	**174.4**
10	163.8	166.7	167.6	164.2	164.8
11	112.8	112.8	115.2	109.0	108.6
12	135.6	**139.4**	138.7	136.6	136.4
12a	136.9	136.5	136.5	135.6	136.4
12b	125.6	129.2	129.6	126.4	127.1
13	61.6	59.1	59.5	58.5	58.6
14	61.4	58.3	59.3	58.0	58.2
15	56.1	54.4	55.8	52.9	53.1
16	170.1	167.0	166.9	166.4	166.7
17	22.8	23.4	23.3	21.9	21.4
18	56.4	55.0	56.1	53.3	53.5
	RMSD:[f]	2.1	2.2	2.3	2.4
	MD:[f]	3.8	4.0	5.0	5.1

a: Data for method 1 (current study – see Table 7 of the main text for details on the model chemistry)

b: Data for method 2 (current study – see Table 7 of the main text for details on the model chemistry)

c: Calculated GIAO chemical shifts from Ref [28] at the mPW1PW91/6-311+G(2d,p) level using optimized geometries at the B3LYP/6-311+G(2d,p) level. A PCM solvent model for chloroform was used for both NMR and geometry optimizations. Gibbs free energies were calculated at the B3LYP/6-311+G(d,p) level for Boltzmann populations. Linear scaling factors were used to convert isotropic magnetic shielding values, σ_H and σ_C, to chemical shifts, δ_H and δ_C, using the formulas, $\delta_H = (31.873 - \sigma_H)/1.072$ and $\delta_C = (186.357 - \sigma_C)/1.042$.

d: Calculated GIAO chemical shifts from Ref [28] at the B3LYP/6-311+G(2d,p) level using optimized geometries at the B3LYP/6-311+G(2d,p) level. A PCM solvent model for chloroform was used for both NMR and geometry optimizations. Gibbs free energies were calculated at the B3LYP/6-311+G(d,p) level for Boltzmann populations. Linear scaling factors were used to convert isotropic magnetic shielding values, σ_H and σ_C, to chemical shifts, δ_H and δ_C, using the formulas, $\delta_H = (31.934 - \sigma_H)/1.056$ and $\delta_C = (181.717 - \sigma_C)/1.043$.

e: Experimental ^{1}H and ^{13}C NMR data for 0.46 mM and 46 mM samples, respectively, in $CDCl_3$ [28].

f: RMSD = root mean square deviation, MD = maximum deviation (largest errors highlighted in blue).

Table S16. Comparison of DFT δ_H and δ_C predictions for **hexacyclinol**

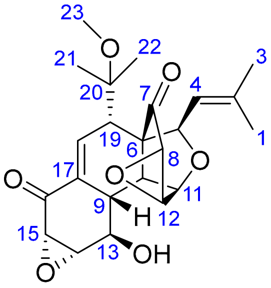

	Method: Expt[f]	1[a] DFT	2[b] DFT	Lit[c] DFT		Method: Expt[f]	1[a] DFT	2[b] DFT	Lit[d] DFT	Lit[c] DFT	Lit[e] ML
Atom	δ_H (ppm)				Atom	δ_C (ppm)					
1	1.79	1.82	1.86	1.91	1	18.6	19.3	18.9	15.4	19.7	22.8
3	1.73	1.74	1.77	1.70	2	142.2	142.9	143.1	138.7	143.6	135.9
4	4.85	4.92	4.87	5.22	3	26.1	27.0	27.0	25.7	29.1	22.8
5	5.49	5.43	5.68	5.54	4	120.7	123.3	122.7	120.4	127.1	120.6
8	3.25	3.15	3.24	2.89	5	75.8	77.0	77.2	77.6	82.8	78.1
9	3.63	3.35	3.65	3.39	6	60.5	63.7	62.7	60.5	65.8	57.0
10	2.76	2.63	2.92	2.50	7	202.9	**209.9**	**208.8**	206.0	210.1	199.3
11	5.01	4.94	4.91	5.06	8	53.1	54.1	54.1	54.4	56.5	53.7
12	3.66	3.59	3.68	3.98	9	40.4	43.0	42.4	37.6	43.5	39.2
13	3.84	3.77	3.93	3.72	10	47.8	48.8	48.7	47.6	51.5	46.3
14	3.53	3.56	3.63	3.15	11	71.5	71.6	71.4	71.8	76.8	76.9
15	3.32	3.18	3.27	3.01	12	54.5	56.0	56.4	**59.7**	58.4	52.8
18	6.76	7.04	7.00	**7.38**	13	72.7	71.9	72.2	76.4	78.7	74.0
19	3.61	**3.99**	**3.91**	4.10	14	61.0	61.5	61.7	63.6	64.3	58.3
21	1.28	1.29	1.22	1.25	15	53.2	53.9	54.5	52.8	55.2	**60.8**
22	1.17	1.13	1.31	1.04	16	192.8	195.7	195.9	188.7	195.4	191.0
23	3.04	3.06	3.01	3.04	17	132.5	133.6	133.4	131.4	137.2	134.9
					18	139.6	145.4	144.6	141.2	**148.6**	144.5
					19	40.9	42.2	41.5	39.5	46.5	48.3
					20	77.3	77.6	77.8	79.7	83.1	79.4
					21	26.6	27.6	27.4	25.6	24.4	24.9
					22	24.7	26.0	26.1	23.9	25.6	24.9
					23	49.1	48.5	48.6	48.0	49.8	50.0
	RMSD:[g]	0.15	0.13	0.29		RMSD:[g]	2.4	2.1	2.3	4.6	3.6
	MD:[g]	0.38	0.30	0.62		MD:[g]	7.0	5.9	5.2	9.0	7.6

[a]: Data for method 1 (current study – see Table 7 of the main text for details on the model chemistry)
[b]: Data for method 2 (current study – see Table 7 of the main text for details on the model chemistry)
[c]: Calculated chemical shifts from Saielli and Bagno [29] at the B97-2/cc-pVTZ level using B3LYP/6-31G(d,p) optimized geometries. Isotropic shielding values were converted to chemical shifts by subtracting from the calculated value for TMS.
[d]: Calculated GIAO ^{13}C chemical shifts from Ref [30] at the mPW1PW91/6-31G(d,p) level using HF/3-21G geometries. Isotropic shielding values were converted to chemical shifts, δ_C, by subtracting from the calculated value for TMS, and then δ_C were scaled by linear least squares regression analysis. Only two conformers were evaluated without Boltzmann weighting. The above data is for the best matching conformer.
[e]: Calculated chemical shifts using machine learning / DFT approach, CASCADE reported in Ref [6].
[f]: Experimental ^{1}H and ^{13}C data from Williams, *et. al.* for a 1.2 mg sample in 0.12 mL of $CDCl_3$ acquired on a 900 MHz NMR spectrometer [31].
[g]: RMSD = root mean square deviation, MD = maximum deviation (largest errors highlighted in blue).

Table S17. Comparison of DFT δ_H and δ_C predictions for **homodimericin A**

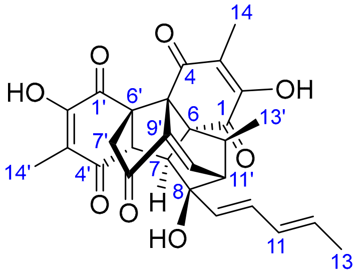

	Method: Expt[d]	1[a] DFT	2[b] DFT		Method: Expt[d]	1[a] DFT	2[b] DFT
Atom	δ_H (ppm)			Atom	δ_C (ppm)		
7	3.26	**3.05**	3.21	1	195.2	202.6	201.4
9	5.90	5.91	5.93	2	154.0	155.3	155.0
10	6.51	**6.72**	**6.70**	3	127.6	127.6	127.7
11	6.07	6.13	6.11	4	194.3	197.8	197.5
12	5.87	5.96	5.93	5	62.8	67.7	67.4
13	1.80	1.88	1.93	6	64.7	66.8	66.3
14	2.08	2.18	2.23	7	59.7	64.2	62.6
5'	3.80	3.83	3.93	8	85.4	86.3	86.7
7'a	2.84	2.70	2.88	9	135.1	137.2	137.0
7'b	2.47	2.38	2.58	10	132.2	132.1	132.2
10'	6.74	6.89	6.75	11	130.3	129.4	129.5
11'	2.71	2.82	2.77	12	132.8	135.9	135.9
12'	2.27	2.28	2.35	13	18.3	19.7	19.6
13'	0.84	0.81	0.86	14	9.4	12.7	12.5
14'	1.94	1.97	2.07	1'	193.4	**201.1**	**199.9**
				2'	155.0	157.3	156.3
				3'	126.0	122.6	123.8
				4'	196.4	200.3	200.0
				5'	55.7	56.8	56.8
				6'	64.3	65.8	65.3
				7'	47.9	50.8	50.1
				8'	197.3	198.5	198.7
				9'	139.2	141.1	140.6
				10'	136.1	142.0	141.4
				11'	52.5	56.1	54.8
				12'	41.1	45.2	44.1
				13'	12.3	13.5	13.4
				14'	9.4	10.1	11.4
	RMSD:[d]	0.10	0.10		RMSD:[d]	3.4	2.9
	MD:[d]	0.21	0.19		MD:[d]	7.7	6.5

[a:] Data for method 1 (current study – see Table 7 of the main text for details on the model chemistry)
[b:] Data for method 2 (current study – see Table 7 of the main text for details on the model chemistry)
[c:] ^1H and ^{13}C NMR data were acquired in this study for a 2 mg synthetic sample dissolved in 0.5 mL $CDCl_3$. Spectra were recorded at 25°C on a 600 MHz NMR spectrometer and referenced to TMS.
[d:] RMSD = root mean square deviation, MD = maximum deviation (largest errors highlighted in blue).

Table S18. Comparison of DFT δH predictions for **strychnobaillonine**

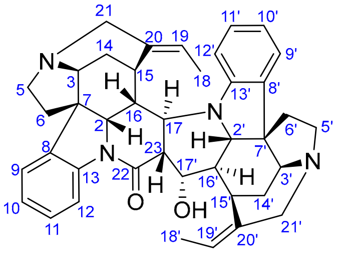

	Method: Expt[d]	1[a] DFT	2[b] DFT	Lit.[c] DFT		Method: Expt[d]	1[a] DFT	2[b] DFT	Lit.[c] DFT
Atom	δH (ppm)				Atom	δH (ppm)			
2	4.45	4.32	4.30	4.33	2’	3.06	3.31	3.38	3.47
3	3.24	3.18	3.03	3.32	3’	3.51	3.29	3.44	3.40
5a	3.18	3.15	3.11	3.39	5a’	3.18	3.02	3.01	3.03
5b	3.03	2.91	2.95	3.01	5b’	2.71	2.63	2.71	2.65
6a	2.46	2.11	2.32	2.17	6a’	2.30	1.49	1.72	1.62
6b	2.14	2.09	2.27	2.04	6b’	(mult.)	2.25	2.40	2.43
9	7.26	7.20	7.22	7.40	9’	7.09	6.97	7.00	7.13
10	7.18	7.09	7.11	7.19	10’	6.85	6.69	6.71	6.83
11	7.29	7.21	7.23	7.29	11’	7.17	6.97	7.00	7.16
12	8.23	8.25	8.09	8.28	12’	6.52	6.61	6.74	6.59
14a	1.72	1.65	1.76	1.81	14a’	2.04	1.88	1.93	1.83
14b	1.73	2.03	1.87	2.05	14b’	1.62	1.45	1.60	1.78
15	2.74	2.99	**3.08**	3.30	15’	3.03	2.87	2.95	3.04
16	2.57	2.56	2.50	2.85	16’	1.61	1.52	1.77	1.67
17	3.86	3.72	3.70	3.86	17’	4.62	4.32	4.34	4.15
18	1.78	1.64	1.78	1.68	18’	1.72	1.75	1.83	1.73
19	5.43	5.36	5.44	5.48	19’	5.62	5.44	5.41	5.69
21a	3.62	3.49	3.31	3.73	21a’	3.59	3.55	3.46	3.45
21b	3.36	**2.90**	3.25	**2.74**	21b’	2.91	2.83	3.06	3.12
23	2.88	2.55	2.72	2.55					
						RMSD[e]:	0.19	0.16	0.22
						MD[e]:	0.46	0.34	0.62

a: Data for method 1 (current study – see Table 7 of the main text for details on the model chemistry)
b: Data for method 2 (current study – see Table 7 of the main text for details on the model chemistry)
c: Data from Semenov and Krivdin [32], PCM-PBE0/pcSseg-2//pcseg-2. Geometries at PCM-B3LYP/aug-cc-pVQZ//cc-pVQZ.
c: Experimental data from A.T. Tchinda, *et. al.* [33].
e: RMSD = Root mean square deviation, MD = maximum deviation (largest errors highlighted in blue).

Table S19. Comparison of DFT δC predictions for **strychnobaillonine**

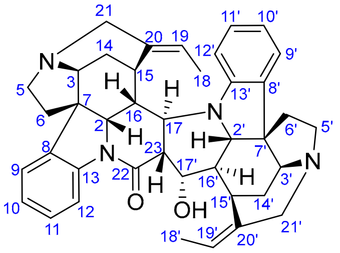

	Method: Expt[d]	1[a] DFT	2[b] DFT	Lit.[c] DFT		Method: Expt[d]	1[a] DFT	2[b] DFT	Lit.[c] DFT
Atom	δC (ppm)				Atom	δC (ppm)			
2	63.7	65.5	67.3	61.2	23	47.8	47.7	48.1	44.7
3	66.3	66.8	68.5	66.7	2'	69.4	67.4	70.3	67.3
5	54.1	55.3	55.3	56.6	3'	61.7	61.1	62.2	61.5
6	36.5	44.4	40.3	43.2	5'	53.7	52.6	53.6	51.6
7	52.8	56.2	56.1	54.8	6'	41.2	41.6	43.4	40.4
8	137.4	140.9	141.0	142.1	7'	51.8	54.7	54.1	54.1
9	122.3	122.8	122.5	121.4	8'	134.3	133.8	135.5	133.9
10	125.3	124.1	124.2	123.8	9'	122.2	123.4	123.0	121.6
11	128.0	127.4	127.8	126.0	10'	119.8	117.7	118.0	116.3
12	117.9	115.7	115.2	115.2	11'	128.2	127.6	127.8	126.1
13	141.4	141.1	140.7	139.1	12'	108.3	107.1	108.3	104.8
14	23.8	24.0	25.1	19.6	13'	150.0	152.6	151.8	150.2
15	29.3	31.6	34.3	28.5	14'	28.5	30.3	30.3	29.4
16	39.0	**49.4**	**45.4**	**45.7**	15'	27.7	31.4	31.2	30.0
17	57.5	56.3	60.1	52.5	16'	47.9	50.2	48.8	48.3
18	13.7	14.7	15.0	12.1	17'	69.5	66.9	67.8	66.6
19	117.7	122.4	121.5	121.0	18'	13.0	15.1	14.7	11.4
20	140.5	142.5	142.6	145.0	19'	122.2	122.8	122.7	121.5
21	52.3	55.0	53.8	57.3	20'	133.8	139.0	138.1	138.3
22	167.7	173.1	171.6	169.0	21'	58.1	56.3	57.4	57.8
						RMSD[e]:	3.0	2.5	2.9
						MD[e]:	10.4	6.4	6.7

[a:] Data for method 1 (current study – see Table 7 of the main text for details on the model chemistry)
[b:] Data for method 2 (current study – see Table 7 of the main text for details on the model chemistry)
[c:] Data from Semenov and Krivdin [32], PCM-PBE0/pcSseg-2//pcseg-2. Geometries at PCM-B3LYP/aug-cc-pVQZ//cc-pVQZ.
[d:] Experimental data from A.T. Tchinda, *et. al.* [33].
[e:] RMSD = Root mean square deviation, MD = maximum deviation (largest errors highlighted in blue).

Table S20. Comparison of DFT δ_H predictions for **sungucine**

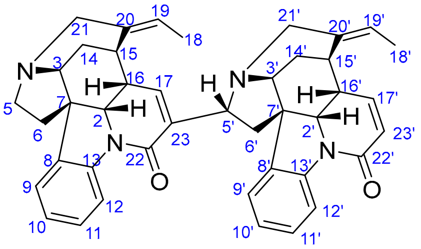

	Method: Expt[d]	1[a] DFT	2[b] DFT	Lit.[c] DFT	ML[e]		Method: Expt[d]	1[a] DFT	2[b] DFT	Lit.[c] DFT	ML[e]
Atom	δ_H (ppm)					Atom	δ_H (ppm)				
2	4.31	3.84	4.14	4.03	4.01	2’	4.38	4.35	4.43	4.23	4.04
3	3.28	3.46	3.47	3.27	3.45	3’	3.65	3.49	3.63	3.58	3.51
5a	3.07	2.96	3.07	3.08	3.37	5’	4.22	4.23	4.29	**3.58**	4.24
5b	3.20	3.10	3.11	3.15	**4.31**	6’a	2.29	2.33	2.42	2.26	2.70
6a	2.16	**1.65**	1.89	1.81	2.21	6’b	2.64	2.70	2.85	2.64	2.88
6b	2.56	2.46	2.53	2.70	3.06	9’	7.30	7.41	7.19	7.21	7.24
9	7.27	7.37	7.45	7.24	7.27	10’	7.09	6.89	6.97	7.07	7.30
10	7.12	6.99	7.10	7.08	7.21	11’	7.29	7.12	7.15	7.23	7.47
11	7.25	6.96	7.16	7.21	7.44	12’	8.28	8.25	8.16	8.25	8.08
12	8.23	8.51	8.13	8.29	8.10	14’a	1.83	1.65	1.73	1.84	1.25
14a	1.81	1.57	1.53	1.79	1.32	14’b	1.86	1.86	2.00	1.91	1.57
14b	1.84	1.92	1.70	1.87	1.53	15’	2.73	2.94	2.70	2.71	2.61
15	2.72	2.57	2.84	2.66	2.39	16’	2.78	2.75	2.70	2.67	2.91
16	2.71	2.33	2.77	2.52	2.82	17’	6.85	6.95	6.93	7.03	7.36
17	7.06	7.21	**7.37**	7.40	7.17	18’	1.65	1.69	1.79	1.88	1.88
18	1.75	1.83	1.85	1.93	1.84	19’	5.21	5.26	5.39	5.51	5.79
19	5.38	5.35	5.32	5.60	5.68	21’a	2.90	2.86	2.93	2.92	3.47
21a	3.25	3.03	3.11	2.99	3.47	21’b	3.64	3.63	3.51	3.61	3.48
21b	3.63	3.72	3.74	3.75	3.59	23’	6.02	5.99	5.92	5.95	6.12
							RMSD:[f]	0.18	0.14	0.18	0.34
							MD:[f]	0.51	0.31	0.64	1.11

a: Data for method 1 (current study – see Table 7 of the main text for details on the model chemistry)
b: Data for method 2 (current study – see Table 7 of the main text for details on the model chemistry)
c: DFT GIAO calculations of ^{1}H and ^{13}C NMR chemical shifts at the mPW1PW91/6-311G(d,p) level on M06-2X/cc-pVTZ//aug-cc-pVTZ geometries (i.e., cc-pVTZ basis set used for H and C while aug-cc-pVTZ was used for O and N). An implicit solvent model (PCM) for chloroform was employed for both geometries and NMR calculations. Isotropic shielding values were converted to chemical shifts by subtracting from the calculated shielding for TMS [34].
d: Experimental data from Frederich, *et. al.* [35] in $CDCl_3$ acquired at 25°C on a 400 MHz NMR spectrometer and referenced to TMS. Single crystal XRD was reported by Lamotte, *et. al.* [36].
e: Results from Machine Learning (ML) / DFT approach that were calculated using an online app at http://nova.chem.colostate.edu/cascade; method details are from Ref [6].
f: RMSD = Root mean square deviation, MD = maximum deviation compared to experiment (largest errors highlighted in blue).

Table S21. Comparison of DFT δC predictions for **sungucine**

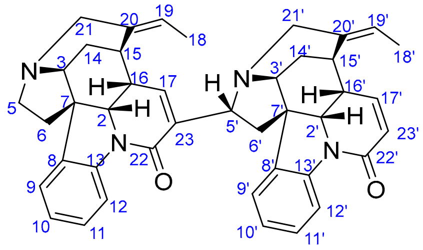

Method:	Expt[d]	1[a] DFT	2[b] DFT	Lit.[c] DFT	ML[e]	Method:	Expt[d]	1[a] DFT	2[b] DFT	Lit.[c] DFT	ML[e]
Atom	δC (ppm)					Atom	δC (ppm)				
2	64.4	67.4	67.3	64.6	65.7	2’	64.9	66.9	66.9	63.8	65.2
3	65.7	64.2	65.1	64.9	64.3	3’	64.3	64.8	65.0	65.3	63.5
5	53.8	52.0	52.5	51.1	48.5	5’	62.1	63.7	63.5	60.4	61.5
6	37.2	38.8	38.2	36.8	36.3	6’	45.6	47.1	46.0	46.1	42.9
7	52.5	54.5	54.1	52.9	55.5	7’	52.7	54.9	54.1	53.0	56.3
8	136.3	136.0	136.8	134.5	136.5	8’	134.2	135.1	135.5	134.2	134.5
9	122.4	122.9	122.8	121.3	123.7	9’	122.5	122.9	122.9	121.2	123.9
10	124.5	122.7	122.9	122.5	122.4	10’	124.1	122.4	122.7	122.6	122.5
11	128.3	127.9	127.9	127.4	129.2	11’	128.5	128.2	128.2	127.6	129.3
12	116.3	115.5	115.6	115.3	116.6	12’	116.5	115.2	115.3	115.3	115.9
13	141.3	143.2	**136.8**	141.4	142.4	13’	141.8	143.4	143.0	141.3	142.0
14	22.5	23.2	22.8	21.1	**30.6**	14’	23.5	23.5	22.9	21.5	30.8
15	31.1	33.3	33.7	33.0	37.4	15’	31.4	33.1	33.4	32.5	38.0
16	37.7	41.8	40.7	41.0	37.4	16’	40.3	42.6	42.2	41.5	39.7
17	137.1	139.9	139.9	138.1	132.7	17’	143.4	146.1	146.1	145.3	144.8
18	13.2	14.6	14.5	13.1	13.9	18’	13.0	14.6	14.5	13.0	14.4
19	119.0	121.4	121.1	122.5	124.3	19’	119.6	121.7	121.6	122.8	123.8
20	140.7	**145.1**	144.8	**146.1**	139.9	20’	142.5	144.7	144.9	145.9	139.8
21	52.5	51.4	51.7	51.9	55.1	21’	50.2	50.0	50.1	49.7	56.1
22	162.5	163.3	162.9	159.6	160.7	22’	161.7	161.8	161.7	158.4	160.6
23	135.1	138.2	136.8	136.3	137.4	23’	123.5	123.9	123.6	123.5	123.9
							RMSD:[f]	1.9	1.8	1.8	3.1
							MD:[f]	4.4	4.5	5.4	8.1

a: Data for method 1 (current study – see Table 7 of the main text for details on the model chemistry)

b: Data for method 2 (current study – see Table 7 of the main text for details on the model chemistry)

c: DFT GIAO calculations of ^{1}H and ^{13}C NMR chemical shifts at the mPW1PW91/6-311G(d,p) level on M06-2X/cc-pVTZ//aug-cc-pVTZ geometries (i.e., cc-pVTZ basis set used for H and C while aug-cc-pVTZ was used for O and N). An implicit solvent model (PCM) for chloroform was employed for both geometries and NMR calculations. Isotropic shielding values were converted to chemical shifts by subtracting from the calculated shielding for TMS [34].

d: Experimental data from Frederich, *et. al.* [35] in $CDCl_3$ acquired at 25°C on a 400 MHz NMR spectrometer and referenced to TMS. Single crystal XRD was reported by Lamotte, *et. al.* [36].

e: Results from Machine Learning (ML) / DFT approach that were calculated using an online app at http://nova.chem.colostate.edu/cascade; method details are from Ref [6].

f: RMSD = Root mean square deviation, MD = maximum deviation compared to experiment (largest errors highlighted in blue).

Table S22. Comparison of DFT δH and δC predictions for **paclitaxel**

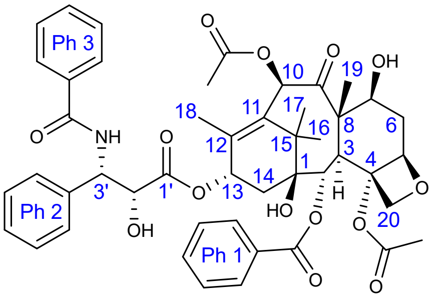

	Method: Expt[d]	1[a] DFT	2[b] DFT		Method: Expt[d]	1[a] DFT	2[b] DFT	Lit[c] DFT
Atom		δH (ppm)		Atom		δC (ppm)		
2	5.67	**5.24**	5.54	1	79.0	81.8	81.1	81.6
3	3.79	3.81	3.85	2	74.9	77.0	76.0	76.3
4 OAc Me	2.38	2.71	2.75	3	45.6	46.9	46.0	43.0
5	4.94	4.66	4.88	4	81.1	82.4	82.5	79.5
6a	2.54	2.48	2.38	4 OAc C=O	170.4	172.9	172.9	168.6
6b	1.88	1.75	1.80	4 OAc Me	22.6	23.8	24.4	23.3
7	4.40	4.31	4.41	5	84.4	82.3	82.4	87.9
10	6.27	6.15	6.30	6	35.6	35.9	35.6	37.0
10 OAc Me	2.23	2.31	2.34	7	72.2	73.7	74.0	77.6
13	6.23	6.22	6.47	8	58.6	61.5	60.6	63.6
14a	2.35	2.40	2.53	9	203.6	210.4	209.1	207.6
14b	2.28	1.99	1.84	10	75.5	78.4	78.1	**84.6**
16	1.14	1.02	1.09	10 OAc C=O	171.2	173.2	172.7	170.8
17	1.24	1.24	1.16	10 OAc Me	20.8	21.7	21.1	21.2
17	1.79	1.76	1.85	11	133.2	135.1	133.2	136.8
19	1.68	1.77	1.76	12	142.0	147.9	147.3	149.2
20a	4.30	4.24	4.34	13	72.3	74.5	73.9	72.4
20b	4.19	3.88	4.23	14	35.7	37.3	36.6	41.6
2'	4.78	4.84	5.09	15	43.2	46.2	45.6	48.7
3'	5.78	5.67	5.93	16	21.8	23.6	23.4	21.6
3' NH	7.01	6.99	7.08	17	26.9	28.4	26.7	23.4

a: Data for method 1 (current study – see Table 7 of the main text for details on the model chemistry)
b: Data for method 2 (current study – see Table 7 of the main text for details on the model chemistry)
c: Calculated ^{13}C chemical shifts from Ref [37]. No calculated ^{1}H NMR data were reported.
d: Experimental data from Ref [38] in $CDCl_3$ on a 500 MHz NMR spectrometer. ^{1}H chemical shifts were referenced to TMS at 0.00 ppm, and ^{13}C chemical shifts were referenced to $CDCl_3$ at 77.00 ppm.

Note: Table continued on next page.

Table S22 (continued). Comparison of DFT δ_H and δ_C predictions for **paclitaxel**

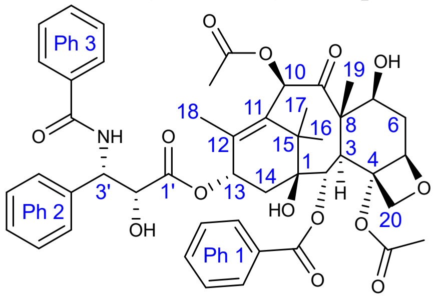

	Method: Expt[d]	1[a] DFT	2[b] DFT
Atom	δ_H (ppm)		
o Ph 1	8.13	8.50	**8.65**
m Ph 1	7.51	7.60	7.49
p Ph 1	7.61	7.71	7.57
o Ph 2	7.48	7.75	7.63
m Ph 2	7.42	7.42	7.40
p Ph 2	7.35	7.40	7.34
o Ph 3	7.74	7.76	7.54
m Ph 3	7.40	7.30	7.10
p Ph 3	7.49	7.43	7.35
	RMSD:[e]	0.17	0.19
	MD:[e]	0.43	0.52

	Method: Expt[d]	1[a] DFT	2[b] DFT	Lit[c] DFT
Atom	δ_C (ppm)			
18	14.8	16.9	17.6	16.8
19	9.5	12.4	12.2	16.1
20	76.5	73.5	73.9	75.5
1'	172.7	176.9	174.8	172.2
2'	73.2	74.4	74.1	81.1
3'	55.0	57.8	56.8	60.1
C=O Ph 1	167.0	169.4	167.1	165.1
q Ph 1	129.1	128.9	128.7	130.5
o Ph 1	130.2	132.4	133.4	130.5
m Ph 1	128.7	127.9	127.7	126.4
p Ph 1	133.7	134.3	134.3	131.7
q Ph 2	133.6	**140.8**	**140.0**	138.5
o Ph 2	127.0	128.5	127.7	127.5
m Ph 2	128.7	128.1	128.2	128.2
p Ph 2	131.9	127.6	127.4	127.1
C=O Ph 3	167.0	168.3	167.9	168.4
q Ph 3	138.0	134.1	134.5	133.5
o Ph 3	127.0	128.2	128.6	127.4
m Ph 3	129.0	127.9	127.6	126.7
p Ph 3	128.3	131.9	131.7	131.0
	RMSD:[e]	2.8	2.4	3.7
	MD:[e]	7.2	6.4	9.1

a: Data for method 1 (current study – see Table 7 of the main text for details on the model chemistry)
b: Data for method 2 (current study – see Table 7 of the main text for details on the model chemistry)
c: Calculated ^{13}C chemical shifts from Ref [37] using GIAO B3LYP/6-311+G(2d,p)//B3LYP/6-31G(d) model chemistry. Linear scaling factors were used to convert isotropic shielding values to chemical shifts. No calculated ^{1}H NMR data were reported.
d: Experimental data from Ref [38] in $CDCl_3$ on a 500 MHz NMR spectrometer. ^{1}H chemical shifts were referenced to TMS at 0.00 ppm, and ^{13}C chemical shifts were referenced to $CDCl_3$ at 77.00 ppm.
e: RMSD = root mean square deviation, MD = maximum deviation; multiplets were not used for RMSD and MD calculations (largest errors highlighted in blue).

Table S23. Comparison of DFT δ$_H$ and δ$_C$ predictions for **sunguicine** versus its 5’-epimer

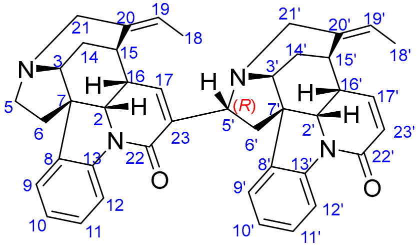

Atom	Expt[a] 5’(*R*)	DFT[b] 5’(*R*)	DFT[b] 5’(*S*)	Atom	Expt[a] 5’(*R*)	DFT[b] 5’(*R*)	DFT[b] 5’(*S*)	Atom	Expt[a] 5’(*R*)	DFT[b] 5’(*R*)	DFT[b] 5’(*S*)	Atom	Expt[a] 5’(*R*)	DFT[b] 5’(*R*)	DFT[b] 5’(*S*)
	δ$_H$ (ppm)				δ$_H$ (ppm)				δ$_C$ (ppm)				δ$_C$ (ppm)		
2	4.31	4.215	4.223	2’	4.38	4.371	4.346	2	64.38	67.29	66.54	2’	64.89	67.25	68.11
3	3.28	3.324	3.306	3’	3.65	3.554	3.191	3	65.71	65.49	65.32	3’	64.29	64.99	67.28
5a	3.07	3.037	3.040	5’	4.22	4.296	4.585	5	53.78	52.50	52.53	5’	62.08	63.38	59.56
5b	3.20	3.082	3.105	6’a	2.29	2.240	2.442	6	37.15	37.99	37.83	6’	45.61	46.47	45.32
6a	2.16	1.934	1.917	6’b	2.64	2.676	2.539	7	52.45	54.30	54.24	7’	52.70	54.18	54.26
6b	2.56	2.596	2.578	9’	7.30	7.190	7.314	8	136.25	136.69	136.34	8’	134.17	135.37	137.81
9	7.27	7.220	7.191	10’	7.09	7.032	7.077	9	122.37	122.93	122.87	9’	122.45	122.81	122.69
10	7.12	7.044	7.078	11’	7.29	7.224	7.213	10	124.46	123.02	123.13	10’	124.10	122.68	122.91
11	7.25	7.198	7.258	12’	8.28	8.196	8.190	11	128.31	128.15	128.29	11’	128.48	128.35	127.92
12	8.23	8.211	8.269	14’a	1.83	1.757	1.692	12	116.29	115.44	115.72	12’	116.54	115.26	115.05
14a	1.81	1.727	1.708	14’b	1.86	1.868	1.890	13	141.33	143.00	143.19	13’	141.76	143.15	142.68
14b	1.84	1.824	1.842	15’	2.73	2.746	2.676	14	22.46	23.04	23.01	14’	23.46	22.88	24.99
15	2.72	2.696	2.797	16’	2.78	2.761	2.899	15	31.11	34.01	33.96	15’	31.37	33.58	33.89
16	2.71	2.621	2.593	17’	6.85	6.938	6.754	16	37.69	40.96	41.66	16’	40.33	42.33	38.28
17	7.06	7.261	6.945	18’	1.65	1.782	1.721	17	137.08	139.86	145.37	17’	143.43	146.16	145.73
18	1.75	1.874	1.847	19’	5.21	5.163	5.211	18	13.17	14.51	14.83	18’	12.98	14.38	14.51
19	5.38	5.351	5.353	21’a	2.90	2.856	3.268	19	119.03	120.91	120.46	19’	119.58	121.98	118.92
21a	3.25	3.050	3.061	21’b	3.64	3.572	3.370	20	140.68	145.06	145.57	20’	142.47	145.24	144.45
21b	3.63	3.587	3.587	23’	6.02	5.939	5.917	21	52.53	51.58	51.35	21’	50.21	50.13	47.02
								22	162.50	162.86	162.06	22’	161.70	161.42	161.57
								23	135.13	137.47	133.69	23’	123.46	123.76	124.09
						RMSD[c]:	0.09	0.15					RMSD[c]:	1.75	2.05
						MD[c]:	0.23	0.46					MD[c]:	4.4	8.3

[a]: Experimental data from Frederich, *et. al.* in $CDCl_3$ [35]. Single crystal XRD from Lamotte, *et. al.* [36].
[b]: DFT calculations using method 2 (see Table 7 of the main text for details on the model chemistry).
[c]: RMSD = Root mean square deviation, MD = maximum deviation compared to experiment.

IV. Homodimericin A NMR Spectra

Homodimericin A NMR spectra were originally acquired for the compound dissolved in DMSO-d_6. For direct comparison to DFT calculations in chloroform, NMR spectra were acquired at 25°C for a 2-3 mg sample of this compound dissolved in 0.5 mL $CDCl_3$, containing 0.03% v/v TMS, using a Bruker 600 MHz AVANCE III HD NMR spectrometer equipped with a 5-mm TXI CryoProbe™. ^{1}H, ^{13}C, ^{1}H-^{1}H gradient COSY, multiplicity-edited ^{1}H-^{13}C gradient HSQC (145 Hz $^1J_{CH}$ optimization), ^{1}H-^{13}C gradient HMBC (8 Hz $^nJ_{CH}$ optimization), and ^{1}H-^{1}H gradient NOESY w/ zero-quantum suppression (500 ms mixing time) spectra were acquired for the purpose of accurately assigning all proton and carbon peaks. TMS at 0.00 ppm was used for ^{1}H and ^{13}C peak referencing. Note that the sample also contains approximately 25% homodimericin B as a synthetic impurity. Spectra were processed in Mestrelab Research's MestReNova, version 14.2.

1H 1D (599.90 MHz, CDCl3, 298.0 K) — homodimericin A

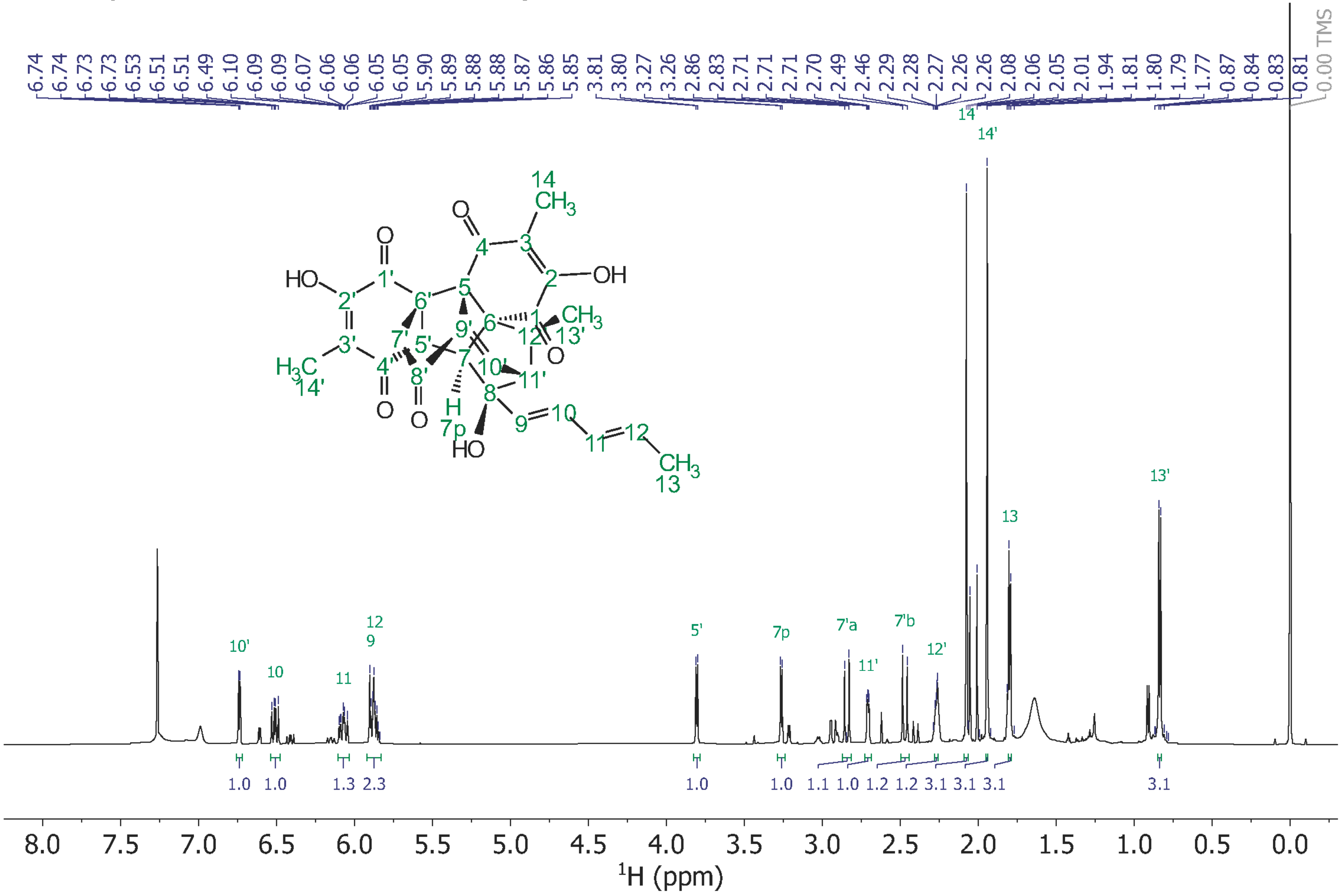

13C 1D (150.86 MHz, CDCl3, 298.0 K) — homodimericin A

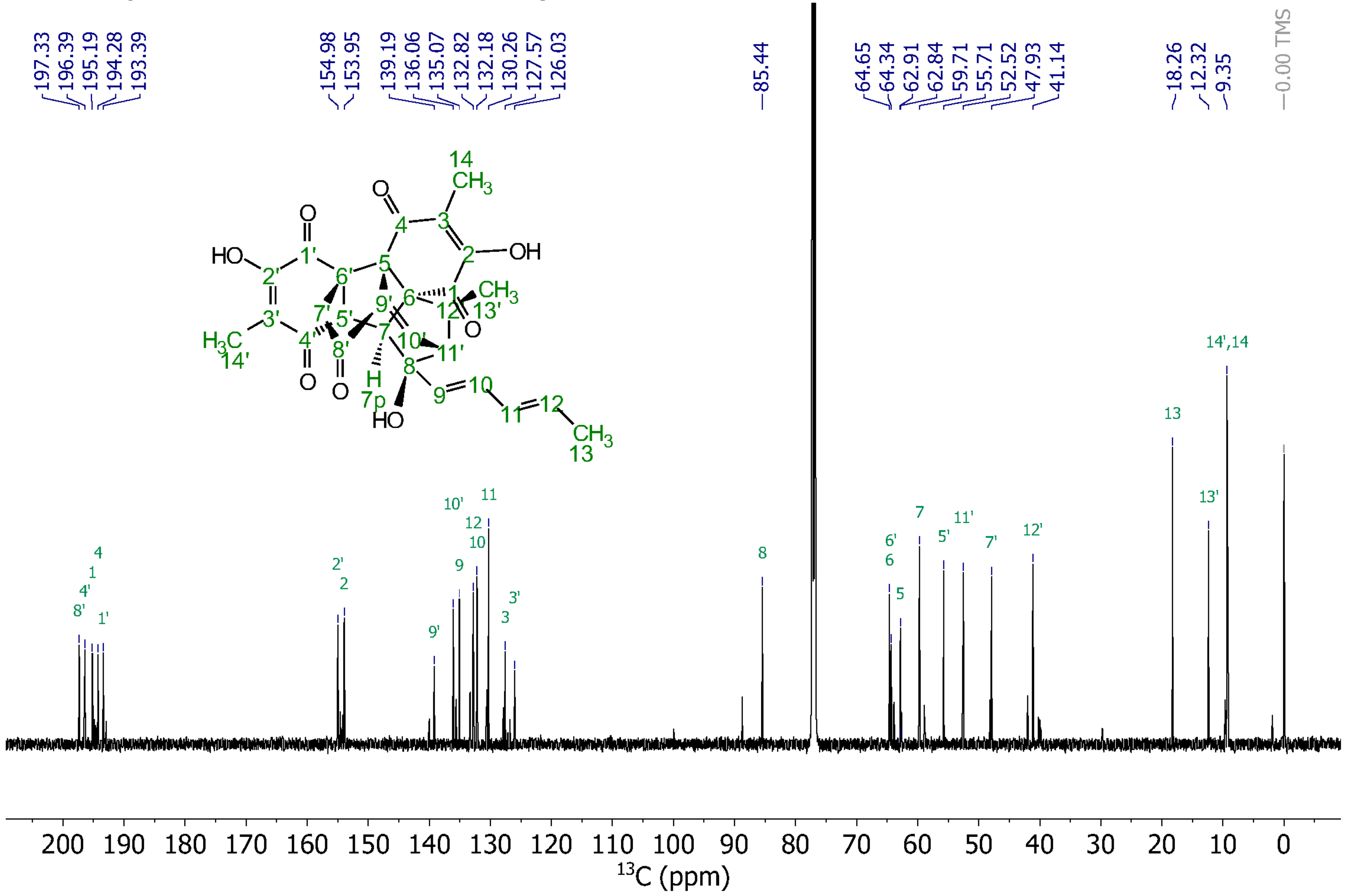

1H COSY (599.90 MHz, CDCl3, 298.0 K) — homodimericin A

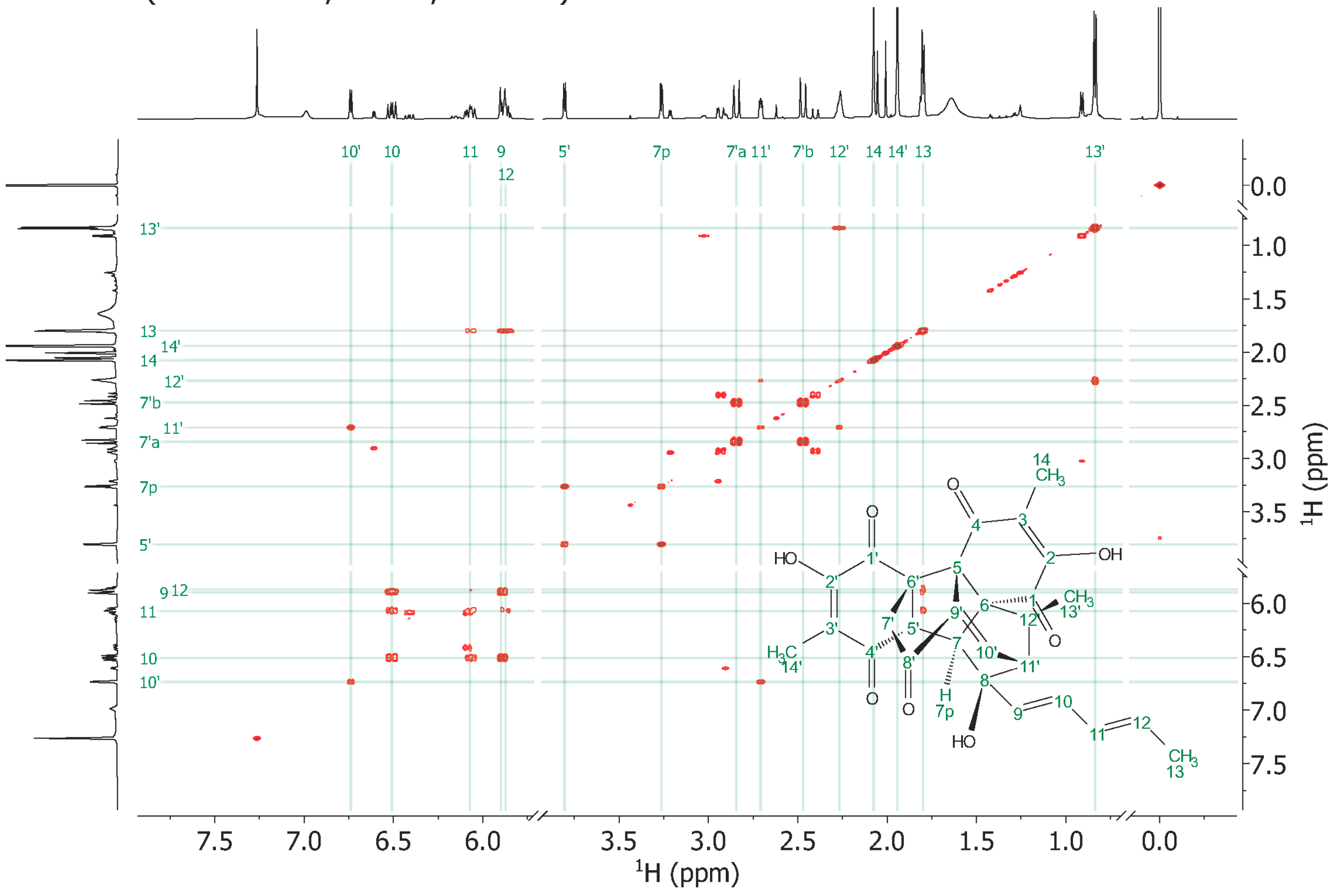

13C pure shift edited HSQC (599.90 MHz, CDCl3, 298.0 K) — homodimericin A

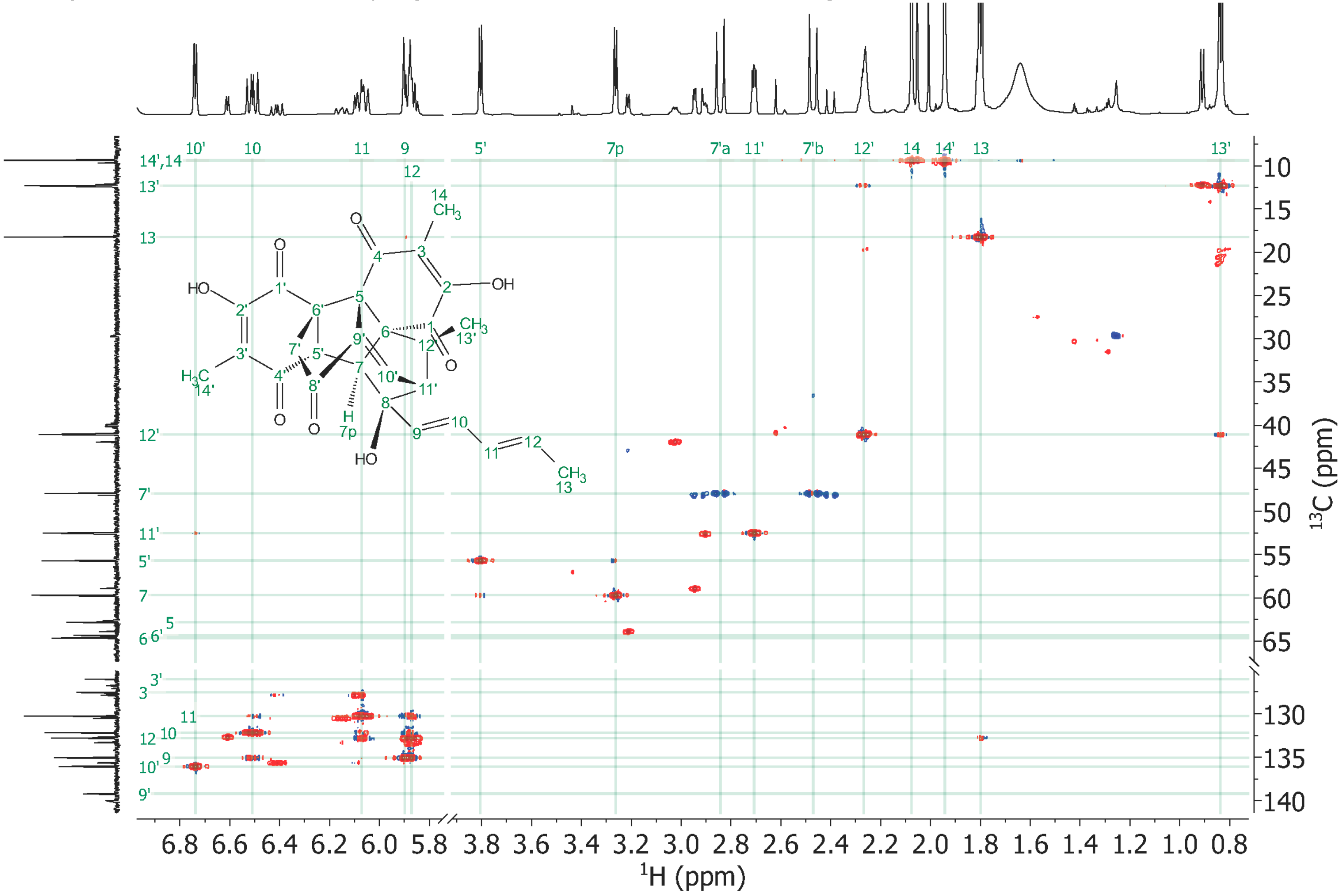

13C HMBC (599.90 MHz, CDCl3, 298.0 K) — homodimericin A

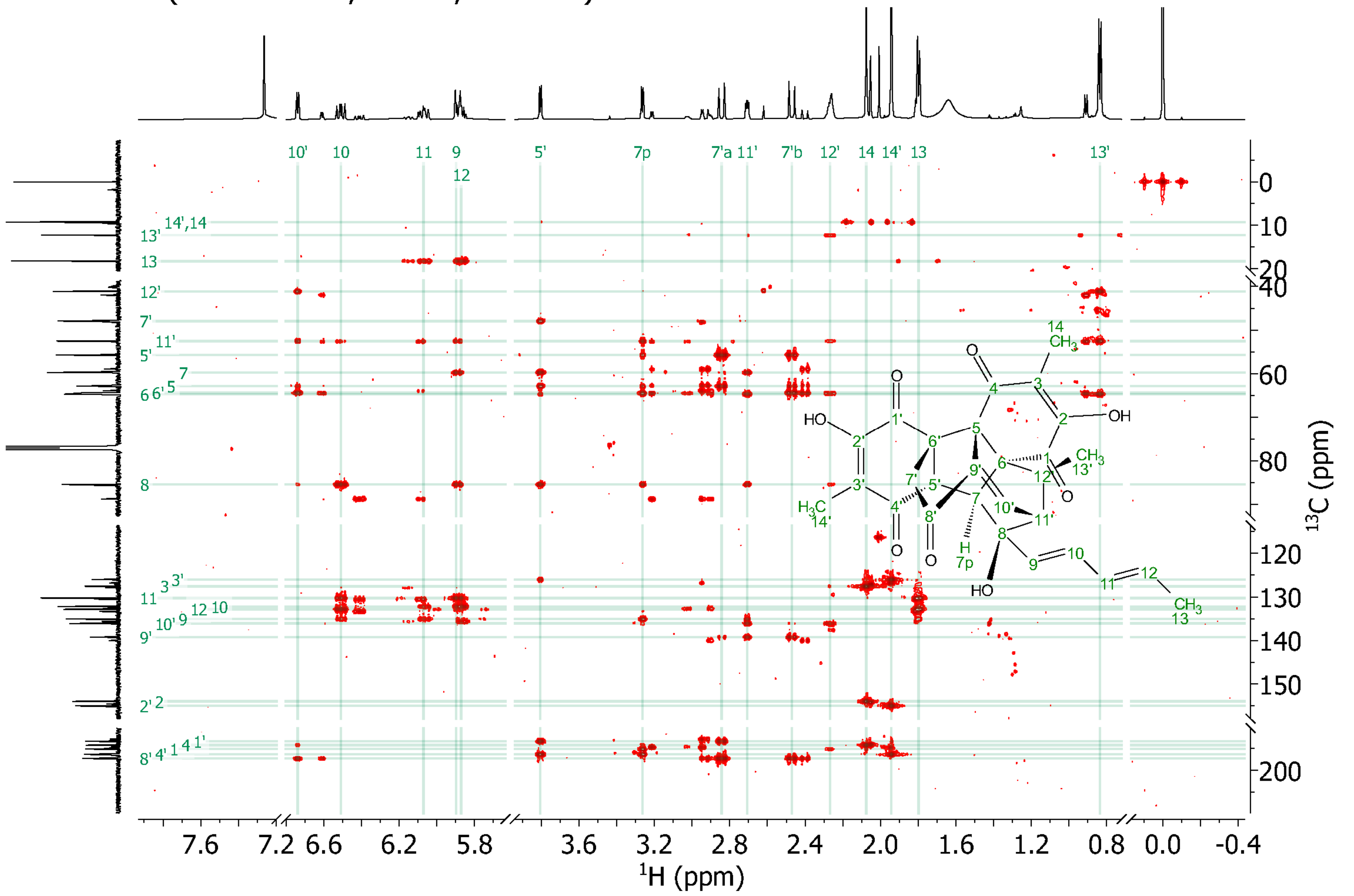

1H NOESY (599.90 MHz, CDCl3, 298.0 K) — homodimericin A

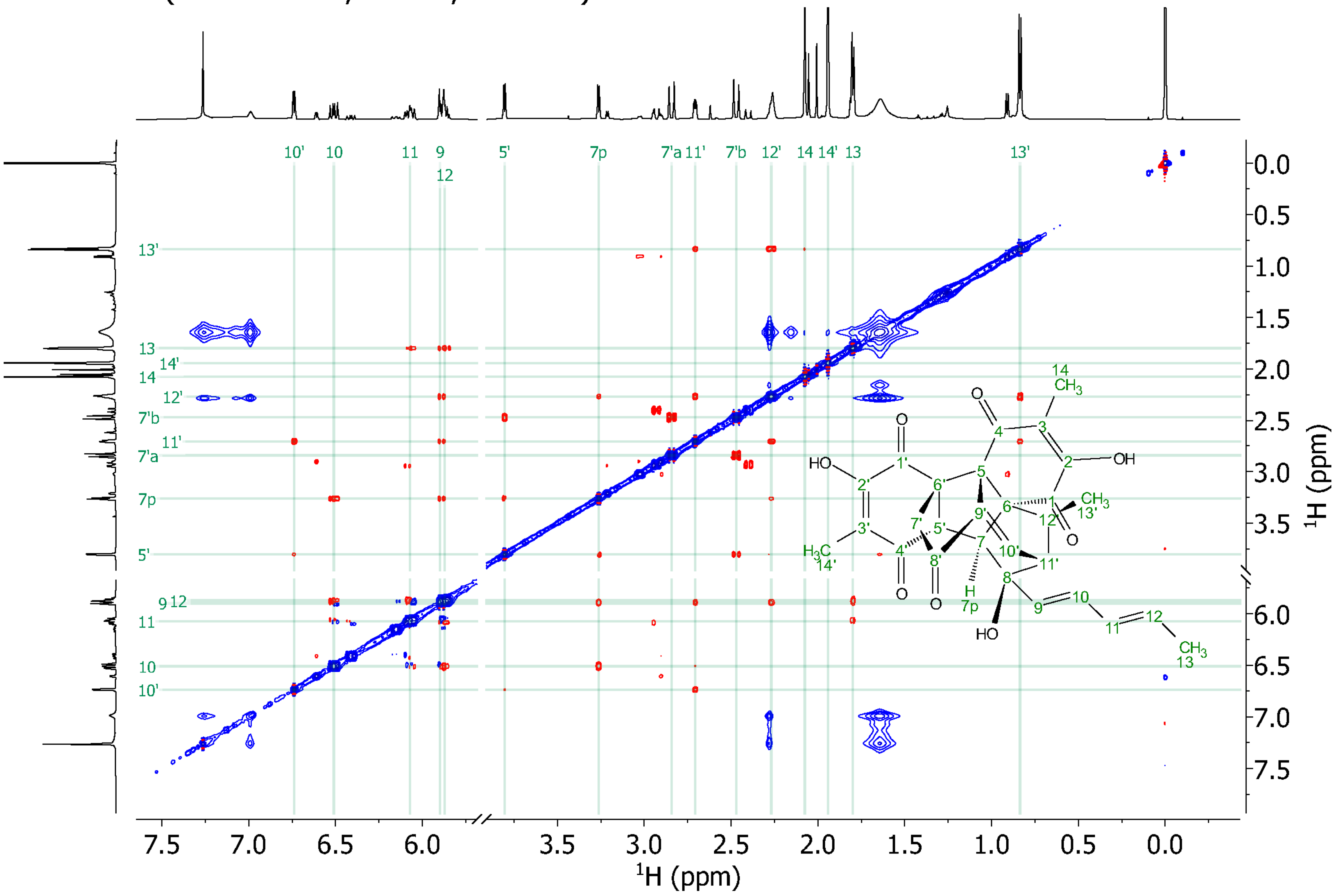

V. Probe Set Geometries

The following molecular coordinates are for each compound of the probe set, which were optimized at the B3LYP-D3/6-311G(d,p) level using an implicit PCM solvent model for chloroform. Calculations were performed in Gaussian '16, Revision C.01 [1]. Structures represent global energy minima, and stationary points were verified by vibrational frequency calculations.

Bicyclohexa2one – Method 1

conf #	Boltzmann pop. (%)	Relative Energy (kcal/mol)
1	100.0	0.0

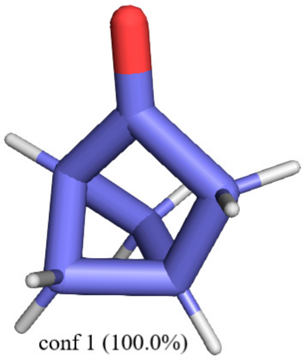

conf 1 (100.0%)

Cartesian Coordinates for Global Minimum of Bicyclohexa2one (Conf #1)

C	-0.318380	1.299849	-0.000070
C	1.127548	0.764006	-0.000034
C	-1.043502	-0.066702	-0.000233
C	0.120901	-1.047451	0.000059
C	1.052129	-0.370588	-1.063031
C	1.052003	-0.370386	1.063150
O	-2.231852	-0.289523	0.000070
H	-0.578285	1.887596	-0.888132
H	-0.578360	1.887468	0.888058
H	1.937487	1.496334	-0.000046
H	-0.123740	-2.109562	0.000153
H	1.996164	-0.911667	-1.176194
H	0.630787	-0.137655	-2.047581
H	1.996036	-0.911423	1.176532
H	0.630539	-0.137273	2.047606

Bicyclohexa2one – Method 2

conf #	Boltzmann pop. (%)	Relative Energy (kcal/mol)
1	100.0	0.0

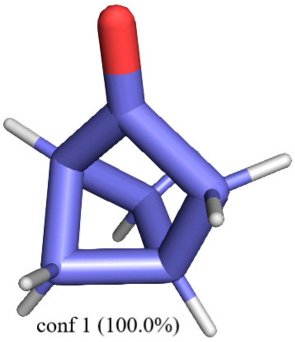

conf 1 (100.0%)

Cartesian Coordinates for Global Minimum of Bicyclohexa2one (Conf #1)

C	-0.318355	1.296505	-0.000099
C	1.128187	0.763132	-0.000047
C	-1.039136	-0.066160	-0.000321
C	0.117497	-1.046866	0.000082
C	1.050195	-0.369697	-1.064770
C	1.050018	-0.369413	1.064936
O	-2.225986	-0.286767	0.000096
H	-0.579225	1.878438	-0.888077
H	-0.579328	1.878261	0.887968
H	1.933912	1.494376	-0.000065
H	-0.131449	-2.105104	0.000212
H	1.988766	-0.914150	-1.176330
H	0.628268	-0.134706	-2.045248
H	1.988584	-0.913811	1.176803
H	0.627922	-0.134171	2.045282

Pinene – Method 1

conf #	Boltzmann pop. (%)	Relative Energy (kcal/mol)
1	100.0	0.0

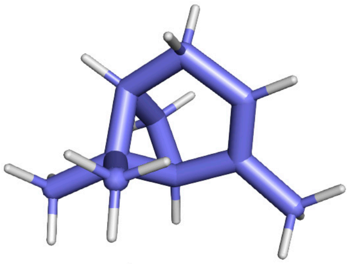

conf 1 (100.0%)

Cartesian Coordinates for Global Minimum of Pinene (Conf #1)

C	-1.088782	-0.326133	0.205103
C	0.125896	-0.641046	-0.757287
C	1.445893	-0.289108	-0.082664
C	1.513627	0.909923	0.513768
C	0.298483	1.814657	0.532442
C	-0.863559	1.165230	-0.245279
C	-0.310493	0.605690	-1.586142
C	2.577452	-1.275689	-0.137881
C	-2.413375	-0.898965	-0.325227
C	-0.982739	-0.679769	1.690708
H	0.148093	-1.621371	-1.246775
H	2.425319	1.248673	1.004164
H	-0.002384	2.028409	1.569669
H	0.541260	2.791224	0.086090
H	-1.743371	1.819695	-0.276395
H	-1.093983	0.392025	-2.315950
H	0.490309	1.170582	-2.073311
H	3.472517	-0.895147	0.365474
H	2.297686	-2.226360	0.337927
H	2.846371	-1.516733	-1.176123
H	-3.260316	-0.478333	0.232081
H	-2.584449	-0.694299	-1.385758
H	-2.440258	-1.987739	-0.190731
H	-1.057144	-1.766013	1.832241
H	-0.044479	-0.350123	2.141610
H	-1.809588	-0.223230	2.250539

Pinene – Method 2

conf #	Boltzmann pop. (%)	Relative Energy (kcal/mol)
1	100.0	0.0

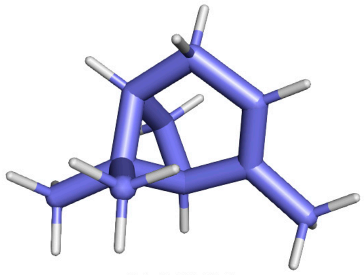

conf 1 (100.0%)

Cartesian Coordinates for Global Minimum of Pinene (Conf #1)

C	-1.083288	-0.328562	0.205942
C	0.125393	-0.632608	-0.764348
C	1.443932	-0.282117	-0.091245
C	1.513788	0.909714	0.511319
C	0.296269	1.809738	0.542768
C	-0.867621	1.165831	-0.233101
C	-0.317815	0.620301	-1.582033
C	2.564178	-1.277628	-0.146836
C	-2.406082	-0.901176	-0.323050
C	-0.965449	-0.692062	1.685542
H	0.147462	-1.607335	-1.257749
H	2.422723	1.242086	1.005312
H	0.000554	2.008057	1.581521
H	0.533123	2.788329	0.105397
H	-1.749161	1.812823	-0.252462
H	-1.100951	0.409525	-2.308547
H	0.478540	1.191107	-2.063432
H	3.461413	-0.909425	0.356158
H	2.265484	-2.220520	0.327019
H	2.823206	-1.519266	-1.184563
H	-3.247801	-0.487294	0.241999
H	-2.580423	-0.687101	-1.378332
H	-2.423889	-1.988624	-0.197988
H	-1.026792	-1.777952	1.814396
H	-0.030687	-0.356677	2.133230
H	-1.792629	-0.246325	2.248298

Aquatolide – Method 1

conf #	Boltzmann pop. (%)	Relative Energy (kcal/mol)
1	92.19	0.0
2	7.76	1.47
5	0.04	4.56
6	0.01	5.64

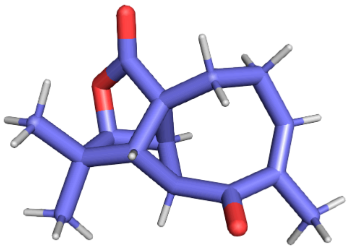
conf 1 (92.19%)

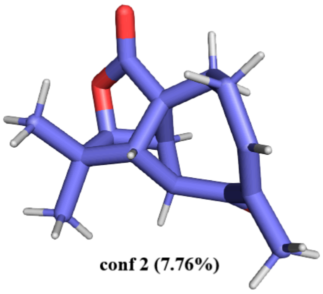
conf 2 (7.76%)

Cartesian Coordinates for Global Minimum of Aquatolide (Conf #1)

C	-1.648807	1.626917	-0.088160
C	-0.449185	0.773758	0.302870
C	-0.139441	-0.013785	-1.013005
C	-1.650807	-0.307799	-1.241585
O	-2.278026	0.994801	-1.123602
O	-2.000191	2.696557	0.343167
C	0.429298	-1.145577	-0.141877
C	-1.996527	-1.148282	0.044211
C	-0.723982	-0.722525	0.822608
C	-2.101474	-2.654244	-0.264393
C	-3.293197	-0.727415	0.759542
C	1.857080	-1.021403	0.399476
C	2.866630	-0.112743	-0.259846
O	2.212234	-1.797239	1.273110
C	4.099181	-0.854082	-0.733616
C	2.765168	1.222788	-0.362890
C	1.719424	2.164718	0.178766
C	0.625635	1.552109	1.061421
H	-1.958704	-0.731308	-2.197825
H	0.405297	0.435229	-1.845864
H	0.378350	-2.167669	-0.529072
H	-0.673125	-0.945825	1.890767
H	-1.356219	-3.011522	-0.980734
H	-3.086409	-2.873646	-0.692656
H	-1.997371	-3.248484	0.650925

H	-3.252498	0.279020	1.183355
H	-3.488667	-1.422817	1.584212
H	-4.149625	-0.765064	0.077937
H	3.852483	-1.604750	-1.496255
H	4.554349	-1.391795	0.105285
H	4.836688	-0.166109	-1.157208
H	3.598883	1.733622	-0.848225
H	2.251439	2.929911	0.762731
H	1.261093	2.720938	-0.653744
H	1.090361	0.924778	1.830496
H	0.107560	2.357920	1.593344

Aquatolide – Method 2

conf #	Boltzmann pop. (%)	Relative Energy (kcal/mol)
1	92.97	0.0
2	6.95	1.54
5	0.07	4.26
6	0.01	5.46

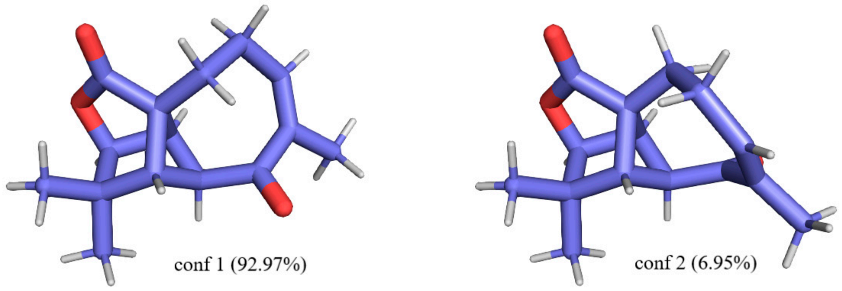

Cartesian Coordinates for Global Minimum of Aquatolide (Conf #1)

C	-1.638532	1.623277	-0.090934
C	-0.447947	0.771798	0.309468
C	-0.129241	-0.015174	-1.005488
C	-1.633852	-0.313722	-1.243563
O	-2.261652	0.996217	-1.123923
O	-1.992902	2.692207	0.332068
C	0.432596	-1.144633	-0.129205
C	-1.987140	-1.149620	0.040011
C	-0.726030	-0.723646	0.827985
C	-2.083763	-2.652440	-0.270226
C	-3.283659	-0.726851	0.747563
C	1.849787	-1.007770	0.418936
C	2.850702	-0.112910	-0.264514
O	2.201745	-1.751472	1.316010
C	4.057974	-0.872875	-0.766987
C	2.750038	1.217683	-0.372454
C	1.710681	2.157631	0.179419
C	0.627067	1.542196	1.068977
H	-1.936006	-0.735065	-2.198217
H	0.421835	0.434571	-1.828472
H	0.396072	-2.164580	-0.514698
H	-0.685329	-0.942319	1.893674
H	-1.330725	-3.000883	-0.978122
H	-3.061053	-2.871441	-0.708434
H	-1.984968	-3.240719	0.646088
H	-3.238941	0.278374	1.167926

H	-3.478748	-1.418157	1.571536
H	-4.133387	-0.764543	0.062035
H	3.771754	-1.627130	-1.508027
H	4.532201	-1.403193	0.063070
H	4.787296	-0.201246	-1.222417
H	3.570370	1.725693	-0.877195
H	2.245630	2.923290	0.753910
H	1.244957	2.705146	-0.651129
H	1.094044	0.909311	1.827735
H	0.115380	2.339609	1.613563

Naupliolide – Method 1

conf #	Boltzmann pop. (%)	Relative Energy (kcal/mol)
2	73.58	0.0
4	24.52	0.65
1	1.9	2.17

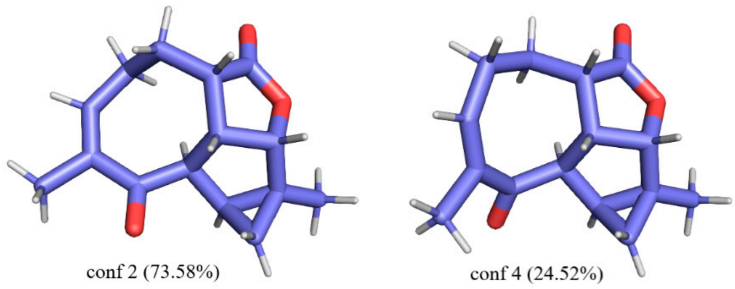

Cartesian Coordinates for Global Minimum of Naupliolide (Conf #2)

C	0.270329	1.613592	0.944678
C	1.376678	2.082524	-0.004914
O	2.342697	1.126389	-0.079236
C	1.980338	-0.044347	0.709061
C	0.451010	0.090314	0.920075
C	2.225819	-1.305230	-0.092790
C	0.877591	-1.730271	-0.650525
C	-0.165292	-0.676687	-0.293694
O	1.445530	3.124943	-0.605380
C	-1.081681	2.288931	0.680812
C	-1.816166	1.953424	-0.643950
C	-2.859055	0.855684	-0.564620
C	-2.789837	-0.461110	-0.271167
C	-1.548949	-1.239391	0.031944
C	-4.055358	-1.285174	-0.169400
O	-1.650661	-2.350600	0.536441
C	1.514709	-2.519304	0.463414
C	3.533368	-1.446098	-0.840125
H	0.807427	-2.190935	-1.632702
H	-0.246471	0.001043	-1.148236
H	0.133876	-0.377235	1.856114
H	2.555766	0.003296	1.642524
H	0.597452	1.955685	1.940442
H	-1.752796	2.093055	1.524916
H	-0.875228	3.364691	0.683841
H	-2.343878	2.859335	-0.957945
H	-1.083489	1.770114	-1.439338

H	-3.871424	1.224510	-0.733690
H	-4.173108	-1.692208	0.840464
H	-4.937719	-0.686919	-0.414328
H	-4.018361	-2.148746	-0.843348
H	1.936690	-3.484195	0.195634
H	1.070160	-2.500636	1.453765
H	3.541747	-2.366518	-1.434029
H	3.695150	-0.599762	-1.515139
H	4.382663	-1.491572	-0.146345

Naupliolide – Method 2

conf #	Boltzmann pop. (%)	Relative Energy (kcal/mol)
3	50.19	0.0
2	38.59	0.16
4	9.37	0.99
1	1.85	1.95

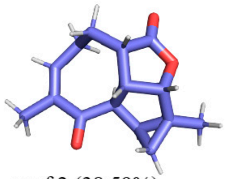

conf 2 (38.59%)

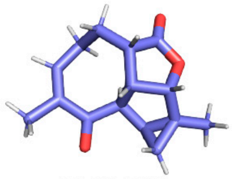

conf 3 (50.19%)

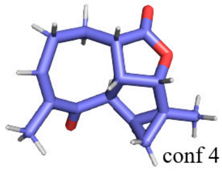

conf 4 (9.37%)

Cartesian Coordinates for Global Minimum of Naupliolide (Conf #3)

C	0.234229	1.682951	0.806163
C	1.434076	2.060176	-0.057316
O	2.361399	1.079480	0.015578
C	1.866095	-0.044824	0.814126
C	0.328612	0.145480	0.825511
C	2.182225	-1.326313	0.087339
C	0.924509	-1.715589	-0.654645
C	-0.146554	-0.657844	-0.434401
O	1.604266	3.060830	-0.701895
C	-1.035749	2.460504	0.456174
C	-1.873538	1.919321	-0.722312
C	-2.869801	0.884235	-0.271753
C	-2.760151	-0.440044	-0.066942
C	-1.542979	-1.271009	-0.309604
C	-3.955989	-1.244274	0.392343
O	-1.669277	-2.483341	-0.381924
C	1.363942	-2.507831	0.551245
C	3.575231	-1.518553	-0.461270
H	0.978014	-2.188103	-1.627173
H	-0.146400	0.001877	-1.306313
H	-0.114718	-0.268954	1.730881
H	2.323466	0.035434	1.804448
H	0.534450	2.009691	1.812481

H	-1.673955	2.526288	1.341135
H	-0.712222	3.478591	0.224169
H	-2.437297	2.753319	-1.146083
H	-1.222191	1.562976	-1.521638
H	-3.847431	1.307831	-0.048432
H	-4.818387	-0.600894	0.572495
H	-4.223446	-1.994454	-0.355546
H	-3.727575	-1.792103	1.311145
H	1.790191	-3.485838	0.363305
H	0.783969	-2.449289	1.465307
H	4.311266	-1.566053	0.347853
H	3.637801	-2.450713	-1.027467
H	3.848414	-0.693682	-1.122590

Echinopine B – Method 1

conf #	Boltzmann pop. (%)	Relative Energy (kcal/mol)
1	38.62	0.0
2	35.65	0.05
3	23.18	0.3
6	0.88	2.24
7	0.8	2.3
9	0.45	2.63
8	0.27	2.94
13	0.1	3.51
11	0.03	4.15
10	0.0	6.02
15	0.0	7.04
19	0.0	7.87
16	0.0	9.86
17	0.0	10.2
18	0.0	10.36

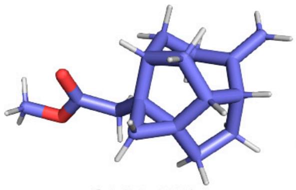

conf 1 (38.62%)

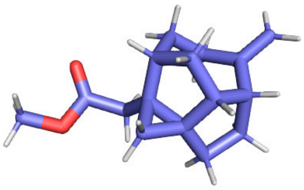

conf 2 (35.65%)

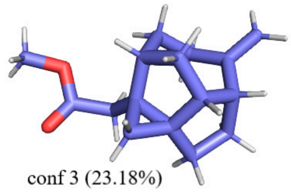

conf 3 (23.18%)

Cartesian Coordinates for Global Minimum of Echinopine B (Conf #1)

C	2.803180	-0.466029	-0.206331
C	2.731673	1.009220	-0.569093
C	1.388993	1.610123	-0.946667
C	0.441196	1.940264	0.232806
C	1.896831	-0.879642	1.000736
C	0.658454	-1.474654	0.337074
C	-0.469169	-0.448464	0.461559
C	-1.519200	-0.271423	-0.618504
C	-2.867357	0.190517	-0.088998
O	-3.844109	-0.064174	-0.992536
C	-5.157896	0.382761	-0.620335

O	-3.072789	0.739602	0.971636
C	3.838686	1.761855	-0.574789
H	3.846862	-0.676374	0.049427
C	0.098142	0.782030	1.193697
H	-0.624990	1.164189	1.922329
C	2.357562	-1.405963	-1.385791
C	1.107194	-2.203343	-0.912949
H	2.433212	-1.637730	1.587892
C	1.368458	0.248163	1.905835
C	-0.517429	-1.765161	1.222613
H	0.875942	0.934414	-1.643322
H	1.562918	2.539699	-1.501939
H	0.894839	2.744660	0.826298
H	-0.485622	2.358868	-0.182031
H	-1.212237	0.464040	-1.374190
H	-1.676736	-1.205456	-1.169053
H	-5.806710	0.111618	-1.453878
H	-5.486344	-0.110980	0.298214
H	-5.167028	1.464666	-0.462200
H	3.815406	2.812841	-0.854939
H	4.810483	1.356822	-0.301730
H	3.172928	-2.080261	-1.666665
H	2.129453	-0.815262	-2.278983
H	0.332067	-2.259999	-1.686179
H	1.380484	-3.237770	-0.665587
H	1.108994	-0.159454	2.889410
H	2.108879	1.036520	2.077450
H	-1.249109	-2.489716	0.869470
H	-0.384413	-1.780283	2.302223

Echinopine B – Method 2

conf #	Boltzmann pop. (%)	Relative Energy (kcal/mol)
2	41.01	0.0
1	32.55	0.14
3	25.0	0.29
7	0.51	2.59
6	0.46	2.66
9	0.17	3.25
8	0.13	3.39
12	0.08	3.7
11	0.06	3.84
13	0.02	4.54
10	0.0	6.92
14	0.0	8.14
19	0.0	8.68
15	0.0	8.84
16	0.0	9.83
18	0.0	10.01
17	0.0	10.11

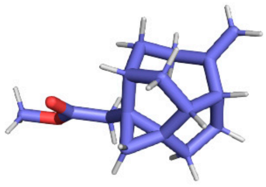
conf 1 (32.55%)

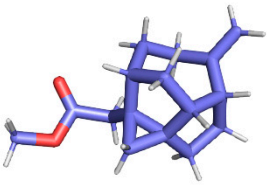
conf 2 (41.01%)

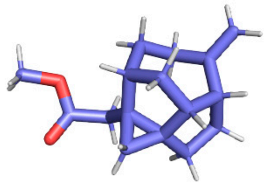
conf 3 (25.00%)

Cartesian Coordinates for Global Minimum of Echinopine B (Conf #2)

C	2.752463	-0.602629	0.041555
C	2.822171	0.867783	-0.330104
C	1.585615	1.531743	-0.904563
C	0.523703	1.961402	0.133591
C	1.654688	-0.942992	1.098902
C	0.488731	-1.449303	0.260705
C	-0.561668	-0.347696	0.226730
C	-1.435152	-0.086401	-0.993462
C	-2.760472	0.487714	-0.548896

O	-3.669405	-0.479488	-0.319870
C	-4.941509	-0.040400	0.202755
O	-2.981646	1.663779	-0.367136
C	3.952334	1.559471	-0.162115
H	3.728313	-0.874487	0.449169
C	-0.027619	0.841877	1.038131
H	-0.815571	1.279033	1.660470
C	2.425282	-1.524078	-1.187264
C	1.049820	-2.202032	-0.922409
H	2.040480	-1.734725	1.750372
C	1.085283	0.223808	1.923996
C	-0.820202	-1.654061	0.962697
H	1.126204	0.857948	-1.633672
H	1.888005	2.422769	-1.461731
H	0.958962	2.733067	0.776904
H	-0.308701	2.433771	-0.396656
H	-0.977767	0.630321	-1.675180
H	-1.606246	-1.017912	-1.534545
H	-5.533662	-0.943949	0.325702
H	-4.804234	0.462281	1.161362
H	-5.424774	0.643746	-0.496180
H	4.025475	2.605781	-0.442761
H	4.842051	1.103130	0.260116
H	3.210922	-2.270820	-1.317239
H	2.401746	-0.938285	-2.108431
H	0.394951	-2.170737	-1.796733
H	1.183145	-3.257098	-0.661695
H	0.660498	-0.157214	2.855597
H	1.846543	0.957952	2.195858
H	-1.544777	-2.312231	0.496545
H	-0.843964	-1.671918	2.047277

Parthenolide – Method 1

conf #	Boltzmann pop. (%)	Relative Energy (kcal/mol)
1	99.15	0.0
2	0.85	2.82
6	0.0	7.49
3	0.0	8.11

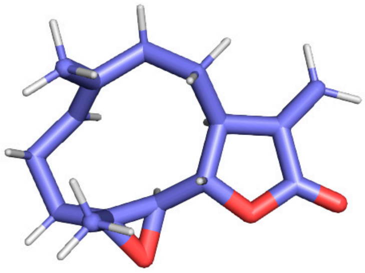

conf 1 (99.15%)

Cartesian Coordinates for Global Minimum of Parthenolide (Conf #1)

C	-3.389830	-0.146276	-0.824001
C	-2.754241	-1.566775	-0.794932
C	-1.557923	-1.644218	0.145042
C	-0.239154	-1.300769	-0.432921
C	-1.874066	1.803384	-0.121152
C	-2.326697	0.904374	-1.009684
C	0.896641	-0.632188	0.307078
C	1.303410	0.759970	-0.276250
C	0.653885	2.024815	0.336518
C	-0.599850	2.576348	-0.396105
C	-2.527407	2.119155	1.204095
O	-0.577075	-2.661067	-0.143064
C	-1.866119	-1.512825	1.623284
O	2.070733	-1.476818	0.174722
C	3.202363	-0.735845	0.012529
C	2.812524	0.696439	-0.154410
O	4.306068	-1.222203	0.009371
C	3.734293	1.658530	-0.196258
H	-4.117620	-0.114174	-1.645972
H	-3.957186	0.021396	0.096651
H	-2.417735	-1.844140	-1.800777
H	-3.513758	-2.302937	-0.500846
H	-0.211309	-1.100131	-1.506809
H	-1.784458	0.826757	-1.954424
H	0.688243	-0.550508	1.378848

H	1.066811	0.741330	-1.352090
H	0.430566	1.849894	1.397032
H	1.405463	2.821910	0.325269
H	-0.393500	2.596202	-1.474052
H	-0.729020	3.622226	-0.084039
H	-3.429559	1.533385	1.394134
H	-1.840713	1.963031	2.046808
H	-2.812196	3.180138	1.233533
H	-2.305147	-0.541885	1.868846
H	-2.586849	-2.288760	1.908755
H	-0.970572	-1.660525	2.230904
H	3.489764	2.709718	-0.319195
H	4.783984	1.393079	-0.107804

Parthenolide – Method 2

conf #	Boltzmann pop. (%)	Relative Energy (kcal/mol)
1	99.29	0.0
2	0.66	2.97
4	0.04	4.61
6	0.0	7.07
3	0.0	7.47

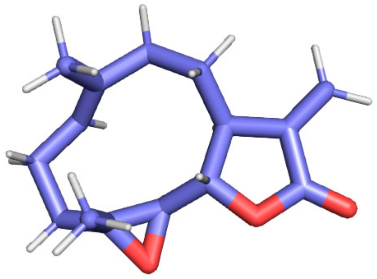

conf 1 (99.29%)

Cartesian Coordinates for Global Minimum of Parthenolide (Conf #1)

C	-3.397539	-0.121540	-0.796582
C	-2.772984	-1.544538	-0.789082
C	-1.570334	-1.622001	0.134627
C	-0.262190	-1.269333	-0.449619
C	-1.842944	1.790680	-0.109902
C	-2.328504	0.917132	-0.999875
C	0.868454	-0.607798	0.295565
C	1.309819	0.764078	-0.294705
C	0.680706	2.038058	0.309416
C	-0.583295	2.572868	-0.408934
C	-2.443440	2.073222	1.245490
O	-0.588644	-2.638762	-0.165928
C	-1.861080	-1.483513	1.611265
O	2.036217	-1.479945	0.184795
C	3.172991	-0.762256	0.021665
C	2.813206	0.672064	-0.159259
O	4.263521	-1.273388	0.029137
C	3.747629	1.616973	-0.200911
H	-4.138350	-0.077982	-1.601761
H	-3.940827	0.043292	0.135526
H	-2.449766	-1.814655	-1.798378
H	-3.525908	-2.278136	-0.483652
H	-0.240570	-1.059700	-1.518082
H	-1.815573	0.848207	-1.958530
H	0.657332	-0.513509	1.361845

H	1.088041	0.744667	-1.369907
H	0.480932	1.878322	1.373400
H	1.429599	2.832030	0.266894
H	-0.392077	2.587249	-1.486723
H	-0.720833	3.615467	-0.099795
H	-3.359170	1.515756	1.439582
H	-1.736125	1.853745	2.052497
H	-2.681766	3.139831	1.324622
H	-2.262888	-0.500087	1.851306
H	-2.603795	-2.233037	1.899245
H	-0.965884	-1.655514	2.207566
H	3.514828	2.666836	-0.331282
H	4.791075	1.339405	-0.103361

Diepoxyguainaolide – Method 1

conf #	Boltzmann pop. (%)	Relative Energy (kcal/mol)
1	96.76	0.0
2	1.96	2.31
4	1.13	2.63
5	0.15	3.84

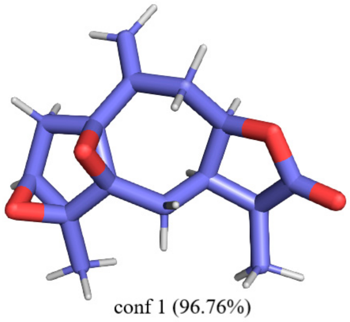

conf 1 (96.76%)

Cartesian Coordinates for Global Minimum of Diepoxyguainaolide (Conf #1)

C	0.322545	-0.852526	0.483241
C	1.196406	-0.233004	-0.625799
C	1.596109	1.239107	-0.348551
C	0.626891	2.105255	0.458295
C	-0.752549	2.141513	-0.160535
C	-1.635765	0.969816	0.124409
C	-1.136735	-0.410349	0.449621
C	-3.040972	0.867368	-0.477267
C	-3.379357	-0.615924	-0.477616
C	-2.244765	-1.406636	0.036397
C	2.598928	-0.873365	-0.773120
C	3.454567	-0.037369	0.177220
O	2.842292	1.160842	0.391391
C	-1.152228	3.171881	-0.916106
O	-3.445031	-1.240124	0.810145
O	-1.573644	0.476575	1.483036
O	4.508113	-0.328530	0.681812
C	2.775434	-2.375420	-0.559432
H	0.647082	-0.290949	-1.572212
C	-1.927020	-2.815830	-0.392666
H	0.358268	-1.941004	0.390052
H	0.732153	-0.620565	1.473058
H	1.813268	1.737161	-1.303381
H	1.040569	3.116320	0.519492

H	0.565005	1.716895	1.479049
H	-3.039958	1.230933	-1.510829
H	-3.764956	1.460058	0.092382
H	-4.081360	-1.006274	-1.214940
H	2.962260	-0.623697	-1.782708
H	-2.138130	3.225219	-1.366250
H	-0.495906	4.018496	-1.098524
H	3.826939	-2.641396	-0.699851
H	2.174219	-2.950707	-1.272060
H	2.498279	-2.679373	0.454334
H	-1.208143	-2.835818	-1.220174
H	-1.508838	-3.399487	0.435840
H	-2.843516	-3.313014	-0.722892

Diepoxyguainaolide – Method 2

conf #	Boltzmann pop. (%)	Relative Energy (kcal/mol)
1	93.47	0.0
2	4.95	1.74
4	1.03	2.67
5	0.28	3.44
3	0.26	3.48
6	0.0	6.92

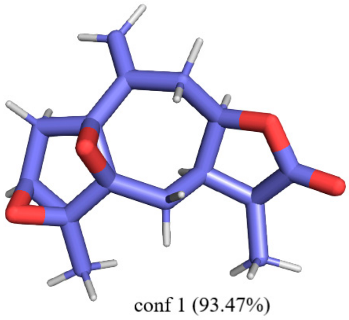

conf 1 (93.47%)

Cartesian Coordinates for Global Minimum of Diepoxyguainaolide (Conf #1)

C	0.327409	-0.828526	0.520813
C	1.176756	-0.231324	-0.614122
C	1.576106	1.240645	-0.359867
C	0.622031	2.107827	0.455362
C	-0.759051	2.141290	-0.154766
C	-1.635805	0.967837	0.130424
C	-1.130691	-0.400468	0.473896
C	-3.024782	0.845508	-0.495329
C	-3.344251	-0.638312	-0.500720
C	-2.213491	-1.409463	0.039054
C	2.573736	-0.872845	-0.770038
C	3.435218	-0.034384	0.163077
O	2.835467	1.162253	0.374505
C	-1.163800	3.168078	-0.906917
O	-3.432631	-1.265751	0.790324
O	-1.590207	0.487002	1.498421
O	4.489250	-0.321267	0.664876
C	2.737270	-2.373122	-0.551293
H	0.614969	-0.304470	-1.547911
C	-1.855988	-2.804702	-0.390509
H	0.373038	-1.915048	0.466334
H	0.739373	-0.558815	1.496372

H	1.790027	1.730206	-1.315615
H	1.035701	3.115685	0.512568
H	0.567283	1.717447	1.472998
H	-3.003900	1.199601	-1.528527
H	-3.763439	1.433090	0.054795
H	-4.017891	-1.041121	-1.252200
H	2.929902	-0.624916	-1.779251
H	-2.149546	3.215554	-1.351952
H	-0.507615	4.011145	-1.092202
H	3.773705	-2.660055	-0.736058
H	2.095076	-2.937280	-1.231505
H	2.495985	-2.660480	0.473711
H	-1.112741	-2.792398	-1.192149
H	-1.449397	-3.380708	0.445500
H	-2.749565	-3.313566	-0.754300

Cannabicitran – Method 1

conf #	Boltzmann pop. (%)	Relative Energy (kcal/mol)
1	99.91	0.0
2	0.09	4.18

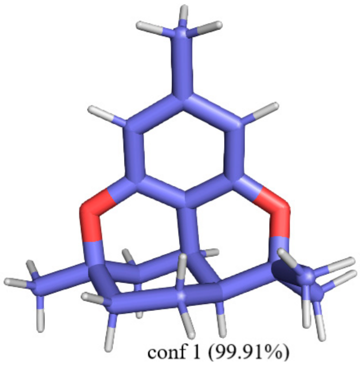

conf 1 (99.91%)

Cartesian Coordinates for Global Minimum of Cannabicitran (Conf #1)

C	-2.234511	-0.173869	1.317680
C	-0.928085	-1.004722	1.442063
C	-0.533858	-1.757529	0.143344
C	-0.741368	-0.793828	-1.046770
C	-2.173546	-0.279318	-1.180742
C	-2.457156	0.637899	0.011489
C	0.189331	0.338061	-0.735050
C	1.528382	-0.000487	-0.539353
C	2.474250	0.965880	-0.204031
C	2.042599	2.282828	0.034503
C	0.672378	2.576266	0.037465
C	-0.255166	1.578535	-0.285488
C	0.947746	-2.284686	0.056404
O	1.848386	-1.331516	-0.637562
C	1.586231	-2.567177	1.420277
C	1.002643	-3.536971	-0.822616
C	-3.863617	1.229713	-0.020720
O	-1.590319	1.811445	-0.078616
C	3.051633	3.365315	0.343863
H	-3.092470	-0.856976	1.387992
H	-2.310031	0.506222	2.174746
H	-0.116848	-0.337871	1.744335
H	-1.056883	-1.718711	2.264839
H	-1.189896	-2.633395	0.033562
H	-0.429935	-1.287594	-1.975882
H	-2.294634	0.283488	-2.114231

H	-2.890370	-1.110269	-1.198087
H	3.517141	0.686719	-0.087427
H	0.313036	3.554757	0.342365
H	2.586068	-2.989326	1.278023
H	0.988080	-3.284182	1.994573
H	1.687831	-1.652711	2.011224
H	0.504018	-4.376537	-0.325790
H	0.514861	-3.366179	-1.787802
H	2.044082	-3.813647	-1.013357
H	-4.006712	1.811095	-0.937085
H	-4.619277	0.437942	0.012397
H	-4.019258	1.896124	0.834334
H	3.917227	2.964906	0.883153
H	3.430317	3.827795	-0.577634
H	2.611796	4.163449	0.951269

Cannabicitran – Method 2

conf #	Boltzmann pop. (%)	Relative Energy (kcal/mol)
1	99.93	0.0
2	0.07	4.28

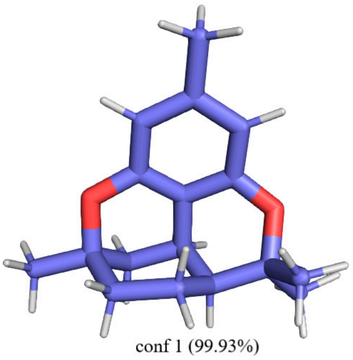

conf 1 (99.93%)

Cartesian Coordinates for Global Minimum of Cannabicitran (Conf #1)

C	-2.224005	-0.133200	1.325727
C	-0.918327	-0.958835	1.445248
C	-0.557446	-1.735905	0.155330
C	-0.758660	-0.787362	-1.047096
C	-2.182205	-0.255900	-1.172636
C	-2.447741	0.665315	0.016528
C	0.191254	0.332734	-0.757249
C	1.523409	-0.026663	-0.563933
C	2.482141	0.919091	-0.216181
C	2.074244	2.239501	0.031490
C	0.711565	2.554889	0.029033
C	-0.231472	1.576979	-0.302246
C	0.906891	-2.293756	0.063552
O	1.820645	-1.360180	-0.659391
C	1.554477	-2.553646	1.422339
C	0.930657	-3.555176	-0.795722
C	-3.840927	1.277243	-0.014097
O	-1.558022	1.830357	-0.083427
C	3.100092	3.300117	0.353166
H	-3.078784	-0.814655	1.402157
H	-2.294595	0.555834	2.172168
H	-0.101697	-0.288046	1.714950
H	-1.028685	-1.655044	2.280901
H	-1.231423	-2.595082	0.064329
H	-0.462453	-1.296120	-1.968821
H	-2.301550	0.306998	-2.102841
H	-2.906852	-1.075353	-1.178562

H	3.515924	0.618072	-0.093893
H	0.365942	3.533255	0.342286
H	2.538056	-3.005255	1.278449
H	0.942076	-3.234262	2.019492
H	1.684600	-1.625121	1.979827
H	0.424784	-4.373986	-0.278478
H	0.433022	-3.386591	-1.753008
H	1.963202	-3.850221	-0.992710
H	-3.973077	1.854327	-0.931732
H	-4.603736	0.496529	0.022819
H	-3.981055	1.945821	0.838594
H	3.951006	2.880200	0.895312
H	3.489758	3.752065	-0.565502
H	2.670865	4.102658	0.957283

Ingenane – Method 1

conf #	Boltzmann pop. (%)	Relative Energy (kcal/mol)
1	70.81	0.0
2	29.19	0.52

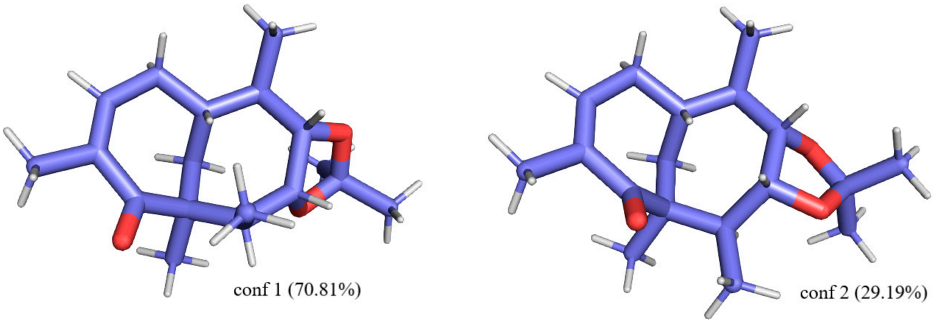

Cartesian Coordinates for Global Minimum of Ingenane (Conf #1)

C	-0.587238	0.199723	-1.111463
C	-0.572585	1.351274	-0.085566
C	-1.690462	2.324428	-0.432173
C	-3.030658	1.630924	-0.444785
C	-3.388529	0.340351	-0.237311
C	-2.531837	-0.884402	0.053226
C	-1.064486	-1.070126	-0.426520
O	-3.091999	-1.830144	0.595583
C	-4.868087	-0.001753	-0.207339
C	-0.062019	-1.449233	0.754029
C	1.340068	-0.778037	0.732458
C	1.659057	0.741301	0.877391
C	0.867198	1.843717	0.106697
O	2.085385	-1.123124	-0.442083
C	3.304549	-0.380943	-0.370325
O	3.040688	0.747064	0.489566
C	3.671925	0.087738	-1.774832
C	4.414448	-1.216331	0.271233
C	-1.103056	-2.242483	-1.434541
H	-0.874479	0.951184	0.891605
C	1.004758	3.179869	0.847583
H	1.627674	0.996545	1.944739
H	1.853883	-1.231747	1.595563
H	-1.243413	0.439484	-1.956578
H	0.405313	0.028122	-1.530062
H	-1.749076	3.156941	0.284597
H	-1.526272	2.794230	-1.416094

H	-3.861025	2.317783	-0.617256
H	-5.478690	0.871637	-0.453898
H	-5.104752	-0.804974	-0.914591
H	-5.159936	-0.370322	0.781125
H	1.335761	1.957125	-0.878544
H	3.836651	-0.773073	-2.430899
H	2.868844	0.699230	-2.194247
H	4.588337	0.685015	-1.743521
H	5.307102	-0.601126	0.421916
H	4.094401	-1.596852	1.245577
H	4.673869	-2.064820	-0.370098
H	-1.494862	-3.146308	-0.959124
H	-1.742127	-2.006136	-2.294469
H	-0.091857	-2.441548	-1.804779
H	2.061634	3.412258	1.019516
H	0.569426	4.006258	0.274900
H	0.504942	3.149011	1.825117
C	-0.624618	-1.373099	2.190971
H	0.131619	-1.717256	2.906312
H	-1.502026	-2.012380	2.290526
H	-0.917045	-0.359037	2.486384
H	0.182946	-2.507116	0.599365

Ingenane – Method 2

conf #	Boltzmann pop. (%)	Relative Energy (kcal/mol)
2	50.79	0.0
1	49.21	0.02

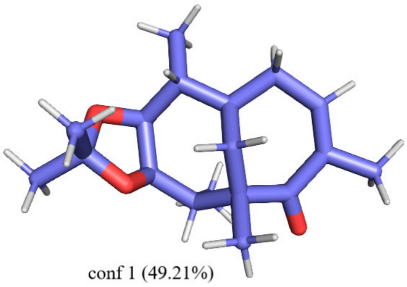

conf 1 (49.21%)

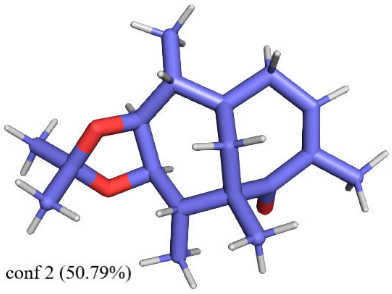

conf 2 (50.79%)

Cartesian Coordinates for Global Minimum of Ingenane (Conf #2)

C	0.977236	0.339179	1.349711
C	0.869623	1.455235	0.291304
C	2.140987	2.291015	0.332863
C	3.334673	1.405092	0.060991
C	3.365021	0.101016	-0.272427
C	2.166453	-0.822613	-0.489870
C	1.118392	-1.000471	0.633021
O	2.143473	-1.490968	-1.507382
C	4.672327	-0.581817	-0.613372
C	-0.318337	-1.420131	0.168911
C	-1.112879	-0.408716	-0.717995
C	-1.499962	1.044619	-0.285145
C	-0.525364	2.081715	0.330105
O	-2.449216	-0.933416	-0.888424
C	-3.361657	-0.244076	-0.010142
O	-2.603376	0.805296	0.591696
C	-3.861888	-1.168890	1.089723
C	-4.494995	0.320349	-0.861728
C	1.710191	-2.059898	1.590081
H	0.916645	0.995974	-0.705120
C	-0.632297	3.421933	-0.404475
H	-1.882826	1.483465	-1.217929
H	-0.661505	-0.384518	-1.713450
H	1.830624	0.520382	2.006446
H	0.093880	0.312598	1.991777
H	2.128397	3.095045	-0.412750
H	2.268112	2.782529	1.307075
H	4.294711	1.916446	0.101615

H	5.517324	0.099780	-0.504881
H	4.841714	-1.448886	0.033954
H	4.643461	-0.953172	-1.640355
H	-0.828385	2.234486	1.370335
H	-3.019342	-1.561717	1.658475
H	-4.521388	-0.621405	1.766520
H	-4.416205	-2.001601	0.652929
H	-5.225153	0.828608	-0.228535
H	-4.099865	1.029221	-1.591037
H	-4.992737	-0.488153	-1.400969
H	1.890186	-3.012128	1.090246
H	2.660003	-1.708353	2.002248
H	1.022925	-2.228889	2.424144
H	-1.667306	3.774145	-0.414532
H	-0.022310	4.186754	0.082091
H	-0.295944	3.331512	-1.442617
C	-0.393394	-2.802804	-0.505301
H	-1.433962	-3.062444	-0.696552
H	0.032208	-3.576558	0.135655
H	0.153482	-2.806866	-1.447256
H	-0.878565	-1.487982	1.107825

Artemisinin – Method 1

conf #	Boltzmann pop. (%)	Relative Energy (kcal/mol)
1	100.0	0.0
3	0.0	7.41
2	0.0	7.53
6	0.0	9.99
7	0.0	10.4
4	0.0	12.34

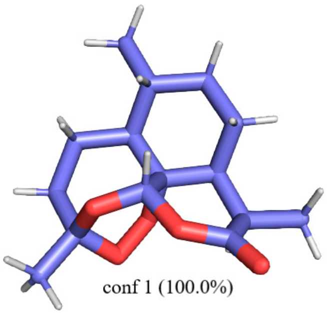
conf 1 (100.0%)

Cartesian Coordinates for Global Minimum of Artemisinin (Conf #1)

C	1.152519	1.321847	0.372654
C	0.052047	0.222919	0.365119
C	-1.353431	0.782426	0.655659
C	-1.696287	1.946461	-0.289308
C	-0.635510	3.050077	-0.200588
C	0.786550	2.547180	-0.506378
C	0.058230	-0.561017	-0.959223
O	-1.071975	-1.429102	-1.157149
C	-2.291313	-1.263576	-0.565920
C	-2.367109	-0.381365	0.674084
C	2.546715	0.751173	0.016677
C	2.863757	-0.665971	0.528207
C	1.844010	-1.740573	0.084036
O	1.212496	-1.324363	-1.142771
C	2.445576	-3.113903	-0.164245
O	0.839653	-1.948497	1.057205
O	0.349833	-0.644745	1.494965
C	1.801259	3.691073	-0.354469
H	-1.314373	1.191601	1.675690
C	-3.812344	0.041441	0.956547
O	-3.228719	-1.884563	-1.004764
H	0.031946	0.147776	-1.791757
H	1.178187	1.661903	1.418570
H	-2.677057	2.354424	-0.022423

H	-1.786792	1.591243	-1.326274
H	-0.887035	3.867917	-0.887698
H	-0.651754	3.481207	0.811897
H	0.807831	2.225959	-1.559834
H	-2.039092	-1.044087	1.487022
H	2.660930	0.748153	-1.074100
H	3.315360	1.428571	0.404505
H	3.848971	-0.948317	0.138801
H	2.929726	-0.695087	1.620509
H	1.648737	-3.838777	-0.348668
H	3.089254	-3.066253	-1.046975
H	3.037775	-3.432762	0.698259
H	1.491341	4.559339	-0.947363
H	1.874281	4.012890	0.692482
H	2.802927	3.405034	-0.689246
H	-3.861806	0.637523	1.875050
H	-4.232170	0.632771	0.136955
H	-4.445504	-0.840018	1.077603

Artemisinin – Method 2

conf #	Boltzmann pop. (%)	Relative Energy (kcal/mol)
1	100.0	0.0
3	0.0	7.35
2	0.0	7.38
6	0.0	9.86
7	0.0	10.23
4	0.0	12.05

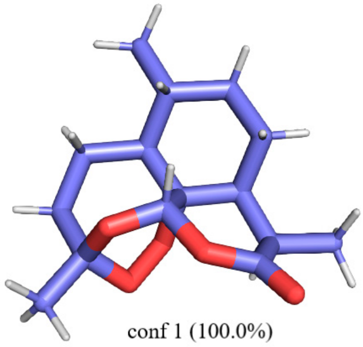

conf 1 (100.0%)

Cartesian Coordinates for Global Minimum of Artemisinin (Conf #1)

C	1.148261	1.321676	0.379621
C	0.050866	0.227093	0.367062
C	-1.352106	0.782792	0.652436
C	-1.694235	1.939807	-0.296604
C	-0.636326	3.042157	-0.203460
C	0.782486	2.538059	-0.506908
C	0.063911	-0.547768	-0.958770
O	-1.071341	-1.426379	-1.154573
C	-2.282599	-1.267504	-0.559195
C	-2.364118	-0.378064	0.669155
C	2.540499	0.753374	0.025931
C	2.856240	-0.660135	0.538092
C	1.846393	-1.734853	0.082520
O	1.209966	-1.315929	-1.145122
C	2.450198	-3.100258	-0.171182
O	0.833940	-1.949176	1.049070
O	0.338741	-0.653810	1.493479
C	1.798267	3.678165	-0.355726
H	-1.315513	1.191452	1.669255
C	-3.805782	0.053050	0.946325
O	-3.211349	-1.903392	-0.989324
H	0.026268	0.159879	-1.786540

H	1.167965	1.664627	1.421592
H	-2.673463	2.345176	-0.034080
H	-1.777913	1.580309	-1.329600
H	-0.885000	3.857478	-0.889421
H	-0.652688	3.467378	0.808508
H	0.804097	2.210268	-1.555106
H	-2.042612	-1.042798	1.479960
H	2.650800	0.749683	-1.062554
H	3.305187	1.429917	0.412756
H	3.842720	-0.943756	0.163499
H	2.901281	-0.690177	1.628531
H	1.656127	-3.821350	-0.367019
H	3.098718	-3.038906	-1.046031
H	3.035409	-3.419530	0.692114
H	1.485323	4.546632	-0.941556
H	1.873635	3.989490	0.691502
H	2.794791	3.392149	-0.696604
H	-3.846026	0.663676	1.851777
H	-4.218920	0.633036	0.118858
H	-4.441559	-0.820697	1.086150

Nobilisitine A – Method 1

conf #	Boltzmann pop. (%)	Relative Energy (kcal/mol)
3	42.08	0.0
9	41.76	0.0
5	8.6	0.94
17	5.93	1.16
1	0.93	2.26
27	0.31	2.92
7	0.24	3.06
19	0.09	3.66
13	0.06	3.92
20	0.01	4.95
29	0.0	8.06
24	0.0	8.59
21	0.0	9.15
31	0.0	11.32

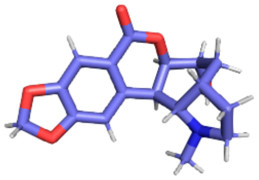
conf 3 (42.08%)

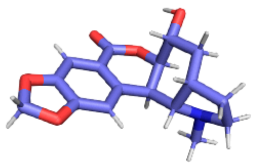
conf 5 (8.60%)

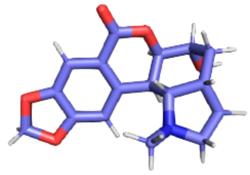
conf 9 (41.76%)

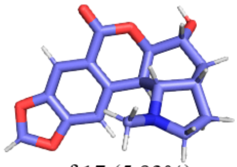
conf 17 (5.93%)

Cartesian Coordinates for Global Minimum of Nobilisitine A (Conf #3)

C	3.199147	1.163046	0.386660
C	2.361322	0.184181	1.261803
C	1.473407	-0.770617	0.434159
C	0.641196	-0.099971	-0.706353
C	1.173779	1.284722	-1.101733
C	2.707881	1.315457	-1.066704
C	-0.839917	-0.017339	-0.399826
C	-1.396753	1.199688	0.019547
C	-0.549745	2.405061	0.215961
O	0.740902	2.334890	-0.202780
C	-1.658332	-1.158708	-0.527740
C	-2.997945	-1.019663	-0.229453

C	-3.544788	0.197265	0.186614
C	-2.772936	1.323769	0.325331
O	-3.976764	-1.971079	-0.291268
C	-5.187138	-1.330034	0.144576
O	-4.893206	0.045417	0.401358
C	3.249721	-0.824379	2.012682
C	3.472682	-1.960889	0.988006
N	2.472112	-1.736519	-0.088674
C	1.927243	-2.981358	-0.615378
O	3.223545	0.320242	-1.938595
H	0.813206	1.535773	-2.105978
H	0.760914	-0.719498	-1.600877
H	0.774234	-1.284010	1.118236
H	1.740079	0.758019	1.955329
O	-0.939361	3.416931	0.758508
H	3.197930	2.145291	0.867861
H	4.244883	0.837809	0.333386
H	3.050145	2.276899	-1.463339
H	-1.255759	-2.111955	-0.854471
H	-3.169930	2.275660	0.656395
H	-5.939883	-1.404323	-0.647681
H	-5.538468	-1.811047	1.065318
H	2.722289	-1.210418	2.892178
H	4.187743	-0.382109	2.362003
H	4.480008	-1.951068	0.555213
H	3.328892	-2.945001	1.458093
H	1.377537	-3.560476	0.149448
H	2.742776	-3.604899	-0.995589
H	1.249652	-2.784550	-1.452194
H	3.135099	-0.537051	-1.459311

Nobilisitine A – Method 2

conf #	Boltzmann pop. (%)	Relative Energy (kcal/mol)
9	22.97	0.0
10	21.81	0.03
3	20.77	0.06
4	12.64	0.35
6	6.43	0.75
5	4.8	0.93
18	4.64	0.95
17	4.47	0.97
2	0.4	2.4
1	0.39	2.41
8	0.16	2.95
7	0.12	3.13
28	0.11	3.18
27	0.08	3.34
19	0.06	3.54
14	0.05	3.61
12	0.03	3.95
13	0.03	3.99
11	0.02	4.13
20	0.01	4.72
23	0.0	6.68
22	0.0	6.85
26	0.0	8.14
24	0.0	8.68
29	0.0	9.01
30	0.0	9.03
21	0.0	9.17
31	0.0	11.64

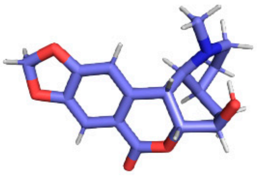
conf 3 (20.77%)

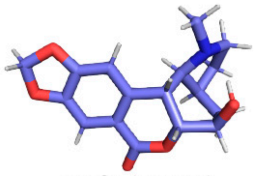
conf 4 (12.64%)

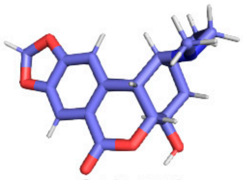
conf 6 (6.43%)

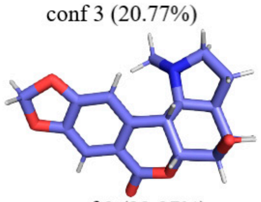
conf 9 (22.97%)

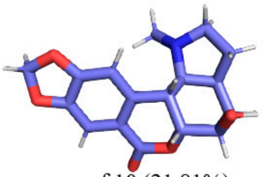
conf 10 (21.81%)

Cartesian Coordinates for Global Minimum of Nobilisitine A (Conf #9)

C	3.553401	-0.838263	-0.354508
C	3.014533	0.558791	-0.682413
C	1.476904	0.689125	-0.639568
C	0.833858	-0.058089	0.566541
C	1.375576	-1.467111	0.757676
C	2.898029	-1.507029	0.853306
C	-0.659515	-0.144622	0.403177
C	-1.185263	-1.270208	-0.248932
C	-0.290911	-2.336864	-0.763222
O	1.006873	-2.319707	-0.357357
C	-1.505101	0.892620	0.828068
C	-2.853784	0.743149	0.587563
C	-3.375685	-0.379620	-0.060195
C	-2.570988	-1.399199	-0.496474
O	-3.860860	1.608809	0.892445
C	-5.086979	0.922276	0.568659
O	-4.735122	-0.246138	-0.186279
C	3.457444	1.744985	0.189177
C	2.494336	2.835542	-0.287342
N	1.212900	2.137689	-0.506472
C	0.391374	2.723683	-1.551945
O	3.198309	-0.852565	2.092190
H	0.955761	-1.903906	1.669615
H	1.079435	0.503433	1.468507
H	1.039033	0.298051	-1.563458
H	3.320263	0.792472	-1.709421
O	-0.641464	-3.199100	-1.536884
H	3.382422	-1.494146	-1.213789
H	4.638227	-0.800247	-0.204193
H	3.206447	-2.559360	0.897023
H	-1.105374	1.775245	1.307457
H	-2.958741	-2.265971	-1.013149
H	-5.586632	0.625275	1.496386
H	-5.713926	1.574202	-0.038891
H	4.504463	2.015065	0.034508
H	3.313862	1.520026	1.247338
H	2.371811	3.652296	0.430135
H	2.871522	3.273491	-1.227497
H	0.867989	2.685838	-2.548928
H	0.191007	3.772575	-1.315800
H	-0.568923	2.207130	-1.607648
H	4.155797	-0.820694	2.186797

Intricarene – Method 1

conf #	Boltzmann pop. (%)	Relative Energy (kcal/mol)
1	50.94	0.0
3	36.95	0.19
2	12.03	0.85
4	0.05	4.1
5	0.03	4.51

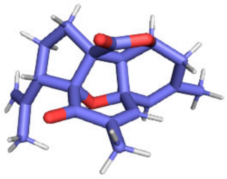

conf 1 (50.94%)

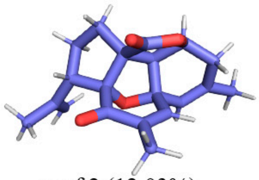

conf 2 (12.03%)

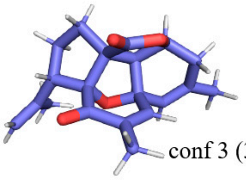

conf 3 (36.95%)

Cartesian Coordinates for Global Minimum of Intricarene (Conf #1)

C	-3.189086	-1.388734	-0.698275
C	-3.468817	-0.344547	0.392564
C	-2.284667	-0.086648	1.370652
C	-1.004543	-0.694004	0.859199
C	-0.908411	-0.596033	-0.692995
C	-1.960141	-1.526753	-1.227203
O	-1.954922	1.322362	1.507641
H	-2.542041	-0.448951	2.371613
C	-0.590703	1.515914	1.535030
C	0.146605	0.185743	1.290333
O	-0.110584	2.595234	1.762334
C	-4.370672	-2.199110	-1.160726
C	1.130776	0.042115	0.016908
C	1.012146	-0.237995	2.493739
C	2.079120	-1.165700	1.900232
C	2.465507	-0.498866	0.566146
C	3.278846	-1.341001	-0.405380
C	3.578355	-0.727749	-1.752450
C	3.757606	-2.544651	-0.074063
H	3.054856	0.404340	0.787433
C	-1.099937	0.792317	-1.313435
C	-0.088924	1.669611	-1.459114
C	1.196839	1.344037	-0.783818

C	-0.191572	2.992534	-2.160336
O	2.174158	2.072151	-0.816165
H	-3.730193	0.616225	-0.071511
H	-4.355794	-0.647523	0.960029
H	-0.897315	-1.749872	1.113400
H	-1.732642	-2.235377	-2.018828
H	-4.094424	-2.893917	-1.959640
H	-4.800105	-2.780417	-0.333364
H	-5.174446	-1.551709	-1.537697
H	1.477158	0.659522	2.918334
H	0.419104	-0.710004	3.285725
H	1.651969	-2.157178	1.706919
H	2.936617	-1.299114	2.567000
H	2.660808	-0.617225	-2.343451
H	4.010584	0.274170	-1.648313
H	4.273484	-1.352695	-2.321429
H	4.371911	-3.111601	-0.768976
H	3.568724	-3.011989	0.886749
H	-2.086667	1.030639	-1.700958
H	0.021050	3.809483	-1.461540
H	-1.186827	3.140735	-2.589425
H	0.552665	3.063763	-2.961886
O	0.475293	-0.936519	-0.836590

Intricarene – Method 2

conf #	Boltzmann pop. (%)	Relative Energy (kcal/mol)
1	55.73	0.0
3	41.47	0.18
2	2.65	1.8
4	0.11	3.7
5	0.05	4.21

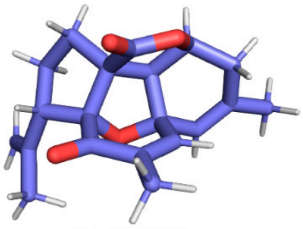

conf 1 (55.73%)

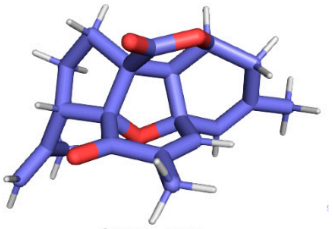

conf 3 (41.47%)

Cartesian Coordinates for Global Minimum of Intricarene (Conf #1)

C	-3.181692	-1.380102	-0.690250
C	-3.453597	-0.330262	0.394018
C	-2.272904	-0.077976	1.369070
C	-0.999321	-0.691126	0.860984
C	-0.905625	-0.600956	-0.689211
C	-1.958520	-1.527514	-1.220164
O	-1.927227	1.339062	1.494934
H	-2.524716	-0.424096	2.372138
C	-0.571465	1.517565	1.521628
C	0.152276	0.184411	1.287215
O	-0.080528	2.592610	1.731884
C	-4.371995	-2.175990	-1.145111
C	1.133682	0.033729	0.017902
C	1.007830	-0.247155	2.489176
C	2.052185	-1.193745	1.890844
C	2.457393	-0.514607	0.571968
C	3.260959	-1.341985	-0.412961
C	3.524483	-0.706428	-1.754912
C	3.742057	-2.547455	-0.105322
H	3.052045	0.377699	0.809419
C	-1.091397	0.785190	-1.311098
C	-0.080934	1.658055	-1.456015
C	1.199146	1.336601	-0.775074
C	-0.184724	2.983306	-2.147091
O	2.165208	2.073883	-0.779733
H	-3.701092	0.624871	-0.082334

H	-4.342019	-0.619320	0.958918
H	-0.893159	-1.741989	1.121562
H	-1.732840	-2.232316	-2.011685
H	-4.110227	-2.872209	-1.943757
H	-4.798227	-2.745501	-0.311896
H	-5.164373	-1.513893	-1.512117
H	1.493845	0.638864	2.907629
H	0.404057	-0.704196	3.276469
H	1.599924	-2.167353	1.679806
H	2.900997	-1.356300	2.556571
H	2.589661	-0.603459	-2.314725
H	3.935145	0.300254	-1.638209
H	4.215776	-1.306081	-2.349529
H	4.344345	-3.103319	-0.816182
H	3.563880	-3.022240	0.851359
H	-2.077650	1.029278	-1.687986
H	0.038489	3.790942	-1.444288
H	-1.180573	3.138123	-2.564391
H	0.551599	3.053831	-2.952634
O	0.478558	-0.945921	-0.831800

Strychnine – Method 1

conf #	Boltzmann pop. (%)	Relative Energy (kcal/mol)
1	97.42	0.0
2	2.25	2.23
3	0.33	3.37

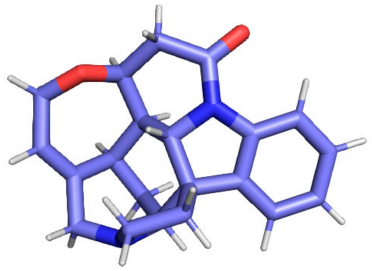

conf 1 (97.42%)

Cartesian Coordinates for Global Minimum of Strychnine (Conf #1)

C	4.306884	-1.417078	-0.511489
C	4.636200	-0.068869	-0.353024
C	3.670977	0.881507	-0.004791
C	2.358992	0.438616	0.172133
C	2.012794	-0.912965	0.023167
C	2.988483	-1.844524	-0.315640
N	1.208793	1.197792	0.488302
C	0.001091	0.335945	0.415113
C	0.553207	-1.128282	0.365125
C	1.081753	2.562686	0.313788
C	-0.358950	3.075342	0.317333
C	-1.419962	2.203583	-0.412224
C	-0.813755	0.836168	-0.792896
C	-1.739454	-0.243125	-1.393285
C	-0.819938	-1.405188	-1.817451
C	-0.269083	-2.078828	-0.557930
N	-1.368486	-2.618712	0.292619
C	-1.099563	-2.280977	1.702659
C	0.365580	-1.839147	1.730282
O	-2.523226	2.141963	0.487915
C	-3.713931	1.536234	-0.022064
C	-3.716722	0.034899	0.139098
C	-2.820920	-0.767451	-0.447777
O	2.038364	3.317216	0.193633
C	-2.721323	-2.250670	-0.154942
H	-0.600725	0.478768	1.318581

H	-0.086050	1.061386	-1.586932
H	-1.748851	2.706883	-1.336060
H	5.074693	-2.136924	-0.780646
H	5.662884	0.255189	-0.501658
H	3.914297	1.928259	0.113849
H	2.733642	-2.895872	-0.425762
H	-0.686440	3.148237	1.362062
H	-0.326804	4.092678	-0.077106
H	-2.223197	0.186816	-2.283604
H	0.010253	-1.034397	-2.429565
H	-1.357670	-2.137306	-2.429769
H	0.389075	-2.908640	-0.853598
H	-1.286690	-3.144151	2.354022
H	-1.751124	-1.462114	2.050067
H	1.032238	-2.708087	1.774394
H	0.605518	-1.192029	2.580058
H	-3.853295	1.827473	-1.077007
H	-4.533581	1.981393	0.549506
H	-4.459281	-0.393092	0.811571
H	-2.973599	-2.842961	-1.044962
H	-3.432700	-2.545743	0.622614

Strychnine – Method 2

conf #	Boltzmann pop. (%)	Relative Energy (kcal/mol)
1	98.16	0.0
2	1.57	2.45
3	0.27	3.5

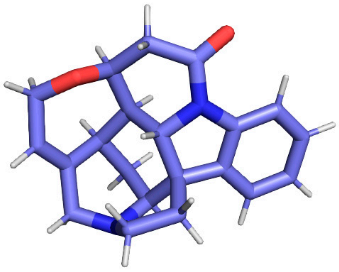

conf 1 (98.16%)

Cartesian Coordinates for Global Minimum of Strychnine (Conf #1)

C	4.288261	-1.423845	-0.520447
C	4.625693	-0.080431	-0.357073
C	3.666293	0.872361	-0.004339
C	2.353213	0.438832	0.172308
C	2.002879	-0.908410	0.020282
C	2.968819	-1.843214	-0.324089
N	1.205961	1.199764	0.488280
C	-0.004056	0.337900	0.413298
C	0.549642	-1.121065	0.366341
C	1.079509	2.558866	0.318491
C	-0.356324	3.068699	0.309915
C	-1.411051	2.197997	-0.422932
C	-0.807883	0.832513	-0.797978
C	-1.734225	-0.246928	-1.390116
C	-0.814651	-1.408632	-1.810381
C	-0.265743	-2.076500	-0.549945
N	-1.362886	-2.614935	0.307680
C	-1.097921	-2.243741	1.712266
C	0.369921	-1.821375	1.734024
O	-2.516436	2.131980	0.478025
C	-3.708657	1.525626	-0.040621
C	-3.708404	0.029911	0.139599
C	-2.812493	-0.768637	-0.441928
O	2.035420	3.313787	0.206708
C	-2.715120	-2.248629	-0.146139
H	-0.608385	0.482027	1.310830
H	-0.079619	1.050785	-1.589623

H	-1.736847	2.699440	-1.344255
H	5.049846	-2.145014	-0.793251
H	5.651630	0.237702	-0.504773
H	3.918543	1.913672	0.120335
H	2.705027	-2.889082	-0.434745
H	-0.682204	3.132864	1.353003
H	-0.326746	4.084281	-0.080762
H	-2.222574	0.179286	-2.274977
H	0.015796	-1.039162	-2.417623
H	-1.348128	-2.140533	-2.420716
H	0.392555	-2.903889	-0.836838
H	-1.301547	-3.083836	2.382246
H	-1.736953	-1.405956	2.029205
H	1.025460	-2.695796	1.772677
H	0.621684	-1.171330	2.574226
H	-3.829668	1.799048	-1.099431
H	-4.532088	1.979045	0.512864
H	-4.443436	-0.393787	0.818493
H	-2.962853	-2.836812	-1.036092
H	-3.429086	-2.540681	0.626387

Holstiine – Method 1

conf #	Boltzmann pop. (%)	Relative Energy (kcal/mol)
8	98.89	0.0
1	0.73	2.91
10	0.37	3.3
22	0.0	5.9
3	0.0	7.05
2	0.0	7.42
7	0.0	7.93
12	0.0	8.4
6	0.0	10.81
26	0.0	11.2
21	0.0	11.27
25	0.0	12.55
29	0.0	12.81
27	0.0	13.07

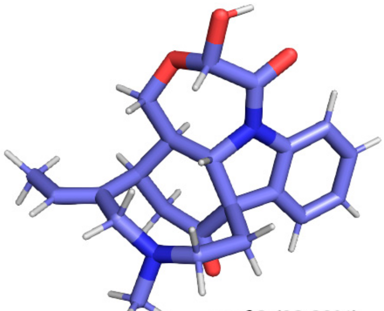

conf 8 (98.89%)

Cartesian Coordinates for Global Minimum of Holstiine (Conf #8)

C	3.415742	-3.034805	-1.025862
C	4.274716	-1.933418	-1.010372
C	3.833354	-0.670848	-0.601801
C	2.498827	-0.548513	-0.210689
C	1.631969	-1.646076	-0.213124
C	2.083552	-2.896238	-0.621246
N	1.802939	0.608193	0.222407
C	0.337761	0.324316	0.313902
C	0.263123	-1.244684	0.328356
C	2.359447	1.831080	0.435246
C	1.399172	3.006620	0.750637
C	-0.583894	2.561395	-0.526386
C	-0.427493	1.058116	-0.818695
C	-1.794240	0.429053	-1.215487
C	-1.556265	-1.049576	-1.584391
C	-0.847926	-1.881291	-0.533408
N	-2.288562	-1.095002	1.386066
C	-1.244821	-1.285435	2.400217

C	0.077630	-1.788443	1.770546
C	-4.614652	1.566138	-2.023452
C	-4.166413	1.102065	-0.666066
C	-2.972578	0.632006	-0.262523
O	3.562132	2.073967	0.328313
C	-2.802041	0.265592	1.202826
H	-0.007646	0.703331	1.277280
H	0.202666	0.973847	-1.714930
O	-1.019759	-3.091678	-0.483058
C	-3.320128	-2.122672	1.367736
O	0.640349	3.270373	-0.429336
O	2.150199	4.104749	1.087421
H	3.781678	-4.004461	-1.351406
H	5.308585	-2.051433	-1.323464
H	4.492897	0.185084	-0.584884
H	1.402164	-3.740631	-0.629300
H	0.706188	2.777250	1.575859
H	-1.129484	3.034909	-1.348641
H	-1.176384	2.711283	0.391709
H	-2.065748	0.932755	-2.148106
H	-2.487562	-1.562271	-1.841774
H	-0.919305	-1.084155	-2.482649
H	-1.074397	-0.337112	2.923265
H	-1.579309	-1.994514	3.169275
H	0.076819	-2.879067	1.700086
H	0.929759	-1.501182	2.398259
H	-5.448670	0.948053	-2.382320
H	-4.993046	2.595622	-1.969791
H	-3.831832	1.537688	-2.785394
H	-4.946553	1.165895	0.094980
H	-3.759586	0.412544	1.730648
H	-2.094015	0.957993	1.672739
H	-4.007817	-1.929985	0.538444
H	-3.905080	-2.150657	2.303964
H	-2.862686	-3.102059	1.199453
H	3.055315	3.903364	0.764868

Holstiine – Method 2

conf #	Boltzmann pop. (%)	Relative Energy (kcal/mol)
8	96.91	0.0
10	1.91	2.33
1	1.14	2.63
22	0.03	4.85
20	0.01	5.64
3	0.0	6.24
13	0.0	6.68
2	0.0	7.28
7	0.0	7.62
11	0.0	7.71
6	0.0	10.35
21	0.0	10.59
26	0.0	11.86
25	0.0	12.1
29	0.0	13.0
27	0.0	13.21

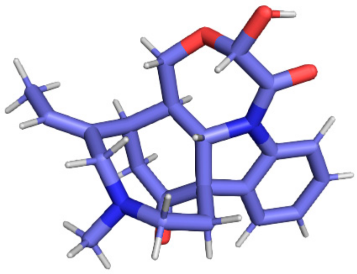

conf 8 (96.91%)

Cartesian Coordinates for Global Minimum of Holstiine (Conf #8)

C	3.381266	-3.036451	-1.040060
C	4.242892	-1.939601	-1.039848
C	3.810407	-0.676518	-0.628405
C	2.483882	-0.547407	-0.220609
C	1.615653	-1.640518	-0.210856
C	2.056703	-2.890917	-0.618746
N	1.797054	0.608944	0.220407
C	0.330323	0.329028	0.311020
C	0.259994	-1.234718	0.346353
C	2.354192	1.824700	0.439806
C	1.381570	2.992061	0.741003
C	-0.585059	2.542237	-0.571524
C	-0.421404	1.039742	-0.837893
C	-1.785930	0.410626	-1.222279

C	-1.564649	-1.075704	-1.550719
C	-0.862776	-1.882915	-0.484622
N	-2.249578	-1.067803	1.380561
C	-1.225676	-1.258315	2.415971
C	0.106133	-1.750250	1.798050
C	-4.594185	1.515732	-2.036550
C	-4.146442	1.092407	-0.668077
C	-2.951787	0.638208	-0.265417
O	3.553378	2.069307	0.343399
C	-2.754668	0.297571	1.197998
H	-0.015848	0.717397	1.265082
H	0.213469	0.937518	-1.725534
O	-1.026886	-3.091129	-0.414886
C	-3.295891	-2.080008	1.359527
O	0.647125	3.250710	-0.454253
O	2.114485	4.105595	1.088133
H	3.739228	-4.006135	-1.365707
H	5.269533	-2.061577	-1.365835
H	4.472999	0.174203	-0.622263
H	1.372342	-3.729483	-0.610225
H	0.683156	2.758636	1.555254
H	-1.106956	3.003679	-1.411203
H	-1.193720	2.707085	0.328600
H	-2.054748	0.892513	-2.163207
H	-2.501813	-1.582166	-1.786845
H	-0.928559	-1.147621	-2.443782
H	-1.066983	-0.314937	2.944637
H	-1.569050	-1.973306	3.169875
H	0.131983	-2.839453	1.755377
H	0.954580	-1.423334	2.405689
H	-5.428744	0.890119	-2.371897
H	-4.967430	2.545303	-2.012976
H	-3.812609	1.459007	-2.794255
H	-4.920754	1.171218	0.093898
H	-3.694636	0.455322	1.746936
H	-2.031890	0.989091	1.637366
H	-3.955738	-1.898484	0.508384
H	-3.901244	-2.072080	2.279040
H	-2.846981	-3.066224	1.230456
H	3.015970	3.935909	0.762476

Colchicine – Method 1

conf #	Boltzmann pop. (%)	Relative Energy (kcal/mol)
2	34.33	0.0
5	10.27	0.71
9	8.54	0.82
1	7.97	0.86
11	7.85	0.87
7	4.7	1.18
15	3.23	1.4
21	2.9	1.46
19	2.06	1.67
18	1.63	1.81
10	1.61	1.81
3	1.28	1.95
33	1.19	1.99
12	1.11	2.03
6	0.97	2.11
39	0.96	2.12
4	0.88	2.17
32	0.79	2.23
48	0.74	2.27
61	0.71	2.29
36	0.66	2.34
22	0.47	2.54
75	0.43	2.59
35	0.39	2.66
34	0.38	2.67
8	0.31	2.78
26	0.3	2.81
14	0.25	2.92
24	0.24	2.93
20	0.23	2.98
51	0.22	2.99
42	0.21	3.02
16	0.18	3.1
29	0.15	3.21
25	0.15	3.24
115	0.12	3.35
38	0.12	3.37
67	0.11	3.42
23	0.1	3.45
55	0.09	3.51
73	0.09	3.52
17	0.09	3.55
66	0.08	3.56
68	0.08	3.57
30	0.06	3.73

40	0.05	3.89
63	0.05	3.89
72	0.04	3.96
81	0.04	3.97
99	0.04	4.01
50	0.04	4.01
41	0.04	4.07
53	0.03	4.09
46	0.03	4.11
44	0.03	4.13
52	0.03	4.15
124	0.03	4.16
45	0.02	4.3
49	0.02	4.31
71	0.02	4.37
47	0.02	4.44
100	0.02	4.46
91	0.02	4.49
64	0.02	4.51
101	0.02	4.53
107	0.02	4.54
94	0.01	4.61
83	0.01	4.67
104	0.01	4.7
70	0.01	4.81
121	0.01	4.87
31	0.01	4.9
116	0.01	4.9
110	0.01	4.99
86	0.01	5.04
112	0.01	5.07
78	0.01	5.11
84	0.01	5.22
77	0.0	5.25
133	0.0	5.36
113	0.0	5.36
125	0.0	5.37
134	0.0	5.41
59	0.0	5.49
137	0.0	5.6
58	0.0	5.65
132	0.0	5.67
62	0.0	5.72
136	0.0	5.77
135	0.0	5.84
93	0.0	5.94
60	0.0	5.97
65	0.0	6.13
87	0.0	6.23
90	0.0	6.31

144	0.0	6.55
89	0.0	6.55
127	0.0	6.61
88	0.0	6.61
96	0.0	6.67
85	0.0	6.69
98	0.0	6.75
103	0.0	6.83
126	0.0	7.04
102	0.0	7.09
128	0.0	7.09
122	0.0	7.11
123	0.0	7.3
129	0.0	7.39
130	0.0	7.69
141	0.0	8.02
143	0.0	8.35
118	0.0	9.4
142	0.0	10.77

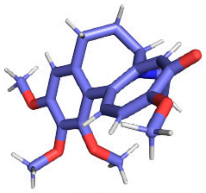
conf 1 (7.97%)

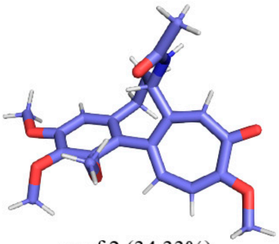
conf 2 (34.33%)

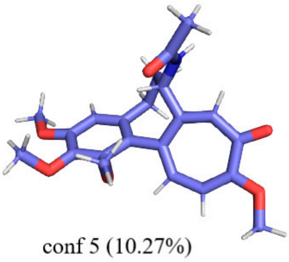
conf 5 (10.27%)

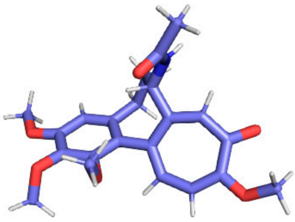
conf 9 (8.54%)

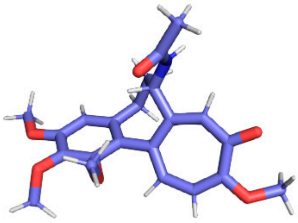
conf 11 (7.85%)

Cartesian Coordinates for Global Minimum of Colchicine (Conf #2)

C	-0.660294	1.155268	-2.442247
C	-1.535543	0.411441	-1.456497
C	-0.927372	-0.357705	-0.443063
C	0.558464	-0.542935	-0.408559
C	1.405322	0.629885	-0.440638
C	0.652622	1.974799	-0.393661
C	0.072331	2.342620	-1.775046
C	-2.926817	0.538921	-1.512713
C	-3.745253	-0.079499	-0.562001
C	-3.150908	-0.806952	0.485986

C	-1.759212	-0.947567	0.532495
C	1.022987	-1.837773	-0.347502
C	2.337681	-2.376582	-0.319969
C	3.562467	-1.766338	-0.431970
C	3.836215	-0.315275	-0.619054
C	2.768460	0.674200	-0.530414
O	-3.937102	-1.319582	1.488270
C	-4.237890	-2.710496	1.353811
O	-5.105874	-0.029250	-0.551007
C	-5.754863	0.726515	-1.560996
O	-1.215107	-1.683396	1.560065
C	-1.012946	-0.927351	2.766481
O	4.996033	0.059503	-0.810773
O	4.724296	-2.449208	-0.400849
C	4.719696	-3.854405	-0.197074
N	1.421086	3.084826	0.139338
H	1.981732	3.643167	-0.488770
C	1.521465	3.297274	1.491336
C	2.431038	4.442499	1.907411
O	0.926266	2.606978	2.310009
H	-1.259399	1.527419	-3.280279
H	0.084591	0.470883	-2.865521
H	-0.176598	1.846323	0.304683
H	0.879900	2.682022	-2.439488
H	-0.616305	3.185611	-1.639754
H	-3.364856	1.125482	-2.312920
H	0.250184	-2.598500	-0.299600
H	2.350358	-3.457230	-0.219446
H	3.213554	1.665634	-0.542846
H	-4.866327	-2.970295	2.209073
H	-4.791034	-2.903985	0.426006
H	-3.322839	-3.313231	1.374475
H	-6.823592	0.638215	-1.357680
H	-5.464838	1.784772	-1.522237
H	-5.542152	0.331055	-2.563263
H	-0.531248	-1.608063	3.471964
H	-0.362133	-0.063421	2.584910
H	-1.972799	-0.591240	3.173018
H	5.769726	-4.149906	-0.179992
H	4.247830	-4.119708	0.757510
H	4.206731	-4.377536	-1.014323
H	2.669687	5.131285	1.090556
H	1.950709	4.995311	2.718409
H	3.367519	4.028540	2.297728

Colchicine – Method 2

conf #	Boltzmann pop. (%)	Relative Energy (kcal/mol)
2	22.21	0.0
9	10.91	0.42
7	10.51	0.44
3	8.01	0.6
1	6.67	0.71
11	6.15	0.76
18	3.76	1.05
4	3.01	1.18
5	2.31	1.34
42	2.16	1.38
6	2.13	1.39
48	2.09	1.4
32	2.08	1.4
21	2.06	1.41
12	2.0	1.43
20	1.6	1.56
15	1.31	1.67
54	1.19	1.73
61	1.15	1.75
19	1.13	1.77
40	0.97	1.85
35	0.69	2.05
33	0.67	2.07
39	0.61	2.13
36	0.45	2.31
23	0.38	2.4
25	0.34	2.47
51	0.29	2.56
67	0.28	2.58
73	0.26	2.63
14	0.23	2.7
10	0.23	2.71
38	0.16	2.94
68	0.14	3.02
46	0.13	3.02
34	0.13	3.05
52	0.12	3.08
55	0.1	3.19
75	0.08	3.37
22	0.08	3.37
30	0.07	3.42
81	0.07	3.45
63	0.06	3.48
120	0.06	3.49
29	0.06	3.51
76	0.05	3.64
26	0.05	3.65

17	0.05	3.65
53	0.04	3.68
115	0.04	3.72
72	0.04	3.75
66	0.04	3.78
16	0.04	3.79
100	0.03	3.83
47	0.03	3.86
91	0.03	3.88
124	0.03	3.9
125	0.03	3.96
114	0.03	3.96
110	0.03	3.98
92	0.03	4.01
50	0.02	4.06
41	0.02	4.12
44	0.02	4.2
116	0.02	4.21
107	0.02	4.21
86	0.02	4.27
71	0.02	4.3
31	0.01	4.32
45	0.01	4.44
49	0.01	4.45
64	0.01	4.5
121	0.01	4.51
83	0.01	4.54
77	0.01	4.7
99	0.01	4.75
78	0.01	4.79
69	0.01	4.8
133	0.01	4.82
106	0.01	4.88
84	0.01	4.97
104	0.0	5.1
59	0.0	5.13
58	0.0	5.13
132	0.0	5.24
62	0.0	5.24
70	0.0	5.27
94	0.0	5.43
93	0.0	5.44
112	0.0	5.48
101	0.0	5.51
136	0.0	5.59
60	0.0	5.61
65	0.0	5.78
113	0.0	5.8
87	0.0	5.82
137	0.0	5.89

134	0.0	5.92
85	0.0	5.95
96	0.0	5.97
122	0.0	6.18
102	0.0	6.19
144	0.0	6.19
135	0.0	6.2
103	0.0	6.3
90	0.0	6.38
126	0.0	6.45
88	0.0	6.46
139	0.0	6.48
145	0.0	6.51
89	0.0	6.52
123	0.0	6.83
129	0.0	6.99
128	0.0	6.99
149	0.0	7.1
98	0.0	7.13
141	0.0	7.27
150	0.0	7.44
131	0.0	7.54
138	0.0	7.57
130	0.0	7.64
146	0.0	7.68
148	0.0	7.91
147	0.0	8.01
143	0.0	8.35
118	0.0	9.21
142	0.0	10.44

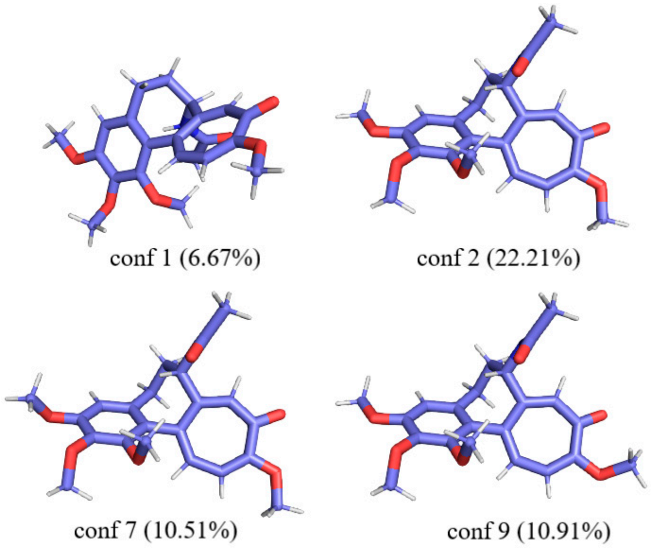

Cartesian Coordinates for Global Minimum of Colchicine (Conf #2)

C	-0.694940	1.173227	-2.445766
C	-1.559521	0.406529	-1.471268
C	-0.940150	-0.373824	-0.478884
C	0.544759	-0.541117	-0.445552
C	1.365976	0.641665	-0.450602
C	0.588338	1.968134	-0.376794
C	0.016112	2.357643	-1.753176
C	-2.948666	0.538558	-1.500158
C	-3.750396	-0.083056	-0.539143
C	-3.138798	-0.815731	0.493558
C	-1.751826	-0.969937	0.502802
C	1.028687	-1.826708	-0.398106
C	2.349210	-2.335617	-0.362183
C	3.561942	-1.700825	-0.460642
C	3.805311	-0.247773	-0.653267
C	2.726673	0.716752	-0.540344
O	-3.903594	-1.328201	1.510509
C	-4.155133	-2.738537	1.408043
O	-5.106773	-0.025715	-0.500215
C	-5.781774	0.730176	-1.504975
O	-1.190704	-1.711320	1.516333
C	-0.851357	-0.921562	2.673405
O	4.956377	0.147416	-0.862513
O	4.731933	-2.361194	-0.419183
C	4.756819	-3.774566	-0.209518
N	1.342472	3.071755	0.187248
H	1.798421	3.725516	-0.429763
C	1.516924	3.190613	1.534871
C	2.379268	4.350543	1.990664
O	1.021968	2.399430	2.328575
H	-1.294069	1.548705	-3.277514
H	0.059048	0.504939	-2.871741
H	-0.244907	1.800053	0.306353
H	0.824313	2.717852	-2.399621
H	-0.684705	3.184368	-1.604248
H	-3.398240	1.134020	-2.283277
H	0.270734	-2.599962	-0.365502
H	2.385654	-3.413466	-0.267678
H	3.145252	1.717728	-0.536871
H	-4.771566	-3.003376	2.266731
H	-4.698296	-2.967600	0.485333
H	-3.217631	-3.299926	1.440644
H	-6.842457	0.636624	-1.280438
H	-5.494787	1.786303	-1.469771
H	-5.584963	0.330650	-2.505458
H	-0.375397	-1.600950	3.379488
H	-0.157675	-0.117088	2.407785

H	-1.756494	-0.498902	3.118862
H	5.810467	-4.043070	-0.182815
H	4.284821	-4.042211	0.740811
H	4.264995	-4.305521	-1.030398
H	2.539233	5.105867	1.219315
H	1.912424	4.810764	2.861900
H	3.350104	3.955549	2.301290

Hexacyclinol – Method 1

conf #	Boltzmann pop. (%)	Relative Energy (kcal/mol)
7	38.93	0.0
8	22.01	0.34
5	14.2	0.6
2	12.99	0.65
3	4.72	1.25
1	2.33	1.67
39	1.98	1.76
24	1.08	2.12
11	0.54	2.54
29	0.44	2.66
21	0.35	2.79
25	0.16	3.24
16	0.13	3.4
32	0.05	3.98
9	0.04	4.03
17	0.04	4.14
48	0.01	5.16
50	0.0	5.31
40	0.0	5.5
30	0.0	5.78
43	0.0	5.98
45	0.0	6.46
53	0.0	7.02
47	0.0	7.14
49	0.0	7.64
46	0.0	7.76
42	0.0	8.37
51	0.0	9.44

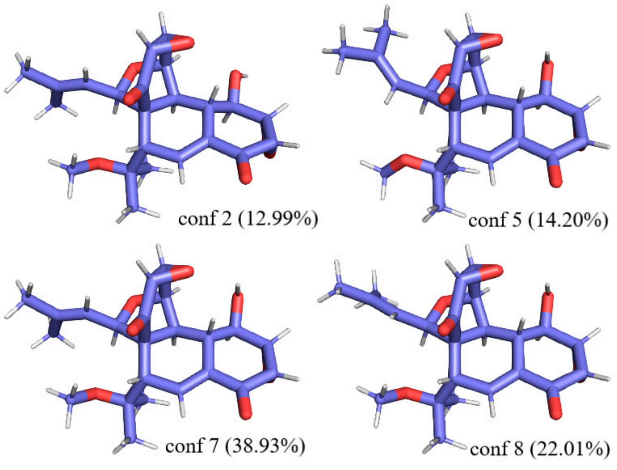

Cartesian Coordinates for Global Minimum of Hexacyclinol (Conf #7)

C	-0.573028	-0.751724	-0.663567
C	0.552650	-0.160036	0.224269
C	0.345561	1.356803	0.513972
C	-1.122258	1.634088	0.731659
C	-2.156860	0.822496	0.448925
C	-1.981772	-0.592194	-0.076223
C	-3.525499	1.270769	0.847994
C	-4.688175	0.544646	0.222420
C	-4.461971	-0.573086	-0.718965
C	-3.062031	-1.075034	-1.065499
C	0.026616	-2.128205	-0.988530
O	1.375848	-1.813357	-1.349152
C	1.820426	-0.632879	-0.601735
C	0.123282	-3.064914	0.208803
C	0.573654	-0.926712	1.573016
C	0.417948	-2.441780	1.507826
O	-0.891182	-2.990512	1.221641
O	0.820765	-0.402944	2.641456
C	0.978116	2.445857	-0.441848
O	2.402923	2.268495	-0.525443
C	3.183240	2.567531	0.621902
C	0.494800	2.356353	-1.895545
C	0.653408	3.859226	0.086244
C	3.071190	-0.971949	0.160574
C	4.334415	-0.756311	-0.242957
C	5.493524	-1.237921	0.596752
C	4.735242	-0.075980	-1.528766
H	-0.560864	-0.197910	-1.609929
O	-3.040175	-2.497733	-1.170083
O	-4.759410	0.734448	-1.207914
O	-3.736399	2.193110	1.620929
H	-2.089272	-1.244924	0.799003
H	0.815165	1.541272	1.488573
H	-1.375218	2.581139	1.200782
H	-5.635017	0.656032	0.748943
H	-5.256324	-1.307438	-0.861923
H	-2.842412	-0.707967	-2.075315
H	-0.448635	-2.617648	-1.840658
H	2.039535	0.133574	-1.338312
H	0.496490	-4.068423	0.007864
H	0.952475	-2.972543	2.293915
H	4.214544	2.331349	0.350582
H	3.128150	3.629003	0.895787
H	2.909888	1.957994	1.493134
H	0.904083	3.196740	-2.465203
H	-0.597300	2.403475	-1.944635
H	0.825778	1.437609	-2.386096
H	-0.385137	4.130814	-0.120479
H	0.817212	3.956208	1.164779
H	1.295964	4.581925	-0.427457

H	2.941069	-1.511976	1.097255
H	5.160439	-1.746467	1.506607
H	6.124987	-1.934202	0.027628
H	6.142150	-0.400940	0.890363
H	3.908416	0.425679	-2.032973
H	5.516358	0.672110	-1.340046
H	5.167925	-0.808728	-2.223916
H	-2.944291	-2.862080	-0.274074

Hexacyclinol – Method 2

conf #	Boltzmann pop. (%)	Relative Energy (kcal/mol)
7	56.85	0.0
2	11.7	0.94
24	7.58	1.19
5	6.52	1.28
8	4.56	1.49
27	2.71	1.8
6	2.37	1.88
11	1.93	2.0
1	1.85	2.03
3	1.49	2.16
29	0.62	2.67
31	0.58	2.72
39	0.36	3.0
16	0.24	3.23
22	0.18	3.39
18	0.15	3.53
25	0.1	3.77
10	0.09	3.84
35	0.03	4.39
20	0.02	4.7
21	0.01	4.94
32	0.01	4.98
9	0.01	4.98
17	0.01	5.5
50	0.0	5.54
37	0.0	5.78
40	0.0	6.04
48	0.0	6.05
34	0.0	6.19
41	0.0	6.2
30	0.0	6.33
43	0.0	6.56
45	0.0	6.6
54	0.0	7.05
53	0.0	7.13
46	0.0	7.95
47	0.0	7.96
49	0.0	8.02
44	0.0	8.64
42	0.0	8.91
51	0.0	9.55

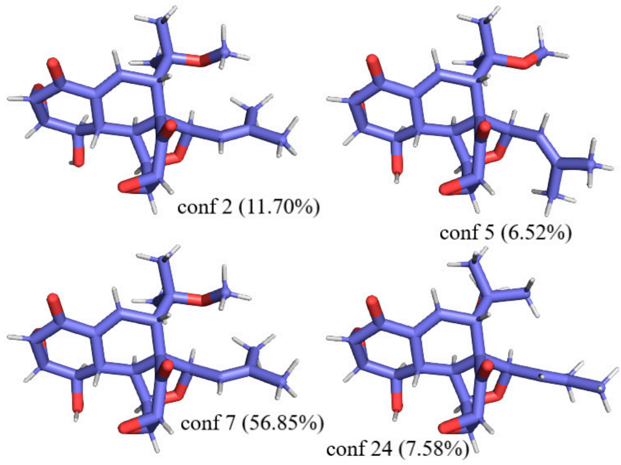

Cartesian Coordinates for Global Minimum of Hexacyclinol (Conf #7)

C	-0.573133	-0.751521	-0.668885
C	0.555849	-0.185575	0.223861
C	0.369318	1.325450	0.519168
C	-1.085859	1.611222	0.775409
C	-2.126449	0.815188	0.490147
C	-1.976272	-0.579214	-0.086086
C	-3.485243	1.249908	0.922879
C	-4.654774	0.572789	0.260285
C	-4.440960	-0.487900	-0.742523
C	-3.048775	-0.984846	-1.110088
C	0.000669	-2.132125	-1.005541
O	1.359216	-1.836246	-1.358884
C	1.812482	-0.657364	-0.606289
C	0.083809	-3.079030	0.181373
C	0.566983	-0.963262	1.562180
C	0.403039	-2.473775	1.479871
O	-0.920485	-2.987893	1.202479
O	0.792221	-0.450841	2.635200
C	0.979470	2.399374	-0.458956
O	2.403471	2.219414	-0.565945
C	3.195998	2.460206	0.590974
C	0.469840	2.283617	-1.897291
C	0.668756	3.816286	0.059801
C	3.056486	-0.997390	0.154568
C	4.310347	-0.704924	-0.213249
C	5.480663	-1.166887	0.616552
C	4.689451	0.058015	-1.456965
H	-0.549845	-0.189486	-1.605465
O	-3.039407	-2.399408	-1.301910
O	-4.720359	0.850498	-1.158135
O	-3.687301	2.122184	1.748978
H	-2.114320	-1.260612	0.758423

H	0.865918	1.515443	1.475453
H	-1.316129	2.548282	1.269681
H	-5.597407	0.661408	0.792384
H	-5.241561	-1.201732	-0.923560
H	-2.815200	-0.557498	-2.089522
H	-0.486697	-2.600804	-1.858115
H	2.027384	0.106624	-1.341245
H	0.434236	-4.084944	-0.030348
H	0.940399	-3.016915	2.251471
H	4.225602	2.263530	0.295385
H	3.121566	3.496819	0.934421
H	2.945036	1.785062	1.415625
H	0.856255	3.120314	-2.482469
H	-0.621032	2.313964	-1.922857
H	0.805788	1.363953	-2.375532
H	-0.366308	4.091541	-0.146212
H	0.838328	3.915916	1.134067
H	1.316948	4.523379	-0.462148
H	2.928688	-1.594329	1.053771
H	5.169230	-1.765362	1.474651
H	6.170963	-1.763268	0.009208
H	6.051566	-0.306201	0.983835
H	3.865810	0.632551	-1.875806
H	5.508491	0.751298	-1.241829
H	5.058219	-0.636921	-2.219926
H	-3.030955	-2.823121	-0.434781

Homodimericin A – Method 1

conf #	Boltzmann pop. (%)	Relative Energy (kcal/mol)
1	93.41	0.0
3	4.17	1.84
4	0.95	2.72
6	0.68	2.91
8	0.57	3.02
12	0.14	3.86
17	0.03	4.86
27	0.01	5.23
16	0.01	5.4
18	0.01	5.54
13	0.01	5.61
25	0.01	5.63
19	0.0	6.06
24	0.0	6.13
29	0.0	7.27
30	0.0	7.31
28	0.0	7.67
26	0.0	8.0
73	0.0	8.31
39	0.0	8.33
33	0.0	8.43
41	0.0	8.46
48	0.0	8.99
36	0.0	9.17
43	0.0	9.78
59	0.0	10.09
46	0.0	10.2
49	0.0	10.38
63	0.0	10.79
50	0.0	10.84
61	0.0	10.94
53	0.0	10.97
64	0.0	11.01
54	0.0	11.43
56	0.0	11.62
67	0.0	12.36
69	0.0	12.57
70	0.0	13.03
77	0.0	14.62

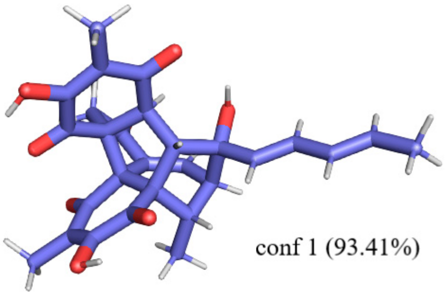

conf 1 (93.41%)

Cartesian Coordinates for Global Minimum of Homodimericin A (Conf #1)

C	2.086558	2.796111	-0.627908
C	0.908340	3.434672	-0.381211
C	-0.139253	2.718454	0.358316
C	0.081204	1.282944	0.798720
C	1.528558	0.723345	0.832820
C	2.447652	1.436401	-0.130683
C	-0.778791	0.322241	-0.091524
C	0.122593	-0.902071	-0.447144
C	1.261300	-0.826676	0.581438
C	-2.037090	-0.271155	0.673139
C	-1.518092	-1.705736	1.083254
C	-0.800911	-2.157475	-0.198383
C	0.615228	-0.925642	-1.869498
C	1.961293	-1.483008	-2.160393
C	2.848902	-1.853703	-1.197692
C	2.533606	-1.606868	0.223904
O	-0.082672	-0.591119	-2.826676
C	-0.136743	-3.535005	-0.136541
O	2.227880	-1.596084	-3.473897
C	4.189005	-2.444933	-1.513661
O	3.288129	-1.980736	1.113761
O	3.548834	1.004563	-0.467559
O	-1.237338	3.226689	0.585105
O	3.052586	3.372316	-1.357168
C	0.641619	4.822105	-0.886810
C	2.179184	0.745559	2.252704
C	1.553923	-0.458669	2.990547
C	0.746081	-1.182865	1.971536
C	-0.521424	-1.555229	2.209030
O	1.631809	-0.667194	4.180461
H	-1.114607	0.828367	-0.998950
O	-2.366124	0.444711	1.852974
C	-3.228798	-0.347376	-0.259015
C	-4.503091	-0.176700	0.134817
C	-5.652306	-0.237963	-0.752863
C	-6.922606	-0.071249	-0.346476

C	-8.129113	-0.131507	-1.233002
H	-0.332198	1.241662	1.808292
H	-2.352608	-2.355331	1.361830
H	-1.528468	-2.174580	-1.016718
H	0.339097	-3.797030	-1.086986
H	-0.899640	-4.295165	0.067291
H	0.616283	-3.605609	0.653109
H	1.420284	-1.264809	-3.927223
H	4.247010	-2.769759	-2.554089
H	4.974159	-1.699724	-1.333048
H	4.396899	-3.286629	-0.846230
H	3.785591	2.715131	-1.381272
H	1.466526	5.496848	-0.633767
H	0.551444	4.827060	-1.980600
H	-0.289307	5.199286	-0.460210
H	3.261968	0.598249	2.184413
H	1.989288	1.672586	2.802328
H	-0.873279	-1.595441	3.236642
H	-2.534327	1.369836	1.587925
H	-3.016572	-0.541030	-1.309937
H	-4.701119	0.027750	1.186156
H	-5.451989	-0.433090	-1.807592
H	-7.108973	0.124589	0.710844
H	-7.856065	-0.328947	-2.274938
H	-8.822892	-0.918760	-0.907015
H	-8.692307	0.811360	-1.200781

Homodimericin A – Method 2

conf #	Boltzmann pop. (%)	Relative Energy (kcal/mol)
1	58.81	0.0
2	33.68	0.33
5	3.1	1.74
3	2.92	1.78
6	0.68	2.64
10	0.25	3.22
9	0.24	3.27
8	0.15	3.52
7	0.1	3.79
17	0.01	5.16
21	0.01	5.17
15	0.01	5.33
16	0.01	5.37
20	0.01	5.42
13	0.01	5.53
25	0.0	5.57
18	0.0	5.59
19	0.0	5.75
24	0.0	6.04
32	0.0	6.43
31	0.0	6.45
29	0.0	7.04
23	0.0	7.1
39	0.0	7.39
28	0.0	7.54
36	0.0	7.77
40	0.0	7.87
26	0.0	7.94
27	0.0	8.05
55	0.0	8.6
42	0.0	8.64
33	0.0	8.73
43	0.0	9.0
35	0.0	9.16
59	0.0	9.19
47	0.0	9.32
48	0.0	9.53
51	0.0	9.75
50	0.0	10.71
46	0.0	10.87
49	0.0	10.88
61	0.0	10.91
65	0.0	11.01
63	0.0	11.08
54	0.0	11.24
72	0.0	11.44
53	0.0	11.45

64	0.0	11.57
56	0.0	11.64
67	0.0	11.82
75	0.0	12.07
69	0.0	12.33
78	0.0	13.0
70	0.0	13.1
77	0.0	13.48

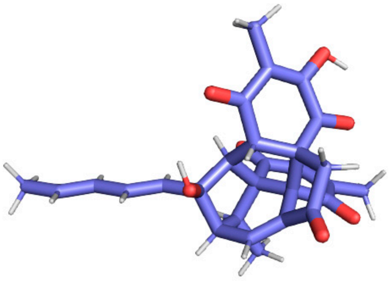

conf 1 (58.81%)

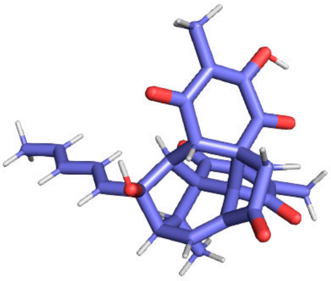

conf 2 (33.68%)

Cartesian Coordinates for Global Minimum of Homodimericin A (Conf #1)

C	2.065065	2.773814	-0.553043
C	0.894110	3.405081	-0.271916
C	-0.137958	2.692621	0.487128
C	0.072617	1.248002	0.879086
C	1.516162	0.687577	0.888116
C	2.441272	1.423006	-0.050072
C	-0.783641	0.332460	-0.054191
C	0.125801	-0.856827	-0.477556
C	1.252250	-0.841445	0.559968
C	-2.033926	-0.319565	0.670877
C	-1.506710	-1.771870	0.987339
C	-0.777852	-2.132360	-0.314278
C	0.626166	-0.783614	-1.891908
C	1.973562	-1.325905	-2.201304
C	2.845382	-1.758362	-1.255607
C	2.522881	-1.599321	0.171167
O	-0.061395	-0.377471	-2.818733
C	-0.082448	-3.491824	-0.333003
O	2.270331	-1.361979	-3.510036
C	4.186915	-2.323464	-1.597767
O	3.267975	-2.020536	1.042364
O	3.541689	0.995153	-0.366782
O	-1.214408	3.214573	0.755073
O	3.007779	3.356792	-1.303611
C	0.598510	4.788892	-0.760773
C	2.157844	0.639473	2.304123
C	1.528586	-0.592269	2.977761

C	0.733102	-1.272395	1.924608
C	-0.525884	-1.674692	2.129774
O	1.602770	-0.860187	4.153445
H	-1.124054	0.882696	-0.929554
O	-2.370978	0.315237	1.895291
C	-3.218108	-0.353311	-0.268438
C	-4.490693	-0.197284	0.122732
C	-5.632974	-0.222218	-0.774168
C	-6.902339	-0.071417	-0.369374
C	-8.102592	-0.095900	-1.263706
H	-0.335348	1.172041	1.885640
H	-2.333553	-2.444004	1.217691
H	-1.498667	-2.114976	-1.134381
H	0.409154	-3.672088	-1.290919
H	-0.825972	-4.279021	-0.186596
H	0.664212	-3.591537	0.456180
H	1.489904	-1.011185	-3.977398
H	4.249227	-2.596875	-2.649714
H	4.961451	-1.580098	-1.381103
H	4.398571	-3.192833	-0.972664
H	3.758326	2.733731	-1.336852
H	1.515381	5.352340	-0.931938
H	0.046820	4.746525	-1.706502
H	-0.034959	5.310853	-0.043076
H	3.236328	0.482651	2.230175
H	1.972379	1.538650	2.892667
H	-0.890700	-1.770141	3.146148
H	-2.580504	1.239960	1.696514
H	-2.997757	-0.509266	-1.321010
H	-4.695984	-0.037181	1.178318
H	-5.426554	-0.375089	-1.832688
H	-7.094312	0.081934	0.691803
H	-7.826199	-0.251008	-2.309290
H	-8.794653	-0.893157	-0.969091
H	-8.663244	0.843139	-1.191909

Sungucine – Method 1

conf #	Boltzmann pop. (%)	Relative Energy (kcal/mol)
3	94.99	0.0
1	3.88	1.89
25	0.56	3.04
12	0.33	3.36
6	0.15	3.8
21	0.05	4.46
5	0.03	4.75
41	0.01	5.49
27	0.0	6.5
45	0.0	6.78
43	0.0	8.17

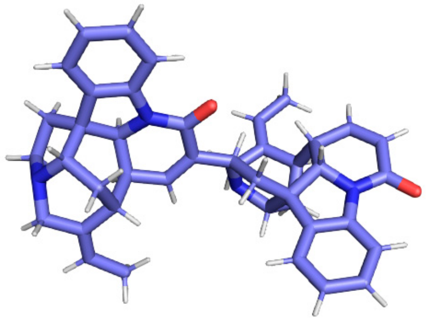

conf 3 (94.99%)

Cartesian Coordinates for Global Minimum of Sungucine (Conf #3)

C	-5.663318	-3.354471	-2.664317
C	-4.335848	-3.658814	-2.974917
C	-3.270963	-3.016420	-2.335347
C	-3.579947	-2.057761	-1.367614
C	-4.907590	-1.738993	-1.046861
C	-5.952621	-2.384608	-1.697419
N	-2.710114	-1.271013	-0.584703
C	-3.480030	-0.602955	0.495280
C	-4.957455	-0.623353	-0.021460
C	-1.337125	-1.263573	-0.640225
C	-0.664256	-0.292727	0.272363
C	-1.367313	0.629272	0.947273
C	-2.868489	0.752757	0.898686
C	-3.325213	1.998300	0.027946
C	-4.169626	1.530331	-1.170856
C	-5.380340	0.746033	-0.645566
N	-6.095106	1.470861	0.435402
C	-6.176006	0.638243	1.639253
C	-5.987044	-0.786962	1.114472
C	-2.199800	4.239001	1.934769

C	-3.645171	3.917998	1.673174
C	-4.149261	2.987879	0.850837
O	-0.693755	-1.971813	-1.418101
C	-5.655828	2.846704	0.647439
H	-3.420058	-1.257631	1.378593
H	-3.205693	0.944898	1.923106
C	4.130923	3.731002	-2.382305
C	5.453517	3.334087	-2.594079
C	5.921925	2.091823	-2.154977
C	5.017758	1.254724	-1.497090
C	3.687691	1.640129	-1.273601
C	3.242315	2.881012	-1.713312
N	5.227871	-0.029221	-0.958930
C	3.919196	-0.642848	-0.611974
C	2.949554	0.578250	-0.479456
C	6.409355	-0.737685	-0.954062
C	6.332857	-2.055423	-0.275431
C	5.270722	-2.447468	0.438885
C	4.018329	-1.617879	0.578169
C	3.916468	-0.955731	2.016733
C	2.726800	0.998789	1.008815
N	1.452090	0.354589	1.421753
C	0.853111	-0.374492	0.295652
C	1.517148	0.260429	-0.941749
C	3.540099	-3.624462	3.646387
C	2.510486	-2.552659	3.418617
C	2.652706	-1.400438	2.749789
O	7.461932	-0.307334	-1.420560
C	1.516894	-0.384501	2.683074
H	3.602333	-1.233600	-1.484427
H	3.167965	-2.301421	0.480947
H	1.111311	-1.446189	0.316533
C	3.903011	0.579373	1.899849
H	-6.473171	-3.868879	-3.173848
H	-4.118769	-4.412308	-3.727412
H	-2.240793	-3.245714	-2.568778
H	-6.985452	-2.140565	-1.460622
H	-0.817087	1.350390	1.548236
H	-2.422932	2.493808	-0.344961
H	-4.497113	2.398190	-1.755141
H	-3.586381	0.899956	-1.849738
H	-6.073400	0.545972	-1.475269
H	-7.142806	0.773944	2.141631
H	-5.394233	0.886449	2.380063
H	-6.930512	-1.158306	0.698916
H	-5.662862	-1.493549	1.885446
H	-2.007188	5.309650	1.784556
H	-1.925652	4.018850	2.976158
H	-1.515820	3.682886	1.287707
H	-4.356959	4.539510	2.220717

H	-5.963457	3.439335	-0.226982
H	-6.203968	3.263534	1.499599
H	3.790138	4.699935	-2.736184
H	6.137722	3.999413	-3.114100
H	6.942756	1.773801	-2.314831
H	2.214190	3.191504	-1.542743
H	7.238720	-2.649240	-0.344586
H	5.299580	-3.392561	0.978425
H	4.799156	-1.259564	2.588834
H	2.613333	2.091344	1.043913
H	0.996980	1.192599	-1.191200
H	1.468693	-0.395284	-1.813667
H	3.607077	-3.877448	4.712700
H	3.269312	-4.553003	3.124196
H	4.539455	-3.333082	3.310525
H	1.536482	-2.752240	3.870155
H	1.634440	0.347186	3.496335
H	0.549692	-0.869311	2.851634
H	3.812357	1.025714	2.897180
H	4.838278	0.956196	1.473957

Sungucine – Method 2

conf #	Boltzmann pop. (%)	Relative Energy (kcal/mol)
3	80.53	0.0
1	14.53	1.01
6	1.45	2.38
16	1.3	2.44
13	0.83	2.71
25	0.64	2.87
12	0.24	3.44
17	0.15	3.71
21	0.15	3.73
5	0.11	3.93
42	0.03	4.59
41	0.01	5.09
28	0.01	5.36
27	0.0	5.8
45	0.0	5.81
35	0.0	6.59
43	0.0	7.24

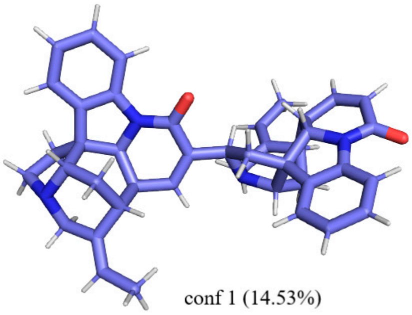
conf 1 (14.53%)

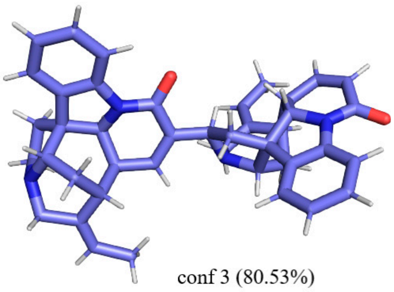
conf 3 (80.53%)

Cartesian Coordinates for Global Minimum of Sungucine (Conf #3)

C	-5.464495	-3.580920	-2.550296
C	-4.130321	-3.777967	-2.910193
C	-3.121500	-3.050633	-2.276796
C	-3.449804	-2.158483	-1.249997
C	-4.786348	-1.912074	-0.932000
C	-5.794599	-2.639638	-1.573533
N	-2.621170	-1.287498	-0.531724
C	-3.431642	-0.587911	0.491772
C	-4.905265	-0.751075	0.034919
C	-1.274166	-1.370105	-0.319873
C	-0.659541	-0.196232	0.301174
C	-1.393881	0.842692	0.766155
C	-2.914502	0.869146	0.757719
C	-3.531530	1.981854	-0.157404
C	-4.407954	1.386225	-1.283026

C	-5.510718	0.520656	-0.622628
N	-6.252587	1.242730	0.435948
C	-6.227398	0.447244	1.656751
C	-5.893763	-0.960766	1.188221
C	-2.495011	4.446556	1.506976
C	-3.942338	4.012937	1.356517
C	-4.410016	2.953586	0.662932
O	-0.610804	-2.339141	-0.701559
C	-5.917806	2.667176	0.569817
H	-3.300915	-1.162474	1.411437
H	-3.194876	1.090224	1.788651
C	4.076411	3.045186	-3.267393
C	5.355289	2.544342	-3.515694
C	5.816185	1.429421	-2.814369
C	5.000674	0.833513	-1.846111
C	3.703446	1.298047	-1.630616
C	3.250721	2.423969	-2.328520
N	5.233798	-0.314720	-1.081522
C	3.951635	-0.760930	-0.461865
C	2.993993	0.460739	-0.584015
C	6.443117	-0.799792	-0.667624
C	6.391367	-1.958288	0.185593
C	5.368934	-2.232045	1.010412
C	4.095817	-1.405180	0.977412
C	3.980496	-0.406537	2.180440
C	2.748674	1.228266	0.743419
N	1.479321	0.722136	1.314544
C	0.876142	-0.228473	0.378070
C	1.556721	0.069204	-0.953420
C	3.662972	-2.622027	4.390172
C	2.600648	-1.623435	3.967143
C	2.707739	-0.674549	3.013170
O	7.529688	-0.296571	-0.953859
C	1.552617	0.294307	2.719727
H	3.592199	-1.543613	-1.132221
H	3.281307	-2.127793	1.052981
H	1.194288	-1.225054	0.693880
C	3.945565	1.064700	1.710328
H	-6.245624	-4.138242	-3.047377
H	-3.877425	-4.487939	-3.684423
H	-2.093040	-3.202365	-2.570322
H	-6.835182	-2.461194	-1.344450
H	-0.869124	1.682583	1.199275
H	-2.733508	2.551304	-0.636050
H	-4.852503	2.173374	-1.892672
H	-3.809891	0.786892	-1.970254
H	-6.238911	0.205966	-1.373534
H	-7.208038	0.481618	2.135163
H	-5.480101	0.805410	2.367964
H	-6.799743	-1.478306	0.870619

H	-5.480064	-1.576451	1.986902
H	-2.371986	5.465962	1.140738
H	-2.206787	4.423852	2.558156
H	-1.800091	3.813326	0.959436
H	-4.654122	4.642931	1.873032
H	-6.283474	3.182565	-0.319867
H	-6.454344	3.099372	1.416632
H	3.721690	3.910391	-3.809122
H	5.989362	3.017251	-4.251877
H	6.805445	1.044162	-3.016284
H	2.260351	2.820414	-2.157812
H	7.280127	-2.569391	0.208071
H	5.436250	-3.089260	1.666497
H	4.846808	-0.512557	2.835690
H	2.603387	2.284840	0.506238
H	1.049918	0.895983	-1.451115
H	1.508547	-0.770337	-1.646988
H	3.881364	-2.505717	5.451986
H	3.305310	-3.638340	4.223711
H	4.597939	-2.505687	3.845898
H	1.667431	-1.708009	4.507896
H	1.714531	1.179058	3.338088
H	0.596455	-0.128306	3.035490
H	3.853169	1.745706	2.556667
H	4.877843	1.338775	1.215102

Strychnobaillonine – Method 1

conf #	Boltzmann pop. (%)	Relative Energy (kcal/mol)
7	29.72	0.0
1	16.97	0.33
14	15.91	0.37
10	12.3	0.52
3	12.12	0.53
2	8.39	0.75
13	2.76	1.41
16	1.82	1.66
8	0.0	5.93
9	0.0	7.08
4	0.0	7.18

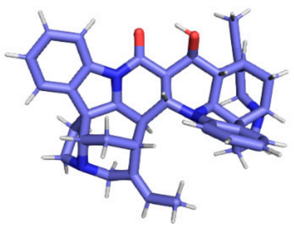
conf 1 (16.97%)

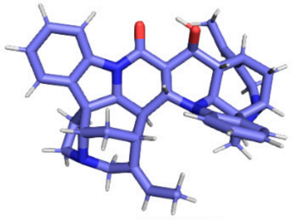
conf 2 (8.39%)

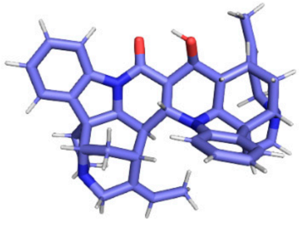
conf 3 (12.12%)

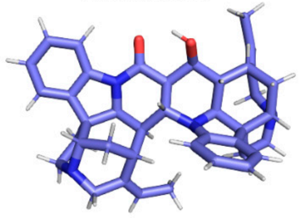
conf 7 (29.72%)

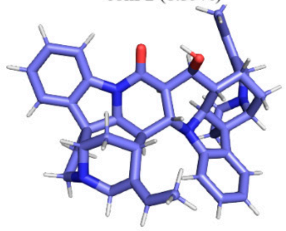
conf 10 (12.30%)

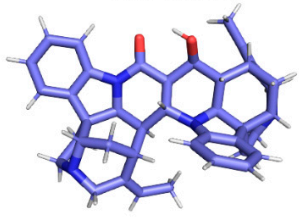
conf 14 (15.91%)

Cartesian Coordinates for Global Minimum of Strychnobaillonine (Conf #7)

C	-6.290935	2.826703	0.488956
C	-5.320039	3.757283	0.865205
C	-3.956568	3.467178	0.758924
C	-3.598094	2.211795	0.263227
C	-4.556994	1.266174	-0.116543
C	-5.909855	1.572692	-0.000452
N	-2.305408	1.669870	0.062374
C	-1.122221	2.318361	0.252955
C	0.110032	1.474299	-0.061604
C	-0.126278	0.019964	0.432587
C	-1.272482	-0.616354	-0.417596
C	-2.407437	0.414844	-0.733737

O	-1.055704	3.480173	0.669000
C	-1.797216	-1.943021	0.202858
C	-2.888658	-1.603854	1.237205
C	-4.160147	-1.191482	0.494639
C	-3.900486	-0.026417	-0.567456
N	-4.745148	-2.333405	-0.237857
C	-5.397481	-1.749984	-1.411859
C	-4.482464	-0.607927	-1.884770
C	-3.773678	-3.401648	-0.529787
C	-2.350833	-2.950387	-0.815128
C	-1.655584	-3.512964	-1.815356
C	-0.200422	-3.345012	-2.155462
C	2.960679	-3.360716	3.034109
C	1.628187	-3.033021	3.276163
C	0.925621	-2.165256	2.428742
C	1.587358	-1.630128	1.320883
C	2.947245	-1.931508	1.089738
C	3.625143	-2.799902	1.932466
N	1.105270	-0.778759	0.310725
C	2.568154	1.160880	0.550089
C	2.298992	-0.112655	-0.270930
C	3.984530	1.762743	0.371350
C	4.977582	0.696131	0.861044
C	4.853968	-0.522281	-0.040519
C	3.433666	-1.174925	-0.132614
N	5.236996	-0.220829	-1.428363
C	4.850136	-1.443582	-2.149597
C	3.594466	-2.039169	-1.422846
C	4.608954	0.988517	-2.011358
C	4.387813	2.142362	-1.041707
C	4.577885	3.401395	-1.466723
C	4.343490	4.686361	-0.720784
C	1.416103	2.166966	0.429984
O	1.290469	2.763967	1.709915
H	-0.803488	-0.854060	-1.375592
H	-2.293507	0.717860	-1.783332
H	0.182903	1.426087	-1.160230
H	-0.416884	0.065367	1.490771
H	2.105438	0.118842	-1.321727
H	2.529054	0.840038	1.599667
H	-7.345013	3.074779	0.576120
H	-5.622892	4.729247	1.245126
H	-3.198685	4.184256	1.039834
H	-6.667423	0.848421	-0.287642
H	-0.949222	-2.419084	0.703677
H	-3.103852	-2.456926	1.890392

H	-2.562114	-0.786375	1.890905
H	-4.907270	-0.821770	1.207215
H	-5.577881	-2.511669	-2.176328
H	-6.378418	-1.355778	-1.110558
H	-4.997958	0.154411	-2.477886
H	-3.673906	-1.022942	-2.497531
H	-4.161509	-4.006181	-1.355449
H	-3.748933	-4.070599	0.344278
H	-2.190699	-4.221514	-2.451022
H	0.347558	-2.758939	-1.414050
H	0.282544	-4.328650	-2.228314
H	-0.070143	-2.865269	-3.136720
H	3.487711	-4.037087	3.700621
H	1.117579	-3.450104	4.140349
H	-0.105562	-1.912953	2.652434
H	4.670913	-3.038290	1.751407
H	4.040400	2.639810	1.021440
H	6.006670	1.070643	0.821401
H	4.759990	0.417946	1.898812
H	5.545845	-1.303278	0.307741
H	4.671350	-1.224103	-3.206719
H	5.680597	-2.158609	-2.103187
H	3.758604	-3.089680	-1.163125
H	2.693025	-2.001094	-2.042831
H	3.635648	0.737038	-2.473142
H	5.244525	1.328782	-2.837993
H	4.945373	3.533604	-2.486520
H	3.873249	4.541307	0.255046
H	5.287268	5.228062	-0.566399
H	3.695299	5.354366	-1.303570
H	1.662488	2.932455	-0.323222
H	0.558435	3.405627	1.620591

Strychnobaillonine – Method 2

conf #	Boltzmann pop. (%)	Relative Energy (kcal/mol)
1	69.49	0.0
6	8.14	1.27
2	7.92	1.29
7	4.61	1.61
16	4.22	1.66
10	3.01	1.86
14	1.37	2.33
3	0.97	2.53
13	0.27	3.29
4	0.0	6.95
8	0.0	7.01
9	0.0	8.31

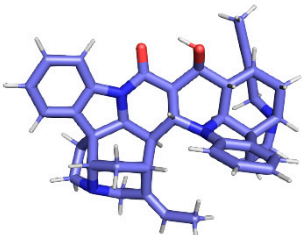
conf 1 (69.49%)

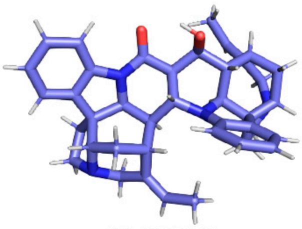
conf 2 (7.92%)

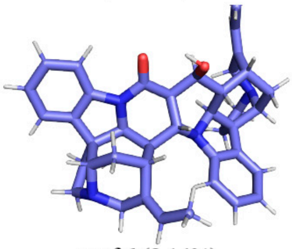
conf 6 (8.14%)

Cartesian Coordinates for Global Minimum of Strychnobaillonine (Conf #7)

C	-6.396128	2.283197	1.007204
C	-5.483289	3.215166	1.502504
C	-4.114963	3.093280	1.252172
C	-3.689090	2.004100	0.491450
C	-4.590191	1.066415	-0.012256
C	-5.948315	1.201416	0.245013
N	-2.378477	1.630978	0.131673
C	-1.236541	2.332072	0.368782
C	0.023779	1.562537	-0.002989
C	-0.174516	0.099526	0.481941
C	-1.254415	-0.574782	-0.405835

C	-2.388585	0.445169	-0.775095
O	-1.226925	3.450050	0.886198
C	-1.709208	-1.932881	0.208636
C	-3.070912	-1.788787	0.897991
C	-4.115553	-1.437382	-0.145979
C	-3.890777	-0.021624	-0.792419
N	-4.200712	-2.401001	-1.256435
C	-5.017717	-1.667457	-2.234859
C	-4.474727	-0.213927	-2.228348
C	-2.894731	-2.758534	-1.858481
C	-1.821508	-3.021509	-0.836440
C	-1.049976	-4.110629	-0.912822
C	0.118607	-4.491321	-0.050609
C	3.087882	-3.035289	3.168055
C	1.760644	-2.723326	3.449090
C	1.002896	-1.918825	2.588777
C	1.606167	-1.435916	1.427986
C	2.960154	-1.722473	1.154425
C	3.693286	-2.524240	2.009400
N	1.072742	-0.658531	0.398012
C	2.505279	1.297667	0.499961
C	2.212691	-0.000304	-0.268072
C	3.886044	1.914648	0.189153
C	4.938083	0.889763	0.638500
C	4.767909	-0.371072	-0.190056
C	3.362227	-1.039205	-0.136522
N	5.023394	-0.131016	-1.620095
C	4.586614	-1.390917	-2.252288
C	3.431947	-1.981396	-1.371569
C	4.328963	1.046338	-2.201330
C	4.142482	2.236280	-1.269227
C	4.202788	3.476961	-1.767152
C	3.945350	4.775616	-1.056168
C	1.323155	2.273985	0.454548
O	1.233927	2.834923	1.761544
H	-0.736152	-0.799039	-1.341361
H	-2.157662	0.827066	-1.772523
H	0.070797	1.532972	-1.099937
H	-0.508195	0.127491	1.524567
H	1.965920	0.189111	-1.311916
H	2.553772	0.998420	1.553060
H	-7.454061	2.399425	1.211460
H	-5.837081	4.054095	2.090895
H	-3.404702	3.814474	1.624063
H	-6.654475	0.476925	-0.145752
H	-0.969364	-2.232986	0.943550

H	-3.338431	-2.724093	1.395135
H	-3.044106	-1.002942	1.660806
H	-5.104927	-1.421009	0.321197
H	-4.969886	-2.140937	-3.217257
H	-6.061032	-1.686991	-1.905482
H	-5.251545	0.519003	-2.448136
H	-3.693305	-0.087869	-2.980701
H	-2.530598	-1.952913	-2.522546
H	-3.047017	-3.634609	-2.492800
H	-1.271312	-4.815798	-1.713477
H	0.302435	-3.797955	0.767995
H	-0.026160	-5.490189	0.375394
H	1.034809	-4.537306	-0.650715
H	3.655842	-3.660412	3.846844
H	1.298539	-3.103527	4.353513
H	-0.024523	-1.681626	2.834779
H	4.732542	-2.748956	1.793331
H	3.980788	2.818564	0.791689
H	5.949201	1.280938	0.497541
H	4.810817	0.654933	1.698918
H	5.498271	-1.121708	0.136146
H	4.287711	-1.215614	-3.287272
H	5.429808	-2.087305	-2.269687
H	3.670831	-2.998832	-1.056031
H	2.473013	-2.021022	-1.892023
H	3.333057	0.758535	-2.574450
H	4.894324	1.363850	-3.081593
H	4.456889	3.581929	-2.821646
H	3.610963	4.645964	-0.026533
H	4.846257	5.399882	-1.044821
H	3.175780	5.350712	-1.582919
H	1.519458	3.068375	-0.278921
H	0.463229	3.424130	1.741150

Paclitaxel – Method 1

conf #	Boltzmann pop. (%)	Relative Energy (kcal/mol)
6	27.03	0.0
2	18.92	0.21
23	15.8	0.32
4	15.79	0.32
21	6.88	0.81
9	4.82	1.02
5	2.67	1.37
1	2.66	1.37
12	1.57	1.68
20	1.37	1.77
3	1.25	1.82
27	0.29	2.68
17	0.25	2.77
11	0.2	2.91
25	0.14	3.12
37	0.14	3.12
51	0.12	3.2
15	0.05	3.69
13	0.01	4.47
10	0.01	4.69
16	0.01	4.78
45	0.0	5.1
52	0.0	5.45
8	0.0	5.51
36	0.0	5.67
22	0.0	6.02
53	0.0	6.04
26	0.0	6.56
35	0.0	6.58
14	0.0	6.76
46	0.0	6.96
43	0.0	7.22
29	0.0	7.32
44	0.0	7.44
50	0.0	7.57
40	0.0	8.3
18	0.0	8.31
49	0.0	8.45
42	0.0	8.73
7	0.0	8.77
28	0.0	8.86
31	0.0	8.93
41	0.0	9.48
39	0.0	9.79
30	0.0	9.97
34	0.0	10.38
33	0.0	10.47

47	0.0	10.94
32	0.0	10.98
38	0.0	11.8
48	0.0	13.23

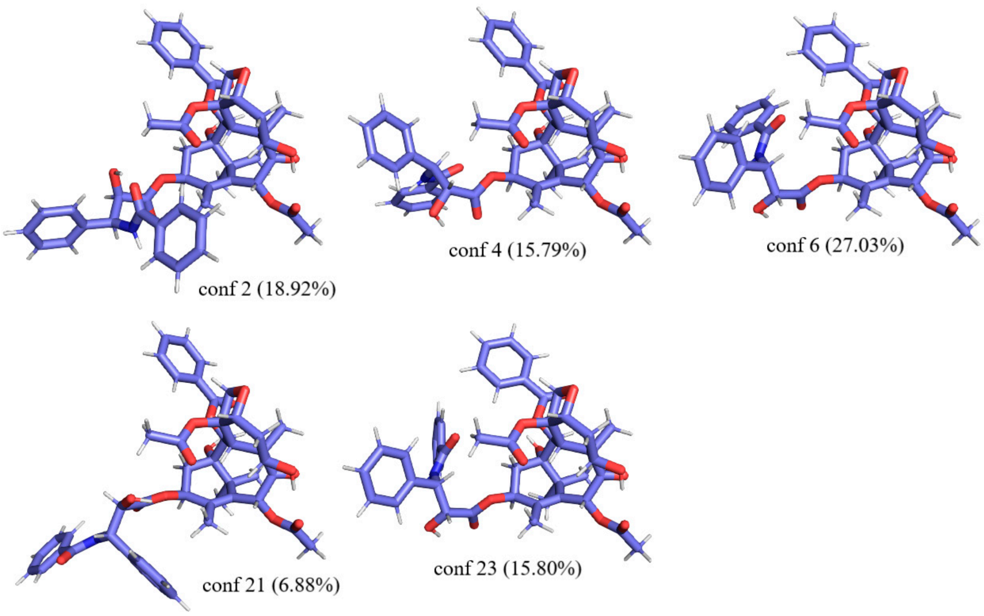

Cartesian Coordinates for Global Minimum of Taxol (Conf #6)

C	1.550727	-1.985784	-1.273545
C	0.196110	-1.441203	-1.687162
C	0.082220	0.077391	-1.462204
C	1.386676	0.885326	-1.713541
C	2.459695	0.011997	-2.466841
C	2.639108	-1.249286	-1.600132
C	1.550168	-3.285674	-0.506316
O	-0.869232	-2.076382	-0.901342
C	-1.830043	-2.711530	-1.578899
C	-2.914432	-3.320164	-0.683080
O	-1.872351	-2.852552	-2.787630
C	-3.560053	-2.273475	0.243584
O	-3.897919	-3.898540	-1.519761
C	-4.473047	-2.859361	1.322447
N	-4.253819	-1.291651	-0.586183
C	-4.091324	0.055571	-0.452350
C	-4.909636	0.912790	-1.374397
O	-3.308812	0.551615	0.366070
C	-6.128957	0.493489	-1.926512
C	-6.846811	1.339722	-2.771102
C	-6.351411	2.608987	-3.075378

C	-5.142662	3.036013	-2.520186
C	-4.429662	2.196498	-1.667393
C	-4.867231	-2.015848	2.373632
C	-5.694107	-2.483984	3.392618
C	-6.142115	-3.807293	3.378965
C	-5.761125	-4.650367	2.336359
C	-4.934350	-4.180998	1.311600
C	3.964771	-1.555809	-0.955453
C	4.645829	-0.321851	-0.300465
O	5.767959	-0.025885	-0.653082
C	3.987842	0.378233	0.908461
C	2.420096	0.594630	0.810510
C	1.941849	1.524063	-0.371125
C	1.840125	1.052356	2.182209
C	2.619218	0.724476	3.494942
C	3.906586	-0.092827	3.453577
C	4.232194	-0.676720	2.079081
C	1.992135	2.516387	2.637736
O	2.845251	2.140819	3.737927
O	0.440937	0.684063	2.287128
C	0.130737	-0.600553	2.580446
O	0.942205	-1.506832	2.591116
C	-1.329634	-0.725860	2.923903
O	0.855931	2.356007	0.114174
C	1.979586	-0.374583	-3.897613
C	3.732032	0.848835	-2.752974
C	4.795303	1.662229	1.180433
O	5.572809	-1.116743	2.151745
O	4.878431	-2.133392	-1.910803
C	5.938579	-2.782438	-1.376148
O	6.078497	-2.979224	-0.183534
C	6.903794	-3.225611	-2.443025
C	0.831808	3.660920	-0.249901
O	1.724659	4.199486	-0.884840
C	-0.389200	4.356835	0.232498
C	-1.474728	3.661516	0.787435
C	-2.607159	4.364447	1.197183
C	-2.657562	5.754466	1.066054
C	-1.575885	6.447473	0.514431
C	-0.446812	5.751196	0.092070
O	0.984573	1.976967	-2.546788
H	1.986007	-0.390644	0.659440
H	-0.027647	-1.700970	-2.720149
H	-0.273858	0.240529	-0.442432
H	-0.684080	0.484516	-2.126419
H	1.172591	-3.150762	0.514652
H	0.888578	-4.007654	-0.999664
H	2.539350	-3.743959	-0.453806
H	-2.431048	-4.088225	-0.059119
H	-2.754440	-1.726849	0.734553

H	-3.516116	-3.885987	-2.420060
H	-4.856280	-1.668789	-1.305080
H	-6.545451	-0.477868	-1.673075
H	-7.795498	1.009800	-3.185291
H	-6.909432	3.265749	-3.737323
H	-4.755894	4.024957	-2.749832
H	-3.496376	2.519803	-1.219205
H	-4.524986	-0.984271	2.383821
H	-5.985746	-1.816799	4.199543
H	-6.784482	-4.175668	4.174348
H	-6.110249	-5.679356	2.312212
H	-4.669067	-4.844808	0.495734
H	3.825254	-2.283109	-0.154433
H	2.751262	2.205132	-0.634016
H	1.932920	0.332931	4.252988
H	3.860991	-0.923892	4.163932
H	4.734876	0.550925	3.765168
H	3.552754	-1.521318	1.897993
H	1.043571	2.953337	2.967892
H	2.500005	3.203248	1.955162
H	-1.598236	-1.779688	3.008350
H	-1.511473	-0.232873	3.886328
H	-1.949260	-0.223579	2.174821
H	1.625505	0.517789	-4.419449
H	2.830376	-0.787127	-4.449828
H	1.189881	-1.123200	-3.936571
H	4.128264	1.409863	-1.908494
H	4.538674	0.212908	-3.124998
H	3.492546	1.576168	-3.534997
H	4.459834	2.164387	2.087311
H	5.846698	1.399733	1.304103
H	4.733582	2.366924	0.344311
H	5.755049	-1.758985	1.440177
H	7.586471	-3.970579	-2.032677
H	6.372661	-3.624221	-3.311440
H	7.479579	-2.353555	-2.773481
H	-1.441675	2.582833	0.884760
H	-3.450117	3.819361	1.611604
H	-3.541013	6.298332	1.390329
H	-1.615631	7.528272	0.411239
H	0.399846	6.267471	-0.348324
H	1.618695	2.708307	-2.446959

Paclitaxel – Method 2

conf #	Boltzmann pop. (%)	Relative Energy (kcal/mol)
6	43.08	0.0
23	18.2	0.51
21	12.75	0.72
3	11.41	0.79
4	3.46	1.49
2	3.15	1.55
9	2.26	1.75
5	1.43	2.02
20	1.33	2.06
19	1.21	2.11
27	0.84	2.33
11	0.43	2.73
1	0.11	3.54
35	0.1	3.59
12	0.09	3.69
15	0.08	3.76
16	0.02	4.49
37	0.02	4.53
13	0.01	5.04
10	0.01	5.15
52	0.0	5.42
36	0.0	5.71
22	0.0	6.07
43	0.0	6.26
46	0.0	6.34
8	0.0	6.4
17	0.0	6.62
29	0.0	6.71
14	0.0	7.45
51	0.0	7.84
45	0.0	7.85
41	0.0	7.86
42	0.0	8.03
53	0.0	8.32
26	0.0	8.38
44	0.0	8.46
54	0.0	8.48
32	0.0	8.8
30	0.0	9.25
39	0.0	9.39
40	0.0	9.43
7	0.0	9.54
49	0.0	9.75
34	0.0	9.94
18	0.0	10.21
50	0.0	10.32
28	0.0	10.4

38	0.0	10.52
47	0.0	10.78
33	0.0	10.79
31	0.0	13.53

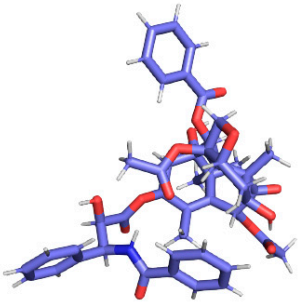
conf 3 (11.41%)

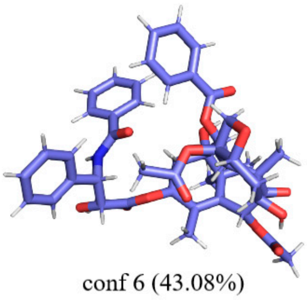
conf 6 (43.08%)

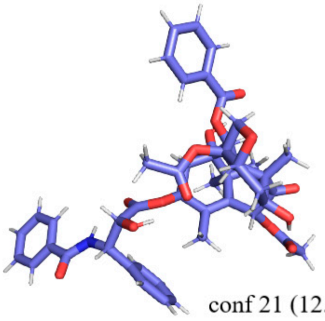
conf 21 (12.75%)

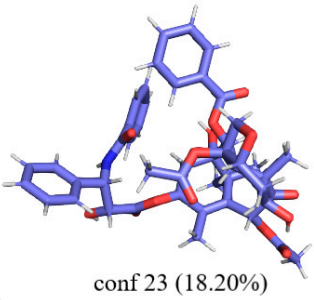
conf 23 (18.20%)

Cartesian Coordinates for Global Minimum of Taxol (Conf #6)

C	1.616817	-2.047600	-1.136973
C	0.234092	-1.634799	-1.583033
C	-0.022354	-0.133378	-1.385549
C	1.200728	0.779340	-1.644417
C	2.356334	-0.005754	-2.364147
C	2.633637	-1.228333	-1.473743
C	1.711665	-3.308339	-0.322979
O	-0.796367	-2.341221	-0.819033
C	-1.722063	-3.003498	-1.515161
C	-2.884422	-3.495525	-0.650611
O	-1.696274	-3.207876	-2.708007
C	-3.459342	-2.333283	0.169059
O	-3.883377	-4.034122	-1.497010
C	-4.552331	-2.705951	1.162544
N	-3.886069	-1.287471	-0.759572
C	-3.509207	0.010502	-0.614971
C	-3.929652	0.957319	-1.695829
O	-2.830885	0.392271	0.339390
C	-5.127634	0.814798	-2.404798

C	-5.473899	1.744266	-3.382774
C	-4.622635	2.812170	-3.665551
C	-3.427057	2.956385	-2.960842
C	-3.086186	2.038917	-1.973399
C	-5.000573	-1.702755	2.033495
C	-5.978404	-1.971917	2.985064
C	-6.526410	-3.252098	3.082003
C	-6.089797	-4.252435	2.218359
C	-5.109180	-3.982570	1.261515
C	3.969612	-1.401385	-0.813672
C	4.520479	-0.108682	-0.166418
O	5.612849	0.290236	-0.490470
C	3.768766	0.524159	1.020742
C	2.199799	0.596013	0.877262
C	1.680600	1.479697	-0.311385
C	1.540924	0.992509	2.225243
C	2.303961	0.732814	3.559088
C	3.665109	0.049700	3.552378
C	4.067579	-0.507830	2.192473
C	1.536448	2.461852	2.675639
O	2.373530	2.165773	3.818354
O	0.184910	0.482642	2.290740
C	0.014104	-0.828133	2.571156
O	0.922092	-1.631294	2.570992
C	-1.412558	-1.115676	2.928146
O	0.527685	2.223604	0.149123
C	1.935695	-0.453884	-3.792241
C	3.560640	0.927485	-2.632302
C	4.441143	1.875182	1.315615
O	5.441874	-0.842324	2.292526
O	4.943166	-1.911051	-1.748097
C	6.046417	-2.462420	-1.197561
O	6.192683	-2.610395	-0.002543
C	7.043807	-2.853426	-2.247168
C	0.419065	3.530602	-0.175194
O	1.267928	4.142386	-0.792031
C	-0.850051	4.114959	0.323597
C	-1.784142	3.350274	1.033668
C	-2.974478	3.936018	1.452146
C	-3.232469	5.278041	1.174780
C	-2.299296	6.041720	0.470241
C	-1.110598	5.462072	0.043568
O	0.711926	1.820022	-2.499389
H	1.860934	-0.419215	0.711683
H	0.075243	-1.926409	-2.616652
H	-0.401405	0.016057	-0.375175
H	-0.816958	0.180993	-2.060993
H	1.310024	-3.152349	0.682343
H	1.109888	-4.092067	-0.790710
H	2.730858	-3.682562	-0.241800

H	-2.493630	-4.258556	0.035607
H	-2.629475	-1.907488	0.727340
H	-3.473811	-4.147568	-2.367943
H	-4.418124	-1.585714	-1.564080
H	-5.809044	0.003034	-2.175799
H	-6.409765	1.637869	-3.918909
H	-4.892916	3.531898	-4.429802
H	-2.761161	3.783898	-3.175673
H	-2.166915	2.146369	-1.415509
H	-4.574142	-0.708681	1.959353
H	-6.310687	-1.185259	3.653170
H	-7.288104	-3.464959	3.823464
H	-6.514344	-5.248142	2.281276
H	-4.801330	-4.766281	0.582307
H	3.883258	-2.119805	0.000329
H	2.441197	2.212504	-0.570221
H	1.639687	0.267703	4.290038
H	3.688932	-0.772470	4.269358
H	4.416899	0.776366	3.867196
H	3.465632	-1.402170	1.996656
H	0.542518	2.813624	2.958660
H	2.014131	3.180931	2.009728
H	-1.599565	-2.186267	2.872915
H	-1.577499	-0.779776	3.956121
H	-2.083727	-0.557926	2.276926
H	1.581262	0.408699	-4.356581
H	2.812173	-0.866903	-4.296108
H	1.160567	-1.213133	-3.827141
H	3.885500	1.525929	-1.786895
H	4.421901	0.351555	-2.970361
H	3.289971	1.623317	-3.428498
H	4.024433	2.351871	2.197810
H	5.502397	1.710617	1.491278
H	4.350787	2.560872	0.471112
H	5.686805	-1.463871	1.589702
H	7.780581	-3.529856	-1.819508
H	6.545378	-3.309635	-3.102757
H	7.544525	-1.945308	-2.593797
H	-1.597507	2.303091	1.221011
H	-3.704449	3.339005	1.985798
H	-4.162722	5.729275	1.501195
H	-2.501993	7.083789	0.252613
H	-0.376362	6.034237	-0.509642
H	1.332594	2.559186	-2.492180

VI. Density Functionals and Basis Sets Employed

The following density functionals and basis sets that are implemented in Gaussian 16, Revision C.01 [1], were used in this benchmark study.

A. Exchange Functionals

a. Local Density Approximation (LDA)

- **Xα**: XAlpha exchange, (0.7)*$\rho^{4/3}$ [39-41]
- **S**: Slater exchange, (2/3)*$\rho^{4/3}$ [39-41]

b. Generalized Gradient Approximation (GGA)

- **B**: Becke's gradient functional (includes Slater exchange) [42]
- **PW91**: Perdew and Wang's gradient functional [43-46]
- **mPW**: modified PW91 gradient functional [47]
- **PBE**: Perdew, Burke, and Ernzerhof's gradient functional [48, 49]
- **G96**: Gill's gradient functional [50, 51]

c. Meta-GGA

- **TPSS**: Tao, Perdew, Staroverov, and Scuseria's meta-GGA exchange functional [52]
- **revTPSS**: revised version of TPSS [53, 54]
- **PKZB**: Perdew, Kurth, Zupan, and Blaha's meta-GGA exchange functional [55]
- **BRx**: Exchange component of Becke and Roussel's 1989 meta-GGA functional [56]

B. Correlation Functionals

a. LDA

- **VWN**: Vosko, Wilk, and Nusair correlation functional [57]
- **PL**: Perdew's 1981 local functional [58]

b. GGA

- **LYP**: Lee, Yang, and Parr's gradient correlation functional [59, 60]
- **V5LYP**: Lee-Yang-Parr non-local (gradient correction) with VWN5 local functional [57, 59, 60]
- **P86**: Perdew's 1986 gradient correlation functional [61]
- **VP86**: VWN5 local and P86 non-local correlation functional [57, 61]
- **PW91**: Perdew and Wang's 1991 gradient-corrected correlation functional [43-46]
- **PBE**: the correlation component of Perdew, Burke, and Ernzerhof [48, 49]

c. Meta-GGA

- **TPSS**: τ-dependent correlation part of TPSS [52]
- **revTPSS**: revised TPSS correlation functional by Perdew *et. al.* [53, 54]
- **PKZB**: Perdew, Kurth, Zupan, and Blaha's meta-GGA [55]
- **BRc**: Becke and Roussel's meta-GGA correlation functional [56]

C. <u>Exchange-Correlation Combinations</u>

a. GGA

- **SOGGA11**: 2011 second-order GGA functional [62]
- **HCTH**, **HCTH/93**, **HCTH/14**: Hamprecht, Cohen, Tozer, and Handy's 15-parameter semi-empirical GGA functionals [63-65]
- **N12**: Minnesota-12 functional with nonseparable gradient approximation (NGA) [66]

b. Hybrid Density Functionals

- **B3LYP**: Becke's 3-parameter hybrid functional with LYP correlation [67]
- **B3PW91**: Becke's 3-parameter hybrid functional with PW91 correlation [67]
- **B1LYP**: Becke's 1-paramter functional using B exchange and LYP correlation [68]
- **O3LYP**: Cohen and Handy's 3-parameter hybrid functional using OPTX exchange [69]
- **X3LYP**: Xu and Goddard's 5-parameter (eXtended) hybrid functional [70]
- **mPW1PW91**: one-parameter hybrid with mPW exchange and PW91 correlation [47]
- **mPW1PBE**: one-parameter hybrid with mPW exchange and PBE correlation [47]
- **mPW1LYP**: one-parameter hybrid with mPW exchange and LYP correlation [47]
- **mPW3PBE**: hybrid functional with three-parameter variant of mPW and PBE correlation [47]
- **PBE0**: Adamo and Barone's hybrid using PBE exchange and correlation [71]
- **PBEh1PBE**: revision of PBE0 using PBEh exchange [72]
- **APF**: Austin-Frisch-Petersson functional [73]
- **APFD**: APF functional with dispersion [73]
- **WC04, WP04**: Empirically-optimized functionals for ^{13}C and ^{1}H NMR predictions, respectively [74]
- **SOGGA11X**: The hybrid variant of SOGGA11 [75]
- **B97-1**: Modified B97 functional by Hamprecht, *et. al.* [63]
- **B97-2**: Modified B97 functional by Wilson, *et. al.* [76]
- **B97-D**: Grimme's B97 functional with empirical dispersion [77]
- **B98**: Becke's 1998 modification of the B97 XC functional [78, 79]

c. Meta-GGA

- **VSXC**: τ-dependent GGA functional from van Voorhis and Scuseria [80]
- **τHCTH**: τ-dependent version of HCTH [81]
- **M06-L**: non-hybrid (local) version of M06 [82]
- **M11-L**: non-hybrid (local) version of M11 [83]
- **MN12-L**: τ-dependent (local) Minnesota-12 functional [84]
- **MN15-L**: non-hybrid (local) version of MN15 [85]

d. Range-Separated Hybrid

- **CAM-B3LYP**: long-range corrected version of B3LYP [86]
- **LC-ωPBE**: long-range corrected version of ωPBE [87-89]
- **LC-ωHPBE**: revised version of LC-ωPBE [90]
- **ωB97**, **ωB97X**, **ωB97X-D**: Head-Gordon and co-workers' long-range hybrid functionals [91, 92]
- **HISS**: range-separated hybrid functional from Henderson, Izmaylov, Scuseria, and Savin [93]
- **HSE06**: Heyd-Scuseria-Ernzerhof range-separated hybrid functional [90, 94-99]
- **N12-SX**: screened exchange hybrid version of N12 [100]

e. Hybrid Meta-GGA

- **B1B95**: meta-GGA hybrid using Becke's 1-parameter functional with B95 correlation [101]
- **TPSSh**: hybrid version of TPSS [52, 102]
- **τHCTHhyb**: hybrid version of τHCTH [81]
- **PW6B95**: Zhao and Truhlar's hybrid meta-GGA functional [103]
- **M05**: Minnesota-05 functional [104]
- **M05-2X**: twice the exchange as M05 [105]
- **M06**: Minnesota-06 functional [106]
- **M06-2X**: twice the exchange as M06 [106]
- **M06-HF**: M06 with 100% HF exchange [107, 108]
- **M08-HX**: Minnesota-08 functional with high exchange [109]
- **MN15**: Minnesota-15 hybrid meta-GGA functional [110]

f. Range-Separated Hybrid Meta-GGA

- **M11**: Minnesota-11 functional [111]
- **MN12-SX**: screened exchange hybrid version of MN12-L [100]

D. Basis Sets

In addition to testing density functionals, a set of 40 basis sets were evaluated. These were as follows:

- **Pople-type basis sets**: [1, 112-119] double-zeta (3-21G, 4-31G, 6-21G, and 6-31G) and triple-zeta (6-311G) with addition of diffuse [120] (+ or ++) and polarization [121] (d, p, and f) functions
- **Dunning's correlation consistent basis sets**: [122, 123] cc-pV*n*Z (where *n* = D or T, for double- and triple-zeta, respectively) and those with additional diffuse functions, aug-cc-pV*n*Z
- **Truhlar's calendar basis sets**: [124] apr-, may-, jun-, and jul-cc-pV*n*Z, which sequentially (starting from jul-cc-pV*n*Z) remove diffuse functions from aug-cc-pV*n*Z
- **Ahlrich's split valence basis sets**: [125, 126] SV, SVP, TZ, and TZVP
- **2nd revised version of Ahlrich's basis sets**: [127, 128] denoted with the prefix "def2" and spanning from double to quadruple zeta with varying sets of polarization functions
- **EPR-II, EPR-III**: [129] Double- and triple-zeta basis sets, respectively, optimized for hyperfine coupling constant calculations
- **MIDI!**: [130] Truhlar's economical basis set, designed for geometry optimizations
- **D95**, **D95V**: [131] Dunning and Huzinaga full and valence double-zeta basis sets, respectively

VII. References

1. Frisch, M. J.; Trucks, G. W.; Schlegel, H. B.; Scuseria, G. E.; Robb, M. A.; Cheeseman, J. R.; Scalmani, G.; Barone, V.; Petersson, G. A.; Nakatsuji, H.; Li, X.; Caricato, M.; Marenich, A. V.; Bloino, J.; Janesko, B. G.; Gomperts, R.; Mennucci, B.; Hratchian, H. P.; Ortiz, J. V.; Izmaylov, A. F.; Sonnenberg, J. L.; Williams; Ding, F.; Lipparini, F.; Egidi, F.; Goings, J.; Peng, B.; Petrone, A.; Henderson, T.; Ranasinghe, D.; Zakrzewski, V. G.; Gao, J.; Rega, N.; Zheng, G.; Liang, W.; Hada, M.; Ehara, M.; Toyota, K.; Fukuda, R.; Hasegawa, J.; Ishida, M.; Nakajima, T.; Honda, Y.; Kitao, O.; Nakai, H.; Vreven, T.; Throssell, K.; Montgomery Jr., J. A.; Peralta, J. E.; Ogliaro, F.; Bearpark, M. J.; Heyd, J. J.; Brothers, E. N.; Kudin, K. N.; Staroverov, V. N.; Keith, T. A.; Kobayashi, R.; Normand, J.; Raghavachari, K.; Rendell, A. P.; Burant, J. C.; Iyengar, S. S.; Tomasi, J.; Cossi, M.; Millam, J. M.; Klene, M.; Adamo, C.; Cammi, R.; Ochterski, J. W.; Martin, R. L.; Morokuma, K.; Farkas, O.; Foresman, J. B.; Fox, D. J. *Gaussian 16 Rev. C.01*, Wallingford, CT, 2016.
2. Lu, C.; Wu, C.; Ghoreishi, D.; Chen, W.; Wang, L.; Damm, W.; Ross, G. A.; Dahlgren, M. K.; Russell, E.; Von Bargen, C. D. OPLS4: Improving Force Field Accuracy on Challenging Regimes of Chemical Space. *J. Chem. Theory Comput.* **2021**, *17*, 4291-4300.
3. MacroModel, Schrödinger Release 2021-1: MacroModel, Schrödinger, LLC, New York, NY, 2021.
4. Lodewyk, M. W.; Soldi, C.; Jones, P. B.; Olmstead, M. M.; Rita, J.; Shaw, J. T.; Tantillo, D. J. The Correct Structure of Aquatolide - Experimental Validation of a Theoretically-Predicted Structural Revision. *J. Am. Chem. Soc.* **2012**, *134*, 18550-18553.
5. Kelly, D. P.; Giansiracusa, J. J.; Leslie, D. R.; McKern, I. D.; Sinclair, G. C. ^{13}C-^{1}H Coupling Constants in Carbocations. 5.[1] Trishomocyclopropenium Cations Generated from Bicyclo[3.1.0]hex-3-yl, Tricyclo[3.2.1.0^{2,4}]oct-8-yl, and Pentacyclo[4.3.0.0^{2,4}.0^{3,8}.0^{5,7}]non-9-yl Precursors. *J. Org. Chem.* **1988**, *53*, 2497-2504.
6. Guan, Y.; Sowndarya, S. S.; Gallegos, L. C.; St. John, P. C.; Paton, R. S. Real-Time Prediction of ^{1}H and ^{13}C Chemical Shifts with DFT Accuracy Using a 3D Graph Neural Network. *Chem. Sci.* **2021**, *12*, 12012-12026.
7. Deng, Q.; Jiang, L.; Yu, Y.; Yang, Y. Theoretical Exploration of the Mechanism of α-Pinene Hydrogenation. *J. Organomet. Chem.* **2022**, *980-981*, 122513.
8. Lee, S. G. α-Pinene and Myrtenol: Complete ^{1}H NMR Assignment. *Magn. Reson. Chem.* **2002**, *40*, 311-312.
9. Akssira, M.; Mellouki, F.; Salhi, A.; Alilou, H.; Saouf, A.; El Hanbali, F.; Arteaga, J. F.; Barrero, A. F. Naupliolide, a Sesquiterpene Lactone with a Novel Tetracyclic Skeleton from *Nauplius graveolens* Subsp. *odorus*. *Tet. Lett.* **2006**, *47*, 6719-6721.
10. Dong, M.; Cong, B.; Yu, S.-H.; Sauriol, F.; Huo, C.-H.; Shi, Q.-W.; Gu, Y.-C.; Zamir, L. O.; Kiyota, H. Echinopines A and B: Sesquiterpenoids Possessing an Unprecedented Skeleton from *Echinops spinosus*. *Org. Lett.* **2008**, *10*, 701-704.
11. Tiuman, T. S.; Ueda-Nakamura, T.; Garcia Cortez, D. g. A. c.; Dias Filho, B. P.; Morgado-Díaz, J. A.; de Souza, W.; Nakamura, C. V. Antileishmanial Activity of Parthenolide, a Sesquiterpene Lactone Isolated from *Tanacetum parthenium*. *Antimicrob. Agents Chemother.* **2005**, *49*, 176-182.

12. Kutateladze, A. G.; Kuznetsov, D. M.; Beloglazkina, A. A.; Holt, T. Addressing the Challenges of Structure Elucidation in Natural Products Possessing the Oxirane Moiety. *J. Org. Chem.* **2018**, *83*, 8341-8352.
13. Kutateladze, A. G.; Reddy, D. S. High-Throughput *in silico* Structure Validation and Revision of Halogenated Natural Products is Enabled by Parametric Corrections to DFT-Computed ^{13}C NMR Chemical Shifts and Spin–Spin Coupling Constants. *J. Org. Chem.* **2017**, *82*, 3368-3381.
14. Valdez-Calderón, A.; Torres-Valencia, J. M.; Manríquez-Torres, J. J.; Velázquez-Jiménez, R.; Román-Marín, L. U.; Hernández-Hernández, J. D.; Cerda-García-Rojas, C. M.; Joseph-Nathan, P. An Unusual Diepoxyguaianolide from Stevia Tomentosa. *Tet. Lett.* **2013**, *54*, 3286-3289.
15. Wood, J. S.; Gordon, W. H.; Morgan, J. B.; Williamson, R. T. Calculated and Experimental ^{1}H and ^{13}C NMR Assignments for Cannabicitran. *Magn. Reson. Chem.* **2022**, *60*, 196-202.
16. Lodewyk, M. W.; Siebert, M. R.; Tantillo, D. J. Computational Prediction of ^{1}H and ^{13}C Chemical Shifts: A Useful Tool for Natural Product, Mechanistic, and Synthetic Organic Chemistry. *Chem. Rev.* **2012**, *112*, 1839-1862.
17. Rigby, J. H.; de Sainte Claire, V.; Cuisiat, S. V.; Heeg, M. J. Synthetic Studies on the Ingenane Diterpenes. Direct Conversion of the *out*,*out*-Bicyclo[4.4.1]undecane System into a Highly Strained In,out Stereoisomer. *J. Org. Chem.* **1996**, *61*, 7992-7993.
18. Galasso, V.; Kovač, B.; Modelli, A. A Theoretical and Experimental Study on the Molecular and Electronic Structures of Artemisinin and Related Drug Molecules. *Chem. Phys.* **2007**, *335*, 141-154.
19. Zhongshan, W.; Nakashima, T. T.; Kopecky, K. R.; Molina, J. Qinghaosu: ^{1}H and ^{13}C Nuclear Magnetic Resonance Spectral Assignments and Luminescence. *Can. J. Chem.* **1985**, *63*, 3070-3074.
20. Lodewyk, M. W.; Tantillo, D. J. Prediction of the Structure of Nobilisitine A Using Computed NMR Chemical Shifts. *J. Nat. Prod.* **2011**, *74*, 1339-1343.
21. Evidente, A.; Abou-Donia, A. H.; Darwish, F. A.; Amer, M. E.; Kassem, F. F.; Hammoda, H. A.; Motta, A. Nobilisitine A and B, Two Masanane-Type Alkaloids from *Clivia nobilis*. *Phytochemistry* **1999**, *51*, 1151-1155.
22. Marrero, J.; Rodríguez, A. D.; Barnes, C. L. Intricarene, an Unprecedented Trispiropentacyclic Diterpene from the Caribbean Sea Plume *Pseudopterogorgia kallos*. *Org. Lett.* **2005**, *7*, 1877-1880.
23. Bagno, A.; Rastrelli, F.; Saielli, G. Toward the Complete Prediction of the ^{1}H and ^{13}C NMR Spectra of Complex Organic Molecules by DFT Methods: Application to Natural Substances. *Chem. - A Eur. J.* **2006**, *12*, 5514-5525.
24. Semenov, V. A.; Krivdin, L. B. DFT Computational Schemes for ^{1}H and ^{13}C NMR Chemical Shifts of Natural Products, Exemplified by Strychnine. *Magn. Reson. Chem.* **2020**, *58*, 56-64.
25. Martin, G. E.; Hadden, C. E.; Crouch, R. C.; Krishnamurthy, V. ACCORD-HMBC: Advantages and Disadvantages of Static Versus Accordion Excitation. *Magn. Reson. Chem.* **1999**, *37*, 517-528.
26. Semenov, V. A.; Samultsev, D. O.; Krivdin, L. B. ^{1}H and ^{13}C NMR Spectra of *Strychnos* Alkaloids: Selected NMR Updates. *Int. J. Quantum Chem.* **2020**, *120*, e26348.

27. Cherif, A.; Martin, G. E.; Soltero, L. R.; Massiot, G. Configuration and Total Assignment of the ^{1}H- and ^{13}C-NMR Spectra of the Alkaloid Holstiine. *J. Nat. Prod.* **1990**, *53*, 793-802.
28. Pierens, G. K.; Venkatachalam, T.; Reutens, D. C. NMR and DFT Investigations of Structure of Colchicine in Various Solvents Including Density Functional Theory Calculations. *Sci. Rep.* **2017**, *7*, 1-9.
29. Saielli, G.; Bagno, A. Can Two Molecules have the Same NMR Spectrum? Hexacyclinol Revisited. *Org. Lett.* **2009**, *11*, 1409-1412.
30. Rychnovsky, S. D. Predicting NMR Spectra by Computational Methods: Structure Revision of Hexacyclinol. *Org. Lett.* **2006**, *8*, 2895-2898.
31. Williams, A.; Elyashberg, M.; Blinov, K.; Lankin, D.; Martin, G.; Reynolds, W.; Porco Jr, J.; Singleton, C.; Su, S. Applying Computer-Assisted Structure Elucidation Algorithms for the Purpose of Structure Validation: Revisiting the NMR Assignments of Hexacyclinol. *J. Nat. Prod.* **2008**, *71*, 581-588.
32. Semenov, V. A.; Krivdin, L. B. Computational ^{1}H and ^{13}C NMR of Strychnobaillonine: On the Way to Larger Molecules Calculated at Lower Computational Costs. *Magn. Reson. Chem.* **2021**, *59*, 108-116.
33. Tchinda, A. T.; Jansen, O.; Nyemb, J.-N.; Tits, M.; Dive, G.; Angenot, L.; Frederich, M. Strychnobaillonine, an Unsymmetrical Bisindole Alkaloid with an Unprecedented Skeleton from *Strychnos icaja* Roots. *J. Nat. Prod.* **2014**, *77*, 1078-1082.
34. Semenov, V. A.; Krivdin, L. B. Simple and Versatile Scheme for the Stereochemical Identification of Natural Products and Diverse Organic Compounds with Multiple Asymmetric Centers. *J. Phys. Chem. A* **2021**, *125*, 10359-10372.
35. Frederich, M.; De Pauw, M.-C.; Llabres, G.; Tits, M.; Hayette, M.-P.; Brandt, V.; Penelle, J.; De Mol, P.; Angenot, L. New Antimalarial and Cytotoxic Sungucine Derivatives from *Strychnos icaja* Roots. *Planta Med.* **2000**, *66*, 262-269.
36. Lamotte, J.; Dupont, L.; Dideberg, O.; Kambu, K.; Angenot, L. Isolation and Structure of Sungucine: a New Type of Bisindoline Alkaloid. *Tet. Lett.* **1979**, *20*, 4227-4228.
37. Aliev, A. E.; Courtier-Murias, D.; Zhou, S. Scaling Factors for Carbon NMR Chemical Shifts Obtained from DFT B3LYP Calculations. *J. Mol. Struct.: THEOCHEM* **2009**, *893*, 1-5.
38. Chmurny, G. N.; Hilton, B. D.; Brobst, S.; Look, S. A.; Witherup, K. M.; Beutler, J. A. ^{1}H- and ^{13}C-NMR Assignments for Taxol, 7-*epi*-Taxol, and Cephalomannine. *J. Nat. Prod.* **1992**, *55*, 414-423.
39. Hohenberg, P.; Kohn, W. Inhomogeneous Electron Gas. *Phys. Rev.* **1964**, *136*, B864-B871.
40. Kohn, W.; Sham, L. J. Self-Consistent Equations Including Exchange and Correlation Effects. *Phys. Rev.* **1965**, *140*, A1133-A1138.
41. Slater, J. C., *Quantum Theory of Molecules and Solids Vol. 4: The Self-Consistent Field for Molecules and Solids*. McGraw-Hill: New York, 1974.
42. Becke, A. D. Density-Functional Exchange-Energy Approximation with Correct Asymptotic Behavior. *Phys. Rev. A* **1988**, *38*, 3098-3100.
43. Perdew, J. P., In *Electronic Structure of Solids '91*, Ziesche, P.; Eschrig, H., Eds. Akademie Verlag: Berlin, 1991; p 11.
44. Perdew, J. P.; Chevary, J. A.; Vosko, S. H.; Jackson, K. A.; Pederson, M. R.; Singh, D. J.; Fiolhais, C. Atoms, Molecules, Solids, and Surfaces: Applications of the

Generalized Gradient Approximation for Exchange and Correlation. *Phys. Rev. B* **1992**, *46*, 6671-6687.
45. Perdew, J. P.; Chevary, J.; Vosko, S.; Jackson, K. A.; Pederson, M. R.; Singh, D.; Fiolhais, C. Erratum: Atoms, Molecules, Solids, and Surfaces: Applications of the Generalized Gradient Approximation for Exchange and Correlation. *Phys. Rev. B* **1993**, *48*, 4978.
46. Perdew, J. P.; Burke, K.; Wang, Y. Generalized Gradient Approximation for the Exchange-Correlation Hole of a Many-Electron System. *Phys. Rev. B* **1996**, *54*, 16533.
47. Adamo, C.; Barone, V. Exchange Functionals with Improved Long-Range Behavior and Adiabatic Connection Methods without Adjustable Parameters: The mPW and mPW1PW Models. *J. Chem. Phys.* **1998**, *108*, 664-675.
48. Perdew, J. P.; Burke, K.; Ernzerhof, M. Generalized Gradient Approximation Made Simple. *Phys. Rev. Lett.* **1996**, *77*, 3865-3868.
49. Perdew, J. P.; Burke, K.; Ernzerhof, M. Erratum: Generalized Gradient Approximation Made Simple. *Phys. Rev. Lett.* **1997**, *78*, 1396.
50. Gill, P. M. A New Gradient-Corrected Exchange Functional. *Mol. Phys.* **1996**, *89*, 433-445.
51. Adamo, C.; Barone, V. Implementation and Validation of the Lacks-Gordon Exchange Functional in Conventional Density Functional and Adiabatic Connection Methods. *J. Comput. Chem.* **1998**, *19*, 418-429.
52. Tao, J.; Perdew, J. P.; Staroverov, V. N.; Scuseria, G. E. Climbing the Density Functional Ladder: Nonempirical Meta-Generalized Gradient Approximation Designed for Molecules and Solids. *Phys. Rev. Lett.* **2003**, *91*, 146401.
53. Perdew, J. P.; Ruzsinszky, A.; Csonka, G. I.; Constantin, L. A.; Sun, J. Workhorse Semilocal Density Functional for Condensed Matter Physics and Quantum Chemistry. *Phys. Rev. Lett.* **2009**, *103*, 026403.
54. Perdew, J. P.; Ruzsinszky, A.; Csonka, G. I.; Constantin, L. A.; Sun, J. Erratum: Workhorse Semilocal Density Functional for Condensed Matter Physics and Quantum Chemistry. *Phys. Rev. Lett.* **2011**, *106*, 179902.
55. Perdew, J. P.; Kurth, S.; Zupan, A.; Blaha, P. Accurate Density Functional with Correct Formal Properties: A Step Beyond the Generalized Gradient Approximation. *Phys. Rev. Lett.* **1999**, *82*, 2544.
56. Becke, A. D.; Roussel, M. R. Exchange Holes in Inhomogeneous Systems: A Coordinate-Space Model. *Physical Review A* **1989**, *39*, 3761-3767.
57. Vosko, S. H.; Wilk, L.; Nusair, M. Accurate Spin-Dependent Electron Liquid Correlation Energies for Local Spin Density Calculations: A Critical Analysis. *Can. J. Phys.* **1980**, *58*, 1200-1211.
58. Perdew, J. P.; Zunger, A. Self-Interaction Correction to Density-Functional Approximations for Many-Electron Systems. *Phys. Rev. B* **1981**, *23*, 5048-5079.
59. Lee, C.; Yang, W.; Parr, R. G. Development of the Colle-Salvetti Correlation-Energy Formula into a Functional of the Electron Density. *Phys. Rev. B* **1988**, *37*, 785-789.
60. Miehlich, B.; Savin, A.; Stoll, H.; Preuss, H. Results Obtained with the Correlation Energy Density Functionals of Becke and Lee, Yang and Parr. *Chem. Phys. Lett.* **1989**, *157*, 200-206.
61. Perdew, J. P. Density-Functional Approximation for the Correlation Energy of the Inhomogeneous Electron Gas. *Phys. Rev. B* **1986**, *33*, 8822-8824.

62. Peverati, R.; Zhao, Y.; Truhlar, D. G. Generalized Gradient Approximation that Recovers the Second-Order Density-Gradient Expansion with Optimized Across-the-Board Performance. *J. Phys. Chem. Lett.* **2011**, *2*, 1991-1997.
63. Hamprecht, F. A.; Cohen, A. J.; Tozer, D. J.; Handy, N. C. Development and Assessment of New Exchange-Correlation Functionals. *J. Chem. Phys.* **1998**, *109*, 6264-6271.
64. Boese, A. D.; Doltsinis, N. L.; Handy, N. C.; Sprik, M. New Generalized Gradient Approximation Functionals. *J. Chem. Phys.* **2000**, *112*, 1670-1678.
65. Boese, A. D.; Handy, N. C. A New Parametrization of Exchange-Correlation Generalized Gradient Approximation Functionals. *J. Chem. Phys.* **2001**, *114*, 5497-5503.
66. Peverati, R.; Truhlar, D. G. Exchange-Correlation Functional with Good Accuracy for Both Structural and Energetic Properties While Depending Only on the Density and its Gradient. *J. Chem. Theory Comput.* **2012**, *8*, 2310-2319.
67. Becke, A. D. Density-Functional Thermochemistry. III. The Role of Exact Exchange. *J. Chem. Phys.* **1993**, *98*, 5648-5652.
68. Adamo, C.; Barone, V. Toward Reliable Adiabatic Connection Models Free from Adjustable Parameters. *Chem. Phys. Lett.* **1997**, *274*, 242-250.
69. Cohen, A. J.; Handy, N. C. Dynamic Correlation. *Mol. Phys.* **2001**, *99*, 607-615.
70. Xu, X.; Goddard III, W. A. The X3LYP Extended Density Functional for Accurate Descriptions of Nonbond Interactions, Spin States, and Thermochemical Properties. *Proc. Natl. Acad. Sci.* **2004**, *101*, 2673-2677.
71. Adamo, C.; Barone, V. Toward Reliable Density Functional Methods without Adjustable Parameters: The PBE0 Model. *J. Chem. Phys.* **1999**, *110*, 6158-6170.
72. Ernzerhof, M.; Scuseria, G. E. Assessment of the Perdew-Burke-Ernzerhof Exchange-Correlation Functional. *J. Chem. Phys.* **1999**, *110*, 5029-5036.
73. Austin, A.; Petersson, G. A.; Frisch, M. J.; Dobek, F. J.; Scalmani, G.; Throssell, K. A Density Functional with Spherical Atom Dispersion Terms. *J. Chem. Theory Comput.* **2012**, *8*, 4989-5007.
74. Wiitala, K. W.; Hoye, T. R.; Cramer, C. J. Hybrid Density Functional Methods Empirically Optimized for the Computation of ^{13}C and ^{1}H Chemical Shifts in Chloroform Solution. *J. Chem. Theory Comput.* **2006**, *2*, 1085-1092.
75. Peverati, R.; Truhlar, D. G. Communication: A Global Hybrid Generalized Gradient Approximation to the Exchange-Correlation Functional that Satisfies the Second-Order Density-Gradient Constraint and has Broad Applicability in Chemistry. *J. Chem. Phys.* **2011**, *135*, 191102.
76. Wilson, P. J.; Bradley, T. J.; Tozer, D. J. Hybrid Exchange-Correlation Functional Determined from Thermochemical Data and *ab initio* Potentials. *J. Chem. Phys.* **2001**, *115*, 9233-9242.
77. Grimme, S. Semiempirical GGA-Type Density Functional Constructed with a Long-Range Dispersion Correction. *J. Comput. Chem.* **2006**, *27*, 1787-1799.
78. Becke, A. D. Density-Functional Thermochemistry. V. Systematic Optimization of Exchange-Correlation Functionals. *J. Chem. Phys.* **1997**, *107*, 8554-8560.
79. Schmider, H. L.; Becke, A. D. Optimized Density Functionals from the Extended G2 Test Set. *J. Chem. Phys.* **1998**, *108*, 9624-9631.
80. Van Voorhis, T.; Scuseria, G. E. A Novel Form for the Exchange-Correlation Energy Functional. *J. Chem. Phys.* **1998**, *109*, 400-410.

81. Boese, A. D.; Handy, N. C. New Exchange-Correlation Density Functionals: The Role of the Kinetic-Energy Density. *J. Chem. Phys.* **2002**, *116*, 9559-9569.
82. Zhao, Y.; Truhlar, D. G. A New Local Density Functional for Main-Group Thermochemistry, Transition Metal Bonding, Thermochemical Kinetics, and Noncovalent Interactions. *J. Chem. Phys.* **2006**, *125*, 194101.
83. Peverati, R.; Truhlar, D. G. M11-L: A Local Density Functional that Provides Improved Accuracy for Electronic Structure Calculations in Chemistry and Physics. *J. Phys. Chem. Lett.* **2012**, *3*, 117-124.
84. Peverati, R.; Truhlar, D. G. An Improved and Broadly Accurate Local Approximation to the Exchange-Correlation Density Functional: The MN12-L Functional for Electronic Structure Calculations in Chemistry and Physics. *Phys. Chem. Chem. Phys.* **2012**, *14*, 13171-13174.
85. Yu, H. S.; He, X.; Truhlar, D. G. MN15-L: A New Local Exchange-Correlation Functional for Kohn-Sham Density Functional Theory with Broad Accuracy for Atoms, Molecules, and Solids. *J. Chem. Theory Comput.* **2016**, *12*, 1280-1293.
86. Yanai, T.; Tew, D. P.; Handy, N. C. A New Hybrid Exchange-Correlation Functional Using the Coulomb-Attenuating Method (CAM-B3LYP). *Chem. Phys. Lett.* **2004**, *393*, 51-57.
87. Vydrov, O. A.; Scuseria, G. E. Assessment of a Long-Range Corrected Hybrid Functional. *J. Chem. Phys.* **2006**, *125*, 234109.
88. Vydrov, O. A.; Heyd, J.; Krukau, A. V.; Scuseria, G. E. Importance of Short-Range Versus Long-Range Hartree-Fock Exchange for the Performance of Hybrid Density Functionals. *J. Chem. Phys.* **2006**, *125*, 074106.
89. Vydrov, O. A.; Scuseria, G. E.; Perdew, J. P. Tests of Functionals for Systems with Fractional Electron Number. *J. Chem. Phys.* **2007**, *126*, 154109.
90. Henderson, T. M.; Izmaylov, A. F.; Scalmani, G.; Scuseria, G. E. Can Short-Range Hybrids Describe Long-Range-Dependent Properties? *J. Chem. Phys.* **2009**, *131*, 044108.
91. Chai, J.-D.; Head-Gordon, M. Long-Range Corrected Hybrid Density Functionals with Damped Atom-Atom Dispersion Corrections. *Phys. Chem. Chem. Phys.* **2008**, *10*, 6615-6620.
92. Chai, J.-D.; Head-Gordon, M. Long-Range Corrected Double-Hybrid Density Functionals. *J. Chem. Phys.* **2009**, *131*, 174105.
93. Henderson, T. M.; Izmaylov, A. F.; Scuseria, G. E.; Savin, A. Assessment of a Middle-Range Hybrid Functional. *J. Chem. Theory Comput.* **2008**, *4*, 1254-1262.
94. Heyd, J.; Scuseria, G. E. Assessment and Validation of a Screened Coulomb Hybrid Density Functional. *J. Chem. Phys.* **2004**, *120*, 7274-7280.
95. Heyd, J.; Scuseria, G. E. Efficient Hybrid Density Functional Calculations in Solids: Assessment of the Heyd-Scuseria-Ernzerhof Screened Coulomb Hybrid Functional. *J. Chem. Phys.* **2004**, *121*, 1187-1192.
96. Heyd, J.; Peralta, J. E.; Scuseria, G. E.; Martin, R. L. Energy Band Gaps and Lattice Parameters Evaluated with the Heyd-Scuseria-Ernzerhof Screened Hybrid Functional. *J. Chem. Phys.* **2005**, *123*, 174101.
97. Heyd, J.; Scuseria, G. E.; Ernzerhof, M. Erratum: "Hybrid Functionals Based on a Screened Coulomb Potential". *J. Chem. Phys.* **2006**, *124*, 219906.

98. Izmaylov, A. F.; Scuseria, G. E.; Frisch, M. J. Efficient Evaluation of Short-Range Hartree-Fock Exchange in Large Molecules and Periodic Systems. *J. Chem. Phys.* **2006**, *125*, 104103.
99. Krukau, A. V.; Vydrov, O. A.; Izmaylov, A. F.; Scuseria, G. E. Influence of the Exchange Screening Parameter on the Performance of Screened Hybrid Functionals. *J. Chem. Phys.* **2006**, *125*, 224106.
100. Peverati, R.; Truhlar, D. G. Screened-Exchange Density Functionals with Broad Accuracy for Chemistry and Solid-State Physics. *Phys. Chem. Chem. Phys.* **2012**, *14*, 16187-16191.
101. Becke, A. D. Density-Functional Thermochemistry. IV. A New Dynamical Correlation Functional and Implications for Exact-Exchange Mixing. *J. Chem. Phys.* **1996**, *104*, 1040-1046.
102. Staroverov, V. N.; Scuseria, G. E.; Tao, J.; Perdew, J. P. Comparative Assessment of a New Nonempirical Density Functional: Molecules and Hydrogen-Bonded Complexes. *J. Chem. Phys.* **2003**, *119*, 12129-12137.
103. Zhao, Y.; Truhlar, D. G. Design of Density Functionals that are Broadly Accurate for Thermochemistry, Thermochemical Kinetics, and Nonbonded Interactions. *J. Phys. Chem. A* **2005**, *109*, 5656-5667.
104. Zhao, Y.; Schultz, N. E.; Truhlar, D. G. Exchange-Correlation Functional with Broad Accuracy for Metallic and Nonmetallic Compounds, Kinetics, and Noncovalent Interactions. *J. Chem. Phys.* **2005**, *123*, 161103.
105. Zhao, Y.; Schultz, N. E.; Truhlar, D. G. Design of Density Functionals by Combining the Method of Constraint Satisfaction with Parametrization for Thermochemistry, Thermochemical Kinetics, and Noncovalent Interactions. *J. Chem. Theory Comput.* **2006**, *2*, 364-382.
106. Zhao, Y.; Truhlar, D. G. The M06 Suite of Density Functionals for Main Group Thermochemistry, Thermochemical Kinetics, Noncovalent Interactions, Excited States, and Transition Elements: Two New Functionals and Systematic Testing of Four M06-Class Functionals and 12 Other Functionals. *Theor. Chem. Acc.* **2008**, *120*, 215-241.
107. Zhao, Y.; Truhlar, D. G. Density Functional for Spectroscopy: No Long-Range Self-Interaction Error, Good Performance for Rydberg and Charge-Transfer States, and Better Performance on Average than B3LYP for Ground States. *J. Phys. Chem. A* **2006**, *110*, 13126-13130.
108. Zhao, Y.; Truhlar, D. G. Comparative DFT Study of van der Waals Complexes: Rare-Gas Dimers, Alkaline-Earth Dimers, Zinc Dimer, and Zinc-Rare-Gas Dimers. *J. Phys. Chem. A* **2006**, *110*, 5121-5129.
109. Zhao, Y.; Truhlar, D. G. Exploring the Limit of Accuracy of the Global Hybrid Meta Density Functional for Main-Group Thermochemistry, Kinetics, and Noncovalent Interactions. *J. Chem. Theory Comput.* **2008**, *4*, 1849-1868.
110. Haoyu, S. Y.; He, X.; Li, S. L.; Truhlar, D. G. MN15: A Kohn-Sham Global-Hybrid Exchange-Correlation Density Functional with Broad Accuracy for Multi-Reference and Single-Reference Systems and Noncovalent Interactions. *Chem. Sci.* **2016**, *7*, 5032-5051.
111. Peverati, R.; Truhlar, D. G. Improving the Accuracy of Hybrid Meta-GGA Density Functionals by Range Separation. *J. Phys. Chem. Lett.* **2011**, *2*, 2810-2817.

112. Ditchfield, R.; Hehre, W. J.; Pople, J. A. Self-Consistent Molecular-Orbital Methods. IX. An Extended Gaussian-Type Basis for Molecular-Orbital Studies of Organic Molecules. *J. Chem. Phys.* **1971**, *54*, 724-728.
113. Dobbs, K.; Hehre, W. Molecular Orbital Theory of the Properties of Inorganic and Organometallic Compounds 4. Extended Basis Sets for Third- and Fourth-Row, Main-Group Elements. *J. Comput. Chem.* **1986**, *7*, 359-378.
114. Gordon, M. S.; Binkley, J. S.; Pople, J. A.; Pietro, W. J.; Hehre, W. J. Self-Consistent Molecular-Orbital Methods. 22. Small Split-Valence Basis Sets for Second-Row Elements. *J. Am. Chem. Soc.* **1982**, *104*, 2797-2803.
115. Hariharan, P. C.; Pople, J. A. The Influence of Polarization Functions on Molecular Orbital Hydrogenation Energies. *Theor. Chim. Acta* **1973**, *28*, 213-222.
116. Hehre, W. J.; Ditchfield, R.; Pople, J. A. Self-Consistent Molecular Orbital Methods. XII. Further Extensions of Gaussian-Type Basis Sets for use in Molecular Orbital Studies of Organic Molecules. *J. Chem. Phys.* **1972**, *56*, 2257-2261.
117. McLean, A.; Chandler, G. Contracted Gaussian Basis Sets for Molecular Calculations. I. Second Row Atoms, Z = 11–18. *J. Chem. Phys.* **1980**, *72*, 5639-5648.
118. Pietro, W. J.; Francl, M. M.; Hehre, W. J.; DeFrees, D. J.; Pople, J. A.; Binkley, J. S. Self-Consistent Molecular Orbital Methods. 24. Supplemented Small Split-Valence Basis Sets for Second-Row Elements. *J. Am. Chem. Soc.* **1982**, *104*, 5039-5048.
119. Binkley, J. S.; Pople, J. A.; Hehre, W. J. Self-Consistent Molecular Orbital Methods. 21. Small Split-Valence Basis Sets for First-Row Elements. *J. Am. Chem. Soc.* **1980**, *102*, 939-947.
120. Clark, T.; Chandrasekhar, J.; Spitznagel, G. W.; Schleyer, P. V. R. Efficient Diffuse Function-Augmented Basis Sets for Anion Calculations. III. The 3-21+G Basis Set for First-Row Elements, Li-F. *J. Comput. Chem.* **1983**, *4*, 294-301.
121. Frisch, M. J.; Pople, J. A.; Binkley, J. S. Self-Consistent Molecular Orbital Methods 25. Supplementary Functions for Gaussian Basis Sets. *J. Chem. Phys.* **1984**, *80*, 3265-3269.
122. Dunning Jr, T. H. Gaussian Basis Sets for use in Correlated Molecular Calculations. I. The Atoms Boron Through Neon and Hydrogen. *J. Chem. Phys.* **1989**, *90*, 1007-1023.
123. Kendall, R. A.; Dunning Jr, T. H.; Harrison, R. J. Electron Affinities of the First-Row Atoms Revisited. Systematic Basis Sets and Wave Functions. *J. Chem. Phys.* **1992**, *96*, 6796-6806.
124. Papajak, E.; Zheng, J.; Xu, X.; Leverentz, H. R.; Truhlar, D. G. Perspectives on Basis Sets Beautiful: Seasonal Plantings of Diffuse Basis Functions. *J. Chem. Theory Comput.* **2011**, *7*, 3027-3034.
125. Schäfer, A.; Horn, H.; Ahlrichs, R. Fully Optimized Contracted Gaussian Basis Sets for Atoms Li to Kr. *J. Chem. Phys.* **1992**, *97*, 2571-2577.
126. Schäfer, A.; Huber, C.; Ahlrichs, R. Fully Optimized Contracted Gaussian Basis Sets of Triple Zeta Valence Quality for Atoms Li to Kr. *J. Chem. Phys.* **1994**, *100*, 5829-5835.
127. Weigend, F.; Ahlrichs, R. Balanced Basis Sets of Split Valence, Triple Zeta Valence and Quadruple Zeta Valence Quality for H to Rn: Design and Assessment of Accuracy. *Phys. Chem. Chem. Phys.* **2005**, *7*, 3297-3305.
128. Weigend, F. Accurate Coulomb-Fitting Basis Sets for H to Rn. *Phys. Chem. Chem. Phys.* **2006**, *8*, 1057-1065.
129. Barone, V., Structure, Magnetic Properties and Reactivities of Open-Shell Species from Density Functional and Self-Consistent Hybrid Methods. In *Recent Advances in Density*

Functional Methods, Part I, Chong, D. P., Ed. World Scientific Publishing Co.: Singapore, 1996.
130. Easton, R. E.; Giesen, D. J.; Welch, A.; Cramer, C. J.; Truhlar, D. G. The MIDI! Basis Set for Quantum Mechanical Calculations of Molecular Geometries and Partial Charges. *Theoretica chimica acta* **1996**, *93*, 281-301.
131. Dunning, T. H.; Hay, P. J., Gaussian Basis Sets for Molecular Calculations. In *Methods of Electronic Structure Theory. Modern Theoretical Chemistry*, Schaefer, H. F., Ed. Springer: Boston, 1977; Vol. 3.
